# Obesity-driven phosphatidylethanolamine dysregulation impairs neuroimmune crosstalk and accelerates Alzheimer’s pathogenesis

**DOI:** 10.1186/s13024-026-00943-3

**Published:** 2026-04-15

**Authors:** Li Yang, Jianting Sheng, Shaohua Qi, Zheng Yin, Michael Chan, Yuliang Cao, Hong Zhao, Zhihao Wan, Bill Chan, Ju Young Ahn, Xiaohui Yu, Matthew Vasquez, Shan Xu, Xianlin Han, Weiming Xia, Willa A. Hsueh, Stephen T. C. Wong

**Affiliations:** 1 Ting Tsung & Wei Fong Chao Center for BRAIN, Houston Methodist Academic Institute and Weill Cornell Medicine, Houston, TX 77030 USA; 2 Systems Medicine and Bioengineering Department, Houston Methodist Neal Cancer Center, Houston Methodist Hospital, Houston, TX 77030 USA; 3 Departments of Pathology and Laboratory Medicine and Radiology, Houston Methodist Hospital and Weill Cornell Medicine, Houston, TX 77030 USA; 4 Barshop Institute for Longevity and Aging Studies, and Department of Medicine, University of Texas Health Science Center at San Antonio, San Antonio, TX 78229 USA; 5 Department of Pharmacology, Physiology & Biophysics, Boston University Chobanian & Avedisian School of Medicine, Boston, MA 02118 USA; 6 Geriatric Research Education and Clinical Center, Bedford VA Healthcare System, Bedford, MA 01730 USA; 7 Diabetes and Metabolism Research Center, Division of Endocrinology, Diabetes & Metabolism, Department of Internal Medicine, The Ohio State University Wexner Medical Center, Columbus, OH 43210 USA

**Keywords:** Phosphatidylethanolamine, Obesity, Lipid homeostasis, Alzheimer’s disease, Cognitive restoration, Neuroimmune crosstalk, Dynamic membrane remodeling

## Abstract

**Background:**

Midlife obesity is a major modifiable risk factor for Alzheimer’s disease (AD), yet the lipid-mediated mechanisms linking peripheral metabolic dysfunction to brain pathology remain poorly understood. In particular, how adipose-derived lipid perturbations influence immune and neuronal compartments in the brain has not been fully elucidated.

**Methods:**

We employed an integrative multi-omics approach combining quantitative lipidomics, single-nucleus RNA sequencing, proteomics, and high-resolution imaging to characterize the metabolic alterations associated with obesity in both peripheral and central tissues. Functional assessments were performed in AD mouse models to evaluate neuroimmune responses and behavioral outcomes. Statistical analyses were performed using appropriate univariate and multivariate methods, with multiple testing correction applied where applicable.

**Results:**

We identified elevated phosphatidylethanolamine (PE) abundance as a metabolic hallmark of obesity. Excess PE accumulation led to disrupted lipid homeostasis and ectopic lipid droplet deposition in the brain, resulting in functional exhaustion of T cells, impaired microglial identity and signaling, and enhanced amyloidogenic processing in excitatory neurons. These effects were linked by membrane remodeling as a unifying structural mechanism. Pharmacological targeting of PE homeostasis using the redox-active compound ebselen ameliorated lipid dysregulation, restored neuroimmune function, and improved cognitive performance in AD models.

**Conclusions:**

Our study reveals a critical role for PE in coordinating immune-neuronal crosstalk under metabolic stress. These findings suggest that lipid remodeling serves as a structural nexus linking obesity to AD progression, and support the potential of lipid-directed interventions as therapeutic strategies for metabolic-risk-associated neurodegeneration.

**Supplementary information:**

The online version contains supplementary material available at 10.1186/s13024-026-00943-3.

## Introduction

Emerging evidence indicates that midlife obesity is the most prominent modifiable risk factor for dementia, particularly for Alzheimer’s disease (AD) and related dementias (ADRD), with hazard ratios (HRs) for incidence ranging from 1.17 to 1.34 [1–4]. Notably, midlife obesity in women is associated with a 39% increased risk of developing dementia compared to age-matched non-obese women [3]. The lack of effective pharmacological interventions targeting lipid homeostasis has prompted the search for novel lipid species and signaling mechanisms involved in AD.

Weight loss interventions targeting obesity can improve memory and executive function [5–7]. In studies on transgenic mouse models of AD, e.g., 5XFAD mice, a high fat diet (HFD) increases cerebral β-amyloid (Aβ) deposition, impairs cognitive function, and elevates hippocampal oxidative stress [8–11]. Similar findings have also been reported in wildtype and other mouse models of obesity or AD [12–16]. Mechanisms linking the HFD/obesity and AD pathology have implicated lipids and lipotoxicity, insulin resistance, inflammation, adipokines, and immune cell fate shift [17–19]; all are enhanced in individuals with obesity.

The novelty of this study lies in its exploration of how the ontology of lipid fluxes, beyond merely disrupting metabolic homeostasis, contributes to intracellular events and extracellular crosstalk of neuro- pathologies. Our study systematically explores the interplay between obesity and AD within a unified multi-omics framework, identifying phosphatidylethanolamine overload (PE^high^) as a potential mechanistic driver of AD. PE, a key glycerophospholipid that accounts for 15–25% of total lipid content in eukaryotic cells, plays a crucial role in essential cellular processes, including autophagy, mitophagy, phosphatidylcholine synthesis, oxidative phosphorylation, membrane topology maintenance, membrane fusion at cellular and organelle levels, mitochondrial biogenesis, ferroptosis, and post-translational modifications [20–23]. Dysregulation of these pathways contributes to pathological conditions such as neuronal dysfunction [24].

The brain is a highly lipid-rich organ. Single-cell genomics uncovered data have been applied to categorize the five major signaling pathways participating in AD etiology, of which lipid signaling and metabolism (lipid handling) is highlighted across cell-type-specific molecular perturbations [25].

Among all the brain cell population, in neurons, amyloid precursor protein (APP) localizes to detergent-soluble microdomains enriched in PE and phosphatidylcholine (PC), where the production of toxic Aβ is closely associated with the internal distribution equilibrium of its cleaving machinery, including β- and γ-secretases, as well as the fluidity of the membrane system [26]. A loss of PE function promotes APP α-cleavage, potentially disrupting the spatial proximity of APP and β-secretase, thereby shifting the process toward a non-amyloidogenic pathway [20, 26]. Despite these findings, detailed mechanistic studies focusing on the role of PE in AD models remain sparse. Key unexplored areas include its potential to activate nuclear receptor-related cascades, its role in lipid dyshomeostasis linked to lipid droplet characterization, and its involvement in endomembrane remodeling.

Resident microglia and infiltrating T cells are key immune populations involved in neuroinflammatory processes linked to dysregulated brain lipid metabolism. In particular, disrupted lipid homeostasis—such as ectopic lipid droplet accumulation in microglia—can drive these cells into a dysfunctional and proinflammatory state [27]. Neurodegeneration further promotes T cell infiltration into affected brain regions [28], and microglia have been shown to mediate the recruitment of cytotoxic CD8^+^ T cells, contributing to tau pathology [29]. Notably, blockade of immune checkpoints such as PD-1/PD-L1 can reduce tauopathy [29–31], suggesting that stem-like T cells—characterized by self-renewal and effector potential—may sustain chronic immune activation and promote long-term inflammatory damage. In contrast, exhausted T cells, defined by high expression of inhibitory receptors (e.g., PD-1, LAG-3, TIM-3), may exert either protective or detrimental effects depending on the extent of dysfunction and the immune context.

However, a comprehensive understanding of how lipid flux drives cell-type-specific changes and neuroimmune crosstalk, particularly in obesity-associated AD, remains limited. In this study, we show that excessive PE abundance, as a metabolic hallmark of midlife obesity, acts as a key driver of AD pathology. Through integrated lipidomics, proteomics, drug repositioning, multi-modal imaging, and biochemical analyses, we demonstrate that elevated PE flux selectively disrupts brain lipid homeostasis, impairs immune competence in microglia and T cells, and promotes the ectopic accumulation of neurotoxic proteins. These changes reflect a coordinated remodeling of metabolic, immunologic, and membrane-associated pathways in a cell-type-specific manner. In this study, PE abundance refers to steady-state PE levels measured in human adipose and brain tissues. In contrast, we use the term “EV-mediated PE flux” (hereafter referred to as PE flux) to denote the net systemic movement of PE across tissues, as modeled by extracellular vesicle–mediated lipid transfer. This definition reflects lipid transport dynamics at the systems level rather than a directly measured kinetic rate. Our findings provide mechanistic insights into how obesity reshapes neuroimmune plasticity to accelerate AD progression and suggest that restoring PE homeostasis may represent a novel lipid-targeted strategy for therapeutic intervention in AD.

The evidence presented here emphasizes the substantial clinical relevance of PE. First, public data analysis reveals that enzymes involved in PE metabolism are closely linked to clinical outcomes in AD. Second, elevated PE levels are shown to drive hallmark pathological changes in AD, such as amyloidosis, and immune suppression of microglia and T cells, demonstrating the potential of targeting PE metabolic pathways in therapeutic strategies for AD in the obese populations. This approach could be particularly effective as a pre-treatment strategy or neoadjuvant for obese individuals, providing a more precise and early intervention.

## Methods

### Human subjects

This study was approved by the Institutional Review Board (IRB) of Houston Methodist Research Institute and the Ohio State University. All participants provided written informed consent. The research complies with all relevant ethical principles.

**Clinical adipose tissue and blood sample harvest:** Subcutaneous adipose tissue (SQA) and visceral adipose tissue (VA) were obtained from 6 non-obese and 6 obese patients undergoing elective abdominal surgery at the Ohio State University Wexner Medical Center. The criteria for surgical patient exclusion included fever or infection, current smokers, renal or neoplastic disease, history of prior organ transplantation, steroid use, estrogen replacement, AIDS, chronic anti-inflammatory use, chemotherapy within the prior year, more than 10% body weight change within the past 3 months, previously diagnosis of Type-1 diabetes, immunocompromised, hemochromatosis, diagnosed with cancers, or lipodystrophy. Subjects were measured for body weight, body mass index (BMI), blood pressure, waist circumference, fasting plasma glucose, triglycerides, high density lipoprotein (HDL), and insulin at the Ohio State University Wexner Medical Center. Fresh SQA and VA were biopsied at the incision site near the umbilicus of the donors, then flash frozen in liquid nitrogen and stored at −80 °C until further analysis for lipidomics, or immediately transferred to ice-cold saline, rapidly processed for adipocyte isolation, and then forwarded to transcriptome study.

### Mice

All animal protocols were approved by the Institutional Animal Care and Use Committee at Houston Methodist Research Institute, and/or the Ohio State University Wexner Medical Center. The protocols were also in compliance with National Institutes of Health guidelines. All mice were bred/housed as 2–5 per cage in the indicated animal facilities under standard pathogen-free conditions with 12 h light/12 h dark cycles (24-hour LD cycles). The mice had free access to the chow diets and water in the indicated facility rooms equipped with control for temperature (21 ± 1.5°C) and humidity (50 ± 10%).

C57B/6J and hereditary AD transgenic (3xTg and 5XFAD) mouse models imported from Jackson Laboratory (JAX lab, Bar Harbor, Maine) were housed at the animal facility of Houston Methodist Research Institute. 3xTg and 5XFAD mice were genotyped following the protocols of JAX lab. The AD mouse models were maintained for extracellular vesicle administration, drug screening, and behavior assessments. Animals were randomly assigned to experimental groups prior to treatment or intervention. Investigators were blinded to group allocation during behavioral testing, histological scoring, and image quantification whenever feasible.

The high fat diet fed C57B/6J mice, and the control group for extracellular vesicle isolation from adipocytes were obtained from Jackson Laboratory and housed at the animal facility of Ohio State University Wexner Medical Center. 8 ~ 12-week-old mice were fed an HFD (Research Diets, D12492, with 60% kcal from fat), while the control group received a standard control diet (Research Diets, D12450B-as chow diet) for 12 ~ 14 weeks. Body weight was tracked weekly to monitor weight gain over time. After 3 months on the HFD, fasting glucose and insulin sensitivity tests were conducted to confirm significant metabolic differences between the HFD and control groups.

### Cells

For all the cell culture systems, 1% (v/v) penicillin/streptomycin/amphotericin-B solution was involved in the culture medium and cell models were maintained 37 °C with a humidified atmosphere containing 5% CO_2_. Table S6 provided all the reagent and resource information applied in this study.

**T cell hybridoma cell model:** Murine DO-11–10 was a suspension cell model, which was cultured in RPMI-1640 medium with 10% FBS and 1% HEPES.

**SH-SY5Y cells:** a human neuroblastoma cell line, were cultured in a 1:1 mixture of Eagle’s Minimum Essential Medium (EMEM) and F-12 medium supplemented with 10% fetal bovine serum (FBS) and 1% penicillin–streptomycin. For ebselen mechanism studies, SH-SY5Y cells were treated with Bay 11–7082 (1 µM), lipopolysaccharide (LPS, 1 µg/mL), insulin (100 nM), fatostatin hydrobromide (20 µM), ebselen (10 µM), ebsulfur (10 µM), or hydrogen peroxide (100 µM), either individually or in combination, for 24 hours at the indicated doses.

**RAW 264.7 cells:** a murine macrophage cell line, were cultured in Dulbecco’s Modified Eagle’s Medium (DMEM) supplemented with 10% fetal bovine serum (FBS) and 1% penicillin–streptomycin.

EVs isolated from mouse adipocyte tissue (3.5 × 10^10^ particles) were gently mixed with DiIC18(3) (DiI) in PBS at a 1:1000 dilution and incubated for 30 min at room temperature in the dark. Following incubation, labeled EVs were washed once with PBS, passed through a 0.22 µm filter to remove dye aggregates, and pelleted by ultracentrifugation at 100,000 × g for 2 h at 4 °C to remove unbound dye. The EV pellet was resuspended in PBS for downstream applications. RAW 264.7 cells cultured on coverslips were switched to exosome-free growth medium and pre-treated with oleic acid (200 µM) to induce lipid droplet formation. After removal of the oleic acid–containing medium, cells were treated with DiI-labeled mouse-derived EVs at the indicated doses (1× = 1 × 10^10^ particles) for 24 h. A dye-only control, consisting of DiI subjected to the same labeling, washing, and ultracentrifugation procedures in the absence of EVs, or an equivalently processed post-ultracentrifugation supernatant labeled with DiI at a matched volume, was included. Following treatment, cells were processed for further immunofluorescence staining and fluorescence imaging.

**Primary human neuron cell models:** Alzheimer’s in a dish^TM^ clonal FAD ReNcell^®^ VM human neural stem cells were commercially available from EMD Millipore (Billerica, MA, USA) [32]. The cells were sub-cultured and differentiated according to the manufacturer’s instruction.

**Primary microglia:** Primary microglia were isolated from postnatal day 0–3 (P0–P3) mouse cortices. After careful removal of meninges, brain tissues were minced and digested with 0.125% trypsin at 37 °C for 15 minutes. Trypsin inhibitor (40 μg/mL) and DNase I (250 μg/mL) were added to stop digestion and reduce cell clumping. The tissue was gently triturated and filtered through a 70 μm strainer. Cells were resuspended in DMEM supplemented with 10% FBS and plated in T75 flasks pre-coated with poly-D-lysine. Mixed glial cultures were maintained for 7–10 days at 37 °C in a humidified 5% CO₂ incubator. Once confluent, flasks were shaken at 200–250 rpm for 2 hours at 37 °C to detach microglia growing on top of the astrocyte layer. The floating cells were collected, centrifuged (1,000 g, 5 min), and plated onto poly-D-lysine-coated plates. Culture medium was refreshed after 24 hours, and microglia were used for experiments within 48–72 hours. To activate CX3CL1–CX3CR1, primary microglia were cultured in conditioned medium from primary neurons (C57BL/6 WT) containing soluble CX3CL1, without B27 Plus supplement to avoid microglial growth inhibition.

**Primary hippocampal neurons:** Primary hippocampal neurons were isolated from E17 embryos of the indicated mice. Hippocampi were dissected, enzymatically digested with 0.02% trypsin in HBSS at 37 °C for 3 minutes, and gently triturated through fire-polished pipettes. Cells were filtered through 40 μm mesh, centrifuged at 1,000 rpm for 3 minutes, and resuspended for plating. Neurons (>94% NeuN^+^, <1% GFAP^+^ by DIV8) were seeded on poly-D-lysine-coated substrates. Cultures were maintained in Neurobasal Plus medium supplemented with 25 μM glutamate, 500 μM glutamine, 0.1% penicillin-streptomycin, and 2% B27 Plus, and used for experiments after 7 days in vitro unless noted otherwise. For crosstalk, DO-11–10 T cells were co-cultured for 24 hours with primary neurons pre-treated with Etn (1 mM), using neuron-conditioned medium without Etn.

### Lipidomics analysis

Cellular lipid composition was quantified using a multidimensional mass spectrometry–based shotgun lipidomics strategy [33] Adipocyte samples were first homogenized, and total protein concentration was determined for each sample using a Pierce BCA protein assay. Homogenates corresponding to 0.1 mg of total protein were transferred into glass tubes and supplemented with a defined mixture of lipid class–specific internal standards prior to lipid extraction.

Total lipids were isolated using a chloroform–methanol–based biphasic extraction protocol adapted from the Bligh and Dyer method [34]. The resulting lipid extracts were diluted to a final concentration of approximately 2 pmol/µL for direct infusion analysis. Mass spectrometric measurements were carried out using both a triple quadrupole mass spectrometer (TSQ Altis, Thermo Fisher Scientific, San Jose, CA) and a quadrupole-Orbitrap high-resolution instrument (Q-Exactive, Thermo Fisher Scientific, San Jose, CA), each equipped with an automated nanospray ionization source (TriVersa NanoMate, Advion Biosciences Ltd., Ithaca, NY), as previously established [35].

Spectral data were processed following established principles of shotgun lipidomics, including automated ion peak selection, baseline subtraction, and correction for natural isotope abundance [33, 36, 37]. Quantification was achieved by referencing class-matched internal standards to correct for extraction efficiency and matrix-dependent ionization effects. Batch-to-batch variability was minimized through internal standard normalization, resulting in reproducibility exceeding intraday variance. Final lipid abundances were normalized to protein content and reported as nmol per mg protein. Validation of analytical linearity, signal-to-noise thresholds, and overall workflow performance was performed in accordance with previously described procedures [34, 35].

### Inducement of *de novo* synthesis of PE or PC

Ethanolamine (0.8–8 mM) or choline (0.8–1 mM) was added into the cell culture medium of the indicated cell models to mimic the PE^high^ microenvironment. The indicated cell models were treated with Etn for 24 hours or 7 days before collection for the further assay. The cell viability was measured with Vi-cell^TM^ XR cell viability analyzer (Beckman Coulter).

### PE level assay

The collected cells (~8 ×10^6^) and/or indicated mouse brain were sonicated in 200 ~ 500 µL water containing 5% (v/v) peroxide free triton X-100 at 10 watts for 1 min to ensure the cellular/tissue PE can dissolve into the buffer. PE assay was performed following manufacturer’s instructions with the kit indicated in the Table S6. The protein level of the homogenate was assessed with Pierce^TM^ BCA Protein Assay Kit to normalize the raw PE concentration.

### Total RNA extraction and quantitative PCR (qPCR)

Total RNAs of cells were extracted with the RNeasy Plus Mini Kit (QIAGEN) following the manufacturer’s instructions. The RNA quality control was evaluated by electrophoresis chip with Agilent 2100 Bioanalyzer upon the program of eukaryote total RNA Nano. The RNA integrity number (RIN) of ≥8.7 was qualified. cDNA from each RNA sample was synthesized using the SuperScript^®^ VILO^TM^Master Mix kit (Invitrogen) following manufacturer’s instructions. The quantitative RT-PCR (qPCR) using the indicated primers in the Table S7 and Itaq^TM^ Universal SYBR were carried out on the Bio-Rad CFX96 system (Bio-Rad Laboratories). Results were normalized by the *36b4* gene and calculated upon the 2-^ΔΔCt^ method.

### Exosome-enriched extracellular vesicle (EV) purification and characterization

Exosome-enriched EVs were isolated for characterization and in vivo studies. Adipocytes from the adipose tissue of chow-fed and high fat diet-fed WT C57BL/6 mice were isolated as previously described [38]. The isolated adipocytes were washed and cultured at 37 °C for 16 ~ 20 hours in the maintenance medium (DMEM F-12 + 25 µL insulin ±5% FBS (exosome depleted) + 1% penicillin/streptomycin, 3 mL/well) of 6-well plate. These EVs were designated for the characterization. The adipocyte maintenance process was repeated in the serum-free medium [39] to exclude the lipid influence from FBS. The resulting EVs were used for the in vivo administration and PE level assessment. Gentle shaking was applied to the plate to ensure that the visible adipocyte lumps were excluded. To increase the purification yield and scalability, the exosome-enriched EVs were isolated by Tangential Flow Filtration (TFF) followed by ultrafiltration to yield more concentrated preparations [40]. The concentration reached to 10^12^/mL. The isolation and characterization methods for the exosome-enriched EVs adhered to the International Society for Extracellular Society (ISEV) guidelines [41]. Characterization of the adipose tissue derived EVs were performed by microfluidic resistive pulse sensing (MRPS) for particle size and concentration, immunomagnetic capture for exosomal markers, western blotting to probe for adipocyte and exosomal-enriched markers, and transmission electron microscopy. The isolated EVs were labeled using the ExoGlow™-Vivo EV Labeling Kit for in vivo tracing, following the manufacturer’s protocol.

### Engineered extracellular vesicle generation

Equal numbers of EVs in PBS (~6 × 10^10^), isolated from chow- and HFD-fed adipocytes, were dissolved in 1 mL of chloroform/acetone (1:1, vol/vol). The solution was vortexed for 3 minutes and centrifuged at 14,000 g for 10 minutes to separate the components: DNA/RNA into the aqueous phase, proteins as a pellet at the bottom of the aqueous phase, and phospholipids into the organic phase. The centrifugation facilitated the clear separation of the aqueous and organic phases. The lower hydrophobic layer containing acetone and chloroform was retained, while the upper hydrophilic layer, containing dissolved DNA/RNA and proteins, was discarded. The organic solvent was then evaporated under a gentle nitrogen stream. The total phospholipid content was quantified using thin-layer chromatography, as previously described [42].

For blank engineered EVs, the thin-film hydration method was employed [43–45]. Lipid fractions of EVs from adipocytes (total phospholipids), DSPE-PEG2000-COOH, and additional PE (10:1:0.5 molar ratio) were used to form the lipid matrix. The lipid film was hydrated with PBS, followed by sonication in a water bath ultrasound at 40 °C for 10 minutes. Subsequently, probe sonication was performed at 200 W with a 3-second pulse on and a 2-second interval for 10 minutes. The size of the newly generated EVs was controlled using a mini-extruder set (Avanti, 610,000) equipped with a 100 nm filter. The resulting solution was placed in a dialysis membrane (MWCO: 3000 Da) and stirred in saline overnight to remove unencapsulated lipids.

The blank-EVs were activated using EDC·HCl and NHS under acidic conditions (pH 5.5) with a molar ratio of DSPE-PEG2000-COOH:EDC·HCl:NHS:Transferrin set at 1:40:100:0.5. The pH of the solution was then adjusted to 7.5, and Transferrin was added. The Transferrin-modified EVs were separated from unbound Transferrin using a Sephadex G100 column with saline as the mobile phase.

For Fig. 4 G, EVs isolated from chow-fed adipocytes underwent the same modification process. To generate PE-enriched EVs, additional PE, including 16:0–18:1 PE (Avanti, 850757P), 16:0–20:4 PE (Avanti, 850759C), C18 (Plasm)-18:1 PE (Avanti, 852758P), C18 (Plasm)-20:4 PE (Cayman, 37,137), and/or 18:1 Cy5 PE (Avanti, 810335C, for imaging purposes), was incorporated into the organic phase. The concentration and size of the engineered EVs were monitored using Nanosight over at least 3 days to ensure the stability for mouse administration.

### Extracellular vesicle administration

Each 7 ~ 12-week 5XFAD mouse was intravenously administered with 100 µL phosphate-buffered saline (PBS) or 6.55 X 10^9^ exosome-enriched EVs derived from the adipose tissues of chow and/or high fat diet fed mice. The mouse brains were collected 2 ~ 8 hours or two weeks after tail vein injection. The brain samples were forwarded to mass spectrometry, immunofluorescence staining, and ELISA assay.

### LC-MS/MS analysis

For the proteomics analysis regarding to the sample preparation, LC-MS/MS analysis procedure, and protein identification and quantification of extracellular administrated 5XFAD mice, please refer to [46] for the details.

### Quantification and statistical analysis of lipidomic profiling

Normalized lipid values based on its protein concentration were defined as the lipid raw value for each lipid species. Lipid values from SQA and VA were calculated with the mean or sum values depending on the requirement of the indicated images. Lipidomic profiling was conducted with MetaboAnalyst 5.0 upon the re-normalization of lipid raw data using the algorithm provided by the online software.

### Histology and immunofluorescence (IF)

For mouse brain slides, 10 µm slides were cut from OCT fixed tissue blocks. The slides were washed 3 times with PBS and then fixed with 4% paraformaldehyde for 20 min at room temperature followed by permeabilization with 0.25% Triton X-100 for 8 min or digitonin treatment for 5 min for calcium staining. The slides were blocked with normal donkey serum for 30 min and then indicated primary antibodies were incubated overnight at 4 °C. The second antibodies were applied for 1 hour at room temperature.

PE in the cell models was pre-treated with 0.5 µM duramycin-LC-biotin in PBS containing 1% BSA for 1.5 hours prior to cell collection. 647-conjugated streptavidin in PBS containing 1% BSA (1:1000) was applied for the next step staining. BODIPY^TM^ 558/568-C_12_ was added into the culture medium (1:4000) for overnight incubation to stain the lipid droplets. Detailed information on additional antibody staining and the recommended working concentrations is provided in Table S6.

The images were captured by Olympus Fluo View 3000 confocal microscope and Olympus SLIDEVIEW™ VS200 slide scanner. The quantification was performed using ImageJ software.

### Compound screening

The Alzheimer’s in a dish™ clonal FAD ReNcell® VM human neural stem cells were seeded in 96-well plates. After fully differentiation, each well was treated with a specific compound at a 2 µM concentration. Half of the medium in each well was replaced every 3 ~ 4 days and the compound concentration was maintained at 2 µM. Three weeks later, the treated cells were fixed and subjected to staining with the indicated antibodies, such p-tau in the Table S6. The cells were subjected to biochemical assay or whole well imaging by ImageXpress Micro-high content screening (HCS) system (Molecular Devices Sunnyvale, CA, USA). The imaging readouts were processed and analyzed by NeuriteIQ software mentioned in the Table S6 to assess the neurite characteristics.

The compounds from LOPAC library (1,280 compounds), Tocriscreen Mini library (1,120 compounds), Spectrum Collection (2,320 compounds), Prestwick Chemical library (1,200 compounds), Prestwick Phytochemicals Library (320 compounds), and Prestwick Natural Compounds Library (320 compounds), in whole or in part, conducted the current library for this study. During the screening process, each compound was tested 2 to 4 times, and the average results were used to determine the potential candidates for double-blind manual analysis.

### Candidate drug administration and brain slice preparation

3xTg mice were fed with ebselen or vehicle daily for 6 weeks by oral gavage (P.O) with 60 mg/kg body weight as ebselen^low^ and 300 mg/kg body weight as the ebselen^high^. Two weeks after the behavior test, mice were euthanized using CO_2_ asphyxiation and then transcardially perfused with 0.1 M cold PBS (pH 7.4). Mouse brains were collected. For ebselen long term biosafety assay, heart, kidney, spleen, and liver were dissected out and drop-fixed in 4% paraformaldehyde over night at 4 °C. Drop-fixed samples were transferred to 30% sucrose for 48 h and mounted and frozen in Tissue-Plus OCT compound (Fisher Scientific). These samples were sectioned at 10 μm in thickness using a cryostat (Leica CM1850 UV) and stored in PBS with 0.05% sodium azide at 4 °C for the following staining work.

### Quantification of cytokines with ELISA

The culture medium of T cell hybridoma cell model was collected in the low protein binding microcentrifuge tubes to avoid the loss of target cytokines, and then forwarded to ELISA assay with the indicated TNF⍺, IFNγ, and GZMB ELISA kit listed in the Table S6. The hybridoma T cells were harvested and the protein level was assessed with Pierce^TM^ BCA Protein Assay Kit to normalize the cytokine concentration.

### Immunoassay for the quantification of p-tau with ELISA

The mouse brain tissue administrated with/without EVs was homogenized by Qiagen Tissue Lyser LT, at 50 Hz for 5 minutes with freshly prepared, ice cold 5 M guanidine hydrochloride in Tris-buffered saline (20 mM Tris-HCl, 150 mM NaCl, pH 7.4 TBS) containing 1:100 Halt protease inhibitor cocktail (Thermo Scientific) and 1:100 Phosphatase inhibitor cocktail (Sigma) as lysate buffer. The ratio between brain tissue and lysate buffer was 1:5 (w/v). The overnight rocked homogenate at room temperature was diluted according to the manufacture protocols and spun down at 17,000 g, 4 °C, for 15 minutes. The supernatant was applied to Meso Scale Discovery platform (MSD, Gaithersburg, MD) to perform ELISA following manufacture’s protocol. The specific phosphorylated tau was assessed with the indicated antibodies listed in the Table S6 [46, 47].

### ChIP-qPCR

Chromatin immunoprecipitation (ChIP) was performed using SimpleChIP® Enzymatic Chromatin IP Kit according to the manufacturer’s instructions. Briefly, 4 × 10^6^ SH-SY5Y cells (insulin-pretreated) were fixed with 4% formaldehyde to crosslink protein–DNA interactions, and chromatin was digested into approximately 150–900 bp fragments using micrococcal nuclease. Digested chromatin was immunoprecipitated with anti–SREBP-1 or anti–NF-κB p65 antibodies, with normal rabbit IgG used as a negative control. Following elution and reversal of crosslinks, DNA was purified and analyzed by quantitative PCR (qPCR). Primer sequences are provided in Table S6.

### Enzyme kinetics

Kinase activity was measured using a luminescence-based ADP detection kit according to the manufacturer’s instructions. Kinase reactions were performed in solid white 384-well plates in a total volume of 5 µL at room temperature and contained ETNK (10 nM), ethanolamine (2 mM), ATP (50 µM), and reaction buffer (40 mM Tris-HCl, pH 7.5; 20 mM MgCl₂; 0.1 mg/mL bovine serum albumin). Reactions were incubated for 20 min and terminated by addition of ADP-Glo reagent. Following a 40 min incubation, kinase detection reagent was added, and plates were incubated for an additional 20 min. Luminescence was measured using a Synergy H1 microplate reader with an integration time of 1 s per well.

### Behavior assessments

Morris Water Maze (MWM) contains four training days with four trials and one probe day with one trial. Mice were alternately placed facing different visual cues for each trial in the 23 °C pool made opaque with white paint. The submerged platform was kept approximately 1 cm below the water surface. The mice were placed in pre-warmed cages with heat pads during intervals and after the test to avoid hypothermia. Each trial had a duration of 1 minute, and if a mouse failed to locate the hidden platform within this timeframe, it was allowed to remain on the platform for 5 seconds. All trials were meticulously monitored and analyzed using video tracking software (Noldus Ethovision XT) for determining space-relevant memory. The behavior assessment was executed between 8 am and 12 pm in a blinded fashion.

In the habituating process of novel objective recognition test (NORT), 3 days before the official test, mice were exposed to an empty open field box for 10 minutes each day. On the third day, 3 hours after the empty box exploration, the mice were placed in the same open field box with two identical objects located in two opposite corners. The mice have free access to the objects. The trial was ended when the mice had a total of 30 seconds of object exploration. The whole process was recorded, and mice have habituated with the existing objects during this process. 3 hours later, the mice were tested in the same box with the same way while one of the objects was replaced by a novel object. The trial duration was extended to 10 minutes. The location of the novel object was counterbalanced to minimize possible bias. The mouse behavior was monitored and scored using video tracking software (Noldus Ethovision XT) to evaluate mouse cognitive restoration.

### *Ex vivo* experiment of human CD8^+^ T cells

Naïve human peripheral blood CD8+ T cells, obtained from STEMCELL, were activated using human T-activator CD3/CD28 Dynabeads with human IL-2 at a concentration of 10 ng/mL [48]. Cells were treated with or without Etn for 72 hours, after which viable cells were stained with ER Tracker and a Calcium probe (Table S6) at room temperature for 2 hours. Following staining, cells underwent three washes before imaging. Confocal microscopy was performed using an Olympus FV3000 with a 100X objective lens and an additional 4X digital zoom.

### CX3CR1 recycling assay

To evaluate CX3CR1 surface recycling, primary microglia were isolated from 5XFAD mice via CD11b magnetic-activated cell sorting (MACS). Cells were incubated with biotin-conjugated anti-CX3CR1 antibody (13–6099-82, Thermo Fisher) in ice-cold HBSS containing 2% FBS to label surface receptors. After washing, cells were transferred to 30 °C for 30 minutes to permit internalization. Noninternalized surface receptors were removed by brief proteinase K treatment (1 mg/mL, 3 min, 37°C). A control aliquot was kept on ice, while the rest were returned to 30 °C for an additional 1-hour recycling period. Cells were then chilled, washed, and incubated with PE-conjugated streptavidin (BD Biosciences) to detect recycled surface CX3CR1, followed by flow cytometric analysis as described [49].

### Single-nucleus RNA sequencing analysis

Freshly collected 5XFAD mouse brains treated with PE^low^-EVs and PE^high^-EVs were preserved in OCT at −80 °C. Tissue sections were processed with 10X Genomics kits (Table S6) for nuclear isolation, total RNA extraction, and library preparation, following the manufacturer’s instructions. Sequencing was performed on an Illumina NovaSeq 6000 platform. The dataset has been deposited in the GEO database under accession number GSE280474. Single-nucleus gene expression data from mouse brain tissue were processed using Cell Ranger 7.1.0, with alignment to the mouse genome reference (refdata-gex-mm10-2020-A). Quality control (QC) was conducted using the Seurat pipeline, removing cells with fewer than 200 or more than 2,500 unique features and those with over 3% mitochondrial content. Data normalization was performed with Seurat’s LogNormalize method, which adjusts expression levels by dividing each feature’s count by the total counts per cell, multiplying by a scale factor (default: 10,000), and applying a natural log transformation. Linear scaling was then applied, followed by dimensionality reduction with Principal Component Analysis (PCA). Cell clustering was performed with Seurat’s FindClusters function, using a graph-based method to group cells. For visualization, clusters were projected onto a two-dimensional space using UMAP. Differentially expressed genes (DEGs) were identified for each cluster with the FindMarkers function, and cell types were annotated based on known marker genes listed in Table S4.

### Compass-based snRNA-seq analysis of metabolic states in microglia and excitatory neuron

The Compass algorithmic tool, with default parameters, were applied to metabolic flux modeling with the conducted various Gene Set Enrichment Analyses (GSEAs) using the snRNA-seq dataset. Each GSEA was evaluated for the activities of 99 metabolic subsystems as defined by the Recon2 model. Compass produced a reaction-by-subgroup matrix, recording reaction penalty scores, followed by post-analysis to identify metabolic reactions and subsystems with significantly altered activities. The post-analysis pipeline outlined in the original publication [50] was followed. Briefly, Compass generated raw penalty scores, which were subsequently transformed using the negative natural logarithm to produce activity scores, with higher values indicating elevated metabolic flux/activity. Wilcoxon Rank-Sum tests, adjusted for multiple comparisons using Benjamini-Hochberg correction, identified metabolic reactions with significantly different activity scores across subgroups. Effect sizes of the differences between subgroups were quantified using Cohen’s d.

### RNA velocity analysis

Single-nuclei RNA velocity data were processed using Scanpy (version 1.10.3) and scVelo (version 0.3.2). The input data, stored in.loom format, were imported via the scv.read_loom() function. Key preprocessing steps included gene filtering, where genes with fewer than 20 shared counts were removed to reduce noise, and normalization, followed by the selection of the top 2000 highly variable genes. These steps were executed using the scv.pp.filter_and_normalize() function to ensure consistency in gene expression across cells. UMAP coordinates, previously generated in Seurat, were imported and aligned with the.loom dataset by matching cell IDs. A custom mapping function was employed to reconcile differences in sample ID suffixes, and the UMAP coordinates were embedded into the AnnData object via the obsm[‘X_umap’] slot for downstream analysis. RNA velocity estimation was performed using scv.tl.velocity(), and the neighborhood graph was recomputed using sc.pp.neighbors() to account for local RNA velocity dynamics. Velocity graph estimation was subsequently conducted with scv.tl.velocity_graph(). RNA velocity streams were visualized in UMAP space with scv.pl.velocity_embedding_stream(), where cells were colored according to their Seurat cluster assignments. To examine condition-specific differences, cells were categorized into lean PE^low^-EVs and PE^high^-EVs groups by creating a condition column in the AnnData object. Cells from clusters of interest (clusters 0, 1, 5, 9, 17, 19) were filtered separately for lean and obese conditions. The neighborhood and velocity graphs were then recomputed to visualize dynamics within each cluster. Custom functions were implemented to generate RNA velocity embedding plots with adjustable point sizes and color mapping based on condition. The plots were saved in SVG format using matplotlib, ensuring high-quality outputs suitable for publication. Separate visualizations were generated for lean and obese conditions, highlighting RNA velocity trajectories for the clusters of interest.

All analyses were conducted in a Conda environment using Python 3.10. Key libraries included: Scanpy (version 1.10.3), scVelo (version 0.3.2), and python-igraph (version 0.11.6).

### Statistical analysis

All data are presented as mean ± SEM unless otherwise indicated. Statistical analyses were performed using unpaired or paired two-tailed Student’s t-tests (e.g., Figs. 4D, 6D), one-way ANOVA followed by Tukey’s multiple comparisons test (e.g., Figs. 3F, 5D–E, 6I–K), two-way ANOVA where appropriate (e.g., Fig. S4C, S7H), or non-parametric tests (e.g., Wilcoxon rank-sum test; Fig. S11G) when data distribution assumptions were not met.

**Fig. 1:**
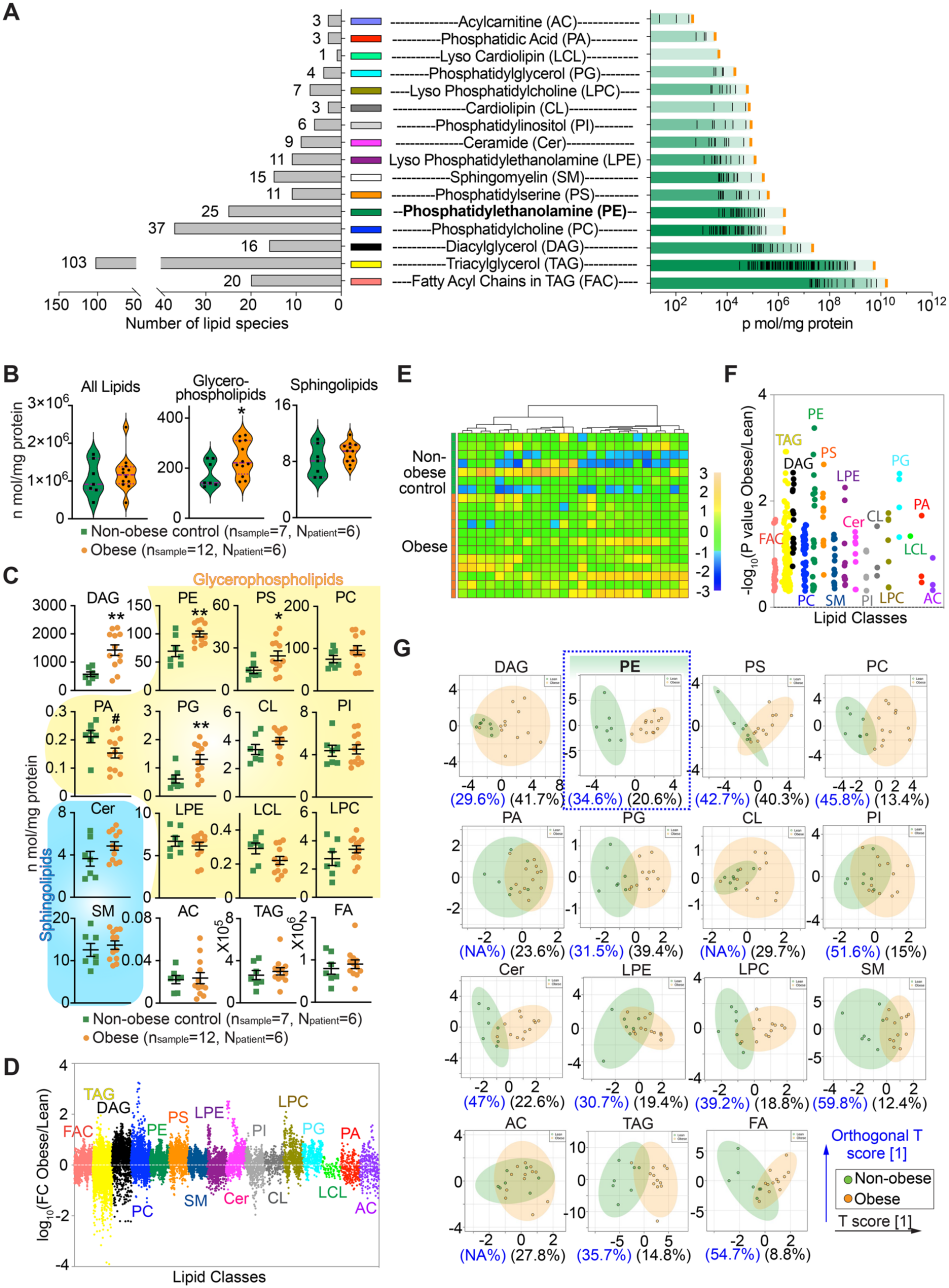
Principal component analysis and classification of lipidomic profiles in human white adipose tissue. **A**. Quantitative distribution of lipid classes from SQA and VA according to their concentration and distribution based on individual lipid species numbers. Left, the concentration of each lipid molecular species is represented by thin black color, and the total lipid class is represented by bold line with orange color. Right, 274 lipid species were detected. **B**. Violin plots compare the mean levels of total lipids, glycerophospholipids, and sphingolipids between non-obese control and obese groups. **C**. Comparison between the mean levels of 16 different lipid classes in the non-obese control and obese individuals. The data represents the sum of both SQA and VA values. **D**. Mirrored Manhattan plot of Fold change of each lipid species upon the comparison between obese and non-obese control individuals. **E**. Heatmap of lipid species distribution of PE. **F**. Simplified Manhattan plot of P values of each lipid classes. **G**. Orthogonal partial least squares discriminant analysis (orthogonal PLS-DA) of 15 different lipid classes in both VA and SQA of non-obese control and obese populations. (**B** and **C**), data are presented as mean ± SEM; *, increase; ^#^, decrease; compared with the corresponding controls; */^#^*p* < 0.05 (**B**) and */^#^*q* < 0.1, **/^##^*q* < 0.05 (**C**). Statistical significance was determined using unpaired two-tailed t-tests (**B**) or two-tailed t-tests followed by Benjamini–Hochberg (BH) correction (**C**)

For high-dimensional datasets (e.g., lipidomics; Fig. 1C; additional examples in Fig. S8D–E), multiple testing correction was applied where appropriate using the Benjamini–Hochberg procedure, and false discovery rate (FDR, q-values) are reported.

Hypothesis-driven or targeted comparisons (e.g., Figs. 4E–I, 6D–G, 7F–J) were evaluated using appropriate statistical tests, generally without multiple testing correction when the number of comparisons was limited and predefined, as detailed in the corresponding figure legends. A P value < 0.05 or FDR-adjusted q value < 0.1 was considered statistically significant.

## Results

### Phosphatidylethanolamine (PE), the second most abundant glycerophospholipid, shows significant elevation in midlife obese individuals

To identify the lipid composition of adipose tissue in obese individuals, most of whom were in midlife, that may be linked to the development of AD, we performed the multidimensional mass spectrometry-based lipidomics analyses on subcutaneous adipose tissue (SQA) and visceral adipose tissue (VA) collected from surgical adipose biopsies of non-obese control and obese donors (Fig. S1A; Table S1). The analyses included 274 lipid species from 16 different lipid classes (Fig. 1A).

Quantification of the lipidome data revealed that all individual lipid classes detected were within a normal physiological or pathological range to guarantee deep profiling (Fig. S1B). Systematic profiling indicated that certain lipid classes significantly increased in the obese group and consistently maintained the same or similar distribution trend between SQA and VA in different individuals (Fig. 1B–D & S1C-S1D), such as phosphatidylethanolamine (PE) (Fig. 1E, yellow color vs. blue color).

The PE class displayed the highest -log_10_ (P value) in all lipid classes (Fig. 1F), indicating that the PE class owned the most outstanding change in the comparison of obese and non-obese groups. Strikingly, we found that PE was the exclusive lipid class that consistently segregated non-obese and obese lipid profiles into non-overlapping clusters (Fig. 1G). Based on the distinctive characteristics of PE, it emerged as a compelling focus among adipose lipid components for our next step studies on neurodegenerative pathologies.

### Identification of top PE species in obese population

The analyses uncovered that enhanced PE level was the unique and important lipid component of the obese adipose tissue, yet it has been less appreciated that whether specific PE species are more prominently altered than the others in obese populations (Fig. 1G). We next focused on the top PE candidate identification.

**Fig. 2:**
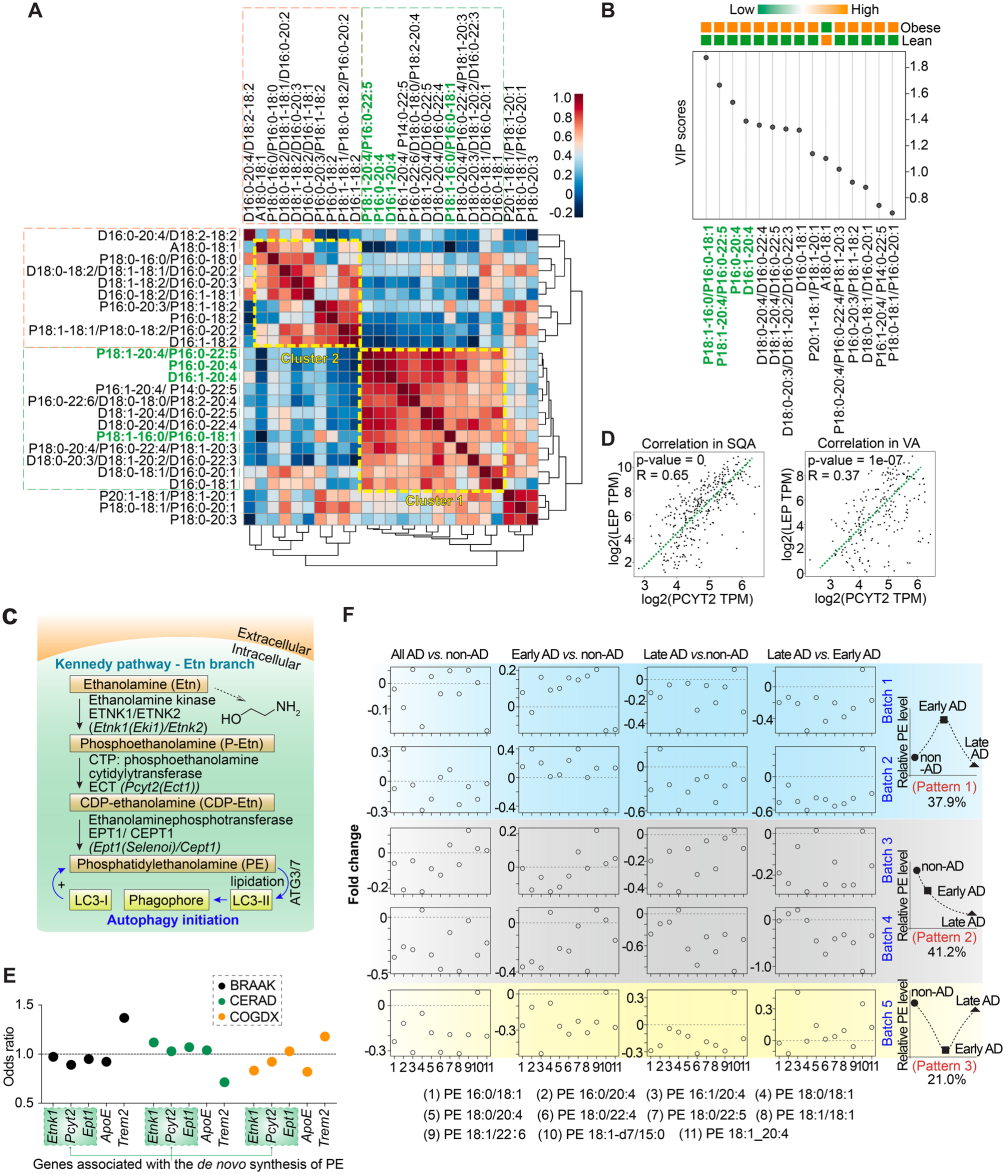
Identification of top PE species altered in obesity and their association with the *de novo* PE synthesis pathway linked to AD. **A**. Correlation heatmap shows the matrix sorted by lipid species of PE and the significantly regulated lipid species between non-obese and obese individuals. Coloring represents the level of correlation between the lipid pairs. **B**. Random Forest analysis shows the top 15 lipid species of PE that leads to differences between non-obese and obese lipid profiles. **C**. Schematic diagram of the molecular pathways involved in *de novo* biosynthesis of PE via ethanolamine branch of Kennedy pathway and LC3 lipidation with PE for phagophore formation to initiate autophagy. **D**. pairwise pearson correlation analysis of LEP and PCYT2 gene expression in SQA and VA. **E**. Association of gene expression levels of *Etnk1*, *Pcyt2*, *Ept1*, *ApoE,* and *Trem2* genes with neuropathology and cognition. Positive associations are represented by an odds ratio > 1 and negative associations by an odds ratio < 1. BRAAK score measures neurofibrillary tangles, CERAD score measures neuritic plaques, and COGDX score measures the final clinical diagnosis and represents the clinical consensus diagnosis. The results are derived from harmonized RNA-seq analysis of post-mortem brains from AD cases and controls. The samples were obtained from three human cohort studies across a total of nine different brain regions. The result is in whole or in part based on data obtained from the AD knowledge portal (https://adknowledgeportal.org). The gene expression levels of *ApoE* and *Trem2* here serve as the positive and negative controls of *Etnk1*, *Pcyt2*, and *Ept1* in the overall dot distribution. **F**. Batch-resolved PE alterations in the ROSMAP cohort across AD stages. Lipidomics data from the emory lipidomics dataset within the ROSMAP cohort were analyzed to compare AD cases with cognitively normal controls. AD status was defined by cognitive diagnosis (COGDX) (AD: COGDX = 4 or 5; control: COGDX = 1). Within the AD group, disease stage was further stratified by Braak neurofibrillary tangle stage, with Braak stages 3–4 classified as early AD and stages 5–6 classified as late AD. Each column represents a pairwise comparison (all AD vs. non-AD, early AD vs. non-AD, late AD vs. non-AD, and late AD vs. Early AD), and each row corresponds to an independent lipidomics batch. Points indicate Fold changes for individual PE species, calculated as the difference in mean log-intensity between groups. The dashed horizontal line denotes zero Fold change. Numbers on the x-axis correspond to PE species listed below the panels. The numbers of early AD, late AD, and control cases for each batch are summarized as follows (values indicate total sample number, with male and female counts in parentheses). Batch 1: early AD, *n* = 10 (3 male, 7 female); late AD, *n* = 8 (1 male, 7 female); control, *n* = 28 (4 male, 24 female). Batch 2: early AD, *n* = 12 (3 male, 9 female); late AD, *n* = 15 (5 male, 10 female); control, *n* = 30 (11 male, 19 female). Batch 3: early AD, *n* = 15 (1 male, 14 female); late AD, *n* = 8 (0 male, 8 female); control, *n* = 38 (12 male, 26 female). Batch 4: early AD, *n* = 15 (4 male, 11 female); late AD, *n* = 8 (2 male, 6 female); control, *n* = 28 (10 male, 18 female). Batch 5: early AD, *n* = 14 (5 male, 9 female); late AD, *n* = 6 (2 male, 4 female); control, *n* = 37 (12 male, 25 female). Batches were additionally grouped by the dominant directional pattern of PE alteration across AD stages, as highlighted by the background colors and schematic summaries on the right. Batch 1–2 (blue, 37.9% of samples) show an early increase followed by a late decrease (pattern 1). Batch 3–4 (grey, 41.2%) show a progressive decrease across stages (pattern 2). Batch 5 (yellow, 21.0%) shows an early decrease with partial late-stage recovery (pattern 3). These schematic plots summarize the major trajectory represented by each batch group

Correlation heatmap depicted co-regulation between all species of PE in human adipose tissue, whereby the two clusters highlighted with yellow dash line represented the PE species with high correlations, especially Cluster 1 (Fig. 2A), indicating that the PE species involved in Cluster 1 were the predominant candidates for top PE identification. We then laid out the PE orders based on their variable importance in projection (VIP) scores (Fig. 2B), of which, the top four PE species also appeared in Cluster 1 of the PE correlation heatmap (Fig. 2A, PE names in green color); these were P18:1–16:0/P16:0–18:1, P18:1–20:4/P16:0–22:5, P16:0–20:4, and D16:1–20:4.

The comparative analysis displayed the PE species of ⍺, β, γ, and δ (Fig. S2A & S2B), which were exclusively identified with significant changes in both SQA and VA groups (Fig. S2C & S2D) and were the same top four PE identified in Fig. 2A–B.

The evidence for the top PE species identification demonstrates that, four eminent PE species beyond the cutoff values in non-obese individuals, which are P18:1–16:0/P16:0–18:1, P18:1–20:4/P16:0–22:5, P16:0–20:4, and D16:1–20:4.

### *De novo* PE synthesis via the CDP-ethanolamine pathway is closely associated with the clinical relevance of obesity and AD

The majority of cellular PE is generated by the *de novo* synthesis of PE through the cytidine diphosphate (CDP)-ethanolamine pathway [51], the ethanolamine (Etn) branch of the Kennedy pathway, in which Etn is the essential precursor (Fig. 2C). The key enzymes, including Etn kinase (ETNK1), phosphoethanolamine cytidylytransferase (PCYT2), and ethanolaminephosphotransferase (EPT1, encoded by *Ept1/Selenoi*) are responsible for the tandem reaction of *de novo* PE synthesis (Fig. 2C).

Notably, PCYT2 is positively correlated with LEPTIN in both SQA and VA tissues (Fig. 2D), confirming the PE-related mechanism was operative in human adipose and linked to obesity. PE *de novo* synthesis enzymes were positively associated with neuritic plaques measured by CERAD score (odds ratio > 1) in post-mortem human AD brain (Fig. 2E). These associations were derived from harmonized RNA-seq analyses of post-mortem human brain tissues across multiple independent cohorts and brain regions. Direct lipidomics analysis of AD brain tissue revealed batch-dependent variability in PE alterations during disease progression [52] (Fig. 2F). In Batch 1 and Batch 2 (Fig. 2F), PE species increased in early AD and decreased in late AD relative to controls, consistent with previously reported dynamic changes in PE during AD progression [53]. In contrast, Batch 3 and Batch 4 exhibited sustained reductions in PE across disease stages. Batch 5 displayed an opposing pattern, with reduced PE levels in early AD and partial increases in late AD. These findings suggest that dysregulated PE metabolism may bridge obesity-related systemic lipid perturbations and AD neuropathology.

Consistent with emerging views that obesity accelerates aging-associated dementias [1–4], our results highlight a potential mechanistic link through excessive PE level in AD. While previous studies have recognized the contribution of lipid dyshomeostasis to AD progression [54], the specific impact of PE dynamics in the context of obesity remains poorly characterized [55]. Building on these observations, we next focused on investigating how PE^high^ flux derived from obesity exacerbates AD-related pathological processes.

### HFD-EVs deliver lipid perturbations to the brain and drive lipid droplet accumulation

**Fig. 3:**
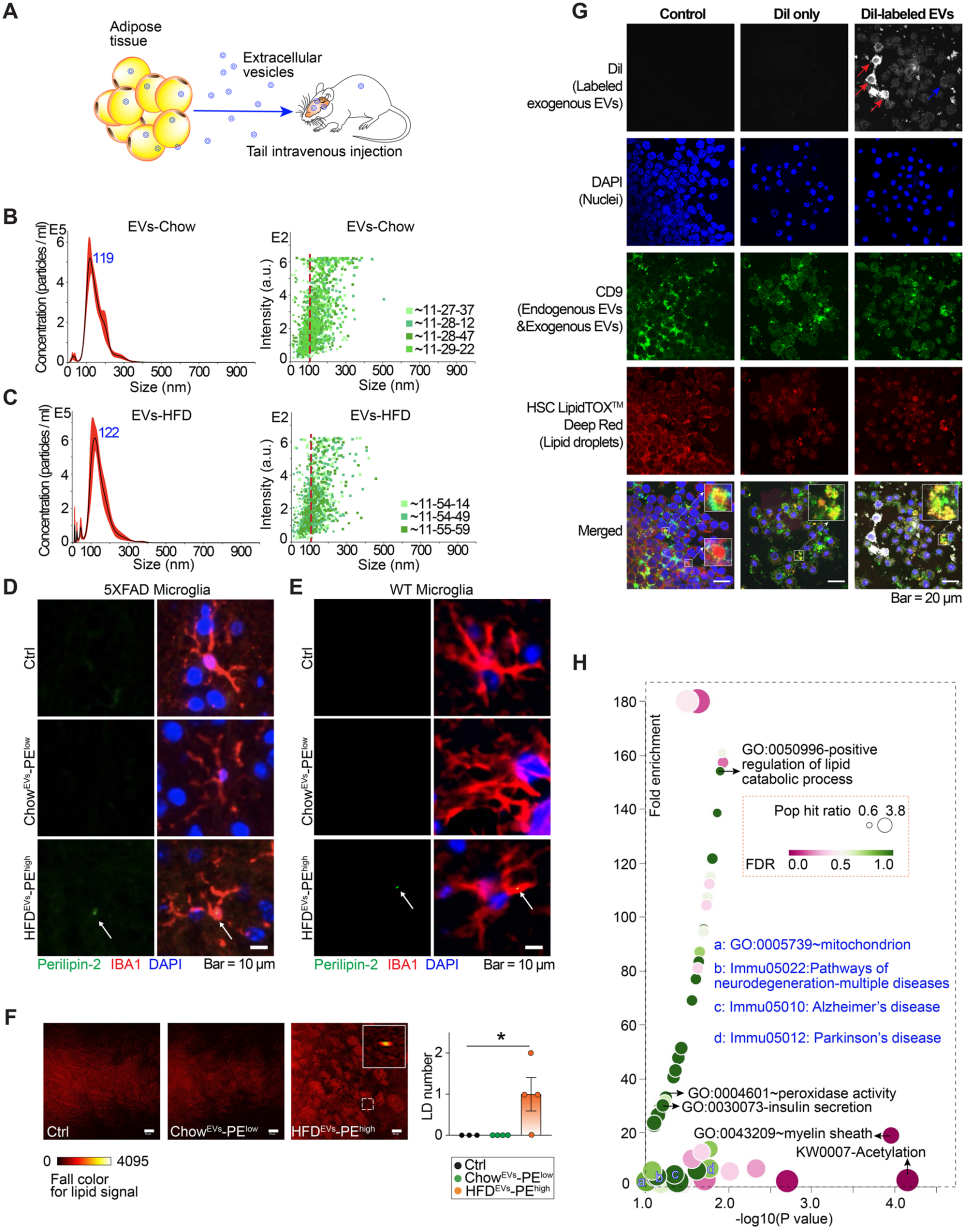
HFD-EVs promote LD accumulation in 5XFAD models. **A**. Schematic diagram of the intravenous administration of white adipose tissue derived extracellular vesicles (EVs) to the transgenic mouse model of AD (5XFAD). **B**-**C**. Quantification and size distribution of EVs from lean and obese adipocytes, assessed by nanoparticle tracking analysis using a NanoSight instrument. **D**-**E**. Representative IF images with ⍺-perilipin-2 antibody, ⍺-IBA1 antibody, and DAPI in the brain section of female 5XFAD and WT littermate mice (7-week) upon the indicated EV administration. Scale bar, 10 µm. **F**. Representative Stimulated Raman Scattering (SRS) microscopy images and the statistics of lipid signal detection in the brain of 7-week-old female 5XFAD mice intravenously administrated with vehicle, Chow^EVs^-PE^low^, and HFD^EVs^-PE^high^. Scale bar, 50 µm. **G**. Representative confocal images illustrating cellular uptake of EVs in RAW 264.7 macrophages. Cells were pretreated with oleic acid to promote lipid droplet formation for visualization. Cells were treated with vehicle control, DiI dye alone, or DiI-labeled EVs. Exogenous EVs were labeled with the lipophilic dye DiI (top row, grayscale). Nuclei were counterstained with DAPI (blue). Endogenous and/or exogenous EV-associated signal was visualized by immunostaining for CD9 (green). Neutral lipid droplets were labeled using HCS LipidTOX™ deep red (red). Merged images show the spatial relationship among DiI-labeled exogenous EVs, nuclei, endogenous EV markers, and lipid droplets. Insets highlight representative regions of interest at higher magnification. Arrows indicate DiI-positive signals associated with exogenous EVs. Scale bar, 20 μm. **H**. Bubble chart of proteomic analysis of pathway signatures in female 5XFAD mouse brain upon HFD^EVs^-PE^high^ administration comparing with the vehicle and Chow^EVs^-PE^low^ administration groups. Pathway selection was based on nominal *p*-values and Fold enrichment, and that the GSEA analysis was intended as an exploratory, hypothesis-generating analysis. (**F**), data are presented as mean ± SEM; *, increase; compared with the control, *p < 0.05. Statistical significance was determined using one-way analysis of variance (ANOVA) followed by Tukey’s multiple comparison test

Extracellular vesicles (EVs) mediate adipose–brain crosstalk (Fig. 3A) [39]. To mimic lipid flux export from adipose tissue to the brain, we isolated high-quality EVs (~100 nm in diameter) from white adipocyte of chow- and HFD-fed mice following rigorous quality control (Fig. 3B–C & Methods). Time-dependent tracking showed that adipocyte-derived EVs displayed efficient biodistribution to the brain, with detectable signals as early as 1 hour and sustained localization at 4 hours post-injection (Fig. S3A). Organ-specific biodistribution confirmed these patterns (Fig. S3B).

Acute administration of HFD-derived EVs led to lipid droplet (LD) formation specifically within cortical microglia of both 5XFAD and WT mice (Fig. 3D–F). Fluorescence-based confocal imaging of DiI-labeled EVs demonstrated uptake of exogenously administered EVs by recipient cells and their close spatial association with lipid droplets (Fig. 3G), supporting a contribution of EV flux to lipid droplet accumulation [56].

Proteomic profiling of EV-treated brains showed that HFD-EVs triggered widespread protein deregulation, which showed a trend of enrichment of several pathways at nominal level with *p* < 0.05, including activation of lipid catabolism, neurodegeneration-related pathways (e.g., Alzheimer’s and Parkinson’s disease), mitochondrial pathways, acetylation pathways, and insulin secretion regulation (Fig. 3H; Tables S2–S3).

These findings demonstrate that HFD-derived EVs can penetrate the brain, disrupt lipid homeostasis, and promote LD accumulation in microglia by reprogramming lipid metabolism, ultimately contributing to neurodegenerative processes. Given the broad metabolic reprogramming observed, along with proteomic alterations in neurodegeneration-associated pathways (Fig. 3H), these results raise the possibility that EV-mediated lipid disturbances may extend to classical AD hallmarks.

The 5XFAD mouse model, being APP- rather than tau-transgenic, exhibits prominent amyloidosis. After acute EV treatment, 5XFAD and age-matched WT mice—modeled here as sporadic AD under obesity—showed increasing trends in amyloidosis-related proteins, though most changes were not statistically significant (Fig. S4A & S4B). These findings prompted us to examine whether PE^high^ flux contributes to the observed amyloidosis burden.

### Isolating the role of PE flux in AD progression

**Fig. 4:**
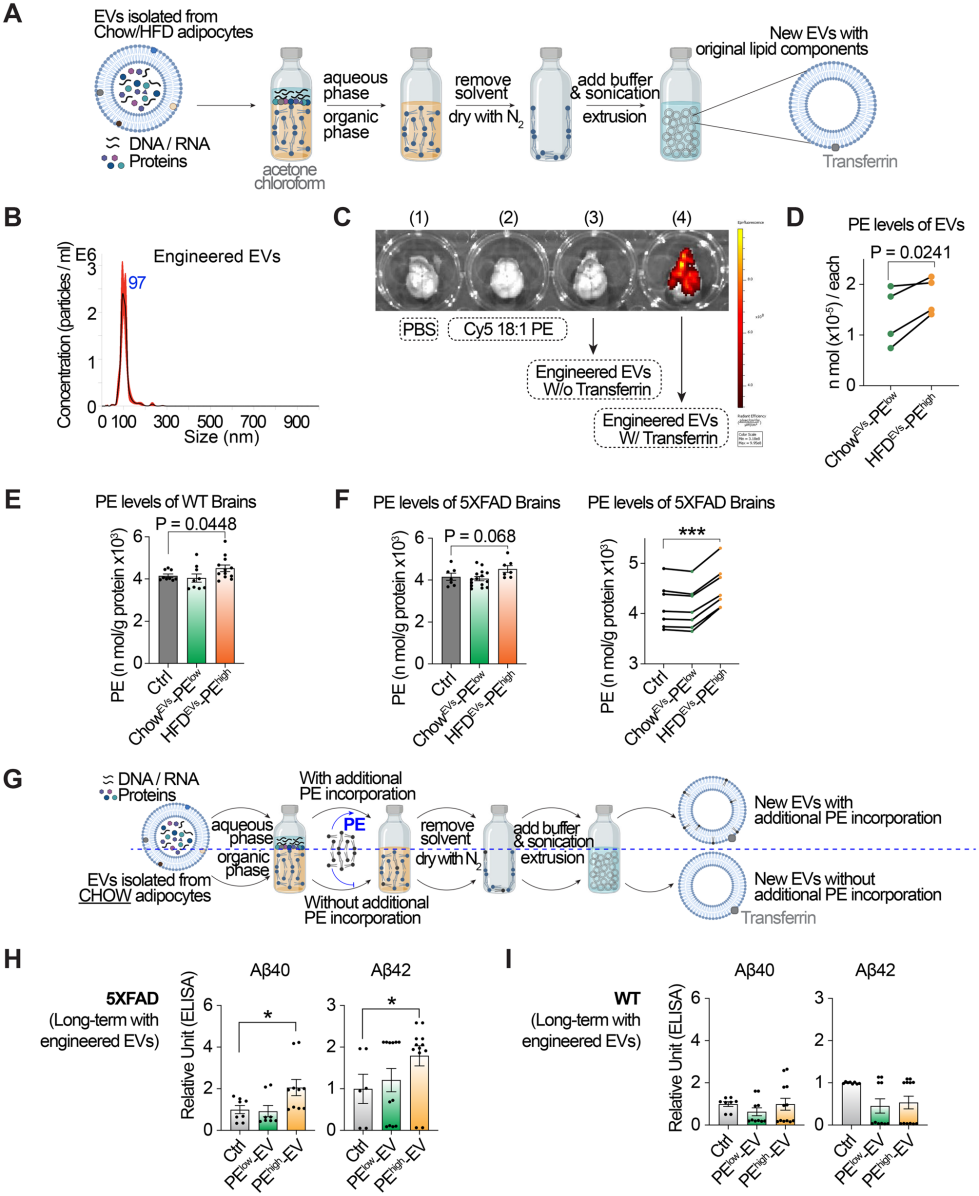
The PE^high^ flux exacerbates the pathologies of AD. **A**. schematic representation of the generation of engineered EVs, designed to be free of endogenous nucleic acids and proteins, and derived from EVs isolated from lean and obese adipocytes. **B**. Quantification and size analysis of engineered EVs derived from adipocytes, performed by nanoparticle tracking analysis using a NanoSight system with a 1:1000 dilution. **C**. Representative biodistribution of engineered EVs incorporating Cy5 PE after intraperitoneal (I.P.) injection in WT mice. The images show the following conditions: (1) brain with PBS administration, serving as the control; (2) brain with Cy5 18:1 PE administration; (3) brain with engineered EVs lacking Transferrin for blood-brain barrier (BBB) crossing; and (4) brain with engineered EVs modified with Transferrin to facilitate BBB crossing. **D**. ELISA assay of PE levels of the nature EVs from lean and obese adipocytes. **E**. ELISA assay of PE levels of WT mouse brains with/without the indicated EV treatment. **F**. ELISA assay of PE levels of 5XFAD mouse brains with/without the indicated EV treatment. **G**. Schematic representation of the generation of engineered EVs, derived from lean adipocyte–derived EVs and supplemented with additional PEs, designed to be free of endogenous nucleic acids and proteins. **H**-**I**. Comparison of the mean levels of the indicated AD pathological proteins in the brains of 5XFAD and age-matched control mice following long-term administration of vehicle, PE^low^-EVs, and PE^high^-EVs, measured by ELISA. (**D**), data are derived from four independent experiments, each treated as a biological replicate for statistical analysis. (**E**, **F**, **H** and **I**), each data point represents an individual mouse, and individual mice were treated as biological replicates for statistical analysis. Each group consisted of 6–14 mice, with approximately equal numbers of males and females. Data are presented as mean ± SEM; *, increase; #, decrease; */^#^*p* < 0.05, **/^##^*p* < 0.01, and ***/^###^*p* < 0.001. Statistical significance was determined using paired one-tailed t-tests for (D and F, right) and unpaired two-tailed t-tests for (**E**, **F** left, **H**, and **I**), with obese-EV and lean-EV groups each compared separately to the PBS control and no direct comparisons made between obese-EV and lean-EV groups

To isolate the role of lipid flux, we engineered lipid-only EVs by removing nucleic acids and proteins from adipose-derived EVs. Equal numbers of EVs derived from both lean and obese adipocytes underwent chloroform/acetone-based phase separation to eliminate DNA/RNA (aqueous phase) and proteins (pellet), yielding a phospholipid-rich organic phase (Fig. 4A). The lipid fraction was nitrogen-dried, reconstituted in PBS for blank EVs using thin-film hydration [43–45], and subsequently extruded to a uniform size of 100 nm (Fig. 4B).

To ensure brain delivery, transferrin, a well-established blood-brain barrier transport facilitator [43–45], was conjugated to the blank EVs. This modification enabled efficient brain localization of Cy5-labeled EVs (Fig. 4C). Engineered EVs derived from HFD adipocytes contained significantly higher PE levels than controls (Fig. 4D), indicating that EVs from obese adipocytes can serve as carriers of PE^high^ flux.

To confirm brain-targeted delivery of PE, we quantified brain PE levels following chronic administration (2 weeks) of engineered EVs. In WT mice, two weeks of treatment with HFD-derived EVs resulted in an increase in brain PE levels (Fig. 4E). In 5XFAD mice, unpaired two-tailed t-tests revealed a trend toward elevated PE levels following HFD-EV treatment (Fig. 4F, left), and age-stratified analysis using a paired one-tailed t-test confirmed the increase (Fig. 4F, right). These results indicate that EVs from obese adipocytes can disrupt brain PE homeostasis through sustained PE delivery, although the magnitude of PE modulation may be limited.

To determine whether the amyloidosis burden observed in acute EV treatments were specifically driven by PE, rather than other lipid components, DNA/RNA, or proteins, we generated chow diet-derived EVs with controlled PE content. Using the specific PE species identified in Fig. 2, we generated PE^high^-EVs and PE^low^-EVs by selectively supplementing these PE components during EV formulation (Fig. 4G), followed by chronic in vivo administration. In 5XFAD mice, PE^high^-EV treatment significantly increased Aβ40 and Aβ42 levels, while WT littermates exhibited sporadic elevation (Fig. 4H–I).

Because EVs are distributed across multiple peripheral organs (Fig. S3B), directly attributing brain-specific effects to EV uptake via tissue-by-tissue tracing was impractical. To address potential concerns that observed brain responses may arise secondarily through systemic or peripheral organ-mediated mechanisms, we evaluated whole-body metabolic status using glucose tolerance testing (GTT), which showed no significant changes following chronic EV administration (Fig. S4C). Furthermore, we used young mice, rather than aged animals or fully chronic diet-induced obesity models, to minimize systemic confounders such as insulin resistance, global lipid remodeling, hepatic or renal stress, or heightened splenic immune activity.

Taken together, these metabolic controls and direct brain PE measurements suggest that the observed cerebral effects are unlikely to result from indirect peripheral EV actions. These data demonstrate that PE^high^ flux directly promotes amyloidosis burden of AD in the brain. The pathological impact of elevated PE flux underscores its gain-of-function contribution to AD progression. We next examined whether restoring PE homeostasis could reverse its detrimental effects on the brain.

### Restoration of PE homeostasis alleviates cognitive dysfunction in an AD mouse model

To identify a compound with both disease-modifying potential in AD and the ability to regulate PE homeostasis, we employed a multi-step drug screening strategy. The anti-AD screen was conducted using our in-house SMART (Systematic Alzheimer’s Disease Drug Repositioning) pipeline (Fig. S5A–S5C), which integrates in silico drug screening, in vitro and in vivo validation, and behavioral testing (see Methods). Among the candidates, ebselen (2-phenyl-1,2-benzisoselenazol-3(2 H)-one) emerged as a top hit during the in silico phase (Fig. S5D) and was prioritized for further experimental validation.

For in vitro testing, we used a high-throughput 3D “AD-in-a-Dish” (3DDS) assay—a human neural stem cell-based model that enables quantitative assessment of p-tau fibril accumulation and neurite integrity (Fig. S6A–S6C) [32]. Both short-term (3-day) and long-term (3-week) ebselen treatment significantly reduced p-tau levels and improved neurite density in 3DDS cultures (Fig. S6D–S6I).

**Fig. 5:**
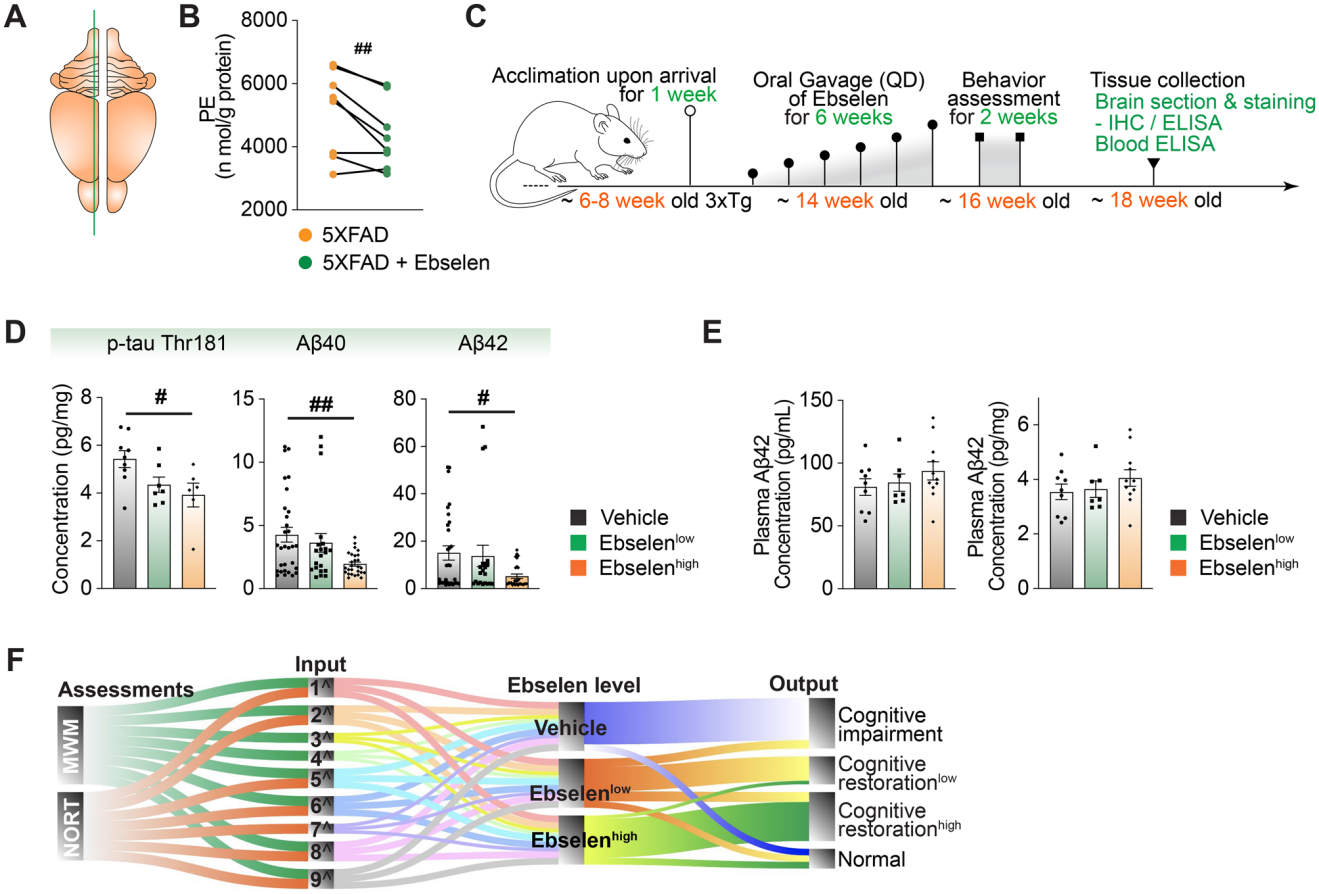
PE deregulation drug, ebselen, alleviates the AD related pathology and reduces neurobehavioral deficits in AD. **A**. Schematic representation of brain regions used for section collection. **B**. ELISA assay of brain PE levels in 5XFAD mice with or without ebselen treatment. *n* = 8 mice per group. Each sample was measured in multiple technical replicates (*n* = 13), which were averaged prior to analysis. **C**. Experimental scheme of 3xTg mice administrated with ebselen. **D**-**E**. ELISA assay of ⍺-p-tau Thr181, ⍺-Aβ40, and ⍺-Aβ42 of brain tissue and plasma from both male and female 3xTg mice upon vehicle, ebselen^low^ (60 mg/kg body weight), and ebselen^high^ (300 mg/kg body weight) administration. Group sizes were as follows: vehicle (male 4–5, female 5; total *n* = 9–10), ebselen^low^ (male 5, female 3; total *n* = 7), and ebselen^high^ (male 3–6, female 3–4; total *n* = 6–10). For the Aβ40 and Aβ42 measurements in panel D, each sample was measured in triplicate. Panel E shows plasma Aβ42 concentrations obtained from the same cohorts, with the right panel representing values normalized to the corresponding plasma protein levels. **F**. Alluvial diagram of semi-quantitative animal behavior assessments upon morris water maze (MWM) and novel object recognition test (NORT) evaluation. 1^^^, visit number of target area; 2^^^, visit number of non-target area; 3^^^, visit number of platform; 4^^^, visit number of opposite area in MWM; 5^^^, distance in target area; 6^^^, distance in non-target area; 7^^^, time in target area; 8^^^, time in non-target area; 9^^^, speed. (**B**, and **D**–**E**), each data point represents an individual mouse, and individual mice were treated as biological replicates for statistical analysis. Data are presented as mean ± SEM; *, increase; ^#^, decrease; */^#^*p* < 0.05, and **/^##^*p* < 0.01. Statistical significance was determined using a paired t-test based on littermate controls (**B**) and one-way ANOVA with Tukey’s post hoc test (**D**–**E**)

In the PE homeostasis screening stage, ebselen significantly reduced PE levels in the brains of 5XFAD mice (Fig. 5A–B). This reduction in PE levels was accompanied by a dose-dependent decrease in p-tau Thr181, Aβ40, and Aβ42 levels in the 3xTg AD mouse model following chronic ebselen treatment (6 weeks) (Fig. 5C–D). The 3xTg model, which exhibits both Aβ deposition and tauopathy, further validated the SMART pipeline predictions and in vitro findings, supporting the therapeutic potential of ebselen in mitigating core AD pathologies. Interestingly, plasma Aβ42 levels exhibited an inverse trend compared to brain levels (Fig. 5E).

Behavioral assessments, including the Morris water maze (MWM) and novel object recognition test (NORT), revealed significant cognitive improvements in ebselen-treated 3xTg mice (Fig. 5F& S7A-S7F), although these behavioral tests were less sensitive than biochemical assays (Fig. 5D). A comprehensive biosafety evaluation of ebselen revealed no significant differences between treated and vehicle groups in body weight (Fig. S7G-S7H), major organ integrity (Fig. S7I), or plasma pathology markers (Fig. S7J).

Further analysis of PE and PC metabolic gene expression in the brains of 5XFAD mice showed that baseline *Etnk2* expression, encoding the rate-limiting enzyme in the first step of de novo PE synthesis, was lower in the hippocampus than in the cortex (Fig. S8A). Following ebselen treatment, this regional difference became more pronounced (Fig. S8B). This effect is attributed to a significant reduction in *Etnk2* levels in the hippocampi following ebselen administration (Fig. S8C). Together, these findings suggest that ebselen, while eliciting antioxidant (*Nrf1*) and anti-inflammatory (*Il6*) responses (Fig. S8C), also plays a crucial role in regulating PE homeostasis.

To investigate the mechanism by which ebselen regulates PE metabolism, we performed lipidomic profiling of mouse brains treated with or without ebselen. This analysis showed that PE and triglyceride (TAG) species represented two of the lipid classes most affected by ebselen treatment, including PE (16:0_22:4) and PE (*P*-17:0_22:4) (Fig. S8D–E). In line with these species-level changes, overall PE abundance was reduced in ebselen-treated brains. Given that TAG synthesis is transcriptionally regulated by SREBP1 [57], and that NFκB signaling has been implicated in the regulation of SREBP1 activity [58], we hypothesized that ebselen modulates PE metabolism through an NFκB–SREBP1–ETNK axis. Consistent with this model, ebselen treatment markedly reduced *Srebf1* expression in both the hippocampus and cortex (Fig. S8F). In parallel, immunofluorescence analysis revealed decreased nuclear localization of NFκB in the brains of ebselen-treated mice (Fig. S8G), supporting a mechanism in which ebselen attenuates NFκB activity and thereby suppresses SREBP1-dependent lipid metabolic signaling.

To further validate the proposed NFκB–SREBP1–ETNK axis underlying ebselen-mediated regulation of PE metabolism, we employed an H₂O₂-induced oxidative stress model, motivated by prior evidence that ebselen modulates NFκB signaling via redox-dependent pathways [59]. Under Reactive Oxygen Species (ROS)-mimicking conditions, ebselen, rather than its structural analog ebsulfur, reduced SREBP1 and ETNK protein levels, and pharmacologic perturbation of NFκB (Bay 11–7082 inhibition; LPS activation) or SREBP1 (fatostatin inhibition; insulin activation) produced corresponding changes in SREBP1 and/or ETNK expression (Fig. S9A–F). Consistently, imaging analyses revealed concordant changes in cellular PE abundance (Fig. S9G–K). ChIP–qPCR further demonstrated NFκB occupancy at the *SREBF1* promoter and SREBP1 binding at the *ETNK* promoter (Fig. S9L–M). Notably, an in vitro ETNK enzymatic assay showed that ebselen more potently inhibited the first rate-limiting step of de novo PE biosynthesis than ebsulfur (Fig. S9N), supporting dual regulation of PE metabolism through both transcriptional control and direct enzymatic inhibition.

Collectively, these multi-functional properties enable ebselen to slow the pathological progression of AD and improve cognitive function in AD mouse models, without inducing adverse side effects. These findings raise the possibility that ebselen can modulate PE metabolism through NFκB-associated mechanisms, consistent with its known involvement in ROS-dependent signaling pathways [59]. However, whether excessive PE accumulation leads to distinct cell-type-specific effects relevant to AD pathogenesis remains unclear. To address this knowledge gap, we next focused on uncovering the underlying mechanisms by which sustained PE^high^ flux perturbs neuroimmune systems.

### Sustained PE overload induces T cell exhaustion

T cells and microglia represent the circulating and resident immune compartments of the brain, respectively, and are central to neuroinflammatory regulation, it remains to be determined whether alterations in PE^high^ context affect their intrinsic states or their crosstalk with neurons.

We used the DO-11–10 CD4^+^ T cell hybridoma to simulate infiltrating lymphocytes and evaluated the effect of ethanolamine (Etn) supplementation on cell viability and PE metabolism. Cell viability remained unaffected at ≤10 mM Etn but was compromised at higher concentrations (Fig. S10A). To selectively elevate intracellular PE levels, cells were treated with low (0.8 mM) or high (8 mM) Etn to promote *de novo* synthesis via the CDP-ethanolamine pathway, resulting in upregulation of ethanolamine pathway enzymes (*Etnk1* and *Pcyt2*) and a significant increase in intracellular PE levels (Fig. S10B–C).

In this model, PE levels increased by 1.47–1.86-fold in Etn^low^-PE and 5.91–6.9-fold in Etn^high^-PE conditions relative to control (calculation based on the data of Fig. S9C), closely matching the fold changes observed in obese versus non-obese individuals from our lipidomic analysis. These results validate the in vitro system as a physiologically relevant model of PE^high^ abundance in obesity.

**Fig. 6:**
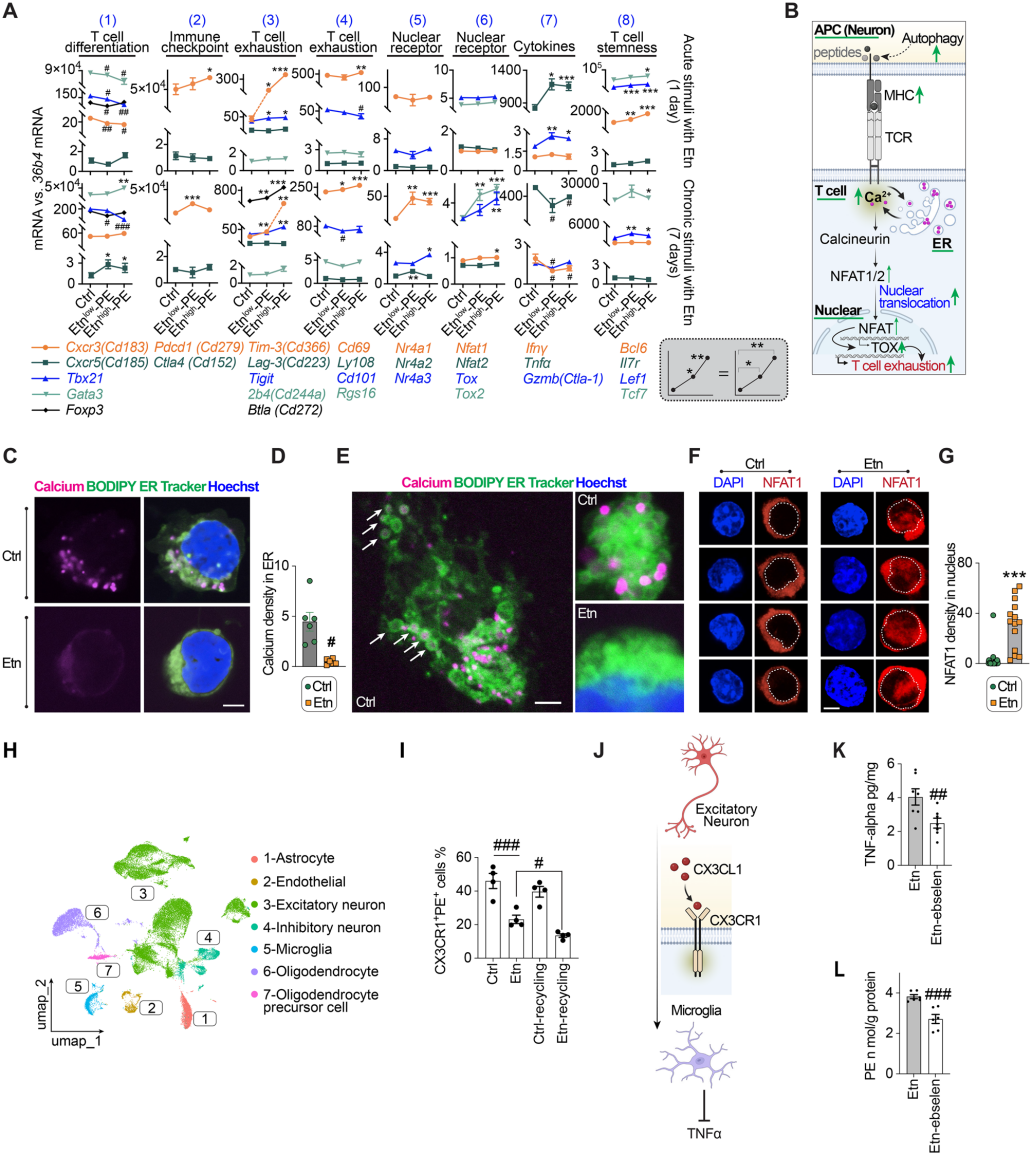
Sustained PE overload impairs T cell function and alters microglial receptor signaling. **A**. The summary of three to six independent RT-PCR studies on the expression of T cell function associated genes on the DO-11–10 T cell model upon Etn^low^-PE and Etn^high^-PE treatment for 1 or 7 days. The genes with the same class of annotation were cataloged into the same group to gain the comparative expression levels of each gene. **B**. Schematic diagram of two potential patterns by which PE^high^ flux mechanically promotes T cell dysfunction. Green labels indicate experimentally validated nodes. The T-cell receptor (TCR) can be activated by antigen presentation via MHC molecules on antigen-presenting cells. This downstream activation initiates a transient spike in cytosolic Ca^2+^ by depleting calcium stores in the endoplasmic reticulum (ER). The increase in Ca^2+^ concentration promotes the activation of calmodulin-dependent enzymes, including calcineurin (CaN). CaN subsequently dephosphorylates multiple serine residues on NFAT proteins, resulting in their translocation to the nucleus and activation of NFAT-dependent transcription, which includes genes essential for immune responses. The first mechanism involves PE acting through the Autophagy-MHC-TCR-Ca^2+^-NFAT signaling pathway. Alternatively, PE may initiate downstream signaling by destabilizing the ER membrane, thereby releasing stored Ca^2+^. **C**-**D**. Representative IF staining and quantification of human peripheral blood CD8^+^ T cells (ex vivo) with or without Etn treatment. Oregon Green™ 488 BAPTA-1, AM was used to detect intracellular calcium signals; BODIPY ER tracker for endoplasmic reticulum (ER); and Hoechst for nuclear staining. Scale bar, 5 µm. **E**. Representative IF staining of the detailed view of the ER structure in human peripheral blood CD8+ T cells with or without Etn treatment, using the calcium probe, BODIPY ER tracker, and Hoechst. Scale bar, 5 µm. In the left and top-right panels, ER structures in the control group display predominantly circular or reticular formations. In contrast, in the bottom-right panel, the ER in the PE^high^ group exhibits a loss of its characteristic organization, appearing more diffuse and disorganized. **F** & **G**. Representative IF staining and quantification of human peripheral blood CD8^+^ T cells with or without Etn treatment, using α-NFAT1 antibody and DAPI for nuclear staining. Scale bar, 5 µm. White dashed lines in the NFAT1 images indicate nuclear contours. **H**. uniform manifold approximation and projection (UMAP) plot of 37,559 cells across 7 combined clusters from female 5XFAD brains treated with engineered EVs. Each group (PE^low^-EVs and PE^high^-EVs) consists of 2 samples, with each sample pooled from 2 mice. **I**. CX3CR1 recycling assay in primary microglia with or without Etn treatment. **J**. Schematic of CX3CL1–CX3CR1-mediated signaling between excitatory neurons and microglia, leading to the suppression of TNFα in microglia. **K**-**L**. quantification of TNF-α secretion in the culture medium and intracellular PE levels in primary microglia with Etn and/or ebselen treatment. (**D** and **G**), data are derived from three independent experiments, with a total of 6–14 images (each dot represents one image) analyzed per condition; independent experiments were treated as biological replicates for statistical analysis. (**I**, **K**, and **L**), each data point represents an independent experiment (*n* = 4–6). Data are presented as mean ± SEM; *, increase; ^#^, decrease; */^#^*p* < 0.05, **/^##^*p* < 0.01, and ***/^###^*p* < 0.001. (**A**), statistical significance was determined using two-tailed Student’s t-tests, with comparisons made only to the corresponding controls at the same time point and no direct comparisons made between PE-high and PE-low groups. (**I**, **K**, and **L**); one-way ANOVA followed by Tukey’s multiple comparison test was used

Short- (1 day) and long-term (7 days) exposure to elevated PE levels induced significant upregulation of immune checkpoint receptor (ICR) genes (*Pdcd1*, *Ctla4*, *Tim-3*, *Lag-3*, *Tigit*, *2B4*, *Btla*, *Cd69*), with *Tim-3* (*Cd366*) exhibiting the most pronounced response (Figs. 6A, 2^nd^ & 3^rd^ columns). Remarkably, the nuclear receptors associated with T cell exhaustion [60] (*Nr4a1*, *Nr4a2*, *Nr4a3, Nfat1*, *Nfat2*, *Tox*, and *Tox2*) remained relatively quiescent during acute (short-term) exposure to either Etn^low^-PE or Etn^high^-PE (Fig. 6A, upper 5^th^ & 6^th^ columns). However, nearly all of these factors were markedly up-regulated after prolonged (7-day) PE^high^ treatment (Fig. 6A, bottom 5^th^ & 6^th^ columns). This suggests that acute PE elevation initiates early T cell dysfunction, while chronic PE exposure drives their transition to a fully exhausted T cell phenotype.

**Fig. 7:**
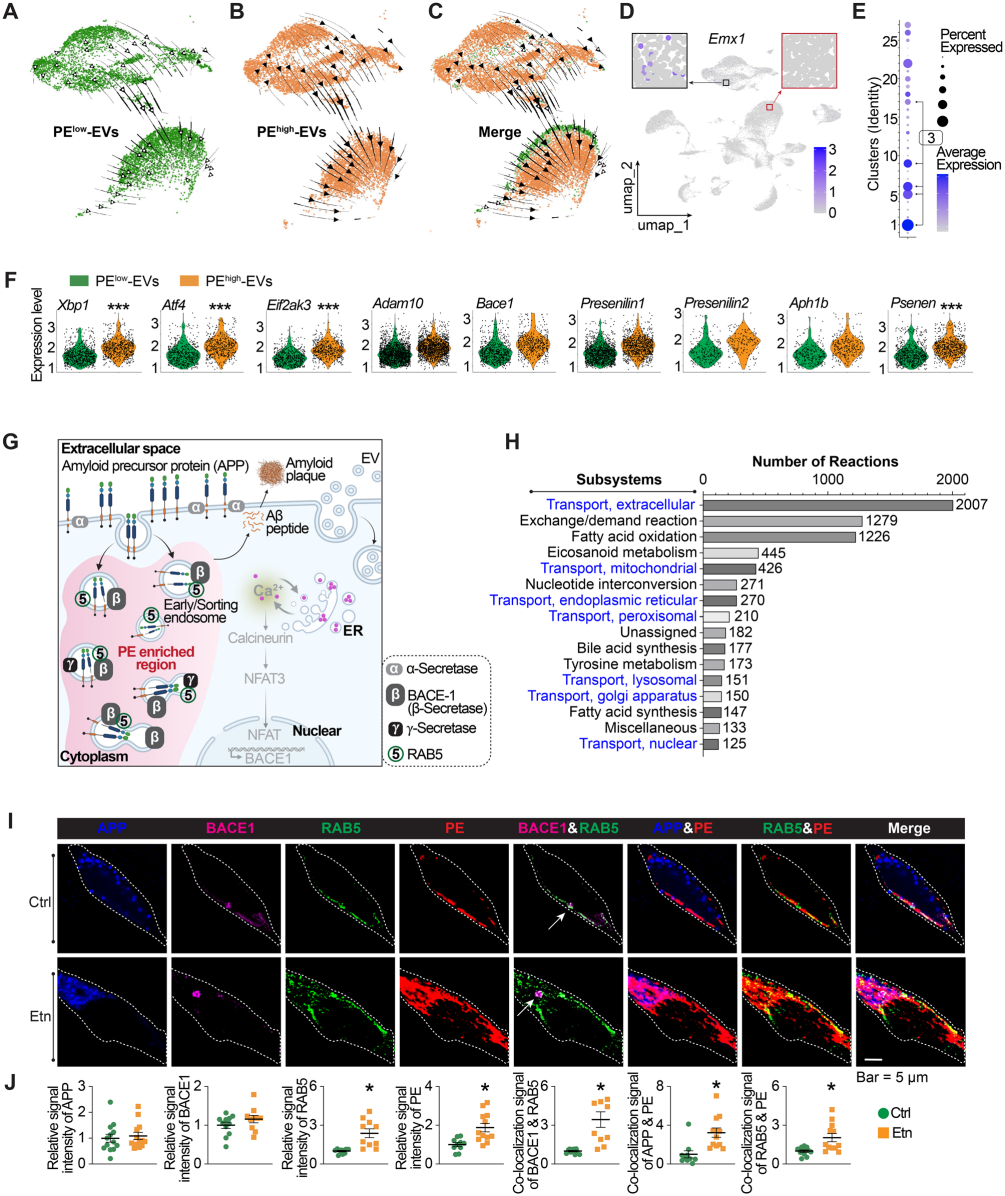
Sustained PE overload remodels membrane dynamics to shift APP processing toward amyloidogenic Aβ production. **A**-**C**. Velocity streams of excitatory neurons clusters with PE^low^-EVs or PE^high^-EVs administration. **D**. Feature plot of empty spiracles Homeobox 1 (Emx1) within the UMAP (Fig. 6H), with zoomed-in regions of the indicated top and bottom clusters. *Emx1* is a marker of neural progenitor cells. **E**. Dot plot of the expression levels of *Emx1*. *Emx1* is highly expressed in clusters 1, 5, 6, 9, and 17 (fig. S10B), which are part of the combined cluster 3 (Fig. 6H). **F**. Violin plots showing the expression levels of genes associated with ER stress (*Xbp1*, *Atf4*, *Eif2ak3*), α-secretase (*Adam10*), β-secretase (*Bace1*), and γ-secretase components (*presenilin 1/2*, *Aph1b*, *Psenen*). **G**. Schematic diagram of two potential mechanisms by which PE^high^ flux promotes Aβ generation in excitatory neurons. Non-amyloidogenic processing primarily occurs at the cell surface, where α-secretase is highly concentrated. In contrast, amyloidogenic processing mainly takes place in endocytic organelles, which maintain an acidic microenvironment conducive to BACE1 activity, facilitating APP’s encounter with β-site cleavage. **H**. Bar chart showing the ranking of top subsystems based on the corresponding number of reactions predicted by COMPASS in the excitatory neuron cluster. **I** & **J**. Representative IF staining and the statistics with ⍺-APP, ⍺-BACE1, ⍺-RAB5 antibodies and ⍺-PE probe in HEK293–APP695-WT cells with or without Etn treatment. Scale bar, 5 µm. The white dashed line marks the contour just external to the cell membrane, rather than the plasma membrane itself. (**J**), data are derived from three independent experiments, with ~10–12 images analyzed per condition (each dot represents one image); independent experiments were treated as biological replicates for statistical analysis. Data are presented as mean ± SEM; *, increase; ^#^, decrease; */^#^*p* < 0.05, **/^##^*p* < 0.01, and ***/^###^*p* < 0.001. Statistical significance was determined using the wilcoxon rank-sum test (**F**) and unpaired two-tailed Student’s t-tests (**J**)

The ability to produce high level of cytokines, such as interferon-γ (IFN-γ), is a key indicator of effector functionality [61] and resistance to exhaustion [62–65]. Under acute PE^high^ flux conditions, T cells retained their capacity to produce key cytokines (*Ifnγ*, *Tnfα*, and *Gzmb*), reflecting preserved effector functionality, whereas these cytokines were significantly diminished under chronic PE^high^ conditions, consistent with progressive T cell dysfunction and exhaustion over time (Figs. 6A, 7^th^ column & S10D). In parallel, canonical T cell stemness–associated markers (*Tcf7*, *Lef1*, *Il7r*, and *Bcl6*) showed a marked increase under acute PE^high^ conditions, whereas under chronic PE^high^ flux their induction was present but less pronounced, particularly at higher PE level, indicating a blunted stem-like response over time (Fig. 6A, 8^th^ column). The in vivo data, including analyses of peripheral blood and brain parenchyma, supported the presence of T cell dysfunction (Fig. S10E-F). Aged mice were therefore selectively employed to reduce the likelihood of false-negative findings and to ensure sufficient statistical power for detecting T cell–associated phenotypes.

These findings suggest that acute PE^high^ conditions initiate T cell dysfunction while preserving stemness and effector functionality, whereas chronic PE^high^ conditions, as seen in obesity, impairs T cell surveillance and effector functionality, ultimately leading to profound exhaustion. As a dynamic immune population in the brain, T cells are susceptible to peripheral influences, and their partial exhaustion may reflect systemic immune alterations. To define the specific relevance of T cell dysfunction within the neuroimmune microenvironment of AD, we next investigated neuron–T cell crosstalk through major histocompatibility complex (MHC)-T cell receptor (TCR) interactions [66], especially under PE^high^ condition where neuronal signaling may be altered.

### Sustained PE overload mechanically disrupts neuroimmune crosstalk

The MHC-TCR crosstalk between neuron and T cell within PE^high^ context represents a distinct yet underexplored mechanism of neuroimmune regulation in AD. This ligand–receptor engagement initiates the TCR signaling cascade, ultimately upregulating genes associated with T cell dysfunction. Mechanistically, this process involves calcium mobilization from the neuronal endoplasmic reticulum (ER), calcineurin activation, and subsequent NFAT1/2 nuclear translocation, which activates the TOX transcriptional program characteristic of T cell exhaustion [60, 67] (Fig. 6B), as supported by the coordinated induction of multiple TOX-regulated exhaustion-associated genes shown in Fig. 6A.

To investigate whether PE^high^ level primes neuronal MHC signaling to facilitate MHC–TCR-mediated communication, we considered the broader regulatory roles of PE. Beyond its membrane role, PE regulates autophagy (Fig. 2C, bottom), which is crucial for antigen presentation (AP) and T cell resilience [68]. In primary neurons (C57bl6-WT), PE enhances autophagy (Fig. S10G), promoting MHC-mediated antigen presentation (Fig. S10H).

To examine whether enhanced neuronal MHC activity under PE^high^ conditions intensifies signaling in T cells, we co-cultured DO-11–10 T cells with Etn pre-treated primary neurons, using neuron-conditioned medium without Etn. The co-cultured T cells exhibited TCR downstream activation and observed induced genes linked to T cell dysfunction (Fig. S10I). The evidence (Fig. S10G-I) suggest that enhanced autophagy flux correlated with increased MHC-related gene expression in neurons, potentially augmenting antigen presentation and, in turn, prompting crosstalk with T cells.

To clarify how PE^high^ conditions drives T cell dysfunction–associated gene expression in the absence of direct MHC-TCR engagement (Fig. 6A), we hypothesized that aberrant downstream TCR signaling may result from dysregulated intracellular calcium handling induced by PE overload. Using human CD8^+^ T cells for ex vivo validation, we observed that under PE^high^ conditions, calcium was inadequately sequestered within the ER (Fig. 6C–D), and PE caused profound morphological changes to the ER—disrupting its typical sheet- or ring-like structure and producing a diffuse, irregular architecture (Fig. 6E).

This structural remodeling enhanced calcium mobilization from ER anchoring sites into the cytosol, triggering TCR downstream signaling and robust NFAT nuclear translocation, as confirmed by subcellular immunofluorescence imaging (Fig. 6F–G). Importantly, similar effects were not observed with PC^high^ treatment (Fig. S10J-K), indicating that these responses are specific to PE and not a general property of glycerophospholipid accumulation.

These findings suggest PE facilitates T cell exhaustion via two complementary mechanisms (Fig. 6B). The first cascade is mediated by autophagy-driven antigen presentation, initiating the TCR associated cascade and subsequent upregulating T cell exhaustion pathways. The second is mediated by ER destabilization and calcium dysregulation under PE^high^ condition, leading to incomplete TCR associated pathway and driving T cell dysfunction. Collectively, these dual mechanisms underscore how PE^high^ flux reprograms both neuron and T cells, facilitating immune milieu changes.

### PE^high^ flux reprograms microglial identity and disrupts neuron–microglia communication

Given the migratory nature of T cells and their susceptibility to peripheral metabolic cues, we shifted our focus to resident immune cells—microglia—to dissect local neuroimmune interactions. Specifically, we examined how elevated PE flux modulates neuron–microglia crosstalk within the brain microenvironment.

To investigate the cell-type-specific effects, we performed single-nucleus RNA sequencing (snRNA-seq) in the 5XFAD model treated with the engineered PE^high^- or PE^low^-EVs (Fig. 6H& S11A–S11B). Major brain cell types were robustly clustered and validated using canonical markers (Fig. S11C; Table S4). While WT mice were included in earlier phenotypic analyses, they exhibited only sporadic and modest Aβ elevation following PE^high^-EV administration (Fig. 4I). To better capture molecular signatures linked to robust pathological phenotypes, snRNA-seq was thus focused on the 5XFAD model.

To assess the metabolic consequences of PE^high^-EV treatment in microglia, we applied COMPASS [50], a transcriptome-based flux balance analysis tool. Dysfunctional microglia and elevated *ApoE* expression were strongly associated with increased activity across energy and biosynthetic pathways, including fructose and mannose metabolism, the pentose phosphate pathway, triacylglycerol synthesis, glycolysis, the tricarboxylic acid (TCA) cycle, and PE metabolism (Fig. S11D, arrows).

Consistent with these metabolic changes, snRNA-seq revealed transcriptional features of microglial dysfunction. Among all the dysfunction associated genes in our analysis (Fig. S11D), several key homeostatic surface markers—including *Trem2*, *P2ry12*, and *Cx3cr1*—were significantly downregulated, with *Cx3cr1* showing the most pronounced reduction (Fig. S11E), indicating impaired microglial identity and surveillance. This transcriptomic pattern, together with our earlier observation of lipid droplet accumulation in PE^high^-treated microglia (Fig. 3), suggests a broader disruption of lipid homeostasis that may extend to membrane receptor regulation.

To determine whether membrane instability under PE^high^ condition disrupts receptor dynamics, we conducted a CX3CR1 recycling assay (Fig. 6I). Primary 5XFAD microglia exhibited reduced plasma membrane localization of CX3CR1, especially under excess PE (Fig. 6I), suggesting impaired receptor trafficking. This effect was absent under PC^high^ condition (Fig. S11F).

CX3CL1–CX3CR1 signaling governs neuron–microglia communication and regulates the inflammatory output such as TNF-α and IL-6 [69] (Fig. 6J). We next evaluated whether PE-driven CX3CR1 abrogation disrupts this axis. Microglia cultured in neuron-conditioned medium (B27-free, rich in CX3CL1) displayed increased TNF-α production under PE^high^ condition (Fig. 6K). Ebselen lowered intracellular PE (Fig. 6L), restored CX3CR1 localization, and suppressed TNF-α levels (Fig. 6K), indicating that PE dysregulation promotes microglial inflammation by impairing CX3CL1–CX3CR1 signaling (Fig. 6I–L).

In summary, PE^high^ flux induces coordinated disruption of membrane-associated proteins and metabolic rewiring in microglia. Deregulation of key signaling receptors—including *Trem2*, *P2ry12*, *Cx3cr1*, and *ApoE*—reflects compromised membrane stability and immune surveillance. Notably, *Cx3cr1* emerges as a particularly sensitive target within PE overloaded condition, linking membrane dysfunction to impaired neuron–microglia crosstalk and proinflammatory activation in AD.

Microglial dysfunction compromise Aβ clearance, contributing to increased amyloid burden [70]. However, it remains unclear whether the Aβ accumulation observed under PE^high^ conditions (Fig. 4H) results solely from impaired degradation, or whether Aβ biogenesis is also actively promoted at the neuronal level.

### PE^high^ flux mechanically alters the amyloidogenic pathway to facilitate Aβ generation in excitatory neurons

Global trajectory analysis of snRNA-seq data revealed a unique shift in excitatory neuron fate under PE^high^ condition, characterized by a transition from an *Emx1*-enriched progenitor cluster through the PE^low^-EV cluster to the PE^high^-EV cluster (Fig. 7A–C), with progressive *Emx1* loss (Fig. 7D–E). *Emx1* marks neural progenitors, this suggests that PE exposure alters neuronal developing identity between PE^low^-EV and PE^high^-EV treatment. In the PE^high^ group, upregulation of *S100b* and *Ldhb* further indicated neuronal stress and damage (Fig. S11G). These findings suggest that Aβ accumulation may result not only from impaired microglial function (Fig. 6) but also from neuron-intrinsic changes in Aβ biogenesis.

Excitatory neurons are known to signal through an NFAT pathway, where ER calcium release induces NFAT3 nuclear translocation and subsequent BACE1 upregulation [71, 72]. In our data, PE^high^ neurons showed elevated ER stress-related genes (*Xbp1*, *Atf4*, *Eif2ak3*; Fig. 7F) and calcium mobilization channels (Fig. S12), suggesting NFAT pathway engagement. However, *Bace1* expression was not significantly changed (Fig. 7F), indicating that this is not the primary driver of Aβ production in PE^high^ context (Fig. 7G). Instead, the marked upregulation of *Psenen* (Fig. 7F)—a γ-secretase-stabilizing chaperone—points to an alternative mechanism by which PE^high^ flux enhances APP cleavage and promotes Aβ accumulation.

To further explore how PE^high^ flux affects APP cleavage, we analyzed COMPASS-derived metabolic pathways in excitatory neurons. Among 99 subsystems, transport-related pathways were markedly upregulated (Fig. 7H; Table S5), indicating enhanced endomembrane remodeling, including PE-associated reactions (Fig. S12, blue arrows).

Given that early/sorting endosomes provide an acidic environment optimal for BACE1-mediated β-cleavage of APP [73], we hypothesized that PE alters the spatial distribution of APP, BACE1, and endosomal compartments. Using APP-overexpressing HEK293 cells to model APP-overload conditions (Fig. 7I–J), similar to those in 5XFAD mice, we validated that PE surplus did not affect overall APP expression but shifted its localization from the plasma membrane—where α-cleavage is favored—to the cytoplasm, where β-cleavage is more likely (Fig. 7I–J).

Consistent with snRNA-seq findings (Fig. 7F), BACE1 expression remained unchanged but exhibited intracellular distribution mirrored that of APP. RAB5, a marker of early/sorting endosomes, showed strong intracellular co-localization with PE (Fig. 7I–J), suggesting increased endosomal trafficking to the cytoplasm. These spatial visualizations support the hypothesis that PE-induced membrane fluidity promotes endosome formation and facilitates APP internalization, enhancing proximity to BACE1. Notably, these effects were not observed with PC^high^ treatment (Fig. S13A). Consistently, brain sections from female mice treated with PE^high^-EVs recapitulated the in vitro findings, showing increased spatial proximity among APP, BACE1, and endosomal markers in AD-relevant contexts (Fig. S13B–H). In contrast, PE^low^-EV–treated controls exhibited minimal overlap of APP and BACE1 with Rab5-positive endosomes (Fig. S13B, C, arrows).

Altogether, our results indicate that PE^high^ condition drives amyloidogenic APP processing by reorganizing endomembrane structure and altering the spatial positioning of APP-cleaving enzymes to facilitate Aβ generation (Fig. 7G).

Finally, we examined BBB integrity using both snRNA-seq and orthogonal functional assays. Analysis of our snRNA-seq dataset from engineered EV–treated mouse brains identified a robust endothelial cell cluster (Fig. S14A-B). Differential expression analysis of this cluster revealed no significant changes in canonical endothelial markers or in gene programs associated with vessel integrity or density, apoptosis, lipid metabolism, or inflammatory signaling [74] (Fig. S14C). Within the resolution of our transcriptomic analysis, these data do not indicate overt endothelial perturbation following engineered EV treatment. Immunostaining for CD31 (Fig. S14E), a marker of endothelial cells and vessel density [74], showed comparable signal intensity across treatment conditions and mouse models. Together, these data provide no evidence of gross endothelial disruption under the experimental conditions tested, supporting interpretation of downstream cellular phenotypes in the absence of detectable vascular structural alterations.

## Discussion

This study builds on comprehensive profiling of the human adipose lipidome and systematically investigates the impact of elevated PE levels on multiple aspects of AD pathology (Fig. S15). Our findings suggest that PE^high^ levels not only reflects metabolic alterations but also contributes to dynamic membrane remodeling in a cell-type-specific manner. Compared with current models of metabolic dysfunction in AD, these results provide additional insight into how lipid homeostasis may influence disease progression.

Unlike previous studies that analyzed adipose lipidomes across broad age ranges [75], our study primarily focused on obesity during midlife, a critical window for dementia risk. Although many studies have examined lipid alterations in obese versus lean individuals, most have concentrated on major lipid species such as triglycerides, cholesterol, and fatty acids [48, 75, 76]. In comparison, PE, a membrane lipid with emerging biological significance, has received relatively little attention, especially in AD models. Through lipidomics-based systems profiling, we identified PE as one of the most discriminative lipid classes in midlife obesity (Fig. 1). This observation prompted further investigation into the functional relevance of PE in AD. Notably, the predominant PE species identified in Fig. 2 are PE plasmalogens. The dysregulation of these lipids reflects altered peroxisomal activity and reactive oxygen species metabolism, suggesting broader disruptions in cellular energy balance and redox homeostasis that are implicated in diabetes and neurodegenerative disorders [77, 78].

Clinical evidence highlights a dynamic association between PE levels and AD progression. Early reports on human brain PE levels indicate that decreased PE levels are positively correlated with AD pathology in both the hippocampus and cortex in end-of-life samples [54, 79]. A comprehensive analysis of previous quantitative detections of PE levels, considering spatiotemporal alterations and species levels (human and AD mouse models), reveals that PE levels exhibit a hierarchical change rather than a unidirectional progression throughout AD progression. Specifically, PE levels significantly increase during the mild, early, and middle stages of AD, followed by a decrease in the late stage [53, 80]. Consistently, analysis of the larger ROSMAP human brain lipidomics cohort shows that [52], despite substantial clinical and biological heterogeneity across samples (e.g., sex, education, race, APOE genotype, age at death, cognitive scores, PMI, Braak and CERAD scores), batch-resolved PE alterations are still observed, with Batch 1 and Batch 2 displaying a similar trajectory in which PE levels rise during early AD pathology before declining at later stages (Fig. 2F). These human data highlight the clinical relevance of our findings and support the pathological significance of altered PE metabolism in AD.

Our findings reveal that EVs derived from obese adipocytes actively promote lipid droplet biogenesis or accumulation in the brain (Fig. 3), implicating EVs as key modulators of lipid homeostasis in AD. Ectopic LD accumulation, particularly in microglia, is a hallmark of lipid dysregulation associated with neuroinflammation and neurodegeneration [27]. Although impaired intercellular lipid trafficking of fatty acids and cholesterol has been linked to amyloidogenesis and tauopathy [54], our data suggest that obese EVs function as lipid carriers that aggravate this burden. These results expand the functional role of adipose-derived EVs from systemic lipid signaling to direct contributors to central nervous system lipid imbalance in obesity-associated AD.

To rule out the potential influence of non-lipid components such as DNA, RNA, and proteins within adipose-derived EVs, we performed biochemical extractions to generate engineered EVs containing lipids only (Fig. 4). Moreover, this approach offers a broadly applicable lipid intervention strategy for probing the functional roles of other lipid species in the brain. While previous studies have primarily focused on miRNA cargo in EVs [39], our work highlights the pathogenic potential of lipids, specifically PE, in AD. To ensure experimental rigor, we included multiple control conditions, including blank EVs and lean adipose-derived EVs. In addition, WT mice were included as a control for 5XFAD to evaluate the pathological effects of PE-defined EVs in both genetic and non-genetic contexts. Notably, WT mice receiving PE^high^-EVs exhibited pathological changes resembling a sporadic AD-like state, whereas 5XFAD mice showed more severe responses (Fig. 4H–I).

Building on the observation that elevated PE flux induces gain-of-function phenotypes with detrimental effects in AD, we investigated whether restoring PE homeostasis could alleviate these outcomes. Using a systematic drug discovery platform developed in our lab (U.S. Patent Application 2019/0010533), we combined in silico screening, in vitro and in vivo validation, and behavioral testing to examine the relationship between PE dysregulation and cognitive impairment. Ebselen, identified for its ability to modulate PE levels, was used as a proof-of-concept compound to explore PE-related pathways (Fig. 5 & S8-9). Our mechanistic studies suggest that ebselen modulates PE metabolism through NFκB-associated pathways, integrating redox-sensitive transcriptional regulation with direct enzymatic effects on PE biosynthesis. Our results also suggest that ebselen exerts broader biological effects beyond its known antioxidant and anti-inflammatory actions in AD [81–83].

Furthermore, our study uncovers a previously unrecognized role of elevated PE flux in reshaping neuroimmune crosstalk in AD. Chronic PE elevation alters both microglial and T cell function through cell-type-specific membrane remodeling, contributing to a proinflammatory brain environment (Fig. 6). In T cells, elevated PE levels induced early immune checkpoint receptor expression and, with sustained exposure, activated exhaustion-related transcriptional programs, including *Tox*, *Nr4a*, and *Nfat1/2*, alongside reduced IFN-γ and TNF-α production, indicating loss of effector functionality (Fig. 6). While pathways such as PE-driven autophagy, T cell exhaustion, and the TCR–calcium–NFAT–TOX axis have been established [48, 60, 61, 67, 68, 84], our study confirms their activity under PE^high^ conditions and further reveals that PE overload causes ER deformation and calcium dysregulation, triggering aberrant TCR downstream signaling and functional exhaustion under metabolic stress (Fig. 6).

In parallel, microglia responded to excess PE with prominent lipid droplet accumulation and downregulation of key surface proteins, notably CX3CR1, which is essential for neuron–microglia communication. These alterations coincided with elevated TNF-α production and disrupted microglial homeostasis. Although the CX3CL1–CX3CR1 axis has been well-characterized in microglial regulation [69], our findings show that elevated PE levels interferes with this signaling pathway by altering receptor distribution (Fig. 6), establishing a mechanistic link between lipid imbalance and neuroimmune dysfunction in the AD brain.

Moreover, we elucidated the mechanisms by which PE contributes to membrane remodeling in excitatory neurons (Fig. 7). In the AD model, accelerated Aβ production appears to be driven by two potential mechanisms under PE elevation. First, PE enhances membrane fluidity, promoting the formation of early/sorting endosomes and facilitating the intracellular trafficking of mature APP and β-secretase. This spatial redistribution increases β-cleavage events and promotes Aβ generation. Unlike most cell surface receptors, mature APP does not remain on the plasma membrane for extended periods [85], making its intracellular routing critical for amyloidogenic processing. Second, PE-induced endoplasmic reticulum stress and abnormal calcium mobilization contribute to modest upregulation of BACE1. These findings are consistent with previous studies showing that genetic reduction of PE biosynthesis suppresses toxic Aβ production [20, 26], primarily by enhancing α-cleavage, reducing β-cleavage, and destabilizing γ-secretase. Our study shows that elevated PE levels increase β-cleavage frequency and enhances γ-secretase stability, revealing a complementary gain-of-function mechanism that aligns with the directional logic of PE-dependent APP processing.

Together, these findings outline a cell-type-specific role of elevated PE levels in modulating neuroimmune interactions in AD. T cells, microglia, and excitatory neurons all exhibited membrane remodeling under PE overload, suggesting a shared structural response that disrupts immune signaling and neuronal function. While each cell type showed distinct phenotypes, such as immune checkpoint activation and altered endosomal trafficking, these changes reflect a broader, multifaceted influence of PE on brain cells. This is consistent with previous observations that PE elevation, induced by extracellular potassium, enhances autophagic flux and mitochondrial AcCoA synthetase 1 while reducing histone acetylation [61] (Fig. 3H). However, unlike that study which emphasized epigenetic remodeling, our work highlights PE-triggered signaling cascades and membrane remodeling as key drivers of neuroimmune dysfunction. Importantly, the experimental design was structured to minimize potential batch effects, as datasets were either derived from single, continuous animal cohorts or analyzed separately across independent cohorts.

Finally, our work reveals that redundant PE levels act independently of other lipid components and exacerbates the progression of AD. While these findings provide important mechanistic insights, several aspects warrant further investigation. First, the human lipidomic dataset used in this study, while informative and obesity-driven, is modest in size; future validation in larger and more diverse cohorts (class I and II obesity) will help to further establish the clinical relevance of PE dysregulation. Second, although our results suggest a conserved role for PE in AD pathogenesis, additional investigation is needed to determine how broadly these mechanisms apply across AD subtypes, including both sporadic and genetically driven forms. Sex as a biological variable also merits further investigation. In addition, genetic or molecular perturbation approaches, including transgenic models targeting PE-regulating enzymes, will be essential to more directly define the causal involvement of PE levels in AD pathogenesis. Furthermore, future studies should examine, potentially using targeted lipidomics approaches to capture lipid species not fully resolved by untargeted profiling (e.g., complex sphingolipids and oxidized lipids), whether other lipid fluxes, such as that of PC or sphingolipids, operate through overlapping or distinct molecular pathways in the context of obesity-related neurodegeneration.

## Conclusion

This study identifies elevated PE levels as a key metabolic signature of midlife obesity that mechanistically links peripheral lipid dysregulation to AD pathogenesis. By integrating human lipidomics with multi-omics profiling and functional validation in AD models, we demonstrate that excessive PE accumulation drives cell-type-specific membrane remodeling in T cells, microglia, and excitatory neurons. These alterations impair neuroimmune communication, promote ectopic lipid storage, and enhance amyloidogenic processing, collectively exacerbating AD progression.

Our findings highlight a previously underappreciated role of membrane lipid homeostasis in coordinating immune and neuronal responses under metabolic stress. Moreover, they position PE flux as a modifiable driver of neurodegeneration, offering a unifying framework that connects systemic metabolic dysfunction with central nervous system pathology. Therapeutically, our data suggest that targeting PE levels through pharmacological agents such as ebselen may restore neuroimmune balance and mitigate cognitive decline.

Overall, this work advances our understanding of how obesity contributes to AD through lipid-driven mechanisms and lays the foundation for lipid-based interventions in metabolically linked neurodegenerative diseases.

## Electronic supplementary material

Below is the link to the electronic supplementary material.

Supplementary Material 1

Supplementary Material 2

Supplementary Material 3

Supplementary Material 4

## Data Availability

The snRNA-seq dataset has been deposited in the GEO database under accession number GSE280474. The raw lipidomics data used in this study have been previously published [86].

## Declarations

### Statement on the use of AI-assisted tools

In the course of preparing this manuscript, the authors made use of AI-assisted language tools to support the refinement and editing of the text. All content was subsequently carefully reviewed, revised, and verified by the authors. The authors assume full responsibility for the accuracy, integrity, and originality of the final published work.

## Abbreviations

AD: Alzheimer’s disease
PE: Phosphatidylethanolamine
TAG: Triacylglycerol
GPL: Glycerophospholipid
EV: Extracellular vesicle
CNS: Central nervous system
snRNA-seq: Single-nucleus RNA sequencing
FC: Fold change
BBB: Blood–brain barrier
GO: Gene Ontology
KEGG: Kyoto Encyclopedia of Genes and Genomes
PCA: Principal component analysis
UMAP: Uniform manifold approximation and projection
TCR: T cell receptor
IHC: Immunohistochemistry
FDR: False discovery rate
WT: Wild type
PBS: Phosphate-buffered saline
LC-MS: Liquid chromatography–mass spectrometry
DAPI: 4′,6-diamidino-2-phenylindole
DMSO: Dimethyl sulfoxide
TNF-α: Tumor necrosis factor alpha
CX3CR1: C-X3-C motif chemokine receptor 1
NFAT1: Nuclear factor of activated T cells 1
