# Supplementary material for: Obesity-driven phosphatidylethanolamine dysregulation impairs neuroimmune crosstalk and accelerates Alzheimer’s pathogenesis
Source: Mol Neurodegener. 2026 Apr 15;21:25. doi: 10.1186/s13024-026-00943-3 (PMC13159212; doi:10.1186/s13024-026-00943-3)
Supplement: Supplementary file 1 — Supplementary Material 1 [file 13024_2026_943_MOESM1_ESM.pdf]

Table S1. Phenotypic parameters of the human adipose tissue lipidomics study, including gender, age range, and BMI range, related to Figure 1.

Group	Parameter	Range	Mean
Non-obese (n=6)	Age (years)	45–66	55.5
	BMI (kg/m^2)	25.0–28.1	25.55
	Sex	5 male / 1 female	
Obese (n=6)	Age (years)	27–54	43.0
	BMI (kg/m^2)	38.0–47.3	43.98
	Sex	5 female / 1 male	

Table S2. Proteome data of 5XFAD mice with vehicle, Chow^{EVs}-PE^{low}, and HFD^{EVs}-PE^{high} administration, related to Figure 3H.

Accession	Description	Sum PEP Sc	Coverage	Score Sequ	# Peptides	# PSMs	# Unique Pe	MW [kDa]	Ctrl Abundance	Ctrl Abundance	Chow^{EVs}-PE^{low} Abundance Rat	Chow^{EVs}-PE^{low} Abundance Ra	Chow^{EVs}-PE^{low} Abundance Ra	HFD^{EVs}-PE^{high} Abundance R	HFD^{EVs}-PE^{high} Abundance Ra	HFD^{EVs}-PE^{high} Abundance F	HFD^{EVs}-PE^{high} Abundance R	Ctrl Abundance	Ctrl Abundance	Chow^{EVs}-PE^{low} Abundance Ra	Chow^{EVs}-PE^{low} Abundance Ra	Chow^{EVs}-PE^{low} Abundance Ra	HFD^{EVs}-PE^{high} Abundance F	HFD^{EVs}-PE^{high} Abundance Ra	HFD^{EVs}-PE^{high} Abundance F	HFD^{EVs}-PE^{high} Abundance	Abundance	Ctrl Abundance	Ctrl Abundance	Chow^{EVs}-PE^{low} Abundance: F	Chow^{EVs}-PE^{low} Abundance: F	Chow^{EVs}-PE^{low} Abundance: F	HFD^{EVs}-PE^{high} Abundance: F	HFD^{EVs}-PE^{high} Abundance: F	HFD^{EVs}-PE^{high} Abundance: F1	HFD^{EVs}-PE^{high} Abundance:	Abundance	Ctrl Abundance	Ctrl Abundance	Chow^{EVs}-PE^{low} Abundance: F2	Chow^{EVs}-PE^{low} Abundance: F2	Chow^{EVs}-PE^{low} Abundance: F2	HFD^{EVs}-PE^{high} Abundance: F	HFD^{EVs}-PE^{high} Abundance: F	HFD^{EVs}-PE^{high} Abundance:	HFD^{EVs}-PE^{high} Abundance	emPAI
P16546	Spectrin alpha chain, non-erythrocytic 1 OS=Mus musculus OX=10090 GN=Sptan1 PE=1 SV=4	1372.626	65.85761	1537.52	150	416	150	284.4	1.205	1.069	1.236	0.925	1.197	1.206	1.261	0.896	1.243	1.226	1.066	1.259	0.943	1.217	1.224	1.293	0.923	1.265	30766.3	37074.6	32902.7	38031.3	28459.6	36816.9	37094.5	38789.2	27573.8	38227.8	32480.1	39807.5	34632.1	40900.2	30643.9	39534.3	39757.6	42004.8	29971.7	41079.4	15.885
Q62261	Spectrin beta chain, non-erythrocytic 1 OS=Mus musculus OX=10090 GN=Sptbn1 PE=1 SV=2	846.05	51.41769	905.21	107	249	100	274.1	1.221	1.073	1.235	0.939	1.23	1.206	1.29	0.883	1.259	1.242	1.075	1.257	0.943	1.23	1.22	1.294	0.9	1.272	19605.6	23942.5	21028	24212.8	18417	24106.7	23635.5	25284.7	17318.8	24690.6	21725	26976.8	23358.6	27302.8	20492.2	26721.2	26495.9	28109.4	19553.7	27634	7.432
Q68FD5	Clathrin heavy chain 1 OS=Mus musculus OX=10090 GN=Cltc PE=1 SV=3	809.899	61.55224	1118.36	85	290	85	191.4	1.287	1.074	1.265	0.922	1.201	1.223	1.284	0.918	1.23	1.301	1.093	1.289	0.948	1.228	1.258	1.315	0.935	1.275	21207.2	27294.6	22775.4	26831.6	19548.1	25471.2	25933.8	27239.7	19478.4	26084.3	17886.5	23276	19549.4	23047.4	16960.5	21970.7	22496.5	23517.4	16725.1	22807.7	27.355
Q6PIC6	Sodium/potassium-transporting ATPase subunit alpha-3 OS=Mus musculus OX=10090 GN=Atp1a3 PE=1 SV=1	758.358	59.82231	2285.5	70	578	41	111.6	1.192	1.009	1.182	0.933	1.191	1.182	1.222	0.837	1.218	1.179	1.008	1.188	0.937	1.208	1.193	1.23	0.84	1.218	31436.2	37476.8	31724.7	37168.8	29317.8	37431.9	37155.6	38409.4	26320.6	38301	38416.9	45288.8	38710.5	45629	36014.3	46406.7	45848.5	47235.8	32260.1	46791.2	214.443
Q9JHU4	Cytoplasmic dynein 1 heavy chain 1 OS=Mus musculus OX=10090 GN=Dync1h1 PE=1 SV=2	721.574	30.92162	716.25	114	206	114	531.7	1.285	1.083	1.251	0.945	1.237	1.233	1.311	0.924	1.287	1.276	1.054	1.259	0.95	1.237	1.23	1.304	0.911	1.283	15480	19884.4	16759.6	19372.7	14632.3	19154.5	19084.1	20295.1	14297.9	19920.6	16321.4	20820	17207.3	20541.4	15500	20191	20074.2	21285.8	14874	20940.6	2.063
Q6PIE5	Sodium/potassium-transporting ATPase subunit alpha-2 OS=Mus musculus OX=10090 GN=Atp1a2 PE=1 SV=1	607.521	55.19608	1288.72	63	348	35	112.1	1.292	1.059	1.205	0.94	1.192	1.265	1.299	0.843	1.245	1.308	1.072	1.231	0.962	1.207	1.252	1.306	0.853	1.245	6371.8	8235.4	6746.8	7680.5	5991.3	7594.6	8060.4	8279.3	5373.2	7934	7419.1	9701.9	7950.9	9130	7137.7	8956.5	9285.8	9686.6	6325.3	9240.3	68.069
P20357	Microtubule-associated protein 2 OS=Mus musculus OX=10090 GN=Map2 PE=1 SV=2	595.019	46.82713	594.88	78	170	78	199	1.151	0.998	1.188	0.918	1.17	1.159	1.202	0.893	1.202	1.118	0.969	1.151	0.899	1.158	1.118	1.158	0.865	1.163	13756.2	15826.6	13726	16339	12624.3	16088.7	15942.9	16531.6	12288	16536.9	14175.7	15852.3	13732.4	16313.5	12745.9	16420.9	15843.2	16418.6	12267.7	16482.8	8.38
Q9QYR6	Microtubule-associated protein 1A OS=Mus musculus OX=10090 GN=Map1a PE=1 SV=2	535.694	34.76225	577.44	80	182	79	300	1.171	1.069	1.232	0.946	1.233	1.215	1.268	0.897	1.263	1.147	1.033	1.205	0.941	1.223	1.193	1.241	0.858	1.244	11903.7	13944.5	12725.5	14661.3	11264.2	14671.8	14460.2	15092.9	10680.5	15028.8	13558.1	15549	14006.4	16339.6	12759.7	16575.4	16178.2	16821.3	11627.4	16863.7	5.084
Q8VDN2	Sodium/potassium-transporting ATPase subunit alpha-1 OS=Mus musculus OX=10090 GN=Atp1a1 PE=1 SV=1	524.176	47.89834	1296.96	51	343	25	112.9	1.208	1.051	1.21	0.929	1.204	1.184	1.234	0.92	1.256	1.155	1.011	1.172	0.923	1.16	1.151	1.19	0.9	1.203	7696.4	9296.9	8089.2	9309.7	7146.2	9266.4	9110.7	9500.3	7077.8	9664.8	7649.5	8834.2	7731.4	8964.1	7056.8	8875	8807.3	9105.1	6887.6	9200	34.112
P63017	Heat shock cognate 71 kDa protein OS=Mus musculus OX=10090 GN=Hspa8 PE=1 SV=1	507.54	82.50774	1001.9	50	256	37	70.8	1.279	1.068	1.257	0.932	1.222	1.249	1.295	0.902	1.258	1.254	1.048	1.247	0.924	1.222	1.221	1.291	0.905	1.258	14906	19067.7	15918.4	18743.5	13890.9	18213.6	18616.4	19303	13444.1	18744.8	15348.8	19248.2	16088.6	19145.9	14185.6	18760.2	18741.4	19817.9	13898.3	19306.9	190.448
Q7TMM9	Tubulin beta-2A chain OS=Mus musculus OX=10090 GN=Tubb2a PE=1 SV=1	433.282	70.11236	1457.75	32	418	7	49.9	1.244	1.022	1.257	0.923	1.214	1.243	1.265	0.925	1.237	1.255	1.016	1.255	0.944	1.23	1.249	1.268	0.937	1.282	5476.5	6815.5	5599.3	6883.6	5057	6647.5	6806.2	6927.4	5064.6	6776.4	5745	7208.7	5839.5	7208.7	5423.7	7068.9	7174.2	7286	5382.8	7365.5	1822.348
P07724	Serum albumin OS=Mus musculus OX=10090 GN=Alb PE=1 SV=3	426.38	71.38158	606.31	46	180	46	68.6	1.252	1.141	1.174	1.066	1.125	1.239	1.494	1.113	1.588	1.207	1.106	1.125	1.051	1.103	1.193	1.45	1.068	1.539	12974	16247.3	14802.9	15225.5	13828.9	14601.6	16072.7	19383.2	14441.7	20600.3	12596.4	15199.4	13936.9	14176	13243.9	13896.7	15028.4	18259	13458.7	19389.1	64.156
P68372	Tubulin beta-4B chain OS=Mus musculus OX=10090 GN=Tubb4b PE=1 SV=1	420.385	76.17978	1422.52	33	421	2	49.8	1.201	1.007	1.212	0.906	1.196	1.191	1.253	0.884	1.234	1.226	1.012	1.234	0.907	1.211	1.213	1.263	0.901	1.238	21990.3	26404.2	22147.7	26642.7	19924.2	26306.2	26199.1	27544.7	19446.6	27126.4	23993.5	29410.1	24273.6	29599.4	21769.7	29044.2	29115.5	30310.1	21616.3	29698.1	1349.314
P56480	ATP synthase subunit beta, mitochondrial OS=Mus musculus OX=10090 GN=Atp5f1b PE=1 SV=2	414.69	78.82798	780.16	28	207	28	56.3	1.205	1.005	1.184	0.9	1.184	1.177	1.237	0.897	1.201	1.221	1.043	1.213	0.926	1.207	1.205	1.272	0.897	1.225	11138.1	13421.4	11197.6	13183.8	10021.9	13184.3	13109	13781.5	9990.3	13374.1	12878.9	15726.6	13430.1	15617	11924.3	15551	15513.7	16382.4	11558.8	15778.8	67.978
P39053	Dynamin-1 OS=Mus musculus OX=10090 GN=Dnm1 PE=1 SV=2	404.221	65.74394	582.95	51	150	34	97.7	1.228	1.051	1.212	0.908	1.181	1.159	1.239	0.914	1.217	1.221	1.041	1.232	0.918	1.199	1.184	1.245	0.896	1.232	10207.4	12538.7	10729.5	12368.2	9272.4	12052.8	11830.9	12650.9	9331.3	12421.9	8834.7	10785.1	9195.8	10880.6	8106.2	10590.6	10458.6	10998.8	7917.8	10887.1	17.139
P16330	2',3'-cyclic-nucleotide 3'-phosphodiesterase OS=Mus musculus OX=10090 GN=Cnp PE=1 SV=3	394.395	69.04762	534.92	39	140	38	47.1	1.336	1.082	1.254	0.961	1.259	1.304	1.346	0.805	1.259	1.262	1.075	1.245	0.98	1.269	1.312	1.328	0.776	1.267	9556.4	12766.4	10341.7	11979.9	9181.7	12028.4	12461	12863.5	7688.6	12036.2	12856.6	16230.7	13815.4	16009.9	12602	16312.5	16871.9	17073.2	9973.5	16290.6	113.976
P63038	60 kDa heat shock protein, mitochondrial OS=Mus musculus OX=10090 GN=Hspd1 PE=1 SV=1	392.757	71.90227	590.52	35	162	35	60.9	1.243	1.021	1.227	0.932	1.232	1.234	1.284	0.902	1.261	1.254	1.037	1.221	0.911	1.196	1.221	1.27	0.889	1.251	6699	8327	6841.8	8216.7	6241.9	8251.5	8267.6	8599.3	6039.3	8447.7	9544.4	11965.7	9894.2	11652	8697.4	11412.6	11654.1	12118.8	8481.4	11939.6	37.312
Q9D6F9	Tubulin beta-4A chain OS=Mus musculus OX=10090 GN=Tubb4a PE=1 SV=3	390.493	75.45045	1405.38	29	409	7	49.6	1.212	1.021	1.172	0.918	1.237	1.241	1.275	0.864	1.214	1.267	1.07	1.254	0.959	1.261	1.285	1.332	0.91	1.283	3185.1	3859.3	3253.3	3731.4	2924.3	3939.6	3953.6	4060.5	2750.4	3866.5	2908.1	3685.1	3112.1	3645.7	2790.3	3667.3	3737.1	3874.4	2645.1	3730.1	495.195
P99024	Tubulin beta-5 chain OS=Mus musculus OX=10090 GN=Tubb5 PE=1 SV=1	385.013	70.04505	1074.09	30	315	4	49.6	1.194	1.058	1.267	0.96	1.246	1.23	1.277	0.9	1.265	1.3	1.125	1.351	1.003	1.306	1.322	1.383	0.955	1.347	2165	2584.5	2291.1	2742.1	2079.1	2698.6	2662.1	2764.7	1948.1	2739	1989.1	2585.7	2237.5	2688.1	1996	2597.8	2629.6	2750	1899.9	2679.3	903.736

O88844	Isocitrate dehydrogenase [NADP] cytoplasmic OS=Mus musculus OX=10090 GN=Idh1 PE=1 SV=2	78.804	35.2657	62.99	13
P28660	Nck-associated protein 1 OS=Mus musculus OX=10090 GN=Nckap1 PE=1 SV=2	78.727	15.42553	73.03	15
Q8BG05	Heterogeneous nuclear ribonucleoprotein A3 OS=Mus musculus OX=10090 GN=Hnrnpa3 PE=1 SV=1	78.102	33.24538	74.55	13
O88342	WD repeat-containing protein 1 OS=Mus musculus OX=10090 GN=Wdr1 PE=1 SV=3	77.626	30.033	78.91	13
Q05920	Pyruvate carboxylase, mitochondrial OS=Mus musculus OX=10090 GN=Pc PE=1 SV=1	77.589	14.43124	80.04	12
Q9R0Y5	Adenylate kinase isoenzyme 1 OS=Mus musculus OX=10090 GN=Ak1 PE=1 SV=1	76.753	55.15464	70.74	12
Q8K310	Matrin-3 OS=Mus musculus OX=10090 GN=Matr3 PE=1 SV=1	75.855	16.66667	95.63	12
Q9D394	Protein RUFY3 OS=Mus musculus OX=10090 GN=Rufy3 PE=1 SV=1	75.436	33.04904	87.23	14
P84075	Neuron-specific calcium-binding protein hippocalcin OS=Mus musculus OX=10090 GN=Hpca PE=1 SV=2	75.182	77.20207	130.79	15
Q91V92	ATP-citrate synthase OS=Mus musculus OX=10090 GN=Acly PE=1 SV=1	74.972	19.43171	84.52	16
Q4KMM3	Oxidation resistance protein 1 OS=Mus musculus OX=10090 GN=Oxr1 PE=1 SV=3	74.944	19.16859	63.41	14
Q9D8E6	60S ribosomal protein L4 OS=Mus musculus OX=10090 GN=Rpl4 PE=1 SV=3	74.912	22.67303	75.18	9
P62835	Ras-related protein Rap-1A OS=Mus musculus OX=10090 GN=Rap1a PE=1 SV=1	74.893	52.71739	71.58	8
P70404	Isocitrate dehydrogenase [NAD] subunit gamma 1, mitochondrial OS=Mus musculus OX=10090 GN=Idh3g PE=1	74.786	27.22646	69.93	7
P31650	Sodium- and chloride-dependent GABA transporter 3 OS=Mus musculus OX=10090 GN=Slc6a11 PE=1 SV=2	74.528	15.78947	85.56	10
Q9CWS0	N(G),N(G)-dimethylarginine dimethylaminohydrolase 1 OS=Mus musculus OX=10090 GN=Ddah1 PE=1 SV=3	74.487	60.70175	96.15	15
Q9QYB8	Beta-adducin OS=Mus musculus OX=10090 GN=Add2 PE=1 SV=4	74.414	19.44828	75.25	12
P80317	T-complex protein 1 subunit zeta OS=Mus musculus OX=10090 GN=Cct6a PE=1 SV=3	73.726	33.33333	106.04	13
P14869	60S acidic ribosomal protein P0 OS=Mus musculus OX=10090 GN=Rplp0 PE=1 SV=3	73.245	38.80126	83.15	10
P31938	Dual specificity mitogen-activated protein kinase kinase 1 OS=Mus musculus OX=10090 GN=Map2k1 PE=1 SV=2	73.128	27.22646	102.69	11
[illegible]	[illegible]	[illegible]	[illegible]	[illegible]	[illegible]

P28740	Kinesin-like protein KIF2A OS=Mus musculus OX=10090 GN=Kif2a PE=1 SV=2	38.704	16.17021	39.12	9	13	9	79.7	1.175	1.006	1.16	[illegible]
Q99K51	Plastin-3 OS=Mus musculus OX=10090 GN=Pls3 PE=1 SV=3	38.569	17.30159	29.72	8	10	8	70.7	1.404	1.142	1.339	[illegible]
Q61885	Myelin-oligodendrocyte glycoprotein OS=Mus musculus OX=10090 GN=Mog PE=1 SV=1	38.566	29.26829	49.65	8	15	8	28.3	1.3	1.06	1.185	[illegible]
P32037	Solute carrier family 2, facilitated glucose transporter member 3 OS=Mus musculus OX=10090 GN=Slc2a3 PE=1	38.528	16.8357	35.61	7	12	7	53.4	1.039	0.938	1.077	[illegible]
Q9CWJ9	Bifunctional purine biosynthesis protein PURH OS=Mus musculus OX=10090 GN=Atic PE=1 SV=2	38.434	15.70946	36.67	6	9	6	64.2	1.306	1.064	1.287	[illegible]
P19536	Cytochrome c oxidase subunit 5B, mitochondrial OS=Mus musculus OX=10090 GN=Cox5b PE=1 SV=1	38.313	42.1875	95.98	6	33	5	13.8	1.288	0.892	1.017	[illegible]
Q9QY76	Vesicle-associated membrane protein-associated protein B OS=Mus musculus OX=10090 GN=Vapb PE=1 SV=3	38.288	39.9177	44.42	8	14	7	26.9	1.359	1.085	1.302	[illegible]
Q8R1Q8	Cytoplasmic dynein 1 light intermediate chain 1 OS=Mus musculus OX=10090 GN=Dync1li1 PE=1 SV=1	38.22	13.00191	40.05	6	11	6	56.6	1.247	1.077	1.217	[illegible]
Q64523	Histone H2A type 2-C OS=Mus musculus OX=10090 GN=Hist2h2ac PE=1 SV=3	38.15	41.86047	195.68	6	59	1	14				[illegible]
Q8BKC5	Importin-5 OS=Mus musculus OX=10090 GN=Ipo5 PE=1 SV=3	38.135	12.21513	32.07	8	10	8	123.5	1.305	1.053	1.267	[illegible]
P97450	ATP synthase-coupling factor 6, mitochondrial OS=Mus musculus OX=10090 GN=Atp5pf PE=1 SV=1	38.09	46.2963	48.63	5	15	5	12.5	1.103	0.985	1.148	[illegible]
Q9DCZ4	MICOS complex subunit Mic26 OS=Mus musculus OX=10090 GN=Apoo PE=1 SV=2	38.082	35.85859	40.25	5	11	5	22.6	1.231	1.064	1.189	[illegible]
Q80U28	MAP kinase-activating death domain protein OS=Mus musculus OX=10090 GN=Madd PE=1 SV=2	37.865	7.79962	35.86	9	13	9	175.1	1.183	1.037	1.183	[illegible]
P57746	V-type proton ATPase subunit D OS=Mus musculus OX=10090 GN=Atp6v1d PE=1 SV=1	37.843	28.74494	41.28	8	14	8	28.4	1.15	1.073	1.187	[illegible]
Q9DCN2	NADH-cytochrome b5 reductase 3 OS=Mus musculus OX=10090 GN=Cyb5r3 PE=1 SV=3	37.833	27.57475	39.64	6	11	6	34.1	1.323	1.079	1.267	[illegible]
Q8R5M8	Cell adhesion molecule 1 OS=Mus musculus OX=10090 GN=Cadm1 PE=1 SV=2	37.79	23.90351	32.36	5	6	5	49.8	1.1	1.044	1.194	[illegible]
Q9DCJ5	NADH dehydrogenase [ubiquinone] 1 alpha subcomplex subunit 8 OS=Mus musculus OX=10090 GN=Ndufa8 PE=	37.561	31.97674	44.67	4	10	4	20	1.302	1.083	1.227	[illegible]
Q8BW75	Amine oxidase [flavin-containing] B OS=Mus musculus OX=10090 GN=Maob PE=1 SV=4	37.521	22.69231	41.71	9	13	8	58.5	1.341	1.035	1.299	[illegible]
Q8K021	Secretory carrier-associated membrane protein 1 OS=Mus musculus OX=10090 GN=Scamp1 PE=1 SV=1	37.506	28.69822	33.5	6	8	6	38	1.299	1.15	1.357	[illegible]
Q8R071	Inositol-trisphosphate 3-kinase A OS=Mus musculus OX=10090 GN=Itpka PE=1 SV=1	37.479	19.38998	32.79	7	10	7	50.9	1.143	0.974	1.109	[illegible]
P35979	60S ribosomal protein L12 OS=Mus musculus OX=10090 GN=Rpl12 PE=1 SV=2	37.47	48.48485	33.39	6	10	6	17.8	1.338	1.132	1.298	[illegible]
Q9ESW4	Acylglycerol kinase, mitochondrial OS=Mus musculus OX=10090 GN=Agk PE=1 SV=1	37.444	14.96437	30.2	6	9	6	46.9	1.26	1.086	1.252	[illegible]
Q8CGK7	Guanine nucleotide-binding protein G(olf) subunit alpha OS=Mus musculus OX=10090 GN=Gnal PE=1 SV=1	37.389	22.83465	41.66	5	11	3	44.3	1.431	1.036	1.285	[illegible]
Q8CGY8	UDP-N-acetylglucosamine--peptide N-acetylglucosaminyltransferase 110 kDa subunit OS=Mus musculus OX=10	37.375	9.751434	36.36	7	12	7	116.9	1.262	1.132	1.321	[illegible]
P23818	Glutamate receptor 1 OS=Mus musculus OX=10090 GN=Gria1 PE=1 SV=1	37.27	10.9151	33.86	8	13	4	101.5	1.284	1.102	1.272	[illegible]
P62881	Guanine nucleotide-binding protein subunit beta-5 OS=Mus musculus OX=10090 GN=Gnb5 PE=1 SV=1	37.206	15.18987	38.34	5	13	5	43.5	1.267	1.029	1.223	[illegible]
P63321	Ras-related protein Ral-A OS=Mus musculus OX=10090 GN=Rala PE=1 SV=1	37.174	19.90291	42.95	4	12	4	23.5	1.183	1.006	1.183	[illegible]
P48678	Prelamin-A/C OS=Mus musculus OX=10090 GN=Lmna PE=1 SV=2	37.11	12.03008	44.45	8	13	6	74.2	1.269	1.08	1.246	[illegible]
Q2PFD7	PH and SEC7 domain-containing protein 3 OS=Mus musculus OX=10090 GN=Psd3 PE=1 SV=2	37.065	9.257473	29.84	9	10	9	114.7	1.131	1.03	1.153	[illegible]
G5E8K5	Ankyrin-3 OS=Mus musculus OX=10090 GN=Ank3 PE=1 SV=1	36.853	5.099439	39.38	8	12	5	213.9	1.25	1.102	1.309	[illegible]
Q8CCT4	Transcription elongation factor A protein-like 5 OS=Mus musculus OX=10090 GN=Tceal5 PE=1 SV=1	36.828	33.5	48.36	7	18	7	22	1.097	0.96	1.136	[illegible]
Q9CR16	Peptidyl-prolyl cis-trans isomerase D OS=Mus musculus OX=10090 GN=Ppid PE=1 SV=3	36.709	22.7027	36.08	10	13	9	40.7	1.254	1.057	1.228	[illegible]
Q9CPP6	NADH dehydrogenase [ubiquinone] 1 alpha subcomplex subunit 5 OS=Mus musculus OX=10090 GN=Ndufa5 PE=	36.627	43.10345	46.83	5	11	5	13.4	1.313	1.119	1.334	[illegible]
O88741	Ganglioside-induced differentiation-associated protein 1 OS=Mus musculus OX=10090 GN=Gdap1 PE=1 SV=1	36.625	21.22905	35.71	8	11	8	41.3	1.239	1.077	1.27	[illegible]
[illegible]	[illegible]	[illegible]	[illegible]	[illegible]	[illegible]	[illegible]	[illegible]	[illegible]	[illegible]	[illegible]	[illegible]	[illegible]

[illegible]

Q9WVA2	Mitochondrial import inner membrane translocase subunit Tim8 A OS=Mus musculus OX=10090 GN=Timm8a1	22.437	38.14433	14.43	[illegible]
Q9R1R2	Tripartite motif-containing protein 3 OS=Mus musculus OX=10090 GN=Trim3 PE=1 SV=1	22.413	6.989247	13.37	[illegible]
Q9D7S9	Charged multivesicular body protein 5 OS=Mus musculus OX=10090 GN=Chmp5 PE=1 SV=1	22.396	29.22374	20.28	[illegible]
O70435	Proteasome subunit alpha type-3 OS=Mus musculus OX=10090 GN=Psma3 PE=1 SV=3	22.381	17.64706	23.48	[illegible]
Q3UPL0	Protein transport protein Sec31A OS=Mus musculus OX=10090 GN=Sec31a PE=1 SV=2	22.368	7.235772	15.98	[illegible]
P08414	Calcium/calmodulin-dependent protein kinase type IV OS=Mus musculus OX=10090 GN=Camk4 PE=1 SV=2	22.316	16.84435	21.8	[illegible]
Q9WVJ2	26S proteasome non-ATPase regulatory subunit 13 OS=Mus musculus OX=10090 GN=Psmd13 PE=1 SV=1	22.273	18.08511	17.35	[illegible]
Q9JIA1	Leucine-rich glioma-inactivated protein 1 OS=Mus musculus OX=10090 GN=Lgi1 PE=1 SV=1	22.262	10.77199	28.94	[illegible]
P61021	Ras-related protein Rab-5B OS=Mus musculus OX=10090 GN=Rab5b PE=1 SV=1	22.164	22.32558	20.91	[illegible]
O70591	Prefoldin subunit 2 OS=Mus musculus OX=10090 GN=Pfdn2 PE=1 SV=2	22.108	22.72727	15.69	[illegible]
Q3UMR5	Calcium uniporter protein, mitochondrial OS=Mus musculus OX=10090 GN=Mcu PE=1 SV=2	22.031	17.42857	24.39	[illegible]
Q99JX4	Eukaryotic translation initiation factor 3 subunit M OS=Mus musculus OX=10090 GN=Eif3m PE=1 SV=1	22.013	18.71658	19.36	[illegible]
Q9CQ60	6-phosphogluconolactonase OS=Mus musculus OX=10090 GN=Pgls PE=1 SV=1	21.99	31.51751	13.03	[illegible]
P52760	2-iminobutanoate/2-iminopropanoate deaminase OS=Mus musculus OX=10090 GN=Rida PE=1 SV=3	21.943	32.59259	27.39	[illegible]
P70414	Sodium/calcium exchanger 1 OS=Mus musculus OX=10090 GN=Slc8a1 PE=1 SV=1	21.892	5.979381	22.29	[illegible]
Q9Z2W9	Glutamate receptor 3 OS=Mus musculus OX=10090 GN=Gria3 PE=1 SV=2	21.862	6.193694	25.02	[illegible]
Q3UIU2	NADH dehydrogenase [ubiquinone] 1 beta subcomplex subunit 6 OS=Mus musculus OX=10090 GN=Ndufb6 PE=	21.839	25	24.6	[illegible]
Q9Z0H8	CAP-Gly domain-containing linker protein 2 OS=Mus musculus OX=10090 GN=Clip2 PE=1 SV=2	21.769	5.82617	14.85	[illegible]
Q63912	Oligodendrocyte-myelin glycoprotein OS=Mus musculus OX=10090 GN=Omg PE=1 SV=1	21.712	16.81818	25.08	[illegible]
P63248	cAMP-dependent protein kinase inhibitor alpha OS=Mus musculus OX=10090 GN=Pkia PE=1 SV=2	21.652	42.10526	14.68	[illegible]
Q9JHK4	Geranylgeranyl transferase type-2 subunit alpha OS=Mus musculus OX=10090 GN=Rabggta PE=1 SV=1	21.648	8.818342	21.17	[illegible]
P01831	Thy-1 membrane glycoprotein OS=Mus musculus OX=10090 GN=Thy1 PE=1 SV=1	21.647	14.81481	26.8	[illegible]
Q9EPL8	Importin-7 OS=Mus musculus OX=10090 GN=Ipo7 PE=1 SV=2	21.627	5.876686	20.42	[illegible]
Q7TMY8	E3 ubiquitin-protein ligase HUWE1 OS=Mus musculus OX=10090 GN=Huwe1 PE=1 SV=5	21.558	1.827736	18.66	[illegible]
P62334	26S proteasome regulatory subunit 10B OS=Mus musculus OX=10090 GN=Psmc6 PE=1 SV=1	21.549	17.73779	27.4	[illegible]
Q9JKB1	Ubiquitin carboxyl-terminal hydrolase isozyme L3 OS=Mus musculus OX=10090 GN=Uchl3 PE=1 SV=2	21.543	22.17391	20.38	[illegible]
P14685	26S proteasome non-ATPase regulatory subunit 3 OS=Mus musculus OX=10090 GN=Psmd3 PE=1 SV=3	21.489	12.64151	19.22	[illegible]
Q9JM52	Misshapen-like kinase 1 OS=Mus musculus OX=10090 GN=Mink1 PE=1 SV=3	21.483	5.275229	16.95	[illegible]
Q8R5C5	Beta-centractin OS=Mus musculus OX=10090 GN=Actr1b PE=1 SV=1	21.468	20.47872	37.08	[illegible]
P70202	Latexin OS=Mus musculus OX=10090 GN=Lxn PE=1 SV=2	21.45	17.56757	18.74	[illegible]
Q91ZX7	Prolow-density lipoprotein receptor-related protein 1 OS=Mus musculus OX=10090 GN=Lrp1 PE=1 SV=1	21.418	1.540154	19.27	[illegible]
Q8BLF1	Neutral cholesterol ester hydrolase 1 OS=Mus musculus OX=10090 GN=Nceh1 PE=1 SV=1	21.413	20.09804	29	[illegible]
Q8VBV7	COP9 signalosome complex subunit 8 OS=Mus musculus OX=10090 GN=Cops8 PE=1 SV=1	21.387	23.92344	9.88	[illegible]
Q61831	Mitogen-activated protein kinase 10 OS=Mus musculus OX=10090 GN=Mapk10 PE=1 SV=2	21.345	11.63793	17.81	[illegible]
P60521	Gamma-aminobutyric acid receptor-associated protein-like 2 OS=Mus musculus OX=10090 GN=Gabarapl2 PE=	21.334	29.05983	22.7	[illegible]
Q9JMG7	Hepatoma-derived growth factor-related protein 3 OS=Mus musculus OX=10090 GN=Hdgfl3 PE=1 SV=2	21.259	23.26733	27.37	[illegible]
P83940	Elongin-C OS=Mus musculus OX=10090 GN=Eloc PE=1 SV=1	21.242	30.35714	21.02	[illegible]
Q922R8	Protein disulfide-isomerase A6 OS=Mus musculus OX=10090 GN=Pdia6 PE=1 SV=3	21.185	8.409091	17.98	[illegible]
Q8BKZ9	Pyruvate dehydrogenase protein X component, mitochondrial OS=Mus musculus OX=10090 GN=Pdhx PE=1 SV=	21.176	10.17964	32.24	[illegible]
Q64516	Glycerol kinase OS=Mus musculus OX=10090 GN=Gk PE=1 SV=2	21.144	5.724508	21.57	[illegible]
Q8VCT3	Aminopeptidase B OS=Mus musculus OX=10090 GN=Rnpep PE=1 SV=2	21.028	7.230769	12.99	[illegible]
P32067	Lupus La protein homolog OS=Mus musculus OX=10090 GN=Ssb PE=1 SV=1	21.006	10.60241	24.65	[illegible]
Q5SVL6	Rap1 GTPase-activating protein 2 OS=Mus musculus OX=10090 GN=Rap1gap2 PE=1 SV=1	20.919	9.97191	16.89	[illegible]
P28656	Nucleosome assembly protein 1-like 1 OS=Mus musculus OX=10090 GN=Nap1l1 PE=1 SV=2	20.899	13.04348	24.6	[illegible]
Q9QYB1	Chloride intracellular channel protein 4 OS=Mus musculus OX=10090 GN=Clic4 PE=1 SV=3	20.785	18.18182	14.83	[illegible]
Q9D164	FXYD domain-containing ion transport regulator 6 OS=Mus musculus OX=10090 GN=Fxyd6 PE=1 SV=2	20.71	37.23404	20.92	[illegible]
O70493	Sorting nexin-12 OS=Mus musculus OX=10090 GN=Snx12 PE=1 SV=1	20.655	24.84848	26.76	[illegible]
Q06890	Clusterin OS=Mus musculus OX=10090 GN=Clu PE=1 SV=1	20.631	12.5	23.9	[illegible]
P62843	40S ribosomal protein S15 OS=Mus musculus OX=10090 GN=Rps15 PE=1 SV=2	20.629	41.37931	22.24	[illegible]
P31648	Sodium- and chloride-dependent GABA transporter 1 OS=Mus musculus OX=10090 GN=Slc6a1 PE=1 SV=2	20.611	7.846411	22.8	[illegible]
Q80T41	Gamma-aminobutyric acid type B receptor subunit 2 OS=Mus musculus OX=10090 GN=Gabbr2 PE=1 SV=2	20.582	7.12766	16.2	[illegible]
Q6P5F9	Exportin-1 OS=Mus musculus OX=10090 GN=Xpo1 PE=1 SV=1	20.545	9.990663	25.51	[illegible]
P62071	Ras-related protein R-Ras2 OS=Mus musculus OX=10090 GN=Rras2 PE=1 SV=1	20.537	23.03922	11.3	[illegible]
O88545	COP9 signalosome complex subunit 6 OS=Mus musculus OX=10090 GN=Cops6 PE=1 SV=1	20.533	20.06173	20.34	[illegible]
Q6PA06	Atlastin-2 OS=Mus musculus OX=10090 GN=Atl2 PE=1 SV=1	20.523	9.262436	18.42	[illegible]
E9Q401	Ryanodine receptor 2 OS=Mus musculus OX=10090 GN=Ryr2 PE=1 SV=1	20.492	1.147805	12.95	[illegible]
B0V2N1	Receptor-type tyrosine-protein phosphatase S OS=Mus musculus OX=10090 GN=Ptprs PE=1 SV=1	20.483	2.93655	12.48	[illegible]
Q8R5J9	PRA1 family protein 3 OS=Mus musculus OX=10090 GN=Arl6ip5 PE=1 SV=2	20.477	10.6383	14.86	[illegible]
Q61701	ELAV-like protein 4 OS=Mus musculus OX=10090 GN=Elavl4 PE=1 SV=1	20.423	16.88312	22.35	[illegible]
P11404	Fatty acid-binding protein, heart OS=Mus musculus OX=10090 GN=Fabp3 PE=1 SV=5	20.394	26.31579	25.66	[illegible]
P36536	GTP-binding protein SAR1a OS=Mus musculus OX=10090 GN=Sar1a PE=1 SV=1	20.334	17.17172	18.56	[illegible]
Q9WVA4	Transgelin-2 OS=Mus musculus OX=10090 GN=Tagln2 PE=1 SV=4	20.333	21.60804	20.38	[illegible]
O88712	C-terminal-binding protein 1 OS=Mus musculus OX=10090 GN=Ctbp1 PE=1 SV=2	20.309	10.43084	20.47	[illegible]
Q9CPQ1	Cytochrome c oxidase subunit 6C OS=Mus musculus OX=10090 GN=Cox6c PE=1 SV=3	20.29	27.63158	26.31	[illegible]
Q8BK30	NADH dehydrogenase [ubiquinone] flavoprotein 3, mitochondrial OS=Mus musculus OX=10090 GN=Ndufv3 PE=	20.286	30.76923	28.55	[illegible]
Q91WK5	Glycine cleavage system H protein, mitochondrial OS=Mus musculus OX=10090 GN=Gcsh PE=1 SV=2	20.277	17.64706	23.14	[illegible]
Q8BGH2	Sorting and assembly machinery component 50 homolog OS=Mus musculus OX=10090 GN=Samm50 PE=1 SV=1	20.262	12.36674	22.21	[illegible]
P62774	Myotrophin OS=Mus musculus OX=10090 GN=Mtpn PE=1 SV=2	20.213	33.05085	17.28	[illegible]
Q9D0S9	Histidine triad nucleotide-binding protein 2, mitochondrial OS=Mus musculus OX=10090 GN=Hint2 PE=1 SV=1	20.213	31.90184	13.51	[illegible]
A2AGT5	Cytoskeleton-associated protein 5 OS=Mus musculus OX=10090 GN=Ckap5 PE=1 SV=1	20.186	4.527559	17.89	[illegible]
Q8BL66	Early endosome antigen 1 OS=Mus musculus OX=10090 GN=Eea1 PE=1 SV=2	20.123	4.890149	15.86	[illegible]
Q91ZP9	N-terminal EF-hand calcium-binding protein 2 OS=Mus musculus OX=10090 GN=Necab2 PE=1 SV=1	20.121	11.82519	15.28	[illegible]
Q8CIE6	Coatomer subunit alpha OS=Mus musculus OX=10090 GN=Copa PE=1 SV=2	20.081	4.166667	16.33	[illegible]
P04925	Major prion protein OS=Mus musculus OX=10090 GN=Prnp PE=1 SV=2	19.979	12.59843	19.64	[illegible]
P67871	Casein kinase II subunit beta OS=Mus musculus OX=10090 GN=Csnk2b PE=1 SV=1	19.923	18.13953	15.44	[illegible]
Q02819	Nucleobindin-1 OS=Mus musculus OX=10090 GN=Nucb1 PE=1 SV=2	19.897	10.23965	28.3	[illegible]
Q91ZJ5	UTP--glucose-1-phosphate uridylyltransferase OS=Mus musculus OX=10090 GN=Ugp2 PE=1 SV=3	19.864	9.645669	18.57	[illegible]
Q925N0	Sideroflexin-5 OS=Mus musculus OX=10090 GN=Sfxn5 PE=1 SV=2	19.864	16.08187	20.1	[illegible]
Q9QYF9	Protein NDRG3 OS=Mus musculus OX=10090 GN=Ndrg3 PE=1 SV=1	19.803	17.86667	17.54	[illegible]
Q9CQ92	Mitochondrial fission 1 protein OS=Mus musculus OX=10090 GN=Fis1 PE=1 SV=1	19.801	15.78947	29.77	[illegible]
Q8BR92	Paralemmin-2 OS=Mus musculus OX=10090 GN=Palm2 PE=1 SV=1	19.779	13.82979	14.28	[illegible]
O35857	Mitochondrial import inner membrane translocase subunit TIM44 OS=Mus musculus OX=10090 GN=Timm44 P	19.72	7.964602	15.48	[illegible]
Q61187	Tumor susceptibility gene 101 protein OS=Mus musculus OX=10090 GN=Tsg101 PE=1 SV=2	19.716	14.0665	15.31	[illegible]
P15209	BDNF/NT-3 growth factors receptor OS=Mus musculus OX=10090 GN=Ntrk2 PE=1 SV=1	19.682	5.968331	9.47	[illegible]
Q01065	Calcium/calmodulin-dependent 3',5'-cyclic nucleotide phosphodiesterase 1B OS=Mus musculus OX=10090 GN	19.63	11.02804	16.71	[illegible]
Q61595	Kinectin OS=Mus musculus OX=10090 GN=Ktn1 PE=1 SV=1	19.52	5.50113	15.29	[illegible]
A2AG50	MAP7 domain-containing protein 2 OS=Mus musculus OX=10090 GN=Map7d2 PE=1 SV=1	19.514	7.93854	16.71	[illegible]
O08989	Ras-related protein M-Ras OS=Mus musculus OX=10090 GN=Mras PE=1 SV=1	19.47	25.96154	16.07	[illegible]
Q99LB6	Methionine adenosyltransferase 2 subunit beta OS=Mus musculus OX=10090 GN=Mat2b PE=1 SV=1	19.465	13.17365	10.31	[illegible]
P16054	Protein kinase C epsilon type OS=Mus musculus OX=10090 GN=Prkce PE=1 SV=1	19.457	9.90502	18.4	[illegible]
Q80VD1	Protein FAM98B OS=Mus musculus OX=10090 GN=Fam98b PE=1 SV=1	19.444	7.692308	15.01	[illegible]
Q91ZR1	Ras-related protein Rab-4B OS=Mus musculus OX=10090 GN=Rab4b PE=1 SV=2	19.423	17.37089	32.15	[illegible]
P28271	Cytoplasmic aconitate hydratase OS=Mus musculus OX=10090 GN=Aco1 PE=1 SV=3	19.417	7.649044	21.66	[illegible]
P70297	Signal transducing adapter molecule 1 OS=Mus musculus OX=10090 GN=Stam PE=1 SV=3	19.403	7.116788	12.37	[illegible]
Q5U3K5	Rab-like protein 6 OS=Mus musculus OX=10090 GN=Rabl6 PE=1 SV=2	19.384	8.551724	12.83	[illegible]
Q9JIF7	Coatomer subunit beta OS=Mus musculus OX=10090 GN=Copb1 PE=1 SV=1	19.351	6.400839	17.24	[illegible]
Q9Z0R4	Intersectin-1 OS=Mus musculus OX=10090 GN=Itsn1 PE=1 SV=2	19.314	2.333722	13.32	[illegible]
Q9JMA1	Ubiquitin carboxyl-terminal hydrolase 14 OS=Mus musculus OX=10090 GN=Usp14 PE=1 SV=3	19.284	14.40162	17.29	[illegible]
Q9DCS9	NADH dehydrogenase [ubiquinone] 1 beta subcomplex subunit 10 OS=Mus musculus OX=10090 GN=Ndufb10 P	19.277	26.70455	21.89	[illegible]
O35658	Complement component 1 Q subcomponent-binding protein, mitochondrial OS=Mus musculus OX=10090 GN	19.273	5.395683	15.45	[illegible]
P97822	Acidic leucine-rich nuclear phosphoprotein 32 family member E OS=Mus musculus OX=10090 GN=Anp32e PE=	19.24	7.307692	15.28	[illegible]
Q5FWK3	Rho GTPase-activating protein 1 OS=Mus musculus OX=10090 GN=Arhgap1 PE=1 SV=1	19.222	12.52847	15.22	[illegible]
P40237	CD82 antigen OS=Mus musculus OX=10090 GN=Cd82 PE=1 SV=1	19.208	12.03008	16.3	[illegible]
P29595	NEDD8 OS=Mus musculus OX=10090 GN=Nedd8 PE=1 SV=2	19.185	24.69136	15.96	[illegible]
P47915	60S ribosomal protein L29 OS=Mus musculus OX=10090 GN=Rpl29 PE=1 SV=2	19.158	23.125	27.61	[illegible]
Q9WUL7	ADP-ribosylation factor-like protein 3 OS=Mus musculus OX=10090 GN=Arl3 PE=1 SV=1	19.15	17.03297	27.19	[illegible]
Q9QUR6	Prolyl endopeptidase OS=Mus musculus OX=10090 GN=Prep PE=1 SV=1	19.139	7.323944	21.79	[illegible]
Q925I1	ATPase family AAA domain-containing protein 3 OS=Mus musculus OX=10090 GN=Atad3 PE=1 SV=1	19.07	9.64467	20.18	[illegible]
Q9DBC7	cAMP-dependent protein kinase type I-alpha regulatory subunit OS=Mus musculus OX=10090 GN=Prkar1a PE=1	19.043	12.59843	20.67	[illegible]
Q9CRD0	OCIA domain-containing protein 1 OS=Mus musculus OX=10090 GN=Ociad1 PE=1 SV=1	19.001	21.05263	19.48	[illegible]
O54901	OX-2 membrane glycoprotein OS=Mus musculus OX=10090 GN=Cd200 PE=1 SV=1	18.984	15.82734	20.32	[illegible]
Q9D2M8	Ubiquitin-conjugating enzyme E2 variant 2 OS=Mus musculus OX=10090 GN=Ube2v2 PE=1 SV=4	18.931	33.7931	33.03	[illegible]
P61971	Nuclear transport factor 2 OS=Mus musculus OX=10090 GN=Nutf2 PE=1 SV=1	18.864	29.92126	22.45	[illegible]
P22599	Alpha-1-antitrypsin 1-2 OS=Mus musculus OX=10090 GN=Serpina1b PE=1 SV=2	18.845	10.65375	23.83	[illegible]
Q922Q8	Leucine-rich repeat-containing protein 59 OS=Mus musculus OX=10090 GN=Lrrc59 PE=1 SV=1	18.838	17.58958	14.34	[illegible]
P51880	Fatty acid-binding protein, brain OS=Mus musculus OX=10090 GN=Fabp7 PE=1 SV=2	18.802	27.27273	19.3	[illegible]
Q9DB27	Malignant T-cell-amplified sequence 1 OS=Mus musculus OX=10090 GN=Mcts1 PE=1 SV=1	18.798	29.83425	13.13	[illegible]
P62751	60S ribosomal protein L23a OS=Mus musculus OX=10090 GN=Rpl23a PE=1 SV=1	18.783	26.28205	25.47	[illegible]
O70252	Heme oxygenase 2 OS=Mus musculus OX=10090 GN=Hmox2 PE=1 SV=1	18.644	14.60317	18.15	[illegible]
P55066	Neurocan core protein OS=Mus musculus OX=10090 GN=Ncan PE=1 SV=1	18.622	4.022082	17.83	[illegible]
Q9CQC7	NADH dehydrogenase [ubiquinone] 1 beta subcomplex subunit 4 OS=Mus musculus OX=10090 GN=Ndufb4 PE=	18.593	35.65891	25.78	[illegible]
Q9D8W5	26S proteasome non-ATPase regulatory subunit 12 OS=Mus musculus OX=10090 GN=Psmd12 PE=1 SV=4	18.588	9.649123	20.95	[illegible]
Q8BHN3	Neutral alpha-glucosidase AB OS=Mus musculus OX=10090 GN=Ganab PE=1 SV=1	18.587	4.555085	12.67	[illegible]
O54865	Guanylate cyclase soluble subunit beta-1 OS=Mus musculus OX=10090 GN=Gucy1b1 PE=1 SV=1	18.586	7.419355	18.97	[illegible]
Q920P5	Adenylate kinase isoenzyme 5 OS=Mus musculus OX=10090 GN=Ak5 PE=1 SV=2	18.56	8.718861	15.98	[illegible]
Q9DBE8	Alpha-1,3/1,6-mannosyltransferase ALG2 OS=Mus musculus OX=10090 GN=Alg2 PE=1 SV=2	18.465	21.20482	22.83	[illegible]
Q91VN4	MICOS complex subunit Mic25 OS=Mus musculus OX=10090 GN=Chchd6 PE=1 SV=2	18.395	13.91941	21.33	[illegible]
O09061	Proteasome subunit beta type-1 OS=Mus musculus OX=10090 GN=Psmb1 PE=1 SV=1	18.393	21.66667	12.94	[illegible]
Q9WUA2	Phenylalanine--tRNA ligase beta subunit OS=Mus musculus OX=10090 GN=Farsb PE=1 SV=2	18.377	7.809847	21.1	[illegible]
Q9JLB0	MAGUK p55 subfamily member 6 OS=Mus musculus OX=10090 GN=Mpp6 PE=1 SV=1	18.374	5.786618	14.41	[illegible]
Q7TPM6	Fibronectin type III and SPRY domain-containing protein 1 OS=Mus musculus OX=10090 GN=Fsd1 PE=1 SV=1	18.336	13.50806	12.91	[illegible]
Q07076	Annexin A7 OS=Mus musculus OX=10090 GN=Anxa7 PE=1 SV=2	18.335	10.15119	22.16	[illegible]
Q9D9V3	Ethylmalonyl-CoA decarboxylase OS=Mus musculus OX=10090 GN=Echdc1 PE=1 SV=2	18.324	9.31677	9.4	[illegible]
Q60749	KH domain-containing, RNA-binding, signal transduction-associated protein 1 OS=Mus musculus OX=10090 GN	18.322	17.83296	17.14	[illegible]
Q61625	Glutamate receptor ionotropic, delta-2 OS=Mus musculus OX=10090 GN=Grid2 PE=1 SV=1	18.313	4.865938	13.6	[illegible]
P47809	Dual specificity mitogen-activated protein kinase kinase 4 OS=Mus musculus OX=10090 GN=Map2k4 PE=1 SV=2	18.266	7.052897	12.91	[illegible]
Q8VED9	Galectin-related protein OS=Mus musculus OX=10090 GN=Lgalsl PE=1 SV=1	18.192	22.67442	22.69	[illegible]
Q9D0I9	Arginine--tRNA ligase, cytoplasmic OS=Mus musculus OX=10090 GN=Rars PE=1 SV=2	18.168	8.939394	18.43	[illegible]
Q8VHK5	Membrane protein MLC1 OS=Mus musculus OX=10090 GN=Mlc1 PE=1 SV=1	18.119	8.115183	16	[illegible]
Q9QZB7	Actin-related protein 10 OS=Mus musculus OX=10090 GN=Actr10 PE=1 SV=2	18.116	14.6283	19.72	[illegible]
P41105	60S ribosomal protein L28 OS=Mus musculus OX=10090 GN=Rpl28 PE=1 SV=2	18.089	21.89781	21.26	[illegible]
O09114	Prostaglandin-H2 D-isomerase OS=Mus musculus OX=10090 GN=Ptgds PE=1 SV=1	18.077	16.93122	13.59	[illegible]
Q78PY7	Staphylococcal nuclease domain-containing protein 1 OS=Mus musculus OX=10090 GN=Snd1 PE=1 SV=1	18.057	6.483516	12.91	[illegible]
Q8CAY6	Acetyl-CoA acetyltransferase, cytosolic OS=Mus musculus OX=10090 GN=Acat2 PE=1 SV=2	18.006	14.35768	16.66	[illegible]
Q00612	Glucose-6-phosphate 1-dehydrogenase X OS=Mus musculus OX=10090 GN=G6pdx PE=1 SV=3	17.987	13.39806	17.78	[illegible]
P20108	Thioredoxin-dependent peroxide reductase, mitochondrial OS=Mus musculus OX=10090 GN=Prdx3 PE=1 SV=1	17.979	19.84436	17.49	[illegible]
Q8R2R9	AP-3 complex subunit mu-2 OS=Mus musculus OX=10090 GN=Ap3m2 PE=1 SV=1	17.953	7.894737	13.71	[illegible]
Q80Z38	SH3 and multiple ankyrin repeat domains protein 2 OS=Mus musculus OX=10090 GN=Shank2 PE=1 SV=2	17.912	4.132791	15.26	[illegible]
P63082	V-type proton ATPase 16 kDa proteolipid subunit OS=Mus musculus OX=10090 GN=Atp6v0c PE=1 SV=1	17.77	33.54839	12.22	[illegible]
Q9Z0H4	CUGBP Elav-like family member 2 OS=Mus musculus OX=10090 GN=Celf2 PE=1 SV=1	17.736	9.055118	15.1	[illegible]
Q62446	Peptidyl-prolyl cis-trans isomerase FKBP3 OS=Mus musculus OX=10090 GN=Fkbp3 PE=1 SV=2	17.699	17.41071	17.62	[illegible]
Q9JM76	Actin-related protein 2/3 complex subunit 3 OS=Mus musculus OX=10090 GN=Arpc3 PE=1 SV=3	17.667	19.66292	24.82	[illegible]
Q9CZY3	Ubiquitin-conjugating enzyme E2 variant 1 OS=Mus musculus OX=10090 GN=Ube2v1 PE=1 SV=1	17.648	29.2517	28.64	[illegible]
P62075	Mitochondrial import inner membrane translocase subunit Tim13 OS=Mus musculus OX=10090 GN=Timm13 P	17.642	42.10526	19.51	[illegible]
Q00493	Carboxypeptidase E OS=Mus musculus OX=10090 GN=Cpe PE=1 SV=2	17.604	11.34454	24.1	[illegible]
Q9CYZ2	Tumor protein D54 OS=Mus musculus OX=10090 GN=Tpd52l2 PE=1 SV=1	17.591	9.545455	11.32	[illegible]
Q61545	RNA-binding protein EWS OS=Mus musculus OX=10090 GN=Ewsr1 PE=1 SV=2	17.589	4.580153	17.27	[illegible]
O08795	Glucosidase 2 subunit beta OS=Mus musculus OX=10090 GN=Prkcsh PE=1 SV=1	17.578	6.333973	19.13	[illegible]
P70663	SPARC-like protein 1 OS=Mus musculus OX=10090 GN=Sparcl1 PE=1 SV=3	17.578	7.384615	15.92	[illegible]
Q9CQI6	Coactosin-like protein OS=Mus musculus OX=10090 GN=Cotl1 PE=1 SV=3	17.552	33.80282	29.79	[illegible]
Q62442	Vesicle-associated membrane protein 1 OS=Mus musculus OX=10090 GN=Vamp1 PE=1 SV=1	17.524	14.40678	15.25	[illegible]
P07758	Alpha-1-antitrypsin 1-1 OS=Mus musculus OX=10090 GN=Serpina1a PE=1 SV=4	17.511	12.10654	23.7	[illegible]
Q9WU28	Prefoldin subunit 5 OS=Mus musculus OX=10090 GN=Pfdn5 PE=1 SV=1	17.494	25.97403	17.18	[illegible]
Q6ZWV7	60S ribosomal protein L35 OS=Mus musculus OX=10090 GN=Rpl35 PE=1 SV=1	17.486	33.33333	25.06	[illegible]
Q8K212	Phosphofurin acidic cluster sorting protein 1 OS=Mus musculus OX=10090 GN=Pacs1 PE=1 SV=2	17.442	5.41103	16.83	[illegible]
Q8R191	Synaptogyrin-3 OS=Mus musculus OX=10090 GN=Syngr3 PE=1 SV=1	17.436	11.79039	22.34	[illegible]
Q66JS6	Eukaryotic translation initiation factor 3 subunit J-B OS=Mus musculus OX=10090 GN=Eif3j2 PE=1 SV=1	17.383	12.92776	13.13	[illegible]
P62702	40S ribosomal protein S4, X isoform OS=Mus musculus OX=10090 GN=Rps4x PE=1 SV=2	17.38	14.8289	17.25	[illegible]
Q9D5V5	Cullin-5 OS=Mus musculus OX=10090 GN=Cul5 PE=1 SV=3	17.328	7.435897	13.16	[illegible]
Q99J85	Neuronal pentraxin receptor OS=Mus musculus OX=10090 GN=Nptxr PE=1 SV=1	17.279	6.490872	16.63	[illegible]
Q3UHX2	28 kDa heat- and acid-stable phosphoprotein OS=Mus musculus OX=10090 GN=Pdap1 PE=1 SV=1	17.191	19.33702	25.02	[illegible]
Q99LD8	N(G),N(G)-dimethylarginine dimethylaminohydrolase 2 OS=Mus musculus OX=10090 GN=Ddah2 PE=1 SV=1	17.185	12.63158	16.62	[illegible]
Q9D1R9	60S ribosomal protein L34 OS=Mus musculus OX=10090 GN=Rpl34 PE=1 SV=2	17.182	34.18803	21.43	[illegible]
P62484	Abl interactor 2 OS=Mus musculus OX=10090 GN=Abi2 PE=1 SV=1	17.14	7.623318	22.38	[illegible]
Q9D154	Leukocyte elastase inhibitor A OS=Mus musculus OX=10090 GN=Serpinb1a PE=1 SV=1	17.127	12.1372	16.86	[illegible]
Q9CQ75	NADH dehydrogenase [ubiquinone] 1 alpha subcomplex subunit 2 OS=Mus musculus OX=10090 GN=Ndufa2 PE=	17.074	36.36364	29.18	[illegible]
Q8C1A5	Thimet oligopeptidase OS=Mus musculus OX=10090 GN=Thop1 PE=1 SV=1	17.074	6.113537	20.66	[illegible]
Q8BHE3	Caytaxin OS=Mus musculus OX=10090 GN=Atcay PE=1 SV=1	17.073	11.02151	10.51	[illegible]
Q91Z31	Polypyrimidine tract-binding protein 2 OS=Mus musculus OX=10090 GN=Ptbp2 PE=1 SV=2	17.021	5.838041	13.3	[illegible]
Q9EPW0	Inositol polyphosphate-4-phosphatase type I A OS=Mus musculus OX=10090 GN=Inpp4a PE=1 SV=1	17.004	4.792332	15.63	[illegible]
Q9DAS9	Guanine nucleotide-binding protein G(I)/G(S)/G(O) subunit gamma-12 OS=Mus musculus OX=10090 GN=Gng12	16.994	37.5	17.5	[illegible]
Q71M36	Chondroitin sulfate proteoglycan 5 OS=Mus musculus OX=10090 GN=Cspg5 PE=1 SV=2	16.968	5.123675	9.86	[illegible]
Q8C854	Myelin expression factor 2 OS=Mus musculus OX=10090 GN=Myef2 PE=1 SV=1	16.966	9.137056	14.71	[illegible]
Q8CA95	cAMP and cAMP-inhibited cGMP 3',5'-cyclic phosphodiesterase 10A OS=Mus musculus OX=10090 GN=Pde10a	16.944	5.063291	13.39	[illegible]
Q5RJI5	Serine/threonine-protein kinase BRSK1 OS=Mus musculus OX=10090 GN=Brsk1 PE=1 SV=1	16.94	6.812339	14.51	[illegible]
Q61016	Guanine nucleotide-binding protein G(I)/G(S)/G(O) subunit gamma-7 OS=Mus musculus OX=10090 GN=Gng7 P	16.923	33.82353	20.87	[illegible]
Q91ZU6	Dystonin OS=Mus musculus OX=10090 GN=Dst PE=1 SV=2	16.885	0.784526	13.64	[illegible]
Q8BLJ3	PI-PLC X domain-containing protein 3 OS=Mus musculus OX=10090 GN=Plcxd3 PE=1 SV=1	16.805	18.06854	11.5	[illegible]
Q8K400	Syntaxin-binding protein 5 OS=Mus musculus OX=10090 GN=Stxbp5 PE=1 SV=3	16.764	3.038194	9.25	[illegible]
P24472	Glutathione S-transferase A4 OS=Mus musculus OX=10090 GN=Gsta4 PE=1 SV=3	16.763	27.47748	23.91	[illegible]
Q91XD7	Cysteine-rich with EGF-like domain protein 1 OS=Mus musculus OX=10090 GN=Creld1 PE=1 SV=1	16.728	11.19048	15.39	[illegible]
Q8JZU2	Tricarboxylate transport protein, mitochondrial OS=Mus musculus OX=10090 GN=Slc25a1 PE=1 SV=1	16.72	9.003215	20.42	[illegible]
E9Q6P5	Tetratricopeptide repeat protein 7B OS=Mus musculus OX=10090 GN=Ttc7b PE=1 SV=1	16.715	4.744958	16.46	[illegible]
Q6PDL0	Cytoplasmic dynein 1 light intermediate chain 2 OS=Mus musculus OX=10090 GN=Dync1li2 PE=1 SV=2	16.663	11.17886	12.79	[illegible]
O08583	THO complex subunit 4 OS=Mus musculus OX=10090 GN=Alyref PE=1 SV=3	16.66	15.29412	9	[illegible]
Q9D7X3	Dual specificity protein phosphatase 3 OS=Mus musculus OX=10090 GN=Dusp3 PE=1 SV=1	16.656	18.91892	12.01	[illegible]
Q8VCW8	Medium-chain acyl-CoA ligase ACSF2, mitochondrial OS=Mus musculus OX=10090 GN=Acsf2 PE=1 SV=1	16.638	8.455285	19.25	[illegible]
Q61035	Histidine--tRNA ligase, cytoplasmic OS=Mus musculus OX=10090 GN=Hars PE=1 SV=2	16.624	10.21611	12.87	[illegible]
P62317	Small nuclear ribonucleoprotein Sm D2 OS=Mus musculus OX=10090 GN=Snrpd2 PE=1 SV=1	16.586	16.94915	14.67	[illegible]
P35980	60S ribosomal protein L18 OS=Mus musculus OX=10090 GN=Rpl18 PE=1 SV=3	16.576	11.70213	17.21	[illegible]
Q3UHB1	5'-nucleotidase domain-containing protein 3 OS=Mus musculus OX=10090 GN=Nt5dc3 PE=1 SV=1	16.541	10.07326	21.47	[illegible]
P56812	Programmed cell death protein 5 OS=Mus musculus OX=10090 GN=Pdcd5 PE=1 SV=3	16.464	32.53968	18.48	[illegible]
Q3UGR5	Haloacid dehalogenase-like hydrolase domain-containing protein 2 OS=Mus musculus OX=10090 GN=Hdhd2 P	16.387	14.28571	17.36	[illegible]
Q9R1P3	Proteasome subunit beta type-2 OS=Mus musculus OX=10090 GN=Psmb2 PE=1 SV=1	16.317	18.90547	17.82	[illegible]
Q8CBE3	WD repeat-containing protein 37 OS=Mus musculus OX=10090 GN=Wdr37 PE=1 SV=1	16.252	6.25	17.87	[illegible]
Q9Z1W9	STE20/SPS1-related proline-alanine-rich protein kinase OS=Mus musculus OX=10090 GN=Stk39 PE=1 SV=1	16.206	6.47482	13.91	[illegible]
Q91VZ6	Stromal membrane-associated protein 1 OS=Mus musculus OX=10090 GN=Smap1 PE=1 SV=1	16.188	9.772727	15.13	[illegible]
Q9WV98	Mitochondrial import inner membrane translocase subunit Tim9 OS=Mus musculus OX=10090 GN=Timm9 PE=	16.151	38.20225	17.86	[illegible]
O70439	Syntaxin-7 OS=Mus musculus OX=10090 GN=Stx7 PE=1 SV=3	16.136	14.17625	13.42	[illegible]
Q9CQX8	28S ribosomal protein S36, mitochondrial OS=Mus musculus OX=10090 GN=Mrps36 PE=1 SV=1	16.109	26.47059	11.88	[illegible]
Q9CZR8	Elongation factor Ts, mitochondrial OS=Mus musculus OX=10090 GN=Tsfm PE=1 SV=1	16.081	5.246914	8.87	[illegible]
Q9CZ04	COP9 signalosome complex subunit 7a OS=Mus musculus OX=10090 GN=Cops7a PE=1 SV=2	16.029	20	8.35	[illegible]
Q99LF4	tRNA-splicing ligase RtcB homolog OS=Mus musculus OX=10090 GN=Rtcb PE=1 SV=1	16.022	6.732673	18.09	[illegible]
P60761	Neurogranin OS=Mus musculus OX=10090 GN=Nrgn PE=1 SV=1	16.005	32.05128	24.42	[illegible]
Q8VD33	Small glutamine-rich tetratricopeptide repeat-containing protein beta OS=Mus musculus OX=10090 GN=Sgtb P	15.996	23.02632	12.14	[illegible]
Q91VM9	Inorganic pyrophosphatase 2, mitochondrial OS=Mus musculus OX=10090 GN=Ppa2 PE=1 SV=1	15.99	13.0303	11.9	[illegible]
P12660	Purkinje cell protein 2 OS=Mus musculus OX=10090 GN=Pcp2 PE=1 SV=2	15.961	30.83333	13.83	[illegible]
Q61335	B-cell receptor-associated protein 31 OS=Mus musculus OX=10090 GN=Bcap31 PE=1 SV=4	15.951	11.83673	20.51	[illegible]
Q99KB8	Hydroxyacylglutathione hydrolase, mitochondrial OS=Mus musculus OX=10090 GN=Hagh PE=1 SV=2	15.874	7.119741	17.14	[illegible]
P24270	Catalase OS=Mus musculus OX=10090 GN=Cat PE=1 SV=4	15.865	5.882353	7.03	[illegible]
P32020	Non-specific lipid-transfer protein OS=Mus musculus OX=10090 GN=Scp2 PE=1 SV=3	15.835	5.667276	14.53	[illegible]
Q8K3H0	DCC-interacting protein 13-alpha OS=Mus musculus OX=10090 GN=Appl1 PE=1 SV=1	15.767	4.950495	15.99	[illegible]
Q9CQN1	Heat shock protein 75 kDa, mitochondrial OS=Mus musculus OX=10090 GN=Trap1 PE=1 SV=1	15.761	2.832861	27.76	[illegible]
Q9CQD1	Ras-related protein Rab-5A OS=Mus musculus OX=10090 GN=Rab5a PE=1 SV=1	15.75	22.32558	19.98	[illegible]
Q8C166	Copine-1 OS=Mus musculus OX=10090 GN=Cpne1 PE=1 SV=1	15.719	7.462687	12.43	[illegible]
Q02357	Ankyrin-1 OS=Mus musculus OX=10090 GN=Ank1 PE=1 SV=2	15.678	2.792696	18.33	[illegible]
Q9Z2W8	Glutamate receptor 4 OS=Mus musculus OX=10090 GN=Gria4 PE=1 SV=2	15.658	5.764967	16.4	[illegible]
Q61481	Calcium/calmodulin-dependent 3',5'-cyclic nucleotide phosphodiesterase 1A OS=Mus musculus OX=10090 GN	15.645	8.318584	13.28	[illegible]
Q9QYI5	DnaJ homolog subfamily B member 2 OS=Mus musculus OX=10090 GN=Dnajb2 PE=1 SV=3	15.626	11.41975	14.95	[illegible]
Q9WTP6	Adenylate kinase 2, mitochondrial OS=Mus musculus OX=10090 GN=Ak2 PE=1 SV=5	15.61	24.26778	15.87	[illegible]
P02798	Metallothionein-2 OS=Mus musculus OX=10090 GN=Mt2 PE=1 SV=2	15.609	32.78689	10.1	[illegible]
P35585	AP-1 complex subunit mu-1 OS=Mus musculus OX=10090 GN=Ap1m1 PE=1 SV=3	15.607	11.11111	11.95	[illegible]
Q8BJY1	26S proteasome non-ATPase regulatory subunit 5 OS=Mus musculus OX=10090 GN=Psmd5 PE=1 SV=4	15.591	8.730159	11.48	[illegible]
Q3V3R1	Monofunctional C1-tetrahydrofolate synthase, mitochondrial OS=Mus musculus OX=10090 GN=Mthfd1l PE=1	15.585	4.094166	10.62	[illegible]
Q6PCP5	Mitochondrial fission factor OS=Mus musculus OX=10090 GN=Mff PE=1 SV=1	15.503	17.86942	11.58	[illegible]
Q9DC70	NADH dehydrogenase [ubiquinone] iron-sulfur protein 7, mitochondrial OS=Mus musculus OX=10090 GN=Ndu	15.479	26.33929	20.39	[illegible]
Q9CR95	Adaptin ear-binding coat-associated protein 1 OS=Mus musculus OX=10090 GN=Necap1 PE=1 SV=2	15.473	13.09091	19.73	[illegible]
Q6X893	Choline transporter-like protein 1 OS=Mus musculus OX=10090 GN=Slc44a1 PE=1 SV=3	15.439	7.503828	14.23	[illegible]
P59999	Actin-related protein 2/3 complex subunit 4 OS=Mus musculus OX=10090 GN=Arpc4 PE=1 SV=3	15.407	30.35714	24.34	[illegible]
P97765	WW domain-binding protein 2 OS=Mus musculus OX=10090 GN=Wbp2 PE=1 SV=1	15.394	11.49425	10.46	[illegible]
P61759	Prefoldin subunit 3 OS=Mus musculus OX=10090 GN=Vbp1 PE=1 SV=2	15.265	20.91837	16	[illegible]
Q9WUC3	Lymphocyte antigen 6H OS=Mus musculus OX=10090 GN=Ly6h PE=1 SV=2	15.252	28.05755	17.19	[illegible]
P56376	Acylphosphatase-1 OS=Mus musculus OX=10090 GN=Acyp1 PE=1 SV=2	15.187	31.31313	17.76	[illegible]
Q8CIB5	Fermitin family homolog 2 OS=Mus musculus OX=10090 GN=Fermt2 PE=1 SV=1	15.149	6.323529	9.82	[illegible]
Q6P1B1	Xaa-Pro aminopeptidase 1 OS=Mus musculus OX=10090 GN=Xpnpep1 PE=1 SV=1	15.143	10.91493	12	[illegible]
Q9Z1Q9	Valine--tRNA ligase OS=Mus musculus OX=10090 GN=Vars PE=1 SV=1	15.141	3.958828	12.7	[illegible]
O35678	Monoglyceride lipase OS=Mus musculus OX=10090 GN=Mgll PE=1 SV=1	15.113	10.89109	21.84	[illegible]
Q80UU9	Membrane-associated progesterone receptor component 2 OS=Mus musculus OX=10090 GN=Pgrmc2 PE=1 SV=	15.112	24.42396	12.18	[illegible]
Q9D1G5	Leucine-rich repeat-containing protein 57 OS=Mus musculus OX=10090 GN=Lrrc57 PE=1 SV=1	15.065	17.99163	13.04	[illegible]
Q80UJ7	Rab3 GTPase-activating protein catalytic subunit OS=Mus musculus OX=10090 GN=Rab3gap1 PE=1 SV=4	15.05	4.587156	12.88	[illegible]
P26883	Peptidyl-prolyl cis-trans isomerase FKBP1A OS=Mus musculus OX=10090 GN=Fkbp1a PE=1 SV=2	15.001	12.96296	17.16	[illegible]
Q8VBW6	NEDD8-activating enzyme E1 regulatory subunit OS=Mus musculus OX=10090 GN=Nae1 PE=1 SV=1	15.001	11.61049	15.5	[illegible]
O54991	Contactin-associated protein 1 OS=Mus musculus OX=10090 GN=Cntnap1 PE=1 SV=2	14.992	3.032491	11.14	[illegible]
Q8C437	PEX5-related protein OS=Mus musculus OX=10090 GN=Pex5l PE=1 SV=2	14.968	5.820106	8	[illegible]
P63325	40S ribosomal protein S10 OS=Mus musculus OX=10090 GN=Rps10 PE=1 SV=1	14.958	20	17.54	[illegible]
P0DN34	NADH dehydrogenase [ubiquinone] 1 beta subcomplex subunit 1 OS=Mus musculus OX=10090 GN=Ndufb1 PE=	14.954	54.38596	16.66	[illegible]
Q91W50	Cold shock domain-containing protein E1 OS=Mus musculus OX=10090 GN=Csde1 PE=1 SV=1	14.947	5.513784	16.48	[illegible]
O70492	Sorting nexin-3 OS=Mus musculus OX=10090 GN=Snx3 PE=1 SV=3	14.928	19.1358	11.81	[illegible]
Q9QZ88	Vacuolar protein sorting-associated protein 29 OS=Mus musculus OX=10090 GN=Vps29 PE=1 SV=1	14.899	18.68132	14.22	[illegible]
P62918	60S ribosomal protein L8 OS=Mus musculus OX=10090 GN=Rpl8 PE=1 SV=2	14.875	18.67704	18.64	[illegible]
Q91VC3	Eukaryotic initiation factor 4A-III OS=Mus musculus OX=10090 GN=Eif4a3 PE=1 SV=3	14.865	15.08516	9.38	[illegible]
P83510	Traf2 and NCK-interacting protein kinase OS=Mus musculus OX=10090 GN=Tnik PE=1 SV=2	14.861	2.645503	14.54	[illegible]
Q01405	Protein transport protein Sec23A OS=Mus musculus OX=10090 GN=Sec23a PE=1 SV=2	14.797	5.751634	15.39	[illegible]
Q99KK7	Dipeptidyl peptidase 3 OS=Mus musculus OX=10090 GN=Dpp3 PE=1 SV=2	14.782	4.878049	9.75	[illegible]
Q8CHT0	Delta-1-pyrroline-5-carboxylate dehydrogenase, mitochondrial OS=Mus musculus OX=10090 GN=Aldh4a1 PE=	14.773	5.516014	6.04	[illegible]
P19253	60S ribosomal protein L13a OS=Mus musculus OX=10090 GN=Rpl13a PE=1 SV=4	14.77	18.2266	13.36	[illegible]
Q8R5H6	Wiskott-Aldrich syndrome protein family member 1 OS=Mus musculus OX=10090 GN=Wasf1 PE=1 SV=2	14.769	11.44902	13.72	[illegible]
Q9R0X4	Acyl-coenzyme A thioesterase 9, mitochondrial OS=Mus musculus OX=10090 GN=Acot9 PE=1 SV=1	14.762	12.75626	16.57	[illegible]
Q8VHJ5	Serine/threonine-protein kinase MARK1 OS=Mus musculus OX=10090 GN=Mark1 PE=1 SV=2	14.759	6.540881	10.79	[illegible]
Q8R4N0	Citramalyl-CoA lyase, mitochondrial OS=Mus musculus OX=10090 GN=Clybl PE=1 SV=2	14.739	9.763314	9.55	[illegible]
P62270	40S ribosomal protein S18 OS=Mus musculus OX=10090 GN=Rps18 PE=1 SV=3	14.705	22.36842	17.07	[illegible]
P62264	40S ribosomal protein S14 OS=Mus musculus OX=10090 GN=Rps14 PE=1 SV=3	14.681	23.84106	17.18	[illegible]
P14131	40S ribosomal protein S16 OS=Mus musculus OX=10090 GN=Rps16 PE=1 SV=4	14.675	21.91781	17.76	[illegible]
Q9D7A8	Armadillo repeat-containing protein 1 OS=Mus musculus OX=10090 GN=Armc1 PE=1 SV=1	14.613	19.85816	10.62	[illegible]
Q8VDM4	26S proteasome non-ATPase regulatory subunit 2 OS=Mus musculus OX=10090 GN=Psmd2 PE=1 SV=1	14.613	4.185022	15.72	[illegible]
Q5SSM3	Rho GTPase-activating protein 44 OS=Mus musculus OX=10090 GN=Arhgap44 PE=1 SV=1	14.605	5.896806	9.54	[illegible]
Q9CQJ6	Density-regulated protein OS=Mus musculus OX=10090 GN=Denr PE=1 SV=1	14.551	21.21212	10.53	[illegible]
Q99JR1	Sideroflexin-1 OS=Mus musculus OX=10090 GN=Sfxn1 PE=1 SV=3	14.53	15.21739	11.37	[illegible]
O88485	Cytoplasmic dynein 1 intermediate chain 1 OS=Mus musculus OX=10090 GN=Dync1i1 PE=1 SV=2	14.468	7.484076	10.93	[illegible]
P62717	60S ribosomal protein L18a OS=Mus musculus OX=10090 GN=Rpl18a PE=1 SV=1	14.455	22.15909	11.85	[illegible]
O70310	Glycylpeptide N-tetradecanoyltransferase 1 OS=Mus musculus OX=10090 GN=Nmt1 PE=1 SV=1	14.445	7.862903	20.51	[illegible]
Q99JX3	Golgi reassembly-stacking protein 2 OS=Mus musculus OX=10090 GN=Gorasp2 PE=1 SV=3	14.423	7.095344	9.58	[illegible]
Q8CG76	Aflatoxin B1 aldehyde reductase member 2 OS=Mus musculus OX=10090 GN=Akr7a2 PE=1 SV=3	14.41	9.536785	16.98	[illegible]
Q8R164	Valacyclovir hydrolase OS=Mus musculus OX=10090 GN=Bphl PE=1 SV=1	14.383	15.80756	17.73	[illegible]
Q9JLZ3	Methylglutaconyl-CoA hydratase, mitochondrial OS=Mus musculus OX=10090 GN=Auh PE=1 SV=1	14.37	21.01911	14.85	[illegible]
Q9ESJ4	NCK-interacting protein with SH3 domain OS=Mus musculus OX=10090 GN=Nckipsd PE=1 SV=2	14.329	5.042017	9.74	[illegible]
P62267	40S ribosomal protein S23 OS=Mus musculus OX=10090 GN=Rps23 PE=1 SV=3	14.324	13.98601	17.79	[illegible]
Q9JM14	5'(3')-deoxyribonucleotidase, cytosolic type OS=Mus musculus OX=10090 GN=Nt5c PE=1 SV=1	14.307	16	12.72	[illegible]
P18572	Basigin OS=Mus musculus OX=10090 GN=Bsg PE=1 SV=2	14.288	5.912596	13.3	[illegible]
Q8C0L0	Thioredoxin-related transmembrane protein 4 OS=Mus musculus OX=10090 GN=Tmx4 PE=1 SV=2	14.279	8.059701	16.08	[illegible]
P20065	Thymosin beta-4 OS=Mus musculus OX=10090 GN=Tmsb4x PE=1 SV=1	14.22	40	29.38	[illegible]
E9Q3L2	Phosphatidylinositol 4-kinase alpha OS=Mus musculus OX=10090 GN=Pi4ka PE=1 SV=2	14.212	2.470309	11.77	[illegible]
O88448	Kinesin light chain 2 OS=Mus musculus OX=10090 GN=Klc2 PE=1 SV=1	14.166	5.843072	13.04	[illegible]
Q9QUP5	Hyaluronan and proteoglycan link protein 1 OS=Mus musculus OX=10090 GN=Hapln1 PE=1 SV=1	14.145	10.95506	16.35	[illegible]
Q99KQ4	Nicotinamide phosphoribosyltransferase OS=Mus musculus OX=10090 GN=Nampt PE=1 SV=1	14.137	9.979633	10.94	[illegible]
P70699	Lysosomal alpha-glucosidase OS=Mus musculus OX=10090 GN=Gaa PE=1 SV=2	14.1	3.777545	9.24	[illegible]
P37040	NADPH--cytochrome P450 reductase OS=Mus musculus OX=10090 GN=Por PE=1 SV=2	13.977	5.162242	9.01	[illegible]
P50153	Guanine nucleotide-binding protein G(I)/G(S)/G(O) subunit gamma-4 OS=Mus musculus OX=10090 GN=Gng4 P	13.962	53.33333	10.49	[illegible]
Q6PGF7	Exocyst complex component 8 OS=Mus musculus OX=10090 GN=Exoc8 PE=1 SV=1	13.959	5.726257	7.09	[illegible]
P56695	Wolframin OS=Mus musculus OX=10090 GN=Wfs1 PE=1 SV=1	13.945	4.94382	8.94	[illegible]
Q80U23	Syntaphilin OS=Mus musculus OX=10090 GN=Snph PE=1 SV=3	13.933	10.30303	12.42	[illegible]
Q6WVG3	BTB/POZ domain-containing protein KCTD12 OS=Mus musculus OX=10090 GN=Kctd12 PE=1 SV=1	13.932	13.45566	17.25	[illegible]
Q6ZWN5	40S ribosomal protein S9 OS=Mus musculus OX=10090 GN=Rps9 PE=1 SV=3	13.909	24.74227	13.4	[illegible]
Q60692	Proteasome subunit beta type-6 OS=Mus musculus OX=10090 GN=Psmb6 PE=1 SV=3	13.871	13.86555	16.09	[illegible]
O35841	Apoptosis inhibitor 5 OS=Mus musculus OX=10090 GN=Api5 PE=1 SV=2	13.866	9.325397	12.69	[illegible]
Q9EQU5	Protein SET OS=Mus musculus OX=10090 GN=Set PE=1 SV=1	13.857	8.304498	18.89	[illegible]
P28658	Ataxin-10 OS=Mus musculus OX=10090 GN=Atxn10 PE=1 SV=2	13.815	9.473684	19.31	[illegible]
Q9CYG7	Mitochondrial import receptor subunit TOM34 OS=Mus musculus OX=10090 GN=Tomm34 PE=1 SV=1	13.795	14.23948	12.22	[illegible]
Q4VAE3	Transmembrane protein 65 OS=Mus musculus OX=10090 GN=Tmem65 PE=1 SV=1	13.788	10.25641	18.84	[illegible]
P49442	Inositol polyphosphate 1-phosphatase OS=Mus musculus OX=10090 GN=Inpp1 PE=1 SV=2	13.784	9.343434	16.93	[illegible]
Q9JHI5	Isovaleryl-CoA dehydrogenase, mitochondrial OS=Mus musculus OX=10090 GN=Ivd PE=1 SV=1	13.779	5.660377	9.27	[illegible]
Q9JKD3	Secretory carrier-associated membrane protein 5 OS=Mus musculus OX=10090 GN=Scamp5 PE=1 SV=1	13.724	11.91489	19.25	[illegible]
Q8BTX9	Inactive hydroxysteroid dehydrogenase-like protein 1 OS=Mus musculus OX=10090 GN=Hsdl1 PE=1 SV=1	13.722	6.666667	14.95	[illegible]
Q9D1D4	Transmembrane emp24 domain-containing protein 10 OS=Mus musculus OX=10090 GN=Tmed10 PE=1 SV=1	13.684	16.89498	16.93	[illegible]
Q99PL5	Ribosome-binding protein 1 OS=Mus musculus OX=10090 GN=Rrbp1 PE=1 SV=2	13.679	12.52336	12.01	[illegible]
Q9R0A0	Peroxisomal membrane protein PEX14 OS=Mus musculus OX=10090 GN=Pex14 PE=1 SV=1	13.635	11.43617	9.18	[illegible]
Q80U63	Mitofusin-2 OS=Mus musculus OX=10090 GN=Mfn2 PE=1 SV=3	13.582	5.284016	8.26	[illegible]
Q812A2	SLIT-ROBO Rho GTPase-activating protein 3 OS=Mus musculus OX=10090 GN=Srgap3 PE=1 SV=1	13.566	2.638763	13.71	[illegible]
Q99MI1	ELKS/Rab6-interacting/CAST family member 1 OS=Mus musculus OX=10090 GN=Erc1 PE=1 SV=1	13.535	3.303571	16.75	[illegible]
P50544	Very long-chain specific acyl-CoA dehydrogenase, mitochondrial OS=Mus musculus OX=10090 GN=Acadvl PE=1	13.525	6.707317	12.89	[illegible]
P61079	Ubiquitin-conjugating enzyme E2 D3 OS=Mus musculus OX=10090 GN=Ube2d3 PE=1 SV=1	13.517	23.80952	21.43	[illegible]
Q9DCU2	Plasmolipin OS=Mus musculus OX=10090 GN=Pllp PE=1 SV=1	13.494	21.42857	10.84	[illegible]
Q6W8Q3	Purkinje cell protein 4-like protein 1 OS=Mus musculus OX=10090 GN=Pcp4l1 PE=1 SV=1	13.492	20.58824	11.2	[illegible]
P36916	Guanine nucleotide-binding protein-like 1 OS=Mus musculus OX=10090 GN=Gnl1 PE=1 SV=4	13.476	6.260297	14.62	[illegible]
O88531	Palmitoyl-protein thioesterase 1 OS=Mus musculus OX=10090 GN=Ppt1 PE=1 SV=2	13.418	10.78431	7.18	[illegible]

P32848	Parvalbumin alpha OS=Mus musculus OX=10090 GN=Pvalb PE=1 SV=3	13.396	25.45455	9.53	3
P62301	40S ribosomal protein S13 OS=Mus musculus OX=10090 GN=Rps13 PE=1 SV=2	13.372	33.77483	19.48	5
Q9CQE8	RNA transcription, translation and transport factor protein OS=Mus musculus OX=10090 GN=RTRAF PE=1 SV=1	13.364	16.80328	15.15	4
O88456	Calpain small subunit 1 OS=Mus musculus OX=10090 GN=Capns1 PE=1 SV=1	13.327	17.47212	8.38	3
P57776	Elongation factor 1-delta OS=Mus musculus OX=10090 GN=Eef1d PE=1 SV=3	13.315	10.32028	19.52	3
Q8BXR1	Probable cationic amino acid transporter OS=Mus musculus OX=10090 GN=Slc7a14 PE=1 SV=1	13.313	3.242542	8.37	2
Q9D1E6	Tubulin-folding cofactor B OS=Mus musculus OX=10090 GN=Tbcb PE=1 SV=2	13.309	15.57377	11.88	3
Q8K4Z3	NAD(P)H-hydrate epimerase OS=Mus musculus OX=10090 GN=Naxe PE=1 SV=1	13.29	9.929078	11.38	2
Q921J2	GTP-binding protein Rheb OS=Mus musculus OX=10090 GN=Rheb PE=1 SV=1	13.276	10.32609	10.79	2
P45952	Medium-chain specific acyl-CoA dehydrogenase, mitochondrial OS=Mus musculus OX=10090 GN=Acadm PE=1 S	13.267	10.21378	11.92	2
Q9EST5	Acidic leucine-rich nuclear phosphoprotein 32 family member B OS=Mus musculus OX=10090 GN=Anp32b PE=	13.18	13.60294	16.48	4
Q61543	Golgi apparatus protein 1 OS=Mus musculus OX=10090 GN=Glg1 PE=1 SV=1	13.166	2.723404	13.65	3
P47753	F-actin-capping protein subunit alpha-1 OS=Mus musculus OX=10090 GN=Capza1 PE=1 SV=4	13.153	16.08392	12.38	4
Q8K406	Leucine-rich repeat LGI family member 3 OS=Mus musculus OX=10090 GN=Lgi3 PE=1 SV=1	13.12	6.021898	13.66	3
Q9CY27	Very-long-chain enoyl-CoA reductase OS=Mus musculus OX=10090 GN=Tecr PE=1 SV=1	13.104	9.415584	14.3	3
Q99L04	Dehydrogenase/reductase SDR family member 1 OS=Mus musculus OX=10090 GN=Dhrs1 PE=1 SV=1	13.102	8.626198	7.77	2
Q62348	Translin OS=Mus musculus OX=10090 GN=Tsn PE=1 SV=1	13.083	9.210526	10.56	2
P13020	Gelsolin OS=Mus musculus OX=10090 GN=Gsn PE=1 SV=3	13.036	5.512821	15.92	3
Q99LG2	Transportin-2 OS=Mus musculus OX=10090 GN=Tnpo2 PE=1 SV=1	13.034	3.494927	8.75	2
Q9DCL9	Multifunctional protein ADE2 OS=Mus musculus OX=10090 GN=Paics PE=1 SV=4	13.006	11.05882	11.33	4
Q9QYR9	Acyl-coenzyme A thioesterase 2, mitochondrial OS=Mus musculus OX=10090 GN=Acot2 PE=1 SV=2	12.989	7.505519	12.25	3
P22892	AP-1 complex subunit gamma-1 OS=Mus musculus OX=10090 GN=Ap1g1 PE=1 SV=3	12.968	4.014599	24.61	3
Q91WJ8	Far upstream element-binding protein 1 OS=Mus musculus OX=10090 GN=Fubp1 PE=1 SV=1	12.932	7.987711	17.46	5
Q80Y14	Glutaredoxin-related protein 5, mitochondrial OS=Mus musculus OX=10090 GN=Glrx5 PE=1 SV=2	12.928	20.39474	11.36	2
O54946	DnaJ homolog subfamily B member 6 OS=Mus musculus OX=10090 GN=Dnajb6 PE=1 SV=4	12.927	6.30137	10.36	2
P22005	Proenkephalin-A OS=Mus musculus OX=10090 GN=Penk PE=1 SV=2	12.915	8.955224	14.69	1
Q9WUD1	STIP1 homology and U box-containing protein 1 OS=Mus musculus OX=10090 GN=Stub1 PE=1 SV=1	12.907	11.84211	9.68	3
Q9CZM2	60S ribosomal protein L15 OS=Mus musculus OX=10090 GN=Rpl15 PE=1 SV=4	12.902	18.13725	13.74	3
Q8VEK0	Cell cycle control protein 50A OS=Mus musculus OX=10090 GN=Tmem30a PE=1 SV=1	12.884	9.89011	10.21	3
Q5DQR4	Syntaxin-binding protein 5-like OS=Mus musculus OX=10090 GN=Stxbp5l PE=1 SV=1	12.879	4.472574	6.37	2
Q60972	Histone-binding protein RBBP4 OS=Mus musculus OX=10090 GN=Rbbp4 PE=1 SV=5	12.87	5.882353	7.32	2
Q7TT50	Serine/threonine-protein kinase MRCK beta OS=Mus musculus OX=10090 GN=Cdc42bpb PE=1 SV=2	12.825	1.926445	7.67	2
Q6A4J8	Ubiquitin carboxyl-terminal hydrolase 7 OS=Mus musculus OX=10090 GN=Usp7 PE=1 SV=1	12.819	4.533092	16.92	4
Q8BMK4	Cytoskeleton-associated protein 4 OS=Mus musculus OX=10090 GN=Ckap4 PE=1 SV=2	12.816	4.869565	7.24	2
Q3UYC0	Protein phosphatase 1H OS=Mus musculus OX=10090 GN=Ppm1h PE=1 SV=1	12.814	6.62768	12.68	2
P63141	Potassium voltage-gated channel subfamily A member 2 OS=Mus musculus OX=10090 GN=Kcna2 PE=1 SV=1	12.803	5.611222	11.8	2
O70325	Phospholipid hydroperoxide glutathione peroxidase OS=Mus musculus OX=10090 GN=Gpx4 PE=1 SV=4	12.797	19.79695	14.4	4
Q0VGU4	Neurosecretory protein VGF OS=Mus musculus OX=10090 GN=Vgf PE=1 SV=1	12.789	5.186386	8.02	3
Q9WV80	Sorting nexin-1 OS=Mus musculus OX=10090 GN=Snx1 PE=1 SV=1	12.788	6.130268	13.97	3
P15327	Bisphosphoglycerate mutase OS=Mus musculus OX=10090 GN=Bpgm PE=1 SV=2	12.715	10.42471	14.17	2
Q99NE5	Regulating synaptic membrane exocytosis protein 1 OS=Mus musculus OX=10090 GN=Rims1 PE=1 SV=2	12.682	2.460697	6.53	3
Q61206	Platelet-activating factor acetylhydrolase IB subunit beta OS=Mus musculus OX=10090 GN=Pafah1b2 PE=1 SV=2	12.658	18.34061	22.09	4
Q6P9K9	Neurexin-3 OS=Mus musculus OX=10090 GN=Nrxn3 PE=1 SV=2	12.634	2.100573	11.19	3
P62082	40S ribosomal protein S7 OS=Mus musculus OX=10090 GN=Rps7 PE=2 SV=1	12.601	14.94845	13.75	3
Q99LI8	Hepatocyte growth factor-regulated tyrosine kinase substrate OS=Mus musculus OX=10090 GN=Hgs PE=1 SV=2	12.573	3.870968	12.81	3
Q91VR8	Protein BRICK1 OS=Mus musculus OX=10090 GN=Brk1 PE=1 SV=1	12.572	45.33333	10.87	4
Q91Z53	Glyoxylate reductase/hydroxypyruvate reductase OS=Mus musculus OX=10090 GN=Grhpr PE=1 SV=1	12.566	11.58537	16.25	3
Q9ER00	Syntaxin-12 OS=Mus musculus OX=10090 GN=Stx12 PE=1 SV=1	12.552	14.23358	10.4	3
Q9CXW4	60S ribosomal protein L11 OS=Mus musculus OX=10090 GN=Rpl11 PE=1 SV=4	12.517	7.865169	14.31	1
P49615	Cyclin-dependent-like kinase 5 OS=Mus musculus OX=10090 GN=Cdk5 PE=1 SV=1	12.463	14.38356	16.85	3
P84309	Adenylate cyclase type 5 OS=Mus musculus OX=10090 GN=Adcy5 PE=1 SV=2	12.461	3.169572	15.85	3
P35282	Ras-related protein Rab-21 OS=Mus musculus OX=10090 GN=Rab21 PE=1 SV=4	12.457	15.31532	16.75	3
Q8QZS1	3-hydroxyisobutyryl-CoA hydrolase, mitochondrial OS=Mus musculus OX=10090 GN=Hibch PE=1 SV=1	12.453	8.571429	7.72	3
Q9D6U8	Protein FAM162A OS=Mus musculus OX=10090 GN=Fam162a PE=1 SV=1	12.447	15.48387	13.97	2
P62627	Dynein light chain roadblock-type 1 OS=Mus musculus OX=10090 GN=Dynlrb1 PE=1 SV=3	12.424	29.16667	12.65	2
Q811P8	Rho GTPase-activating protein 32 OS=Mus musculus OX=10090 GN=Arhgap32 PE=1 SV=2	12.41	1.914792	9.89	3
O89112	Glutathione S-transferase LANCL1 OS=Mus musculus OX=10090 GN=Lancl1 PE=1 SV=1	12.41	9.022556	17.87	3
P51410	60S ribosomal protein L9 OS=Mus musculus OX=10090 GN=Rpl9 PE=2 SV=2	12.393	29.6875	9.74	3
Q69ZS6	Synaptic vesicle glycoprotein 2C OS=Mus musculus OX=10090 GN=Sv2c PE=1 SV=2	12.383	3.713893	14.16	2
Q9CR00	26S proteasome non-ATPase regulatory subunit 9 OS=Mus musculus OX=10090 GN=Psmd9 PE=1 SV=1	12.379	11.71171	10.84	2
P27612	Phospholipase A-2-activating protein OS=Mus musculus OX=10090 GN=Plaa PE=1 SV=4	12.361	4.534005	12.4	3
Q62393	Tumor protein D52 OS=Mus musculus OX=10090 GN=Tpd52 PE=1 SV=2	12.34	25.44643	9.62	3
Q9R1P1	Proteasome subunit beta type-3 OS=Mus musculus OX=10090 GN=Psmb3 PE=1 SV=1	12.286	15.60976	13.81	3
Q8JZS0	Protein lin-7 homolog A OS=Mus musculus OX=10090 GN=Lin7a PE=1 SV=2	12.282	12.01717	16.14	3
P55821	Stathmin-2 OS=Mus musculus OX=10090 GN=Stmn2 PE=1 SV=1	12.275	19.55307	29.01	4
P16460	Argininosuccinate synthase OS=Mus musculus OX=10090 GN=Ass1 PE=1 SV=1	12.271	13.1068	16.32	4
Q8BG32	26S proteasome non-ATPase regulatory subunit 11 OS=Mus musculus OX=10090 GN=Psmd11 PE=1 SV=3	12.258	9.71564	13.09	3
Q8VCD6	Receptor expression-enhancing protein 2 OS=Mus musculus OX=10090 GN=Reep2 PE=1 SV=2	12.213	9.448819	11.01	1
Q8BHG2	CXXC motif containing zinc binding protein OS=Mus musculus OX=10090 GN=Czib PE=1 SV=1	12.184	20.625	8.45	2
P39688	Tyrosine-protein kinase Fyn OS=Mus musculus OX=10090 GN=Fyn PE=1 SV=4	12.148	5.214153	19.17	2
Q148V7	RAB11-binding protein RELCH OS=Mus musculus OX=10090 GN=Relch PE=1 SV=1	12.13	3.125	9.16	3
P62281	40S ribosomal protein S11 OS=Mus musculus OX=10090 GN=Rps11 PE=1 SV=3	12.109	20.88608	9.75	3
Q5SSL4	Active breakpoint cluster region-related protein OS=Mus musculus OX=10090 GN=Abr PE=1 SV=1	12.108	4.889406	11.56	3
Q9D6Z1	Nucleolar protein 56 OS=Mus musculus OX=10090 GN=Nop56 PE=1 SV=2	12.074	4.827586	5.81	2
Q9CQ62	2,4-dienoyl-CoA reductase, mitochondrial OS=Mus musculus OX=10090 GN=Decr1 PE=1 SV=1	12.023	6.567164	14.6	2
Q8VDQ1	Prostaglandin reductase 2 OS=Mus musculus OX=10090 GN=Ptgr2 PE=1 SV=2	11.98	8.831909	13.6	3
P84089	Enhancer of rudimentary homolog OS=Mus musculus OX=10090 GN=Erh PE=1 SV=1	11.933	33.65385	9.14	3
Q8R366	Immunoglobulin superfamily member 8 OS=Mus musculus OX=10090 GN=Igsf8 PE=1 SV=2	11.926	7.364975	7.69	3
Q99JP6	Homer protein homolog 3 OS=Mus musculus OX=10090 GN=Homer3 PE=1 SV=2	11.884	9.831461	8.16	3
Q6ZPJ3	(E3-independent) E2 ubiquitin-conjugating enzyme UBE2O OS=Mus musculus OX=10090 GN=Ube2o PE=1 SV=3	11.832	2.872671	9.61	3
P46664	Adenylosuccinate synthetase isozyme 2 OS=Mus musculus OX=10090 GN=Adss PE=1 SV=2	11.819	6.798246	9.37	3
Q9DB15	39S ribosomal protein L12, mitochondrial OS=Mus musculus OX=10090 GN=Mrpl12 PE=1 SV=2	11.813	22.38806	14.6	2
P40630	Transcription factor A, mitochondrial OS=Mus musculus OX=10090 GN=Tfam PE=1 SV=2	11.727	11.11111	12.24	3
Q9CQR4	Acyl-coenzyme A thioesterase 13 OS=Mus musculus OX=10090 GN=Acot13 PE=1 SV=1	11.714	21.42857	14.26	3
Q8BJU0	Small glutamine-rich tetratricopeptide repeat-containing protein alpha OS=Mus musculus OX=10090 GN=Sgta	11.698	7.936508	9.08	2
Q9JLV5	Cullin-3 OS=Mus musculus OX=10090 GN=Cul3 PE=1 SV=1	11.671	4.296875	12.24	3
P21300	Aldo-keto reductase family 1 member B7 OS=Mus musculus OX=10090 GN=Akr1b7 PE=1 SV=4	11.658	8.544304	12.23	3
A2A432	Cullin-4B OS=Mus musculus OX=10090 GN=Cul4b PE=1 SV=1	11.607	2.57732	7.39	2
O88746	Target of Myb protein 1 OS=Mus musculus OX=10090 GN=Tom1 PE=1 SV=1	11.606	6.707317	6.69	2
Q8BNW9	Kelch repeat and BTB domain-containing protein 11 OS=Mus musculus OX=10090 GN=Kbtbd11 PE=1 SV=3	11.585	4.897314	8.16	2
Q9CX86	Heterogeneous nuclear ribonucleoprotein A0 OS=Mus musculus OX=10090 GN=Hnrnpa0 PE=1 SV=1	11.539	9.836066	9.49	3
Q924M7	Mannose-6-phosphate isomerase OS=Mus musculus OX=10090 GN=Mpi PE=1 SV=1	11.505	12.76596	13.4	3
Q6NZJ6	Eukaryotic translation initiation factor 4 gamma 1 OS=Mus musculus OX=10090 GN=Eif4g1 PE=1 SV=1	11.472	2.5	8.29	3
Q8R0S4	Voltage-dependent L-type calcium channel subunit beta-4 OS=Mus musculus OX=10090 GN=Cacnb4 PE=1 SV=2	11.462	5.973025	8.22	2
Q9QXK3	Coatomer subunit gamma-2 OS=Mus musculus OX=10090 GN=Copg2 PE=1 SV=1	11.456	5.396096	16.5	3
Q99PL6	UBX domain-containing protein 6 OS=Mus musculus OX=10090 GN=Ubxn6 PE=1 SV=1	11.454	10.181	11.18	4
O70589	Peripheral plasma membrane protein CASK OS=Mus musculus OX=10090 GN=Cask PE=1 SV=2	11.445	2.699784	7.67	2
Q8BGN8	Synaptoporin OS=Mus musculus OX=10090 GN=Synpr PE=1 SV=1	11.421	9.056604	10.78	2
Q9ESN6	Tripartite motif-containing protein 2 OS=Mus musculus OX=10090 GN=Trim2 PE=1 SV=1	11.416	3.629032	7.86	2
Q922J6	Tetraspanin-2 OS=Mus musculus OX=10090 GN=Tspan2 PE=1 SV=1	11.378	8.144796	15.36	2
Q61029	Lamina-associated polypeptide 2, isoforms beta/delta/epsilon/gamma OS=Mus musculus OX=10090 GN=Tmpo	11.34	11.06195	8.48	3
Q9DBG6	Dolichyl-diphosphooligosaccharide--protein glycosyltransferase subunit 2 OS=Mus musculus OX=10090 GN=Rp	11.336	4.912837	6.22	2
Q9QVP9	Protein-tyrosine kinase 2-beta OS=Mus musculus OX=10090 GN=Ptk2b PE=1 SV=2	11.307	1.982161	12.08	2
P26516	26S proteasome non-ATPase regulatory subunit 7 OS=Mus musculus OX=10090 GN=Psmd7 PE=1 SV=2	11.287	8.722741	17.41	2
Q0KL02	Triple functional domain protein OS=Mus musculus OX=10090 GN=Trio PE=1 SV=3	11.232	0.999355	10.49	3
P17665	Cytochrome c oxidase subunit 7C, mitochondrial OS=Mus musculus OX=10090 GN=Cox7c PE=1 SV=1	11.228	28.57143	12.81	2
Q9CQ54	NADH dehydrogenase [ubiquinone] 1 subunit C2 OS=Mus musculus OX=10090 GN=Ndufc2 PE=1 SV=1	11.213	37.5	14.15	4
Q3UH60	Disco-interacting protein 2 homolog B OS=Mus musculus OX=10090 GN=Dip2b PE=1 SV=1	11.136	1.905972	7.04	2
Q8C078	Calcium/calmodulin-dependent protein kinase kinase 2 OS=Mus musculus OX=10090 GN=Camkk2 PE=1 SV=2	11.107	5.102041	13.33	2
Q9DCZ1	GMP reductase 1 OS=Mus musculus OX=10090 GN=Gmpr PE=1 SV=1	11.051	7.246377	11	2
Q62443	Neuronal pentraxin-1 OS=Mus musculus OX=10090 GN=Nptx1 PE=1 SV=1	11.043	8.333333	9.94	4
P09055	Integrin beta-1 OS=Mus musculus OX=10090 GN=Itgb1 PE=1 SV=1	11.043	4.010025	5.9	2
Q8C878	NEDD8-activating enzyme E1 catalytic subunit OS=Mus musculus OX=10090 GN=Uba3 PE=1 SV=2	11.029	6.060606	14.69	2
Q9D4C9	Clavesin-1 OS=Mus musculus OX=10090 GN=Clvs1 PE=1 SV=1	11.014	8.19209	10.14	2
Q8K1Z0	Ubiquinone biosynthesis protein COQ9, mitochondrial OS=Mus musculus OX=10090 GN=Coq9 PE=1 SV=1	11.01	8.945687	11.48	2
Q9R1T2	SUMO-activating enzyme subunit 1 OS=Mus musculus OX=10090 GN=Sae1 PE=1 SV=1	11.003	9.714286	7.59	2
Q99LD4	COP9 signalosome complex subunit 1 OS=Mus musculus OX=10090 GN=Gps1 PE=1 SV=1	10.99	6.794055	12.06	3
Q99KF1	Transmembrane emp24 domain-containing protein 9 OS=Mus musculus OX=10090 GN=Tmed9 PE=1 SV=2	10.951	11.91489	5.59	2
Q9CPW0	Contactin-associated protein-like 2 OS=Mus musculus OX=10090 GN=Cntnap2 PE=1 SV=2	10.948	4.054054	8.33	4
P97823	Acyl-protein thioesterase 1 OS=Mus musculus OX=10090 GN=Lypla1 PE=1 SV=1	10.943	10.86957	10.8	2
Q8VDP6	CDP-diacylglycerol--inositol 3-phosphatidyltransferase OS=Mus musculus OX=10090 GN=Cdipt PE=1 SV=1	10.922	14.55399	9.37	3
Q9D8W7	OCIA domain-containing protein 2 OS=Mus musculus OX=10090 GN=Ociad2 PE=1 SV=1	10.918	14.28571	10.32	2
Q9CR98	Protein FAM136A OS=Mus musculus OX=10090 GN=Fam136a PE=1 SV=1	10.908	23.18841	3.65	2
Q62283	Tetraspanin-7 OS=Mus musculus OX=10090 GN=Tspan7 PE=1 SV=2	10.894	12.4498	10.37	3
Q9CXW3	Calcyclin-binding protein OS=Mus musculus OX=10090 GN=Cacybp PE=1 SV=1	10.866	15.72052	11.74	3
Q9JM63	ATP-sensitive inward rectifier potassium channel 10 OS=Mus musculus OX=10090 GN=Kcnj10 PE=1 SV=1	10.843	3.430079	12.39	1
Q61137	Astrotactin-1 OS=Mus musculus OX=10090 GN=Astn1 PE=1 SV=4	10.843	3.225806	7	3
O35954	Membrane-associated phosphatidylinositol transfer protein 1 OS=Mus musculus OX=10090 GN=Pitpnm1 PE=1	10.788	2.333065	9.72	2
O88703	Potassium/sodium hyperpolarization-activated cyclic nucleotide-gated channel 2 OS=Mus musculus OX=1009(	10.788	5.098494	8.88	3
Q4VAA2	Protein CDV3 OS=Mus musculus OX=10090 GN=Cdv3 PE=1 SV=2	10.78	8.185053	4.75	1
Q5HZI9	Solute carrier family 25 member 51 OS=Mus musculus OX=10090 GN=Slc25a51 PE=1 SV=1	10.751	9.060403	12.91	2
Q9Z2Y8	Pyridoxal phosphate homeostasis protein OS=Mus musculus OX=10090 GN=Plpbp PE=1 SV=1	10.745	14.23358	11.22	4
Q8VEJ9	Vacuolar protein sorting-associated protein 4A OS=Mus musculus OX=10090 GN=Vps4a PE=1 SV=1	10.695	8.237986	17.97	3
Q60865	Caprin-1 OS=Mus musculus OX=10090 GN=Caprin1 PE=1 SV=2	10.62	3.11174	10.23	2
Q6PEB6	MOB-like protein phocein OS=Mus musculus OX=10090 GN=Mob4 PE=1 SV=1	10.582	16.44444	8.58	3
O88851	Putative hydrolase RBBP9 OS=Mus musculus OX=10090 GN=Rbbp9 PE=1 SV=2	10.571	13.44086	6.33	2
Q3TDN2	FAS-associated factor 2 OS=Mus musculus OX=10090 GN=Faf2 PE=1 SV=2	10.564	6.292135	8.64	2
Q5SUR0	Phosphoribosylformylglycinamidine synthase OS=Mus musculus OX=10090 GN=Pfas PE=1 SV=1	10.56	2.243829	12.98	1
Q922Q1	Mitochondrial amidoxime reducing component 2 OS=Mus musculus OX=10090 GN=Marc2 PE=1 SV=1	10.56	8.87574	10.95	4
Q9WTL7	Acyl-protein thioesterase 2 OS=Mus musculus OX=10090 GN=Lypla2 PE=1 SV=1	10.532	18.18182	14	2
A2AJI0	MAP7 domain-containing protein 1 OS=Mus musculus OX=10090 GN=Map7d1 PE=1 SV=1	10.51	4.018913	9.91	3
Q62093	Serine/arginine-rich splicing factor 2 OS=Mus musculus OX=10090 GN=Srsf2 PE=1 SV=4	10.503	7.692308	8.15	1
Q91VW3	SH3 domain-binding glutamic acid-rich-like protein 3 OS=Mus musculus OX=10090 GN=Sh3bgrl3 PE=1 SV=1	10.475	29.03226	11.35	2
Q9EQQ9	Protein O-GlcNAcase OS=Mus musculus OX=10090 GN=Oga PE=1 SV=2	10.433	3.49345	11.75	2
Q8VHI6	Wiskott-Aldrich syndrome protein family member 3 OS=Mus musculus OX=10090 GN=Wasf3 PE=1 SV=1	10.409	3.992016	9.41	2
Q9CQF3	Cleavage and polyadenylation specificity factor subunit 5 OS=Mus musculus OX=10090 GN=Nudt21 PE=1 SV=1	10.406	24.6696	9.58	3
Q9Z2H2	Regulator of G-protein signaling 6 OS=Mus musculus OX=10090 GN=Rgs6 PE=1 SV=2	10.382	5.932203	9.85	2
Q99KV1	DnaJ homolog subfamily B member 11 OS=Mus musculus OX=10090 GN=Dnajb11 PE=1 SV=1	10.359	8.938547	8.22	2
O35864	COP9 signalosome complex subunit 5 OS=Mus musculus OX=10090 GN=Cops5 PE=1 SV=3	10.321	6.586826	7.36	2
Q8C8N2	Protein SCAI OS=Mus musculus OX=10090 GN=Scai PE=1 SV=2	10.302	4.290429	12.49	2
P59325	Eukaryotic translation initiation factor 5 OS=Mus musculus OX=10090 GN=Eif5 PE=1 SV=1	10.298	6.993007	11.67	2
O35226	26S proteasome non-ATPase regulatory subunit 4 OS=Mus musculus OX=10090 GN=Psmd4 PE=1 SV=1	10.289	7.978723	7.46	2
P54822	Adenylosuccinate lyase OS=Mus musculus OX=10090 GN=Adsl PE=1 SV=2	10.263	5.991736	8.21	2
Q02257	Junction plakoglobin OS=Mus musculus OX=10090 GN=Jup PE=1 SV=3	10.254	3.355705	4.69	2
Q6NS52	Diacylglycerol kinase beta OS=Mus musculus OX=10090 GN=Dgkb PE=1 SV=2	10.194	5.112219	8.79	3
Q5DTY9	BTB/POZ domain-containing protein KCTD16 OS=Mus musculus OX=10090 GN=Kctd16 PE=1 SV=2	10.164	8.899297	10.11	4
P28571	Sodium- and chloride-dependent glycine transporter 1 OS=Mus musculus OX=10090 GN=Slc6a9 PE=1 SV=3	10.158	4.624277	12.93	2
Q8C8T8	Pre-rRNA-processing protein TSR2 homolog OS=Mus musculus OX=10090 GN=Tsr2 PE=1 SV=1	10.153	8.376963	7.85	1
Q99LR1	Lysophosphatidylserine lipase ABHD12 OS=Mus musculus OX=10090 GN=Abhd12 PE=1 SV=2	10.14	8.291457	13.15	3
Q9D880	Mitochondrial import inner membrane translocase subunit TIM50 OS=Mus musculus OX=10090 GN=Timm50 P	10.096	7.932011	9.41	2
Q61081	Hsp90 co-chaperone Cdc37 OS=Mus musculus OX=10090 GN=Cdc37 PE=1 SV=1	10.068	7.915567	9.59	4
O35143	ATPase inhibitor, mitochondrial OS=Mus musculus OX=10090 GN=ATP5IF1 PE=1 SV=2	10.047	16.03774	15.13	3
Q8CHT1	Ephexin-1 OS=Mus musculus OX=10090 GN=Ngef PE=1 SV=1	10.043	8.028169	12.53	5
P11031	Activated RNA polymerase II transcriptional coactivator p15 OS=Mus musculus OX=10090 GN=Sub1 PE=1 SV=3	10.036	25.98425	13.14	3
O35551	Rab GTPase-binding effector protein 1 OS=Mus musculus OX=10090 GN=Rabep1 PE=1 SV=2	10.03	3.596288	13.31	2
Q80Z24	Neuronal growth regulator 1 OS=Mus musculus OX=10090 GN=Negr1 PE=1 SV=1	10.028	6.609195	7.96	2
Q6ZWY8	Thymosin beta-10 OS=Mus musculus OX=10090 GN=Tmsb10 PE=1 SV=3	10.025	45.45455	27.77	4
P70195	Proteasome subunit beta type-7 OS=Mus musculus OX=10090 GN=Psmb7 PE=1 SV=1	10.011	15.16245	16.35	3
Q9QZ23	NFU1 iron-sulfur cluster scaffold homolog, mitochondrial OS=Mus musculus OX=10090 GN=Nfu1 PE=1 SV=2	10.002	15.29412	11.42	3
Q3V038	Tetratricopeptide repeat protein 9A OS=Mus musculus OX=10090 GN=Ttc9 PE=1 SV=1	9.993	10.04566	7.37	2
Q9WTM5	RuvB-like 2 OS=Mus musculus OX=10090 GN=Ruvbl2 PE=1 SV=3	9.99	8.207343	7.51	3
P54823	Probable ATP-dependent RNA helicase DDX6 OS=Mus musculus OX=10090 GN=Ddx6 PE=1 SV=1	9.925	8.281573	11.17	2
P42125	Enoyl-CoA delta isomerase 1, mitochondrial OS=Mus musculus OX=10090 GN=Eci1 PE=1 SV=2	9.922	11.41869	7.86	2
P56379	ATP synthase subunit ATP5MPL, mitochondrial OS=Mus musculus OX=10090 GN=Atp5mpl PE=1 SV=1	9.903	34.48276	13.08	3
Q8BH58	TIP41-like protein OS=Mus musculus OX=10090 GN=Tiprl PE=1 SV=1	9.902	7.380074	7.93	2
P28667	MARCKS-related protein OS=Mus musculus OX=10090 GN=Marcksl1 PE=1 SV=2	9.899	11	5.11	2
P31230	Aminoacyl tRNA synthase complex-interacting multifunctional protein 1 OS=Mus musculus OX=10090 GN=Aim	9.869	9.354839	7.77	2
P47802	Metaxin-1 OS=Mus musculus OX=10090 GN=Mtx1 PE=1 SV=1	9.839	9.463722	7.29	2
P61957	Small ubiquitin-related modifier 2 OS=Mus musculus OX=10090 GN=Sumo2 PE=1 SV=1	9.81	23.15789	12.13	2
Q8K341	Alpha-tubulin N-acetyltransferase 1 OS=Mus musculus OX=10090 GN=Atat1 PE=1 SV=1	9.804	7.83848	5.86	3
O35286	Pre-mRNA-splicing factor ATP-dependent RNA helicase DHX15 OS=Mus musculus OX=10090 GN=Dhx15 PE=1 SV	9.793	2.767296	6.89	2
P03911	NADH-ubiquinone oxidoreductase chain 4 OS=Mus musculus OX=10090 GN=Mtnd4 PE=1 SV=1	9.79	6.535948	16.78	2
Q8R050	Eukaryotic peptide chain release factor GTP-binding subunit ERF3A OS=Mus musculus OX=10090 GN=Gspt1 PE=	9.786	4.402516	9.11	3
P17563	Methanethiol oxidase OS=Mus musculus OX=10090 GN=Selenbp1 PE=1 SV=2	9.783	6.991525	7.98	2
Q9WUR2	Enoyl-CoA delta isomerase 2, mitochondrial OS=Mus musculus OX=10090 GN=Eci2 PE=1 SV=2	9.773	6.649616	6.31	2
Q8BNN1	Spermatogenesis-associated protein 2-like protein OS=Mus musculus OX=10090 GN=Spata2l PE=1 SV=1	9.768	5.868545	5.87	1
P63137	Gamma-aminobutyric acid receptor subunit beta-2 OS=Mus musculus OX=10090 GN=Gabrb2 PE=1 SV=2	9.738	4.882813	8.45	2
O35887	Calumenin OS=Mus musculus OX=10090 GN=Calu PE=1 SV=1	9.731	8.571429	9.21	2
P62743	AP-2 complex subunit sigma OS=Mus musculus OX=10090 GN=Ap2s1 PE=1 SV=1	9.691	30.98592	16.03	5
P47964	60S ribosomal protein L36 OS=Mus musculus OX=10090 GN=Rpl36 PE=1 SV=2	9.678	18.09524	14.39	2
Q9WUM3	Coronin-1B OS=Mus musculus OX=10090 GN=Coro1b PE=1 SV=1	9.652	8.471074	8.96	5
P59017	Bcl-2-like protein 13 OS=Mus musculus OX=10090 GN=Bcl2l13 PE=1 SV=2	9.64	8.294931	8.11	2
Q05BC3	Echinoderm microtubule-associated protein-like 1 OS=Mus musculus OX=10090 GN=Eml1 PE=1 SV=1	9.591	2.825553	6.96	2
Q9WV18	Gamma-aminobutyric acid type B receptor subunit 1 OS=Mus musculus OX=10090 GN=Gabbr1 PE=1 SV=1	9.587	2.1875	6.01	2
P61961	Ubiquitin-fold modifier 1 OS=Mus musculus OX=10090 GN=Ufm1 PE=1 SV=1	9.537	50.58824	14.59	2
P60867	40S ribosomal protein S20 OS=Mus musculus OX=10090 GN=Rps20 PE=1 SV=1	9.501	16.80672	11.98	3
Q6P2B1	Transportin-3 OS=Mus musculus OX=10090 GN=Tnpo3 PE=1 SV=1	9.488	3.033586	6.48	2
P03888	NADH-ubiquinone oxidoreductase chain 1 OS=Mus musculus OX=10090 GN=Mtnd1 PE=1 SV=3	9.473	5.974843	6.89	1
Q8VE47	Ubiquitin-like modifier-activating enzyme 5 OS=Mus musculus OX=10090 GN=Uba5 PE=1 SV=2	9.457	5.955335	4.26	1
Q8BH04	Phosphoenolpyruvate carboxykinase [GTP], mitochondrial OS=Mus musculus OX=10090 GN=Pck2 PE=1 SV=1	9.447	4.84375	6.75	2
P09602	Non-histone chromosomal protein HMG-17 OS=Mus musculus OX=10090 GN=Hmgn2 PE=1 SV=2	9.445	31.11111	11.09	5
O08663	Methionine aminopeptidase 2 OS=Mus musculus OX=10090 GN=Metap2 PE=1 SV=1	9.437	4.60251	8.94	1
Q8BTV2	Cleavage and polyadenylation specificity factor subunit 7 OS=Mus musculus OX=10090 GN=Cpsf7 PE=1 SV=2	9.434	5.307856	4.65	1
Q9Z1J3	Cysteine desulfurase, mitochondrial OS=Mus musculus OX=10090 GN=Nfs1 PE=1 SV=3	9.409	7.843137	8.87	3
P60122	RuvB-like 1 OS=Mus musculus OX=10090 GN=Ruvbl1 PE=1 SV=1	9.407	5.701754	8.12	2
Q9JII5	DAZ-associated protein 1 OS=Mus musculus OX=10090 GN=Dazap1 PE=1 SV=2	9.371	7.881773	9.7	2
Q56A07	Sodium channel subunit beta-2 OS=Mus musculus OX=10090 GN=Scn2b PE=1 SV=1	9.348	9.767442	5.7	2
Q59J78	NADH dehydrogenase [ubiquinone] 1 alpha subcomplex assembly factor 2 OS=Mus musculus OX=10090 GN=Nd	9.333	13.69048	10.05	2
P61087	Ubiquitin-conjugating enzyme E2 K OS=Mus musculus OX=10090 GN=Ube2k PE=1 SV=3	9.297	10.5	6.49	2
Q7M750	Opalin OS=Mus musculus OX=10090 GN=Opalin PE=1 SV=1	9.282	14.68531	8.69	1
Q8JZQ9	Eukaryotic translation initiation factor 3 subunit B OS=Mus musculus OX=10090 GN=Eif3b PE=1 SV=1	9.256	4.358655	6.77	3
Q9CR67	Transmembrane protein 33 OS=Mus musculus OX=10090 GN=Tmem33 PE=1 SV=1	9.244	7.287449	10.65	2
Q9Z0Y1	Dynactin subunit 3 OS=Mus musculus OX=10090 GN=Dctn3 PE=1 SV=2	9.223	16.12903	7.02	3
P28798	Progranulin OS=Mus musculus OX=10090 GN=Grn PE=1 SV=2	9.22	4.923599	6.77	2
P26039	Talin-1 OS=Mus musculus OX=10090 GN=Tln1 PE=1 SV=2	9.216	1.338056	8.74	3
Q60854	Serpin B6 OS=Mus musculus OX=10090 GN=Serpinb6 PE=1 SV=1	9.216	7.936508	6.78	2
P29699	Alpha-2-HS-glycoprotein OS=Mus musculus OX=10090 GN=Ahsg PE=1 SV=1	9.216	13.33333	11.86	2
Q8R574	Phosphoribosyl pyrophosphate synthase-associated protein 2 OS=Mus musculus OX=10090 GN=Prpsap2 PE=1 S	9.215	5.96206	5.56	1
Q9CQX2	Cytochrome b5 type B OS=Mus musculus OX=10090 GN=Cyb5b PE=1 SV=1	9.213	28.76712	5.69	2
Q9QZ73	DCN1-like protein 1 OS=Mus musculus OX=10090 GN=Dcun1d1 PE=2 SV=1	9.199	10.03861	5.85	2
Q9D2N4	Dystrobrevin alpha OS=Mus musculus OX=10090 GN=Dtna PE=1 SV=2	9.099	2.546917	9.81	1
P97441	Zinc transporter 3 OS=Mus musculus OX=10090 GN=Slc30a3 PE=1 SV=1	9.089	6.958763	8.96	2
P09813	Apolipoprotein A-II OS=Mus musculus OX=10090 GN=Apoa2 PE=1 SV=2	9.082	18.62745	9.82	2
Q9R257	Heme-binding protein 1 OS=Mus musculus OX=10090 GN=Hebp1 PE=1 SV=2	9.065	8.421053	6.8	1
Q9R226	KH domain-containing, RNA-binding, signal transduction-associated protein 3 OS=Mus musculus OX=10090 GN	9.05	6.647399	8.85	3
Q8R1I1	Cytochrome b-c1 complex subunit 9 OS=Mus musculus OX=10090 GN=Uqcr10 PE=1 SV=1	9.043	37.5	9.9	2
Q9D1L0	Coiled-coil-helix-coiled-coil-helix domain-containing protein 2 OS=Mus musculus OX=10090 GN=Chchd2 PE=2	9.04	15.68627	5.54	1
Q9D358	Low molecular weight phosphotyrosine protein phosphatase OS=Mus musculus OX=10090 GN=Acp1 PE=1 SV=3	9.027	17.08861	7.13	3
P56135	ATP synthase subunit f, mitochondrial OS=Mus musculus OX=10090 GN=Atp5mf PE=1 SV=3	9.018	13.63636	13.65	1
Q9D1M0	Protein SEC13 homolog OS=Mus musculus OX=10090 GN=Sec13 PE=1 SV=3	8.993	10.55901	8.59	2
Q9D710	Thioredoxin-related transmembrane protein 2 OS=Mus musculus OX=10090 GN=Tmx2 PE=1 SV=1	8.982	4.067797	11.26	1
Q60875	Rho guanine nucleotide exchange factor 2 OS=Mus musculus OX=10090 GN=Arhgef2 PE=1 SV=4	8.962	3.045685	13.37	2
Q8R010	Aminoacyl tRNA synthase complex-interacting multifunctional protein 2 OS=Mus musculus OX=10090 GN=Aim	8.956	7.5	6.35	2
P47941	Crk-like protein OS=Mus musculus OX=10090 GN=Crkl PE=1 SV=2	8.946	11.22112	10.93	2
Q62426	Cystatin-B OS=Mus musculus OX=10090 GN=Cstb PE=1 SV=1	8.941	21.42857	4.53	1
Q9CPW4	Actin-related protein 2/3 complex subunit 5 OS=Mus musculus OX=10090 GN=Arpc5 PE=1 SV=3	8.926	21.8543	15.06	3
Q8R1A4	Dedicator of cytokinesis protein 7 OS=Mus musculus OX=10090 GN=Dock7 PE=1 SV=3	8.916	0.892019	4.44	1
Q60870	Receptor expression-enhancing protein 5 OS=Mus musculus OX=10090 GN=Reep5 PE=1 SV=1	8.911	10.27027	9.13	2
Q8CI71	Syndetin OS=Mus musculus OX=10090 GN=Vps50 PE=1 SV=2	8.875	1.86722	4.38	1
Q505D7	Optic atrophy 3 protein homolog OS=Mus musculus OX=10090 GN=Opa3 PE=1 SV=1	8.871	10.61453	9.89	2
P53986	Monocarboxylate transporter 1 OS=Mus musculus OX=10090 GN=Slc16a1 PE=1 SV=1	8.853	7.707911	6.34	2
Q9D1F4	Proline-rich AKT1 substrate 1 OS=Mus musculus OX=10090 GN=Akt1s1 PE=1 SV=1	8.851	4.669261	6.9	1
Q8R016	Bleomycin hydrolase OS=Mus musculus OX=10090 GN=Blmh PE=1 SV=1	8.77	7.912088	12.27	3
P62245	40S ribosomal protein S15a OS=Mus musculus OX=10090 GN=Rps15a PE=1 SV=2	8.763	17.69231	7.62	3
O08997	Copper transport protein ATOX1 OS=Mus musculus OX=10090 GN=Atox1 PE=1 SV=1	8.763	32.35294	16.1	2
Q99JB2	Stomatin-like protein 2, mitochondrial OS=Mus musculus OX=10090 GN=Stoml2 PE=1 SV=1	8.761	7.932011	8.03	2
P24529	Tyrosine 3-monooxygenase OS=Mus musculus OX=10090 GN=Th PE=1 SV=3	8.741	7.228916	6.55	2
Q64674	Spermidine synthase OS=Mus musculus OX=10090 GN=Srm PE=1 SV=1	8.725	8.940397	5.54	2
P41216	Long-chain-fatty-acid--CoA ligase 1 OS=Mus musculus OX=10090 GN=Acsl1 PE=1 SV=2	8.711	3.862661	6.94	2
Q8C6E0	Cilia- and flagella-associated protein 36 OS=Mus musculus OX=10090 GN=Cfap36 PE=1 SV=1	8.698	9.912536	7.25	2
P56565	Protein S100-A1 OS=Mus musculus OX=10090 GN=S100a1 PE=1 SV=2	8.658	23.40426	24.84	2
Q8BVI5	Syntaxin-16 OS=Mus musculus OX=10090 GN=Stx16 PE=1 SV=3	8.657	5.521472	4.95	1
Q7TNG5	Echinoderm microtubule-associated protein-like 2 OS=Mus musculus OX=10090 GN=Eml2 PE=1 SV=1	8.592	3.697997	5.93	2
Q6P4T2	U5 small nuclear ribonucleoprotein 200 kDa helicase OS=Mus musculus OX=10090 GN=Snrnp200 PE=1 SV=1	8.562	1.685393	10.41	2
Q9CQC6	Basic leucine zipper and W2 domain-containing protein 1 OS=Mus musculus OX=10090 GN=Bzw1 PE=1 SV=1	8.548	8.591885	7.11	2
Q9DCS2	Methyltransferase-like 26 OS=Mus musculus OX=10090 GN=Mettl26 PE=1 SV=1	8.546	13.72549	10.9	2
Q7M6Y3	Phosphatidylinositol-binding clathrin assembly protein OS=Mus musculus OX=10090 GN=Picalm PE=1 SV=1	8.539	5.606061	7.31	2
E9Q8I9	Protein furry homolog OS=Mus musculus OX=10090 GN=Fry PE=1 SV=1	8.524	0.860927	4.47	1
Q9CQE5	Regulator of G-protein signaling 10 OS=Mus musculus OX=10090 GN=Rgs10 PE=1 SV=1	8.507	16.0221	7.23	2
Q8BGS2	BolA-like protein 2 OS=Mus musculus OX=10090 GN=Bola2 PE=1 SV=1	8.481	22.09302	11.4	1
Q6P5E4	UDP-glucose:glycoprotein glucosyltransferase 1 OS=Mus musculus OX=10090 GN=Uggt1 PE=1 SV=4	8.481	3.094778	4.2	3
Q80X50	Ubiquitin-associated protein 2-like OS=Mus musculus OX=10090 GN=Ubap2l PE=1 SV=1	8.479	1.806685	5.54	1
Q60996	Serine/threonine-protein phosphatase 2A 56 kDa regulatory subunit gamma isoform OS=Mus musculus OX=10(	8.476	7.061069	8.85	3
Q8BJH1	Zinc finger C2HC domain-containing protein 1A OS=Mus musculus OX=10090 GN=Zc2hc1a PE=1 SV=1	8.458	6.790123	7.03	2
Q3TDK6	Protein rogdi homolog OS=Mus musculus OX=10090 GN=Rogdi PE=1 SV=2	8.438	8.362369	7.84	2
P35235	Tyrosine-protein phosphatase non-receptor type 11 OS=Mus musculus OX=10090 GN=Ptpn11 PE=1 SV=2	8.433	5.025126	11.58	3
Q80WQ2	Protein VAC14 homolog OS=Mus musculus OX=10090 GN=Vac14 PE=1 SV=1	8.408	3.580563	6.95	2
Q05A62	Dynein light chain 1, axonemal OS=Mus musculus OX=10090 GN=Dnal1 PE=1 SV=2	8.402	10.52632	3.71	1
O35660	Glutathione S-transferase Mu 6 OS=Mus musculus OX=10090 GN=Gstm6 PE=1 SV=4	8.39	11.00917	11.2	3
Q9D0R2	Threonine--tRNA ligase, cytoplasmic OS=Mus musculus OX=10090 GN=Tars PE=1 SV=2	8.385	4.709141	8.11	3
O08532	Voltage-dependent calcium channel subunit alpha-2/delta-1 OS=Mus musculus OX=10090 GN=Cacna2d1 PE=1	8.381	2.357208	9.06	2
P97493	Thioredoxin, mitochondrial OS=Mus musculus OX=10090 GN=Txn2 PE=1 SV=1	8.355	9.036145	18.81	1
P35438	Glutamate receptor ionotropic, NMDA 1 OS=Mus musculus OX=10090 GN=Grin1 PE=1 SV=1	8.354	2.025586	5.92	2
O09131	Glutathione S-transferase omega-1 OS=Mus musculus OX=10090 GN=Gsto1 PE=1 SV=2	8.332	9.166667	6.78	2
O09172	Glutamate--cysteine ligase regulatory subunit OS=Mus musculus OX=10090 GN=Gclm PE=1 SV=1	8.3	5.839416	8.93	1
Q99KP6	Pre-mRNA-processing factor 19 OS=Mus musculus OX=10090 GN=Prpf19 PE=1 SV=1	8.29	5.555556	9.3	2
Q9DCS3	Enoyl-[acyl-carrier-protein] reductase, mitochondrial OS=Mus musculus OX=10090 GN=Mecr PE=1 SV=2	8.259	10.72386	5.68	1
Q9DCB4	cAMP-regulated phosphoprotein 21 OS=Mus musculus OX=10090 GN=Arpp21 PE=1 SV=2	8.246	2.354399	5.72	2
P56383	ATP synthase F(0) complex subunit C2, mitochondrial OS=Mus musculus OX=10090 GN=Atp5mc2 PE=1 SV=2	8.236	26.0274	17.15	2
Q99MN1	Lysine--tRNA ligase OS=Mus musculus OX=10090 GN=Kars PE=1 SV=1	8.22	7.563025	6.34	3
Q6QWF9	Calcium/calmodulin-dependent protein kinase II inhibitor 1 OS=Mus musculus OX=10090 GN=Camk2n1 PE=1 S	8.209	34.61538	6.61	2
Q9QZE7	Translin-associated protein X OS=Mus musculus OX=10090 GN=Tsnax PE=1 SV=1	8.123	6.551724	5.15	1
Q99JY8	Phospholipid phosphatase 3 OS=Mus musculus OX=10090 GN=Plpp3 PE=1 SV=1	8.122	8.974359	12.72	3
Q9D1T0	Leucine-rich repeat and immunoglobulin-like domain-containing nogo receptor-interacting protein 1 OS=Mus	8.119	7.32899	7.13	3
O35609	Secretory carrier-associated membrane protein 3 OS=Mus musculus OX=10090 GN=Scamp3 PE=1 SV=3	8.114	4.584527	8.36	1
Q9CPX6	Ubiquitin-like-conjugating enzyme ATG3 OS=Mus musculus OX=10090 GN=Atg3 PE=1 SV=1	8.107	8.917197	7.54	2
P62852	40S ribosomal protein S25 OS=Mus musculus OX=10090 GN=Rps25 PE=1 SV=1	8.083	14.4	7.85	2
Q9JMF3	Guanine nucleotide-binding protein G(I)/G(S)/G(O) subunit gamma-13 OS=Mus musculus OX=10090 GN=Gng13	8.038	14.92537	7.2	1
Q9JKY5	Huntingtin-interacting protein 1-related protein OS=Mus musculus OX=10090 GN=Hip1r PE=1 SV=2	8.038	2.434457	6.84	2
O88910	MAGUK p55 subfamily member 3 OS=Mus musculus OX=10090 GN=Mpp3 PE=1 SV=2	8.006	7.570423	8.39	3
P48193	Protein 4.1 OS=Mus musculus OX=10090 GN=Epb41 PE=1 SV=2	8.003	2.913753	4.64	2
O55100	Synaptogyrin-1 OS=Mus musculus OX=10090 GN=Syngr1 PE=1 SV=2	7.983	5.128205	7.61	1
Q9QWY8	Arf-GAP with SH3 domain, ANK repeat and PH domain-containing protein 1 OS=Mus musculus OX=10090 GN=A	7.972	2.005231	5.05	2
Q9CR09	Ubiquitin-fold modifier-conjugating enzyme 1 OS=Mus musculus OX=10090 GN=Ufc1 PE=1 SV=1	7.954	13.77246	6.51	2
Q922P9	Putative oxidoreductase GLYR1 OS=Mus musculus OX=10090 GN=Glyr1 PE=1 SV=1	7.927	4.395604	4.76	2
Q8R4E6	Purine-rich element-binding protein gamma OS=Mus musculus OX=10090 GN=Purg PE=1 SV=1	7.893	7.142857	6.85	2
O35344	Importin subunit alpha-4 OS=Mus musculus OX=10090 GN=Kpna3 PE=1 SV=1	7.891	7.293666	3.55	2
P03899	NADH-ubiquinone oxidoreductase chain 3 OS=Mus musculus OX=10090 GN=Mtnd3 PE=1 SV=3	7.869	13.04348	2.89	1
Q9Z0N1	Eukaryotic translation initiation factor 2 subunit 3, X-linked OS=Mus musculus OX=10090 GN=Eif2s3x PE=1 SV=	7.858	5.932203	5.45	1
Q8BWM0	Prostaglandin E synthase 2 OS=Mus musculus OX=10090 GN=Ptges2 PE=1 SV=3	7.858	7.03125	5.91	1
P61202	COP9 signalosome complex subunit 2 OS=Mus musculus OX=10090 GN=Cops2 PE=1 SV=1	7.833	5.643341	9.51	2
Q8BTG7	Protein NDRG4 OS=Mus musculus OX=10090 GN=Ndrg4 PE=1 SV=1	7.792	11.64773	8.33	3
Q9WVL0	Maleylacetoacetate isomerase OS=Mus musculus OX=10090 GN=Gstz1 PE=1 SV=1	7.791	9.259259	5.09	1
P33173	Kinesin-like protein KIF1A OS=Mus musculus OX=10090 GN=Kif1a PE=1 SV=2	7.775	1.887906	10.72	3
Q62059	Versican core protein OS=Mus musculus OX=10090 GN=Vcan PE=1 SV=2	7.746	0.685136	4.39	2
P28650	Adenylosuccinate synthetase isozyme 1 OS=Mus musculus OX=10090 GN=Adssl1 PE=1 SV=2	7.731	5.251641	8.36	2
Q8JZR6	Electroneutral sodium bicarbonate exchanger 1 OS=Mus musculus OX=10090 GN=Slc4a8 PE=2 SV=1	7.724	2.112029	14.95	3
P62849	40S ribosomal protein S24 OS=Mus musculus OX=10090 GN=Rps24 PE=1 SV=1	7.717	16.54135	12.9	2
Q9D8B4	NADH dehydrogenase [ubiquinone] 1 alpha subcomplex subunit 11 OS=Mus musculus OX=10090 GN=Ndufa11	7.714	7.801418	9.9	1
Q99KK2	N-acylneuraminate cytidylyltransferase OS=Mus musculus OX=10090 GN=Cmas PE=1 SV=2	7.706	4.861111	7.55	2
Q9QYS2	Metabotropic glutamate receptor 3 OS=Mus musculus OX=10090 GN=Grm3 PE=1 SV=1	7.704	2.957907	14.46	2
Q9WVK8	Cholesterol 24-hydroxylase OS=Mus musculus OX=10090 GN=Cyp46a1 PE=1 SV=1	7.701	2.2	6.88	1
Q9D7N9	Adipocyte plasma membrane-associated protein OS=Mus musculus OX=10090 GN=Apmap PE=1 SV=1	7.692	4.337349	6.65	1
Q6ZWR6	Nesprin-1 OS=Mus musculus OX=10090 GN=Syne1 PE=1 SV=2	7.653	0.545517	3.43	2
Q9CQW1	Synaptobrevin homolog YKT6 OS=Mus musculus OX=10090 GN=Ykt6 PE=1 SV=1	7.641	8.585859	10.24	2
Q9CQS8	Protein transport protein Sec61 subunit beta OS=Mus musculus OX=10090 GN=Sec61b PE=1 SV=3	7.613	21.875	9.59	2
Q8BZJ7	DCN1-like protein 2 OS=Mus musculus OX=10090 GN=Dcun1d2 PE=1 SV=3	7.602	8.880309	3.65	2
P26369	Splicing factor U2AF 65 kDa subunit OS=Mus musculus OX=10090 GN=U2af2 PE=1 SV=3	7.564	3.789474	5.55	1
Q791T5	Mitochondrial carrier homolog 1 OS=Mus musculus OX=10090 GN=Mtch1 PE=1 SV=1	7.552	3.341902	2.86	1
Q9CR26	Vacuolar protein sorting-associated protein VTA1 homolog OS=Mus musculus OX=10090 GN=Vta1 PE=1 SV=1	7.539	6.472492	9.87	1
Q99JP7	Glutathione hydrolase 7 OS=Mus musculus OX=10090 GN=Ggt7 PE=1 SV=2	7.528	2.567976	4.13	1
Q8BFU3	RING finger protein 214 OS=Mus musculus OX=10090 GN=Rnf214 PE=1 SV=1	7.511	3.592814	6.61	2
Q9CXI5	Mesencephalic astrocyte-derived neurotrophic factor OS=Mus musculus OX=10090 GN=Manf PE=1 SV=1	7.503	15.0838	9.6	2
Q8JZK9	Hydroxymethylglutaryl-CoA synthase, cytoplasmic OS=Mus musculus OX=10090 GN=Hmgcs1 PE=1 SV=1	7.488	5	7.43	2
P56380	Bis(5'-nucleosyl)-tetraphosphatase [asymmetrical] OS=Mus musculus OX=10090 GN=Nudt2 PE=1 SV=3	7.473	9.52381	3.12	1

P60229	Eukaryotic translation initiation factor 3 subunit E OS=Mus musculus OX=10090 GN=Eif3e PE=1 SV=1	7.29	2.47191	5.86	1	2	1	52.2	1.224	1.089	1.238
Q9D289	Trafficking protein particle complex subunit 6B OS=Mus musculus OX=10090 GN=Trappc6b PE=1 SV=1	7.287	8.860759	6.38	1	2	1	17.9	1.336	1.048	1.249
P58044	Isopentenyl-diphosphate Delta-isomerase 1 OS=Mus musculus OX=10090 GN=Idi1 PE=1 SV=1	7.284	9.251101	5.97	2	2	2	26.3	1.347	1.065	1.349
Q9CXJ4	ATP-binding cassette sub-family B member 8, mitochondrial OS=Mus musculus OX=10090 GN=Abcb8 PE=1 SV=1	7.278	4.46304	8.22	2	3	2	78	1.357	1.411	1.537
Q99LT0	Protein dpy-30 homolog OS=Mus musculus OX=10090 GN=Dpy30 PE=1 SV=1	7.272	20.20202	8.3	1	2	1	11.2	1.437	0.941	1.261
Q9CY57	Chromatin target of PRMT1 protein OS=Mus musculus OX=10090 GN=Chtop PE=1 SV=2	7.262	5.220884	3.55	1	1	1	26.6	1.411	1.121	1.358
Q9DC61	Mitochondrial-processing peptidase subunit alpha OS=Mus musculus OX=10090 GN=Pmpca PE=1 SV=1	7.262	8.969466	3.31	3	3	3	58.2	1.106	1.043	1.098
Q8JZP2	Synapsin-3 OS=Mus musculus OX=10090 GN=Syn3 PE=1 SV=2	7.254	5.872193	7.42	2	3	2	63.3	1.283	1.139	1.332
Q8VE62	Polyadenylate-binding protein-interacting protein 1 OS=Mus musculus OX=10090 GN=Paip1 PE=1 SV=1	7.247	5	4.72	1	1	1	45.7	1.268	1.219	1.285
P12815	Programmed cell death protein 6 OS=Mus musculus OX=10090 GN=Pdcd6 PE=1 SV=2	7.196	15.18325	7.82	3	3	3	21.9	1.213	1.102	1.213
Q8BY89	Choline transporter-like protein 2 OS=Mus musculus OX=10090 GN=Slc44a2 PE=1 SV=2	7.192	2.266289	9.99	1	2	1	80.1	1.19	1.064	1.276
P03930	ATP synthase protein 8 OS=Mus musculus OX=10090 GN=Mtatp8 PE=1 SV=1	7.18	31.34328	8.81	2	3	2	7.8	1.167	1.096	1.326
Q9DCP2	Sodium-coupled neutral amino acid transporter 3 OS=Mus musculus OX=10090 GN=Slc38a3 PE=1 SV=1	7.175	3.168317	5.46	1	2	1	55.6	1.283	1.04	1.303
Q920E5	Farnesyl pyrophosphate synthase OS=Mus musculus OX=10090 GN=Fdps PE=1 SV=1	7.17	10.1983	12.25	3	5	3	40.6	1.313	1.124	1.386
Q9WTR5	Cadherin-13 OS=Mus musculus OX=10090 GN=Cdh13 PE=1 SV=2	7.164	5.882353	7.62	3	3	3	78.1	1.165	0.974	1.167
O89023	Tripeptidyl-peptidase 1 OS=Mus musculus OX=10090 GN=Tpp1 PE=1 SV=2	7.162	3.736655	4.5	1	1	1	61.3			
Q9EPN1	Neurobeachin OS=Mus musculus OX=10090 GN=Nbea PE=1 SV=1	7.154	0.749319	10.23	2	3	2	326.5	1.018	1.042	1.136
P51655	Glypican-4 OS=Mus musculus OX=10090 GN=Gpc4 PE=1 SV=2	7.136	4.48833	5.39	2	3	2	62.5	1.292	1.041	1.313
A2ASZ8	Calcium-binding mitochondrial carrier protein SCaMC-2 OS=Mus musculus OX=10090 GN=Slc25a25 PE=1 SV=1	7.134	7.462687	9.95	2	3	1	52.6	1.033	0.959	1.15
Q8BL97	Serine/arginine-rich splicing factor 7 OS=Mus musculus OX=10090 GN=Srsf7 PE=1 SV=1	7.124	4.494382	3.87	1	1	1	30.8			
O35593	26S proteasome non-ATPase regulatory subunit 14 OS=Mus musculus OX=10090 GN=Psmd14 PE=1 SV=2	7.077	10.64516	10.11	2	4	2	34.6	0.7	0.453	0.545
Q9CQZ1	Heat shock factor-binding protein 1 OS=Mus musculus OX=10090 GN=Hsbp1 PE=1 SV=1	7.075	32.89474	6.26	1	1	1	8.6	1.356	1.205	1.526
O88587	Catechol O-methyltransferase OS=Mus musculus OX=10090 GN=Comt PE=1 SV=2	7.072	11.69811	8.02	2	2	2	29.5			
Q9EQI8	39S ribosomal protein L46, mitochondrial OS=Mus musculus OX=10090 GN=Mrpl46 PE=1 SV=1	7.049	7.067138	9	1	2	1	32.1			
P05480	Neuronal proto-oncogene tyrosine-protein kinase Src OS=Mus musculus OX=10090 GN=Src PE=1 SV=4	7.046	7.763401	10.21	3	4	2	60.6	1.233	0.969	1.173
Q9CQI7	U2 small nuclear ribonucleoprotein B'' OS=Mus musculus OX=10090 GN=Snrpb2 PE=1 SV=1	7.042	8.444444	5.51	2	2	1	25.3			
Q9ES28	Rho guanine nucleotide exchange factor 7 OS=Mus musculus OX=10090 GN=Arhgef7 PE=1 SV=2	7.024	2.552204	4.76	2	2	2	97	1.264	1.12	1.269
Q9CQ91	NADH dehydrogenase [ubiquinone] 1 alpha subcomplex subunit 3 OS=Mus musculus OX=10090 GN=Ndufa3 PE	7.018	28.57143	12.98	3	5	3	9.3	1.344	1.25	1.314
P59648	FXYD domain-containing ion transport regulator 7 OS=Mus musculus OX=10090 GN=Fxyd7 PE=3 SV=1	7.017	28.75	7.05	2	3	2	8.5	1.065	0.903	1.113
Q8BWG8	Beta-arrestin-1 OS=Mus musculus OX=10090 GN=Arrb1 PE=1 SV=1	6.989	8.851675	7.75	2	3	2	46.9			
P47740	Aldehyde dehydrogenase family 3 member A2 OS=Mus musculus OX=10090 GN=Aldh3a2 PE=1 SV=2	6.961	2.892562	4.6	1	1	1	53.9			
Q8BG73	SH3 domain-binding glutamic acid-rich-like protein 2 OS=Mus musculus OX=10090 GN=Sh3bgrl2 PE=1 SV=1	6.937	11.21495	6.9	1	2	1	12.2			
P0C0A3	Charged multivesicular body protein 6 OS=Mus musculus OX=10090 GN=Chmp6 PE=1 SV=2	6.937	7	6.3	1	2	1	23.4	1.169	1.079	1.327
Q9DBL1	Short/branched chain specific acyl-CoA dehydrogenase, mitochondrial OS=Mus musculus OX=10090 GN=Acads	6.934	9.259259	6.26	2	3	2	47.8	0.975	0.843	1.152
Q99KY4	Cyclin-G-associated kinase OS=Mus musculus OX=10090 GN=Gak PE=1 SV=2	6.928	1.685824	7.2	2	3	1	143.6			
D3YZU1	SH3 and multiple ankyrin repeat domains protein 1 OS=Mus musculus OX=10090 GN=Shank1 PE=1 SV=1	6.926	0.646054	3.74	1	1	1	226.2	1.196	1.03	1.204
Q9D554	Splicing factor 3A subunit 3 OS=Mus musculus OX=10090 GN=Sf3a3 PE=1 SV=2	6.873	2.39521	3.58	1	1	1	58.8			
Q8BGX2	Mitochondrial import inner membrane translocase subunit Tim29 OS=Mus musculus OX=10090 GN=Timm29 P	6.868	5.263158	6.84	1	2	1	29.4	1.228	1.053	1.206
Q8BRF7	Sec1 family domain-containing protein 1 OS=Mus musculus OX=10090 GN=Scfd1 PE=1 SV=1	6.854	2.660407	9.76	1	2	1	72.3	1.348	1.156	1.32
P03921	NADH-ubiquinone oxidoreductase chain 5 OS=Mus musculus OX=10090 GN=Mtnd5 PE=1 SV=3	6.837	4.448105	5.98	2	2	2	68.4			
Q921H8	3-ketoacyl-CoA thiolase A, peroxisomal OS=Mus musculus OX=10090 GN=Acaa1a PE=1 SV=1	6.835	8.018868	5.21	2	2	2	43.9	1.26	0.995	0.985
Q8BWQ6	VPS35 endosomal protein sorting factor-like OS=Mus musculus OX=10090 GN=Vps35l PE=1 SV=2	6.832	2.803738	5.02	2	2	2	109	1.316	0.993	1.29
P60670	Nuclear protein localization protein 4 homolog OS=Mus musculus OX=10090 GN=Nploc4 PE=1 SV=3	6.823	5.263158	3.37	2	3	2	68	1.054	0.881	1.188
Q3TIR3	Synembryn-A OS=Mus musculus OX=10090 GN=Ric8a PE=1 SV=2	6.817	2.830189	5.4	1	2	1	59.8			
Q7TNV0	Protein DEK OS=Mus musculus OX=10090 GN=Dek PE=1 SV=1	6.804	8.157895	9.85	3	4	3	43.1	1.257	1.015	1.12
Q9EQZ6	Rap guanine nucleotide exchange factor 4 OS=Mus musculus OX=10090 GN=Rapgef4 PE=1 SV=1	6.798	2.670623	8.3	2	3	2	115.4			
Q8R3S6	Exocyst complex component 1 OS=Mus musculus OX=10090 GN=Exoc1 PE=1 SV=4	6.796	2.46085	3.57	1	1	1	101.8	1.038	0.66	1.02

Q3V3V9	Capping protein, Arp2/3 and myosin-I linker protein 2 OS=Mus musculus OX=10090 GN=Carmil2 PE=1 SV=2	4.225	0.848765	2.87	1	1	1	141.3				[illegible]
E9PVA8	eIF-2-alpha kinase activator GCN1 OS=Mus musculus OX=10090 GN=Gcn1 PE=1 SV=1	4.215	0.561587	3.49	1	1	1	292.8	1.332	0.961	1.355	[illegible]
Q58A65	C-Jun-amino-terminal kinase-interacting protein 4 OS=Mus musculus OX=10090 GN=Spag9 PE=1 SV=2	4.178	0.832702	2.3	1	1	1	146.1				[illegible]
Q6PEE2	CBP80/20-dependent translation initiation factor OS=Mus musculus OX=10090 GN=Ctif PE=1 SV=2	4.168	3	3.35	1	1	1	67.8				[illegible]
Q80VC9	Calmodulin-regulated spectrin-associated protein 3 OS=Mus musculus OX=10090 GN=Camsap3 PE=1 SV=1	4.159	1.118211	0	1	1	1	135.1				[illegible]
Q64442	Sorbitol dehydrogenase OS=Mus musculus OX=10090 GN=Sord PE=1 SV=3	4.158	5.602241	2.63	2	2	2	38.2	1.06	0.947	1.136	[illegible]
Q6NS82	Reticulophagy regulator 2 OS=Mus musculus OX=10090 GN=Retreg2 PE=1 SV=2	4.128	2.033272	2.79	1	1	1	57.5				[illegible]
Q9QZ29	Immunoglobulin-binding protein 1b OS=Mus musculus OX=10090 GN=Igbp1b PE=1 SV=1	4.12	3.498542	2.3	1	1	1	39.2	1.299	1.134	1.239	[illegible]
Q8K274	Ketosamine-3-kinase OS=Mus musculus OX=10090 GN=Fn3krp PE=1 SV=2	4.114	6.148867	8.55	1	2	1	34.4	1.33	1.141	1.435	[illegible]
Q8BW86	Rho guanine nucleotide exchange factor 33 OS=Mus musculus OX=10090 GN=Arhgef33 PE=1 SV=3	4.102	2.117647	4.95	2	2	2	94.7				[illegible]
Q9JJF0	Nucleosome assembly protein 1-like 5 OS=Mus musculus OX=10090 GN=Nap1l5 PE=1 SV=1	4.098	6.410256	3.28	1	1	1	17				[illegible]
P29416	Beta-hexosaminidase subunit alpha OS=Mus musculus OX=10090 GN=Hexa PE=1 SV=2	4.092	2.083333	3.06	1	1	1	60.6				[illegible]
P80560	Receptor-type tyrosine-protein phosphatase N2 OS=Mus musculus OX=10090 GN=Ptprn2 PE=1 SV=2	4.087	1.198801	3.53	1	1	1	111.4	1.294	1.047	1.27	[illegible]
P54763	Ephrin type-B receptor 2 OS=Mus musculus OX=10090 GN=Ephb2 PE=1 SV=3	4.069	1.926978	3.71	1	1	1	109.8	0.96	0.928	1.09	[illegible]
Q8VDL4	ADP-dependent glucokinase OS=Mus musculus OX=10090 GN=Adpgk PE=1 SV=2	4.06	2.620968	3.03	1	1	1	53.9	1.452	1.314	1.175	[illegible]
Q3TY86	Apoptosis-inducing factor 3 OS=Mus musculus OX=10090 GN=Aifm3 PE=1 SV=1	4.06	8.099174	2.24	2	2	2	66.8				[illegible]
P34884	Macrophage migration inhibitory factor OS=Mus musculus OX=10090 GN=Mif PE=1 SV=2	4.045	7.826087	5.5	1	2	1	12.5	1.681	1.005	1.246	[illegible]
Q7TPV4	Myb-binding protein 1A OS=Mus musculus OX=10090 GN=Mybbp1a PE=1 SV=2	4.042	0.967262	2.91	1	1	1	151.9				[illegible]
Q8BXT1	Regulator of G-protein signaling 8 OS=Mus musculus OX=10090 GN=Rgs8 PE=1 SV=1	4.034	6.666667	6.54	1	2	1	21				[illegible]
Q8BX10	Serine/threonine-protein phosphatase PGAM5, mitochondrial OS=Mus musculus OX=10090 GN=Pgam5 PE=1 S	4.028	3.472222	3.19	1	1	1	32	1.23	1.141	1.281	[illegible]
Q62421	Endophilin-A3 OS=Mus musculus OX=10090 GN=Sh3gl3 PE=1 SV=1	4.021	9.510086	1.74	2	2	2	38.9				[illegible]
P22723	Gamma-aminobutyric acid receptor subunit gamma-2 OS=Mus musculus OX=10090 GN=Gabrg2 PE=1 SV=3	4.021	3.375527	7.24	2	5	2	55.1	1.177	1	1.201	[illegible]
Q8BHW2	Protein OSCP1 OS=Mus musculus OX=10090 GN=Oscp1 PE=1 SV=1	4.02	5.277045	4.06	1	1	1	43.2	1.36	1.282	1.256	[illegible]
Q8BR90	UPF0600 protein C5orf51 homolog OS=Mus musculus OX=10090 PE=1 SV=1	4.019	4.761905	3.27	1	1	1	33.5	1.225	1.107	1.283	[illegible]
Q99M87	DnaJ homolog subfamily A member 3, mitochondrial OS=Mus musculus OX=10090 GN=Dnaja3 PE=1 SV=1	4.013	3.333333	2.46	1	1	1	52.4	1.192	0.949	1.209	[illegible]
P24668	Cation-dependent mannose-6-phosphate receptor OS=Mus musculus OX=10090 GN=M6pr PE=1 SV=1	4.008	9.71223	3.1	1	1	1	31.2				[illegible]
Q9R0M4	Podocalyxin OS=Mus musculus OX=10090 GN=Podxl PE=1 SV=2	4.007	5.168986	3.02	1	1	1	53.4				[illegible]
Q8BPM0	Disheveled-associated activator of morphogenesis 1 OS=Mus musculus OX=10090 GN=Daam1 PE=1 SV=4	4.006	1.392758	4.51	1	2	1	123.3				[illegible]
Q8VHC3	Selenoprotein M OS=Mus musculus OX=10090 GN=Selenom PE=1 SV=3	4.006	9.655172	3.4	1	1	1	16.4				[illegible]
O54962	Barrier-to-autointegration factor OS=Mus musculus OX=10090 GN=Banf1 PE=1 SV=1	3.976	8.988764	2.41	1	1	1	10.1				[illegible]
Q9ERE7	LRP chaperone MESD OS=Mus musculus OX=10090 GN=Mesd PE=1 SV=1	3.971	8.928571	8.49	1	2	1	25.2	1.138	0.975	1.182	[illegible]
Q65CL1	Catenin alpha-3 OS=Mus musculus OX=10090 GN=Ctnna3 PE=1 SV=2	3.969	2.905028	2.66	1	1	1	99.7	1.523	1.093	1.442	[illegible]
Q921C1	Gap junction gamma-3 protein OS=Mus musculus OX=10090 GN=Gjc3 PE=1 SV=2	3.957	2.973978	3.13	1	1	1	30.3	1.146	1.018	1.215	[illegible]
Q8BI08	Protein MAL2 OS=Mus musculus OX=10090 GN=Mal2 PE=1 SV=1	3.95	6.285714	6.37	1	2	1	19.1				[illegible]
Q9QZI8	Serine incorporator 1 OS=Mus musculus OX=10090 GN=Serinc1 PE=1 SV=1	3.936	1.986755	2.15	1	2	1	50.5	1.279	1.146	1.169	[illegible]
Q6IR34	G-protein-signaling modulator 1 OS=Mus musculus OX=10090 GN=Gpsm1 PE=1 SV=3	3.934	3.71471	4.46	1	2	1	74.3	0.916	0.853	0.932	[illegible]
Q9DCB1	High mobility group nucleosome-binding domain-containing protein 3 OS=Mus musculus OX=10090 GN=Hmgn	3.925	9.090909	2.95	1	1	1	10.8				[illegible]
P16014	Secretogranin-1 OS=Mus musculus OX=10090 GN=Chgb PE=1 SV=2	3.919	1.772526	3.28	1	1	1	77.9				[illegible]
O35343	Importin subunit alpha-3 OS=Mus musculus OX=10090 GN=Kpna4 PE=1 SV=1	3.916	2.68714	3.92	1	1	1	57.9	1.152	0.99	1.236	[illegible]
P22315	Ferrochelatase, mitochondrial OS=Mus musculus OX=10090 GN=Fech PE=1 SV=2	3.906	7.142857	2.87	2	2	2	47.1				[illegible]
P97352	Protein S100-A13 OS=Mus musculus OX=10090 GN=S100a13 PE=1 SV=1	3.895	12.2449	3.8	1	1	1	11.2	1.253	1.096	1.204	[illegible]
Q9JJI8	60S ribosomal protein L38 OS=Mus musculus OX=10090 GN=Rpl38 PE=1 SV=3	3.888	18.57143	2.97	1	2	1	8.2				[illegible]
Q921Q3	Chitobiosyldiphosphodolichol beta-mannosyltransferase OS=Mus musculus OX=10090 GN=Alg1 PE=1 SV=3	3.88	3.112033	2.79	1	7	1	54.4	1.201	1.291	1.288	[illegible]
P11352	Glutathione peroxidase 1 OS=Mus musculus OX=10090 GN=Gpx1 PE=1 SV=2	3.873	10.44776	5.11	2	3	2	22.3	1.367	1.077	1.35	[illegible]
Q8JZW4	Copine-5 OS=Mus musculus OX=10090 GN=Cpne5 PE=1 SV=1	3.863	4.047218	5.7	2	2	1	65.6				[illegible]
A2A5R2	Brefeldin A-inhibited guanine nucleotide-exchange protein 2 OS=Mus musculus OX=10090 GN=Arfgef2 PE=1 SV	3.861	1.004464	3.27	1	1	1	202.1	1.298	1.017	1.303	[illegible]
P27659	60S ribosomal protein L3 OS=Mus musculus OX=10090 GN=Rpl3 PE=1 SV=3	3.808	3.473945	4.41	2	2	2	46.1				[illegible]
P35290	Ras-related protein Rab-24 OS=Mus musculus OX=10090 GN=Rab24 PE=1 SV=2	3.806	4.433498	2.25	1	1	1	23.1				[illegible]
O09167	60S ribosomal protein L21 OS=Mus musculus OX=10090 GN=Rpl21 PE=1 SV=3	3.804	8.75	4.53	2	2	2	18.6				[illegible]
Q8BTH8	Casein kinase I isoform gamma-1 OS=Mus musculus OX=10090 GN=Csnk1g1 PE=1 SV=2	3.795	1.960784	3.09	1	1	1	52.7				[illegible]
Q9CXS4	Centromere protein V OS=Mus musculus OX=10090 GN=Cenpv PE=1 SV=2	3.771	5.15873	1.71	1	1	1	27.5				[illegible]
Q8K3C3	Protein LZIC OS=Mus musculus OX=10090 GN=Lzic PE=1 SV=1	3.76	12.63158	4.48	2	2	2	21.5				[illegible]
Q99J83	Autophagy protein 5 OS=Mus musculus OX=10090 GN=Atg5 PE=1 SV=1	3.757	6.909091	4.93	1	1	1	32.4	1.185	1.192	1.398	[illegible]
Q3UI43	BRISC and BRCA1-A complex member 1 OS=Mus musculus OX=10090 GN=Babam1 PE=1 SV=1	3.745	5.405405	2.69	1	1	1	36.8				[illegible]
Q8C7R4	Ubiquitin-like modifier-activating enzyme 6 OS=Mus musculus OX=10090 GN=Uba6 PE=1 SV=1	3.728	3.608737	6.11	3	4	3	117.9	0.999	0.954	1.029	[illegible]
Q5HZI2	C2 calcium-dependent domain-containing protein 4C OS=Mus musculus OX=10090 GN=C2cd4cC2CD4 family F	3.719	4.295943	2.15	1	1	1	44.6				[illegible]
Q9D020	Cytosolic 5'-nucleotidase 3A OS=Mus musculus OX=10090 GN=Nt5c3a PE=1 SV=4	3.714	5.740181	2.76	2	2	2	37.2	1.255	0.929	1.256	[illegible]
Q9CXW2	28S ribosomal protein S22, mitochondrial OS=Mus musculus OX=10090 GN=Mrps22 PE=1 SV=1	3.712	3.064067	2.49	1	1	1	41.2				[illegible]
Q5SYD0	Unconventional myosin-Id OS=Mus musculus OX=10090 GN=Myo1d PE=1 SV=1	3.704	1.192843	2.74	1	1	1	116	0.982	1.086	1.198	[illegible]
Q64310	Surfeit locus protein 4 OS=Mus musculus OX=10090 GN=Surf4 PE=1 SV=1	3.702	4.832714	3.38	1	2	1	30.4	2.232	1.296	1.69	[illegible]
Q8VDZ4	Palmitoyltransferase ZDHHC5 OS=Mus musculus OX=10090 GN=Zdhhc5 PE=1 SV=1	3.701	2.237762	1.95	1	1	1	77.5				[illegible]
P12849	cAMP-dependent protein kinase type I-beta regulatory subunit OS=Mus musculus OX=10090 GN=Prkar1b PE=1	3.701	2.624672	3.36	1	1	1	43.2	1.217	1.005	1.241	[illegible]
Q921M3	Splicing factor 3B subunit 3 OS=Mus musculus OX=10090 GN=Sf3b3 PE=1 SV=1	3.695	1.972062	3.22	2	4	2	135.5	1.28	1.1	1.183	[illegible]
Q8VCM8	Nicalin OS=Mus musculus OX=10090 GN=Ncln PE=1 SV=2	3.695	2.486679	3.41	1	1	1	62.9				[illegible]
Q8K411	Presequence protease, mitochondrial OS=Mus musculus OX=10090 GN=Pitrm1 PE=1 SV=1	3.688	1.254826	4.02	1	1	1	117.3				[illegible]
P97855	Ras GTPase-activating protein-binding protein 1 OS=Mus musculus OX=10090 GN=G3bp1 PE=1 SV=1	3.686	3.655914	6.39	1	2	1	51.8				[illegible]
Q64012	RNA-binding protein Raly OS=Mus musculus OX=10090 GN=Raly PE=1 SV=3	3.672	2.884615	2.46	1	1	1	33.2				[illegible]
Q80U95	Ubiquitin-protein ligase E3C OS=Mus musculus OX=10090 GN=Ube3c PE=1 SV=2	3.667	1.569714	3.33	1	1	1	123.9				[illegible]
Q922J3	CAP-Gly domain-containing linker protein 1 OS=Mus musculus OX=10090 GN=Clip1 PE=1 SV=1	3.664	0.790798	3.61	1	1	1	155.7				[illegible]
P62858	40S ribosomal protein S28 OS=Mus musculus OX=10090 GN=Rps28 PE=1 SV=1	3.657	13.04348	4.96	1	2	1	7.8				[illegible]
Q3TY60	Protein FAM131B OS=Mus musculus OX=10090 GN=Fam131b PE=1 SV=1	3.652	3.614458	1.94	1	1	1	35.9				[illegible]
P50171	Estradiol 17-beta-dehydrogenase 8 OS=Mus musculus OX=10090 GN=Hsd17b8 PE=1 SV=2	3.648	5.791506	2.35	1	1	1	26.6				[illegible]
O35405	Phospholipase D3 OS=Mus musculus OX=10090 GN=Pld3 PE=1 SV=1	3.645	2.459016	2.93	1	1	1	54.4				[illegible]
Q8BMI3	ADP-ribosylation factor-binding protein GGA3 OS=Mus musculus OX=10090 GN=Gga3 PE=1 SV=2	3.643	1.253482	3.18	1	1	1	77.9				[illegible]
Q8VBT9	Tether containing UBX domain for GLUT4 OS=Mus musculus OX=10090 GN=Aspscr1 PE=1 SV=1	3.639	2.909091	3.08	1	1	1	59.8				[illegible]
Q8BFY6	Peflin OS=Mus musculus OX=10090 GN=Pef1 PE=1 SV=1	3.628	4.727273	3.1	1	2	1	29.2	1.544	1.296	1.578	[illegible]
Q5U458	DnaJ homolog subfamily C member 11 OS=Mus musculus OX=10090 GN=Dnajc11 PE=1 SV=2	3.626	3.756708	5.04	2	3	2	63.2	1.093	1.047	1.347	[illegible]
Q9JI90	E3 ubiquitin-protein ligase RNF14 OS=Mus musculus OX=10090 GN=Rnf14 PE=1 SV=2	3.624	4.329897	1.68	2	2	2	54.9				[illegible]
G3XA57	Rab11 family-interacting protein 2 OS=Mus musculus OX=10090 GN=Rab11fip2 PE=1 SV=1	3.622	2.539063	2.93	1	1	1	58.2				[illegible]
Q91YI0	Argininosuccinate lyase OS=Mus musculus OX=10090 GN=Asl PE=1 SV=1	3.607	1.939655	2.87	1	1	1	51.7				[illegible]
P55302	Alpha-2-macroglobulin receptor-associated protein OS=Mus musculus OX=10090 GN=Lrpap1 PE=1 SV=1	3.596	3.055556	3.86	1	1	1	42.2	1.606	1.33	1.546	[illegible]
Q6PDI5	Proteasome adapter and scaffold protein ECM29 OS=Mus musculus OX=10090 GN=Ecpas PE=1 SV=3	3.596	0.706522	2.95	1	1	1	203.6				[illegible]
Q811I0	ATP synthase mitochondrial F1 complex assembly factor 1 OS=Mus musculus OX=10090 GN=Atpaf1 PE=1 SV=1	3.594	4.012346	3.2	1	1	1	36.3				[illegible]
Q9DB29	Isoamyl acetate-hydrolyzing esterase 1 homolog OS=Mus musculus OX=10090 GN=Iah1 PE=1 SV=1	3.59	6.024096	3.41	1	1	1	28	1.292	1.033	1.142	[illegible]
Q3V1U8	ELMO domain-containing protein 1 OS=Mus musculus OX=10090 GN=Elmod1 PE=1 SV=2	3.588	9.202454	2.96	2	2	2	38				[illegible]
P56395	Cytochrome b5 OS=Mus musculus OX=10090 GN=Cyb5a PE=1 SV=2	3.567	9.701493	6.49	1	2	1	15.2				[illegible]
O54984	ATPase Asna1 OS=Mus musculus OX=10090 GN=Asna1 PE=1 SV=2	3.567	9.195402	7.77	2	4	2	38.8	1.416	1.34	1.525	[illegible]
Q99J39	Malonyl-CoA decarboxylase, mitochondrial OS=Mus musculus OX=10090 GN=Mlycd PE=1 SV=1	3.557	3.861789	6.33	1	2	1	54.7	1.506	1.196	1.346	[illegible]
Q7TQ95	Endoplasmic reticulum junction formation protein lunapark OS=Mus musculus OX=10090 GN=Lnpk PE=1 SV=1	3.555	2.117647	2.36	1	1	1	47.5				[illegible]
Q3TCN2	Putative phospholipase B-like 2 OS=Mus musculus OX=10090 GN=Plbd2 PE=1 SV=2	3.535	2.861953	3.5	1	1	1	66.2				[illegible]
P63300	Selenoprotein W OS=Mus musculus OX=10090 GN=Selenow PE=1 SV=3	3.534	11.36364	2.79	1	1	1	9.7	1.138	1.036	1.178	[illegible]
Q91VC7	Protein phosphatase 1 regulatory subunit 14A OS=Mus musculus OX=10090 GN=Ppp1r14a PE=1 SV=1	3.529	7.482993	2.85	1	1	1	16.6				[illegible]
P28028	Serine/threonine-protein kinase B-raf OS=Mus musculus OX=10090 GN=Braf PE=1 SV=3	3.526	1.492537	3.26	1	1	1	88.7	1.46	1.068	1.399	[illegible]
Q3TWL2	Type 1 phosphatidylinositol 4,5-bisphosphate 4-phosphatase OS=Mus musculus OX=10090 GN=Pip4p1 PE=1 SV	3.513	3.521127	2.74	1	1	1	30				[illegible]
Q8BGB7	Enolase-phosphatase E1 OS=Mus musculus OX=10090 GN=Enoph1 PE=1 SV=1	3.509	3.11284	3.41	1	1	1	28.6	1.461	1.145	1.415	[illegible]
Q9Z0G0	PDZ domain-containing protein GIPC1 OS=Mus musculus OX=10090 GN=Gipc1 PE=1 SV=1	3.509	4.504505	2.67	1	1	1	36.1				[illegible]
P48024	Eukaryotic translation initiation factor 1 OS=Mus musculus OX=10090 GN=Eif1 PE=1 SV=2	3.508	11.50442	2.21	1	1	1	12.7				[illegible]
A2AAE1	Transmembrane protein KIAA1109 OS=Mus musculus OX=10090 GN=Kiaa1109 PE=1 SV=4	3.487	0.35964	6.06	2	3	2	555	1.195	1.184	1.376	[illegible]
O35382	Exocyst complex component 4 OS=Mus musculus OX=10090 GN=Exoc4 PE=1 SV=2	3.473	1.025641	3.25	1	1	1	110.5				[illegible]
Q9JKN6	RNA-binding protein Nova-1 OS=Mus musculus OX=10090 GN=Nova1 PE=1 SV=2	3.441	2.761341	7.48	1	2	1	51.7				[illegible]
Q61425	Hydroxyacyl-coenzyme A dehydrogenase, mitochondrial OS=Mus musculus OX=10090 GN=Hadh PE=1 SV=2	3.438	5.414013	10.71	2	4	2	34.4	1.253	1.002	1.247	[illegible]
Q14BB9	MAP6 domain-containing protein 1 OS=Mus musculus OX=10090 GN=Map6d1 PE=1 SV=1	3.424	4.712042	5.3	1	2	1	20.4	1.314	1	1.196	[illegible]
P20352	Tissue factor OS=Mus musculus OX=10090 GN=F3 PE=1 SV=2	3.422	5.782313	2.21	1	1	1	32.9				[illegible]
Q9CZ42	ATP-dependent (S)-NAD(P)H-hydrate dehydratase OS=Mus musculus OX=10090 GN=Naxd PE=1 SV=1	3.394	9.620991	2.78	2	2	2	36.7				[illegible]
P35505	Fumarylacetoacetase OS=Mus musculus OX=10090 GN=Fah PE=1 SV=2	3.392	2.863962	2.93	1	1	1	46.1				[illegible]
P97861	Keratin, type II cuticular Hb6 OS=Mus musculus OX=10090 GN=Krt86 PE=2 SV=2	3.392	3.08642	7.29	2	3	1	53.2				[illegible]
B2RQC6	CAD protein OS=Mus musculus OX=10090 GN=Cad PE=1 SV=1	3.382	0.674157	2.22	1	1	1	243.1	1.263	1.161	1.229	[illegible]
O89079	Coatomer subunit epsilon OS=Mus musculus OX=10090 GN=Cope PE=1 SV=3	3.378	8.441558	0	1	3	1	34.5	1.358	1.026	1.477	[illegible]
Q9D4H8	Cullin-2 OS=Mus musculus OX=10090 GN=Cul2 PE=1 SV=2	3.376	1.47651	2.71	1	1	1	86.8	1.228	1.039	1.228	[illegible]
Q9DBZ5	Eukaryotic translation initiation factor 3 subunit K OS=Mus musculus OX=10090 GN=Eif3k PE=1 SV=1	3.376	6.422018	3.03	1	1	1	25.1				[illegible]
Q9JK42	[Pyruvate dehydrogenase (acetyl-transferring)] kinase isozyme 2, mitochondrial OS=Mus musculus OX=10090 G	3.364	3.194103	6.39	1	2	1	46				[illegible]
Q8BGY7	Protein FAM210A OS=Mus musculus OX=10090 GN=Fam210a PE=1 SV=1	3.364	8.058608	5.53	1	2	1	31.6	1.304	1.256	1.203	[illegible]
Q505F5	Leucine-rich repeat-containing protein 47 OS=Mus musculus OX=10090 GN=Lrrc47 PE=1 SV=1	3.336	2.065404	6.31	1	2	1	63.6	1.129	1.073	1.292	[illegible]
Q78XF5	Oligosaccharyltransferase complex subunit OSTC OS=Mus musculus OX=10090 GN=Ostc PE=1 SV=1	3.308	8.053691	2.79	1	1	1	16.8	1.268	1.065	1.38	[illegible]
P35428	Transcription factor HES-1 OS=Mus musculus OX=10090 GN=Hes1 PE=1 SV=1	3.285	3.191489	2.82	1	1	1	29.7	0.805	0.876	0.849	[illegible]
Q8BL65	Actin-binding LIM protein 2 OS=Mus musculus OX=10090 GN=Ablim2 PE=1 SV=1	3.282	3.267974	4.27	2	3	2	68.1	1.277	1.049	1.22	[illegible]
Q60780	Growth arrest-specific protein 7 OS=Mus musculus OX=10090 GN=Gas7 PE=1 SV=1	3.28	3.800475	2.65	1	1	1	48.1				[illegible]
Q9CQ22	Ragulator complex protein LAMTOR1 OS=Mus musculus OX=10090 GN=Lamtor1 PE=1 SV=1	3.274	8.074534	2.72	1	1	1	17.7				[illegible]
Q99NB9	Splicing factor 3B subunit 1 OS=Mus musculus OX=10090 GN=Sf3b1 PE=1 SV=1	3.272	0.766871	6.19	1	2	1	145.7	1.272	1.05	1.336	[illegible]
Q6PGL7	WASH complex subunit 2 OS=Mus musculus OX=10090 GN=Washc2 PE=1 SV=1	3.265	1.649175	0	1	1	1	145.2				[illegible]
Q0VBF8	Protein stum homolog OS=Mus musculus OX=10090 GN=Stum PE=1 SV=1	3.253	9.219858	2.17	1	1	1	15	1.435	1.207	1.325	[illegible]
Q9CPS6	Histidine triad nucleotide-binding protein 3 OS=Mus musculus OX=10090 GN=Hint3 PE=1 SV=1	3.245	10.90909	4.02	2	3	2	18.8				[illegible]
P08207	Protein S100-A10 OS=Mus musculus OX=10090 GN=S100a10 PE=1 SV=2	3.235	17.52577	2.92	1	1	1	11.2	1.501	1.112	1.365	[illegible]
Q99M71	Mammalian ependymin-related protein 1 OS=Mus musculus OX=10090 GN=Epdr1 PE=1 SV=1	3.226	5.357143	2.65	1	1	1	25.5	1.021	0.917	1.114	[illegible]
S4R1M9	Oxysterol-binding protein-related protein 10 OS=Mus musculus OX=10090 GN=Osbpl10 PE=1 SV=1	3.224	1.305483	3.33	1	1	1	83.8				[illegible]
P30999	Catenin delta-1 OS=Mus musculus OX=10090 GN=Ctnnd1 PE=1 SV=2	3.218	1.705757	3.3	1	1	1	104.9				[illegible]
Q921Q7	Ras and Rab interactor 1 OS=Mus musculus OX=10090 GN=Rin1 PE=1 SV=1	3.217	2.096986	0	1	1	1	83				[illegible]
Q9JHL1	Na(+)/H(+) exchange regulatory cofactor NHE-RF2 OS=Mus musculus OX=10090 GN=Slc9a3r2 PE=1 SV=2	3.177	5.04451	3.2	1	1	1	37.4	1.1	1.062	1.122	[illegible]
Q9CQ65	S-methyl-5'-thioadenosine phosphorylase OS=Mus musculus OX=10090 GN=Mtap PE=1 SV=1	3.17	3.533569	2.56	1	1	1	31				[illegible]
O88998	Noelin OS=Mus musculus OX=10090 GN=Olfm1 PE=1 SV=1	3.156	2.680412	8.95	2	4	2	55.4	1.086	1.027	1.137	[illegible]
Q61655	ATP-dependent RNA helicase DDX19A OS=Mus musculus OX=10090 GN=Ddx19a PE=1 SV=2	3.147	3.34728	4.66	2	2	2	53.9	0.999	0.859	0.932	[illegible]
Q9D1X0	Nucleolar protein 3 OS=Mus musculus OX=10090 GN=Nol3 PE=1 SV=1	3.134	4.545455	2.08	1	1	1	24.6				[illegible]
Q6PD28	Serine/threonine-protein phosphatase 2A 56 kDa regulatory subunit beta isoform OS=Mus musculus OX=10090	3.13	2.615694	2.62	1	1	1	57.3	1.129	1.061	1.196	[illegible]
Q62376	U1 small nuclear ribonucleoprotein 70 kDa OS=Mus musculus OX=10090 GN=Snrnp70 PE=1 SV=2	3.128	2.455357	3.18	1	1	1	52				[illegible]
Q8VCH8	UBX domain-containing protein 4 OS=Mus musculus OX=10090 GN=Ubxn4 PE=1 SV=1	3.095	4.150198	2.63	1	1	1	56.4				[illegible]
Q9JKW0	ADP-ribosylation factor-like protein 6-interacting protein 1 OS=Mus musculus OX=10090 GN=Arl6ip1 PE=1 SV=	3.083	3.940887	2.28	1	1	1	23.4				[illegible]
Q8BPU7	Engulfment and cell motility protein 1 OS=Mus musculus OX=10090 GN=Elmo1 PE=1 SV=2	3.07	2.475928	2.76	1	1	1	83.9				[illegible]
Q9CX80	Cytoglobin OS=Mus musculus OX=10090 GN=Cygb PE=1 SV=1	3.067	4.736842	2.56	1	1	1	21.5				[illegible]
Q80VL1	Tudor and KH domain-containing protein OS=Mus musculus OX=10090 GN=Tdrkh PE=1 SV=1	3.062	3.035714	2.02	1	1	1	62.1				[illegible]
Q80ZJ6	Protein zer-1 homolog OS=Mus musculus OX=10090 GN=Zer1 PE=1 SV=1	3.06	1.925546	3.35	1	1	1	89				[illegible]
P61620	Protein transport protein Sec61 subunit alpha isoform 1 OS=Mus musculus OX=10090 GN=Sec61a1 PE=1 SV=2	3.052	1.890756	2.88	1	3	1	52.2	1.51	1.709	1.644	[illegible]
P17095	High mobility group protein HMG-I/HMG-Y OS=Mus musculus OX=10090 GN=Hmga1 PE=1 SV=4	3.047	8.411215	2.86	1	1	1	11.6	1.203	0.997	1.092	[illegible]
Q8BTG3	T-complex protein 11-like protein 1 OS=Mus musculus OX=10090 GN=Tcp11l1 PE=1 SV=1	3.039	2.357564	3.24	1	1	1	56.3				[illegible]
Q8CCN5	Breast carcinoma-amplified sequence 3 homolog OS=Mus musculus OX=10090 GN=Bcas3 PE=1 SV=2	3.03	1.508621	2.26	1	1	1	101	0.877	0.831	0.986	[illegible]
Q9QUH0	Glutaredoxin-1 OS=Mus musculus OX=10090 GN=Glrx PE=1 SV=3	3.025	10.28037	3.67	1	1	1	11.9				[illegible]
Q9R1Q9	V-type proton ATPase subunit S1 OS=Mus musculus OX=10090 GN=Atp6ap1 PE=1 SV=1	3.007	3.239741	3.02	1	1	1	51				[illegible]
Q6NZL0	Protein SOGA3 OS=Mus musculus OX=10090 GN=Soga3 PE=1 SV=2	3.002	1.164021	2.33	1	1	1	103.4	1.166	1.032	1.15	[illegible]
O08600	Endonuclease G, mitochondrial OS=Mus musculus OX=10090 GN=Endog PE=1 SV=1	2.987	4.421769	3.14	1	1	1	32.2	1.386	1.242	1.391	[illegible]
O08582	GTP-binding protein 1 OS=Mus musculus OX=10090 GN=Gtpbp1 PE=1 SV=2	2.982	2.844311	2.87	1	1	1	72.3				[illegible]
Q99NF2	NMDA receptor synaptonuclear signaling and neuronal migration factor OS=Mus musculus OX=10090 GN=Nsm	2.98	2.631579	2.38	1	1	1	60.3	1.313	1.039	1.394	[illegible]
P62309	Small nuclear ribonucleoprotein G OS=Mus musculus OX=10090 GN=Snrpg PE=1 SV=1	2.972	9.210526	7.42	1	3	1	8.5				[illegible]
Q62417	Sorbin and SH3 domain-containing protein 1 OS=Mus musculus OX=10090 GN=Sorbs1 PE=1 SV=2	2.971	1.24031	4.02	1	1	1	143				[illegible]
Q9D832	DnaJ homolog subfamily B member 4 OS=Mus musculus OX=10090 GN=Dnajb4 PE=1 SV=1	2.964	5.637982	2.17	2	2	2	37.8	1.308	1.283	1.375	[illegible]
P58021	Transmembrane 9 superfamily member 2 OS=Mus musculus OX=10090 GN=Tm9sf2 PE=1 SV=1	2.961	2.114804	2.93	1	1	1	75.3				[illegible]
Q9CWM4	Prefoldin subunit 1 OS=Mus musculus OX=10090 GN=Pfdn1 PE=1 SV=1	2.933	8.196721	2.44	1	1	1	14.2				[illegible]
Q8BMJ2	Leucine--tRNA ligase, cytoplasmic OS=Mus musculus OX=10090 GN=Lars PE=1 SV=2	2.929	1.103565	2.98	1	1	1	134.1				[illegible]
Q8VHN8	Tudor-interacting repair regulator protein OS=Mus musculus OX=10090 GN=Nudt16l1 PE=1 SV=2	2.924	5.21327	2.21	1	1	1	23.4	1.142	0.995	1.279	[illegible]
Q9CQQ8	U6 snRNA-associated Sm-like protein LSm7 OS=Mus musculus OX=10090 GN=Lsm7 PE=1 SV=1	2.924	25.24272	4.77	1	2	1	11.6	1.024	0.959	1.115	[illegible]
Q569Z6	Thyroid hormone receptor-associated protein 3 OS=Mus musculus OX=10090 GN=Thrap3 PE=1 SV=1	2.872	0.84122	2.03	1	1	1	108.1	1.213	1.059	1.219	[illegible]
Q91W69	Epsin-3 OS=Mus musculus OX=10090 GN=Epn3 PE=1 SV=1	2.871	1.572327	2.83	1	1	1	68.2				[illegible]
Q8BMG7	Rab3 GTPase-activating protein non-catalytic subunit OS=Mus musculus OX=10090 GN=Rab3gap2 PE=1 SV=2	2.871	0.951684	3.11	1	1	1	152.4				[illegible]
P61514	60S ribosomal protein L37a OS=Mus musculus OX=10090 GN=Rpl37a PE=1 SV=2	2.85	8.695652	2.38	1	1	1	10.3	1.569	1.167	1.23	[illegible]
Q3UTJ2	Sorbin and SH3 domain-containing protein 2 OS=Mus musculus OX=10090 GN=Sorbs2 PE=1 SV=2	2.849	0.762712	5.21	1	2	1	132.3	1.18	1.072	1.26	[illegible]
Q8BG02	Serine/threonine-protein phosphatase 2A 55 kDa regulatory subunit B gamma isoform OS=Mus musculus OX=1(	2.849	3.131991	0	1	1	1	51.4				[illegible]
Q9D975	Sulfiredoxin-1 OS=Mus musculus OX=10090 GN=Srxn1 PE=2 SV=1	2.842	17.64706	2.24	1	1	1	14.1				[illegible]
P11531	Dystrophin OS=Mus musculus OX=10090 GN=Dmd PE=1 SV=3	2.829	0.489396	2.17	1	1	1	425.6				[illegible]
Q9ER58	Testican-2 OS=Mus musculus OX=10090 GN=Spock2 PE=1 SV=1	2.826	4.018913	0	1	1	1	46.8				[illegible]
P63089	Pleiotrophin OS=Mus musculus OX=10090 GN=Ptn PE=1 SV=1	2.825	5.357143	0	1	1	1	18.9				[illegible]
Q5SNZ0	Girdin OS=Mus musculus OX=10090 GN=Ccdc88a PE=1 SV=2	2.824	0.694074	2.94	1	1	1	215.8				[illegible]
Q9WU79	Proline dehydrogenase 1, mitochondrial OS=Mus musculus OX=10090 GN=Prodh PE=1 SV=2	2.809	2.003339	2.61	1	1	1	68				[illegible]
P21126	Ubiquitin-like protein 4A OS=Mus musculus OX=10090 GN=Ubl4a PE=1 SV=1	2.795	10.19108	3.19	1	1	1	17.8				[illegible]
Q9QZS3	Protein numb homolog OS=Mus musculus OX=10090 GN=Numb PE=1 SV=1	2.793	2.29709	2.41	1	1	1	70.8				[illegible]
Q9D061	Acyl-CoA-binding domain-containing protein 6 OS=Mus musculus OX=10090 GN=Acbd6 PE=1 SV=2	2.787	4.609929	2.75	1	1	1	30.9				[illegible]
P98203	Armadillo repeat protein deleted in velo-cardio-facial syndrome homolog OS=Mus musculus OX=10090 GN=Arv	2.77	1.559252	3.18	1	1	1	105				[illegible]
P59281	Rho GTPase-activating protein 39 OS=Mus musculus OX=10090 GN=Arhgap39 PE=1 SV=2	2.762	1.445348	3.06	1	1	1	125.1				[illegible]
B9EJ86	Oxysterol-binding protein-related protein 8 OS=Mus musculus OX=10090 GN=Osbpl8 PE=1 SV=1	2.757	1.687289	2.07	1	2	1	101.2				[illegible]
P11438	Lysosome-associated membrane glycoprotein 1 OS=Mus musculus OX=10090 GN=Lamp1 PE=1 SV=2	2.753	2.955665	2.82	1	1	1	43.8				[illegible]
O35668	Huntingtin-associated protein 1 OS=Mus musculus OX=10090 GN=Hap1 PE=1 SV=1	2.753	1.433121	2.78	1	1	1	70.1	1.322	0.928	1.171	[illegible]
Q9DC33	High mobility group protein 20A OS=Mus musculus OX=10090 GN=Hmg20a PE=1 SV=1	2.752	4.913295	2.15	1	1	1	39.9				[illegible]
Q8BHL5	Engulfment and cell motility protein 2 OS=Mus musculus OX=10090 GN=Elmo2 PE=1 SV=1	2.741	1.092896	2.08	1	2	1	83.8	1.271	1.123	1.226	[illegible]
Q8VH51	RNA-binding protein 39 OS=Mus musculus OX=10090 GN=Rbm39 PE=1 SV=2	2.736	2.075472	5.37	1	2	1	59.4				[illegible]
O08576	RUN domain-containing protein 3A OS=Mus musculus OX=10090 GN=Rundc3a PE=1 SV=1	2.694	2.690583	2.3	1	1	1	50	1.306	0.994	1.27	[illegible]
P0C7L0	WAS/WASL-interacting protein family member 3 OS=Mus musculus OX=10090 GN=Wipf3 PE=1 SV=1	2.69	1.85567	2.73	1	1	1	49.4	1.13	0.998	1.167	[illegible]
Q9JIX8	Apoptotic chromatin condensation inducer in the nucleus OS=Mus musculus OX=10090 GN=Acin1 PE=1 SV=3	2.689	1.195815	2.07	1	1	1	150.6				[illegible]
A2CG49	Kalirin OS=Mus musculus OX=10090 GN=Kalrn PE=1 SV=1	2.687	0.37112	3.01	1	1	1	336.8				[illegible]
Q91V76	Ester hydrolase C11orf54 homolog OS=Mus musculus OX=10090 PE=1 SV=1	2.686	3.809524	4.69	1	2	1	35	1.317	1.159	1.37	[illegible]
Q9DCT1	1,5-anhydro-D-fructose reductase OS=Mus musculus OX=10090 GN=Akr1e2 PE=1 SV=1	2.683	6.312292	6.45	2	3	1	34.4				[illegible]
Q8VDK1	Deaminated glutathione amidase OS=Mus musculus OX=10090 GN=Nit1 PE=1 SV=2	2.655	3.095975	5.8	1	2	1	35.7				[illegible]
Q8BGR9	Ubiquitin-like domain-containing CTD phosphatase 1 OS=Mus musculus OX=10090 GN=Ublcp1 PE=1 SV=1	2.641	3.144654	2.91	1	2	1	36.8	1.453	1.104	1.433	[illegible]
Q99PG2	Opioid growth factor receptor OS=Mus musculus OX=10090 GN=Ogfr PE=1 SV=1	2.633	7.109005	2.24	1	2	1	70.6				[illegible]
Q76LS9	Ubiquitin carboxyl-terminal hydrolase MINDY-1 OS=Mus musculus OX=10090 GN=Mindy1 PE=1 SV=1	2.632	2.564103	2.39	1	2	1	51.2	1.377	1.051	1.344	[illegible]
Q9Z2D0	Myotubularin-related protein 9 OS=Mus musculus OX=10090 GN=Mtmr9 PE=1 SV=2	2.628	2.018349	4.89	1	2	1	62.9	1.325	1.09	1.211	[illegible]
Q3UHH2	Solute carrier family 22 member 23 OS=Mus musculus OX=10090 GN=Slc22a23 PE=2 SV=1	2.621	1.451379	4.65	1	2	1	74.3	1.23	1.175	1.355	[illegible]
Q9Z1M8	Protein Red OS=Mus musculus OX=10090 GN=Ik PE=1 SV=2	2.62	2.333932	2.91	1	1	1	65.6				[illegible]
Q99KW9	T-cell immunomodulatory protein OS=Mus musculus OX=10090 GN=Itfg1 PE=1 SV=2	2.62	2.295082	0	1	1	1	67.4	1.063	1.013	1.095	[illegible]
Q8BPQ7	Small G protein signaling modulator 1 OS=Mus musculus OX=10090 GN=Sgsm1 PE=1 SV=2	2.615	1.006404	1.89	1	1	1	123.1	1.292	1.116	1.394	[illegible]
Q9R0Q3	Transmembrane emp24 domain-containing protein 2 OS=Mus musculus OX=10090 GN=Tmed2 PE=1 SV=1	2.591	4.477612	2.04	1	2	1	22.7	1.605	1.157	1.387	[illegible]
B9EJA2	Cortactin-binding protein 2 OS=Mus musculus OX=10090 GN=Cttnbp2 PE=1 SV=2	2.59	0.728155	1.91	1	1	1	178.7	1.135	0.991	1.03	[illegible]
Q9D6Y7	Mitochondrial peptide methionine sulfoxide reductase OS=Mus musculus OX=10090 GN=Msra PE=1 SV=1	2.583	3.862661	5.23	1	2	1	26				[illegible]
Q91YX5	Acyl-CoA:lysophosphatidylglycerol acyltransferase 1 OS=Mus musculus OX=10090 GN=Lpgat1 PE=1 SV=1	2.568	2.162162	2.34	1	1	1	43.1	1.313	1.228	1.353	[illegible]
A2ALU4	Protein Shroom2 OS=Mus musculus OX=10090 GN=Shroom2 PE=1 SV=1	2.568	0.607698	2.63	1	1	1	164.6				[illegible]
Q9JMH6	Thioredoxin reductase 1, cytoplasmic OS=Mus musculus OX=10090 GN=Txnrd1 PE=1 SV=3	2.567	1.468189	1.77	1	1	1	67				[illegible]
P61211	ADP-ribosylation factor-like protein 1 OS=Mus musculus OX=10090 GN=Arl1 PE=1 SV=1	2.544	4.972376	4.96	1	2	1	20.4	0.954	1.306	1.284	[illegible]
Q08460	Calcium-activated potassium channel subunit alpha-1 OS=Mus musculus OX=10090 GN=Kcnma1 PE=1 SV=2	2.538	0.82713	2.2	1	1	1	134.3				[illegible]
Q922D4	Serine/threonine-protein phosphatase 6 regulatory subunit 3 OS=Mus musculus OX=10090 GN=Ppp6r3 PE=1 SV	2.534	1.184834	2.16	1	1	1	94.6				[illegible]
Q80WM4	Hyaluronan and proteoglycan link protein 4 OS=Mus musculus OX=10090 GN=Hapln4 PE=2 SV=2	2.528	4	2.05	1	1	1	42.8	1.359	1.154	1.282	[illegible]
Q9JL04	Formin-2 OS=Mus musculus OX=10090 GN=Fmn2 PE=1 SV=2	2.502	1.204056	2.53	1	1	1	167.3				[illegible]
O55222	Integrin-linked protein kinase OS=Mus musculus OX=10090 GN=Ilk PE=1 SV=2	2.502	3.097345	6.57	1	2	1	51.3	0.495	0.471	0.524	[illegible]
Q3UHK1	Proton myo-inositol cotransporter OS=Mus musculus OX=10090 GN=Slc2a13 PE=1 SV=2	2.499	2.040816	2.08	1	1	1	69				[illegible]
Q6P5F7	Protein tweety homolog 3 OS=Mus musculus OX=10090 GN=Ttyh3 PE=1 SV=1	2.498	1.335878	4.95	1	2	1	57.7	0.379	0.241	0.266	[illegible]
P83870	PHD finger-like domain-containing protein 5A OS=Mus musculus OX=10090 GN=Phf5a PE=1 SV=1	2.492	6.363636	2.36	1	1	1	12.4				[illegible]
Q6DFW4	Nucleolar protein 58 OS=Mus musculus OX=10090 GN=Nop58 PE=1 SV=1	2.476	2.238806	3.06	1	1	1	60.3	1.31	1.106	1.364	[illegible]
Q9EPC1	Alpha-parvin OS=Mus musculus OX=10090 GN=Parva PE=1 SV=1	2.476	3.763441	2.32	1	2	1	42.3				[illegible]
Q3UVL4	Vacuolar protein sorting-associated protein 51 homolog OS=Mus musculus OX=10090 GN=Vps51 PE=1 SV=2	2.474	1.40665	2.62	1	1	1	86.1				[illegible]
Q9JKS5	Intracellular hyaluronan-binding protein 4 OS=Mus musculus OX=10090 GN=Habp4 PE=1 SV=2	2.469	2.189781	3.14	1	1	1	45.9				[illegible]
A6H630	Damage-control phosphatase ARMT1 OS=Mus musculus OX=10090 GN=Armt1 PE=1 SV=1	2.45	1.594533	2.18	1	1	1	50.5				[illegible]
P61022	Calcineurin B homologous protein 1 OS=Mus musculus OX=10090 GN=Chp1 PE=1 SV=2	2.424	6.153846	4.74	1	2	1	22.4	1.272	1.062	1.307	[illegible]
Q6GQS1	Calcium-binding mitochondrial carrier protein SCaMC-3 OS=Mus musculus OX=10090 GN=Slc25a23 PE=1 SV=1	2.424	3.426124	6.24	2	3	1	52.5	1.29	1.145	1.257	[illegible]
Q9JKV1	Proteasomal ubiquitin receptor ADRM1 OS=Mus musculus OX=10090 GN=Adrm1 PE=1 SV=2	2.417	1.965602	2.25	1	1	1	42				[illegible]
Q8VDP4	Cell cycle and apoptosis regulator protein 2 OS=Mus musculus OX=10090 GN=Ccar2 PE=1 SV=2	2.409	1.193059	2.36	1	1	1	102.9				[illegible]
Q5XG69	Soluble lamin-associated protein of 75 kDa OS=Mus musculus OX=10090 GN=Fam169a PE=1 SV=3	2.396	1.353383	2.15	1	1	1	73.2				[illegible]
Q80WG5	Volume-regulated anion channel subunit LRRC8A OS=Mus musculus OX=10090 GN=Lrrc8a PE=1 SV=1	2.382	1.234568	2.39	1	1	1	94.1				[illegible]
P09528	Ferritin heavy chain OS=Mus musculus OX=10090 GN=Fth1 PE=1 SV=2	2.379	3.846154	2.22	1	1	1	21.1				[illegible]
Q80XK6	Autophagy-related protein 2 homolog B OS=Mus musculus OX=10090 GN=Atg2b PE=1 SV=3	2.375	1.253012	0	1	1	1	231.3				[illegible]
Q8R0F8	Acylpyruvase FAHD1, mitochondrial OS=Mus musculus OX=10090 GN=Fahd1 PE=1 SV=2	2.361	3.0837	1.87	1	1	1	25.2	1.156	1.097	1.141	[illegible]
O08848	60 kDa SS-A/Ro ribonucleoprotein OS=Mus musculus OX=10090 GN=RO60 PE=1 SV=1	2.359	1.672862	2.83	1	1	1	60.1				[illegible]
P70362	Ubiquitin recognition factor in ER-associated degradation protein 1 OS=Mus musculus OX=10090 GN=Ufd1 PE=	2.347	4.234528	2.9	1	1	1	34.5	1.329	1.137	1.267	[illegible]
Q61239	Protein farnesyltransferase/geranylgeranyltransferase type-1 subunit alpha OS=Mus musculus OX=10090 GN=Fr	2.342	7.427056	2.46	2	4	2	44	1.461	0.944	1.288	[illegible]
Q8C052	Microtubule-associated protein 1S OS=Mus musculus OX=10090 GN=Map1s PE=1 SV=2	2.327	1.336074	0	1	1	1	102.9				[illegible]
Q7TMC8	L-fucose kinase OS=Mus musculus OX=10090 GN=Fcsk PE=1 SV=1	2.325	1.284404	2.55	1	1	1	119.2				[illegible]
Q9JLN9	Serine/threonine-protein kinase mTOR OS=Mus musculus OX=10090 GN=Mtor PE=1 SV=2	2.316	0.941546	1.68	2	3	2	288.6				[illegible]
O08688	Calpain-5 OS=Mus musculus OX=10090 GN=Capn5 PE=1 SV=1	2.313	1.5625	2.55	1	1	1	72.9	1.26	0.928	1.314	[illegible]
Q8BXZ1	Protein disulfide-isomerase TMX3 OS=Mus musculus OX=10090 GN=Tmx3 PE=1 SV=2	2.309	1.754386	0	1	1	1	51.8				[illegible]
Q3TPX4	Exocyst complex component 5 OS=Mus musculus OX=10090 GN=Exoc5 PE=1 SV=2	2.281	1.129944	2.34	1	1	1	81.7	1.195	1.161	1.228	[illegible]
F8VPU2	FERM, ARHGEF and pleckstrin domain-containing protein 1 OS=Mus musculus OX=10090 GN=Farp1 PE=1 SV=1	2.267	1.049618	4.35	1	2	1	118.8				[illegible]
Q64010	Adapter molecule crk OS=Mus musculus OX=10090 GN=Crk PE=1 SV=1	2.265	2.631579	0	1	1	1	33.8				[illegible]
Q9R0H0	Peroxisomal acyl-coenzyme A oxidase 1 OS=Mus musculus OX=10090 GN=Acox1 PE=1 SV=5	2.245	2.118003	2.22	1	2	1	74.6	1.074	0.965	1.146	[illegible]
Q9D024	Coiled-coil domain-containing protein 47 OS=Mus musculus OX=10090 GN=Ccdc47 PE=1 SV=2	2.239	3.519669	2.35	1	2	1	55.8				[illegible]
Q80TM9	Nischarin OS=Mus musculus OX=10090 GN=Nisch PE=1 SV=2	2.239	0.753296	5.08	1	2	1	174.9	1.286	1.199	1.43	[illegible]
O35639	Annexin A3 OS=Mus musculus OX=10090 GN=Anxa3 PE=1 SV=4	2.229	3.405573	0	1	1	1	36.4				[illegible]
Q8K4Z5	Splicing factor 3A subunit 1 OS=Mus musculus OX=10090 GN=Sf3a1 PE=1 SV=1	2.225	1.643489	2.23	1	1	1	88.5				[illegible]
P70698	CTP synthase 1 OS=Mus musculus OX=10090 GN=Ctps1 PE=1 SV=2	2.22	2.707276	2.43	1	1	1	66.6				[illegible]
Q9Z1Q5	Chloride intracellular channel protein 1 OS=Mus musculus OX=10090 GN=Clic1 PE=1 SV=3	2.217	7.46888	3.5	1	1	1	27				[illegible]
P70429	Ena/VASP-like protein OS=Mus musculus OX=10090 GN=Evl PE=1 SV=2	2.205	3.140097	1.69	1	1	1	44.3	1.111	1.019	1.157	[illegible]
Q80VP0	Tectonin beta-propeller repeat-containing protein 1 OS=Mus musculus OX=10090 GN=Tecpr1 PE=1 SV=1	2.198	0.857633	0	1	1	1	130.2				[illegible]
Q8BFQ8	Glutamine amidotransferase-like class 1 domain-containing protein 1 OS=Mus musculus OX=10090 GN=Gatd1 I	2.195	5.909091	0	1	1	1	23.3				[illegible]
Q9R062	Glycogenin-1 OS=Mus musculus OX=10090 GN=Gyg1 PE=1 SV=3	2.187	3.003003	2.86	1	1	1	37.4				[illegible]
Q9QXB9	Developmentally-regulated GTP-binding protein 2 OS=Mus musculus OX=10090 GN=Drg2 PE=1 SV=1	2.186	2.197802	2.42	1	1	1	40.7	1.264	1.02	1.223	[illegible]
Q8C3Q5	Protein shisa-7 OS=Mus musculus OX=10090 GN=Shisa7 PE=1 SV=3	2.184	1.612903	3.17	1	1	1	58.3	1.437	1.219	1.411	[illegible]
Q9R1B9	Slit homolog 2 protein OS=Mus musculus OX=10090 GN=Slit2 PE=2 SV=2	2.182	0.52597	2.09	1	1	1	168.7				[illegible]
Q9CQ06	39S ribosomal protein L24, mitochondrial OS=Mus musculus OX=10090 GN=Mrpl24 PE=1 SV=1	2.173	6.481481	1.74	1	1	1	24.9				[illegible]
Q91Z38	Tetratricopeptide repeat protein 1 OS=Mus musculus OX=10090 GN=Ttc1 PE=1 SV=1	2.171	3.424658	2.24	1	1	1	33.2				[illegible]
Q9QY06	Unconventional myosin-IXb OS=Mus musculus OX=10090 GN=Myo9b PE=1 SV=2	2.162	0.331126	4.73	1	2	1	238.7	1.328	1.284	1.22	[illegible]
Q8BIG7	Catechol O-methyltransferase domain-containing protein 1 OS=Mus musculus OX=10090 GN=Comtd1 PE=1 SV	2.16	3.435115	4.29	1	2	1	28.9	1.292	1.121	1.259	[illegible]
Q9JIG8	PRA1 family protein 2 OS=Mus musculus OX=10090 GN=Praf2 PE=1 SV=1	2.151	10.11236	2.13	1	1	1	19.5	1.286	1.219	1.37	[illegible]
Q924D0	Reticulon-4-interacting protein 1, mitochondrial OS=Mus musculus OX=10090 GN=Rtn4ip1 PE=1 SV=2	2.142	3.282828	3.44	1	1	1	43.3	1.298	1.119	1.205	[illegible]
Q6PHZ8	Kv channel-interacting protein 4 OS=Mus musculus OX=10090 GN=Kcnip4 PE=1 SV=1	2.136	2.8	8.13	1	4	1	28.7	1.394	1.169	1.322	[illegible]
Q62448	Eukaryotic translation initiation factor 4 gamma 2 OS=Mus musculus OX=10090 GN=Eif4g2 PE=1 SV=2	2.136	1.103753	2.43	1	1	1	102	1.226	0.972	1.135	[illegible]
A2AN08	E3 ubiquitin-protein ligase UBR4 OS=Mus musculus OX=10090 GN=Ubr4 PE=1 SV=1	2.132	0.19305	2.26	1	1	1	571.9				[illegible]
A2AWA9	Rab GTPase-activating protein 1 OS=Mus musculus OX=10090 GN=Rabgap1 PE=1 SV=1	2.124	0.845865	2.19	1	1	1	120.7				[illegible]
Q9CQR2	40S ribosomal protein S21 OS=Mus musculus OX=10090 GN=Rps21 PE=1 SV=1	2.11	8.433735	2.63	1	1	1	9.1				[illegible]
Q99MK8	Beta-adrenergic receptor kinase 1 OS=Mus musculus OX=10090 GN=Grk2 PE=1 SV=2	2.101	1.161103	5.41	1	2	1	79.6				[illegible]
Q3UEB3	Poly(U)-binding-splicing factor PUF60 OS=Mus musculus OX=10090 GN=Puf60 PE=1 SV=2	2.087	1.241135	2.44	1	1	1	60.2				[illegible]
P56382	ATP synthase subunit epsilon, mitochondrial OS=Mus musculus OX=10090 GN=Atp5f1e PE=1 SV=2	2.069	13.46154	5.13	1	2	1	5.8	1.248	0.988	1.253	[illegible]
Q80VP1	Epsin-1 OS=Mus musculus OX=10090 GN=Epn1 PE=1 SV=3	2.064	1.73913	2.89	1	1	1	60.2	1.624	1.235	1.529	[illegible]

Table S3. Top pathway gene sets detected from the proteome signature of HFD^{EVs}-PE^{high} administrated 5XFAD mice, related to Figure 3H.

Category	Set Name	#Overlapped Proteins	#Queried Proteins	(Col C/Col D) %	Overlapped Protein IDs	#Member Proteins in Set	#Proteins in Category	Fold Enrichment	Over-representation p-values	Bonferroni	Benjamini	FDR
**UP_KW_PTM**	KW-0007~Acetylation	17	27	56.667	P34884, Q9D7A8, Q9CQ60, Q5U458, P70318, P14131, Q9QUM9, P01942, P0DP27, P60761, Q60829, Q8VED9, Q91XU3	3161	12816	2.552778657	7.08E-05	8.49E-04	9.20E-04	9.20E-04
**GOTERM_CC_DIRECT**	GO:0043209~myelin sheath	5	29	16.667	P02088, P12787, P01942, P34884, P0DP27	192	21179	19.01849856	1.13E-04	0.012915365	0.012998759	0.012772693
**UP_KW_CELLULAR_COMPONENT**	KW-0963~Cytoplasm	15	25	50.000	Q9CPX6, P34884, Q9D7A8, Q05BC3, Q9CQ60, Q8R071, Q9WV34, P70318, Q8R570, P16054, Q9QUM9, P0DP27, P60761, Q60829, Q91XU3	5081	17960	2.120842354	0.001965923	0.042369117	0.043250306	0.043250306
**GOTERM_CC_DIRECT**	GO:0098978~glutamatergic synapse	5	29	16.667	Q8R570, P16054, P60761, Q9WV34, Q60829	522	21179	6.995309816	0.004670479	0.416297162	0.268552538	0.263882059
**GOTERM_CC_DIRECT**	GO:0043025~neuronal cell body	5	29	16.667	Q8R570, P11627, Q0VGU4, P60761, Q60829	643	21179	5.678929587	0.009657305	0.672408371	0.370196683	0.36375848
**UP_KW_BIOLOGICAL_PROCESS**	KW-0561~Oxygen transport	2	14	6.667	P02088, P01942	10	11003	157.1857143	0.011757124	0.210642303	0.194218937	0.194218937
**UP_SEQ_FEATURE**	METAL:Iron (heme b distal ligand)	2	28	6.667	P02088, P01942	10	22511	160.7928571	0.011931979	0.799812458	0.541431373	0.541431373
**UP_SEQ_FEATURE**	METAL:Iron (heme b proximal ligand)	2	28	6.667	P02088, P01942	10	22511	160.7928571	0.011931979	0.799812458	0.541431373	0.541431373
**GOTERM_BP_DIRECT**	GO:0050996~positive regulation of lipid catabolic process	2	29	6.667	P09813, P16054	9	20094	153.9770115	0.012473852	0.981530462	1	1
**GOTERM_BP_DIRECT**	GO:0015671~oxygen transport	2	29	6.667	P02088, P01942	10	20094	138.5793103	0.013850538	0.988147989	1	1
**GOTERM_MF_DIRECT**	GO:0031720~haptoglobin binding	2	28	6.667	P02088, P01942	11	18713	121.512987	0.015761496	0.877186435	1	1
**UP_SEQ_FEATURE**	METAL:Iron (heme distal ligand)	2	28	6.667	P02088, P01942	14	22511	114.8520408	0.01666628	0.894822006	0.541431373	0.541431373
**GOTERM_CC_DIRECT**	GO:0005833~hemoglobin complex	2	29	6.667	P02088, P01942	13	21179	112.3554377	0.017055956	0.861703412	0.386744271	0.380018284
**KEGG_PATHWAY**	mmu05012:Parkinson disease	4	20	13.333	Q8BK30, P12787, P0DP27, Q9QUM9	264	8992	6.812121212	0.017117142	0.788573966	0.77130599	0.77130599
**KEGG_PATHWAY**	mmu04070:Phosphatidylinositol signaling system	3	20	10.000	P0DP27, Q8R071, Q91XU3	96	8992	14.05	0.017140133	0.7890186	0.77130599	0.77130599
**UP_SEQ_FEATURE**	DOMAIN:GLOBIN	2	28	6.667	P02088, P01942	15	22511	107.1952381	0.017846438	0.910455468	0.541431373	0.541431373
**GOTERM_CC_DIRECT**	GO:0031838~haptoglobin-hemoglobin complex	2	29	6.667	P02088, P01942	14	21179	104.3300493	0.01835627	0.881232349	0.386744271	0.380018284
**UP_KW_BIOLOGICAL_PROCESS**	KW-0813~Transport	7	14	23.333	P02088, Q8BK30, P09813, Q9JKD3, Q9CPX6, P01942, Q91XL9	2006	11003	2.742522433	0.019421894	0.324471233	0.194218937	0.194218937
**GOTERM_MF_DIRECT**	GO:0043177~organic acid binding	2	28	6.667	P02088, P01942	14	18713	95.4744898	0.020018395	0.930694781	1	1
**UP_SEQ_FEATURE**	METAL:Iron (heme proximal ligand)	2	28	6.667	P02088, P01942	17	22511	94.58403361	0.020202663	0.935097993	0.541431373	0.541431373
**GOTERM_CC_DIRECT**	GO:0031966~mitochondrial membrane	3	29	10.000	P12787, P0DP27, P60761	170	21179	12.88782961	0.021117223	0.914092899	0.386744271	0.380018284
**INTERPRO**	IPR000971:Globin	2	30	6.667	P02088, P01942	16	20908	87.11666667	0.021970888	0.893945286	0.739686579	0.739686579
**INTERPRO**	IPR009050:Globin-like	2	30	6.667	P02088, P01942	16	20908	87.11666667	0.021970888	0.893945286	0.739686579	0.739686579
**INTERPRO**	IPR012292:Globin, structural domain	2	30	6.667	P02088, P01942	16	20908	87.11666667	0.021970888	0.893945286	0.739686579	0.739686579
**GOTERM_MF_DIRECT**	GO:0005344~oxygen transporter activity	2	28	6.667	P02088, P01942	16	18713	83.54017857	0.022846472	0.952675048	1	1
**UP_KW_PTM**	KW-0597~Phosphoprotein	22	27	73.333	P12787, Q9D7A8, Q0VGU4, Q9CQ60, Q8R071, Q5U458, Q9WV34, Q91XL9, P70318, P14131, P16054, Q9QUM9, P01942, P0DP27, P60761, Q60829,	7742	12816	1.348833204	0.023504848	0.248305979	0.152781515	0.152781515
**GOTERM_CC_DIRECT**	GO:0044327~dendritic spine head	2	29	6.667	P60761, Q60829	18	21179	81.14559387	0.023540956	0.935402796	0.386744271	0.380018284
**GOTERM_BP_DIRECT**	GO:0035556~intracellular signal transduction	4	29	13.333	P16054, Q9Z268, P60761, Q60829	450	20094	6.15908046	0.024139846	0.999578059	1	1
**GOTERM_BP_DIRECT**	GO:1900242~regulation of synaptic vesicle endocytosis	2	29	6.667	Q9JKD3, P0DP27	18	20094	76.98850575	0.024797597	0.999659486	1	1
**UP_KW_LIGAND**	KW-0349~Heme	3	11	10.000	P02088, P12787, P01942	174	6582	10.31661442	0.027197929	0.240994601	0.27197929	0.27197929
**GOTERM_BP_DIRECT**	GO:0098869~cellular oxidant detoxification	2	29	6.667	P02088, P01942	20	20094	69.28965517	0.027515998	0.99985984	1	1

**GOTERM_CC_DIRECT**	GO:0005737~cytoplasm	16	29	53.333	Q9CPX6, P34884, Q9D7A8, Q05BC3, Q9CQ60, Q8R071, Q9WV34, P70318, Q8R570, P14131, P16054, Q9QUM9, P0DP27, P60761, Q60829, Q91XU3	7357	21179	1.588278581	0.031379973	0.974434982	0.451087105	0.443242112
**GOTERM_CC_DIRECT**	GO:0005829~cytosol	11	29	36.667	P14131, P09813, P16054, Q9Z268, Q9CPX6, P34884, Q9QUM9, Q9D7A8, Q05BC3, Q91XU3, Q91XL9	4193	21179	1.915910754	0.036580125	0.986234431	0.46741271	0.459283793
**GOTERM_MF_DIRECT**	GO:0019825~oxygen binding	2	28	6.667	P02088, P01942	26	18713	51.40934066	0.036869377	0.992978252	1	1
**GOTERM_BP_DIRECT**	GO:0042744~hydrogen peroxide catabolic process	2	29	6.667	P02088, P01942	29	20094	47.78596908	0.03965864	0.999997422	1	1
**KEGG_PATHWAY**	mmu01100:Metabolic pathways	8	20	26.667	Q8BK30, Q921H8, P12787, P34884, Q9CQ60, P70699, Q8R071, Q91XU3	1620	8992	2.220246914	0.041092213	0.97709575	1	1
**GOTERM_BP_DIRECT**	GO:0048821~erythrocyte development	2	29	6.667	P02088, P01942	32	20094	43.30603448	0.0436736	0.99999932	1	1
**KEGG_PATHWAY**	mmu05010:Alzheimer disease	4	20	13.333	Q8BK30, P12787, P0DP27, Q9QUM9	383	8992	4.695561358	0.044762284	0.983780721	1	1
**GOTERM_BP_DIRECT**	GO:0043278~response to morphine	2	29	6.667	P16054, Q60829	34	20094	40.75862069	0.046341242	0.99999972	1	1
**GOTERM_MF_DIRECT**	GO:0004601~peroxidase activity	2	28	6.667	P02088, P01942	40	18713	33.41607143	0.05617629	0.999515162	1	1
**GOTERM_BP_DIRECT**	GO:0001975~response to amphetamine	2	29	6.667	P0DP27, Q60829	42	20094	32.99507389	0.056940234	0.999999992	1	1
**GOTERM_CC_DIRECT**	GO:0030017~sarcomere	2	29	6.667	P0DP27, Q9QUM9	46	21179	31.75262369	0.059101473	0.99909339	0.656928143	0.645503305
**GOTERM_BP_DIRECT**	GO:0010811~positive regulation of cell-substrate adhesion	2	29	6.667	P16054, P11627	46	20094	30.12593703	0.062197043	0.999999999	1	1
**GOTERM_CC_DIRECT**	GO:0022627~cytosolic small ribosomal subunit	2	29	6.667	P14131, P01942	49	21179	29.8085855	0.062836605	0.999426199	0.656928143	0.645503305
**UP_KW_DOMAIN**	KW-0677~Repeat	9	17	30.000	P70318, Q8R570, P16054, P11627, Q9Z268, P0DP27, Q05BC3, Q9WV34, Q91XL9	4487	15344	1.810406534	0.064858862	0.521756818	0.600807656	0.600807656
**GOTERM_BP_DIRECT**	GO:0030073~insulin secretion	2	29	6.667	P16054, Q0VGU4	49	20094	28.28149191	0.066121095	1	1	1
**GOTERM_MF_DIRECT**	GO:0015485~cholesterol binding	2	28	6.667	P09813, Q91XL9	49	18713	27.27842566	0.068390487	0.999913129	1	1
**GOTERM_BP_DIRECT**	GO:0046854~phosphatidylinositol phosphorylation	2	29	6.667	Q8R071, Q91XU3	52	20094	26.64986737	0.070029312	1	1	1
**GOTERM_MF_DIRECT**	GO:0044877~macromolecular complex binding	4	28	13.333	P02088, Q9JKD3, P01942, P34884	677	18713	3.948723359	0.072514584	0.999951634	1	1
**KEGG_PATHWAY**	mmu05022:Pathways of neurodegeneration - multiple diseases	4	20	13.333	Q8BK30, P12787, P0DP27, Q9QUM9	471	8992	3.818259023	0.074167855	0.99902747	1	1
**GOTERM_BP_DIRECT**	GO:0048167~regulation of synaptic plasticity	2	29	6.667	Q0VGU4, Q8R071	58	20094	23.89298454	0.077798485	1	1	1
**UP_SEQ_FEATURE**	METAL:Calcium 4	2	28	6.667	Q9Z268, P0DP27	68	22511	23.6460084	0.07848004	0.999982476	1	1
**KEGG_PATHWAY**	mmu05143:African trypanosomiasis	2	20	6.667	P02088, P01942	39	8992	23.05641026	0.079343947	0.999412826	1	1
**GOTERM_CC_DIRECT**	GO:0014069~postsynaptic density	3	29	10.000	Q8R570, P60761, Q9WV34	363	21179	6.035622685	0.082713359	0.999951238	0.792669692	0.778884132
**UP_KW_CELLULAR_COMPONENT**	KW-0206~Cytoskeleton	5	25	16.667	P16054, P0DP27, Q05BC3, Q8R071, Q9WV34	1271	17960	2.826121164	0.085538188	0.860156388	0.940920063	0.940920063
**GOTERM_CC_DIRECT**	GO:0005739~mitochondrion	6	29	20.000	Q8BK30, Q921H8, P16054, P12787, Q9D7A8, Q5U458	1880	21179	2.330777696	0.097225063	0.999992208	0.860067865	0.845110163

Table S4. Gene markers used for cell type annotation in snRNA-seq analysis.

Cell type	Genes
Neuron	Syt1, Snap25, Grin1
Excitatory Neuron	Slc17a7, Camk2a, Nrgn
Inhibitory Neuron	Gad1, Gad2
Astrocyte	Aqp4, Gfap
Endothelial cell	Flt1, Cldn5
Microglia	Cd74, Csf1r, C3
Oligodendrocyte	Mbp, Mobp, Plp1
Oligodendrocyte Progenitor Cells	Pdgfra, Vcan, Cspg4

Table S5. COMPASS results ranking subsystems by the corresponding reaction counts in 5XFAD brains treated with engineered EVs (PE^{high}-EVs *vs* . PE^{low}-EV

No.	Subsystems	NumberOfReactions
1	Transport, extracellular	2007
2	Exchange/demand reaction	1279
3	Fatty acid oxidation	1226
4	Eicosanoid metabolism	445
5	Transport, mitochondrial	426
6	Nucleotide interconversion	271
7	Transport, endoplasmic reticular	270
8	Transport, peroxisomal	210
9	Unassigned	182
10	Bile acid synthesis	177
11	Tyrosine metabolism	173
12	Transport, lysosomal	151
13	Transport, golgi apparatus	150
14	Fatty acid synthesis	147
15	Miscellaneous	133
16	Transport, nuclear	125
17	Vitamin A metabolism	112
18	N-glycan synthesis	110
19	Arachidonic acid metabolism	108
20	Androgen and estrogen synthesis and metabolism	98
21	Tryptophan metabolism	98
22	Steroid metabolism	95
23	Urea cycle	93
24	Keratan sulfate degradation	92
25	Phosphatidylinositol phosphate metabolism	84
26	Sphingolipid metabolism	83
27	Cholesterol metabolism	81
28	Glycerophospholipid metabolism	81
29	Valine, leucine, and isoleucine metabolism	67
30	Folate metabolism	66
31	Inositol phosphate metabolism	64
32	Pentose phosphate pathway	62
33	Keratan sulfate synthesis	59
34	Chondroitin sulfate degradation	55
35	Methionine and cysteine metabolism	53
36	Glycolysis/gluconeogenesis	52
37	Purine catabolism	52
38	Glycine, serine, alanine and threonine metabolism	51
39	Arginine and Proline Metabolism	49
40	Pyrimidine catabolism	47
41	Blood group synthesis	46
42	Chondroitin synthesis	45
43	Starch and sucrose metabolism	42
44	Fructose and mannose metabolism	37
45	Pyruvate metabolism	36
46	Aminosugar metabolism	35
47	Linoleate metabolism	35
48	Heparan sulfate degradation	33
49	Vitamin D metabolism	33
50	Citric acid cycle	32

No.	Subsystems	NumberOfReactions
51	Pyrimidine synthesis	32
52	Vitamin E metabolism	32
53	NAD metabolism	28
54	Tetrahydrobiopterin metabolism	28
55	Lysine metabolism	26
56	Alanine and aspartate metabolism	23
57	Glutamate metabolism	22
58	Glutathione metabolism	21
59	CoA synthesis	20
60	Histidine metabolism	20
61	Purine synthesis	20
62	Vitamin C metabolism	20
63	Galactose metabolism	18
64	Selenoamino acid metabolism	18
65	Propanoate metabolism	17
66	Glyoxylate and dicarboxylate metabolism	16
67	N-glycan degradation	16
68	Cytochrome metabolism	15
69	O-glycan synthesis	15
70	C5-branched dibasic acid metabolism	14
71	Heme synthesis	14
72	Ubiquinone synthesis	14
73	beta-Alanine metabolism	13
74	Glycosphingolipid metabolism	13
75	Limonene and pinene degradation	13
76	Phenylalanine metabolism	13
77	Triacylglycerol synthesis	13
78	Vitamin B6 metabolism	13
79	Biotin metabolism	12
80	Dietary fiber binding	12
81	Taurine and hypotaurine metabolism	11
82	CoA catabolism	8
83	Oxidative phosphorylation	8
84	Vitamin B2 metabolism	8
85	ROS detoxification	7
86	Squalene and cholesterol synthesis	7
87	Alkaloid synthesis	6
88	Thiamine metabolism	6
89	Butanoate metabolism	5
90	Hyaluronan metabolism	5
91	Nucleotide sugar metabolism	5
92	Cysteine Metabolism	4
93	D-alanine metabolism	4
94	Lipoate metabolism	4
95	Vitamin B12 metabolism	4
96	Nucleotide salvage pathway	3
97	R group synthesis	3
98	Heme degradation	2
99	Stilbene, coumarine and lignin synthesis	2

Table S6. Key resources table, related to Methods.

	REAGENT or RESOURCE	SOURCE	IDENTIFIER
Antibodies and Probes	Guinea pig polyclonal anti-Perilipin-2	CiteAb	Cat# 20R-AP002 RRID: AB_1282475
	Rabbit polyclonal anti-β-Amyloid	Abcam	Cat# ab2539 RRID: AB_303141
	Mouse monoclonal anti-β-Amyloid, 1-40	Biolegend	Cat# SIG-39140 RRID: AB_10175638
	Mouse monoclonal anti-β-Amyloid (21F12), 1-42	Absolute antibody	Cat# Ab02391-3.0
	Rabbit polyclonal anti-β-Amyloid (21F12), 1-42	Absolute antibody	Cat# Ab02391-23.0
	Mouse monoclonal anti-β-Actin	Signalway	Cat# 21800
	Rabbit monoclonal anti-APP (6E10)	Novus Biologicals	Cat# NBP2-62566 RRID: AB_2917960
	Mouse monoclonal anti-tau (BT2)	Invitrogen	Cat# MN1010 RRID: AB_10975238
	Mouse monoclonal anti-p-tau (Thr181) (AT270)	Invitrogen	Cat# MN1050 RRID: AB_223651
	Mouse monoclonal anti-p-tau (Ser202 Thr205) (A	Invitrogen	Cat# MN1020 RRID: AB_223647
	Rabbit monoclonal anti-p-tau (Ser396)	Abcam	Cat# ab156623
	Mouse monoclonal anti-β-III Tubulin	Santa Cruz Biotechnology	Cat# sc-80005 RRID: AB_2210816
	Streptavidin, Alexa Fluor 647™ conjugate	Thermo Fisher Scientific	Cat# S21374 RRID: AB_2336066
	Duramycin-LC-Bioton	Polysciences	Cat# 25690-100
	Rabbit polyclonal anti-NFAT1 (D43B1)	Cell Signaling	Cat# 5861
	Oregon Green™ 488 BAPTA-1, AM	Thermo Fisher Scientific	Cat# O6807
	ER-Tracker™ Red (BODIPY™ TR Glibenclamide	Thermo Fisher Scientific	Cat# E34250
	NeuN Polyclonal Antibody	Thermo Fisher Scientific	Cat# PA5-143567
	NFkB p65 Polyclonal Antibody	Thermo Fisher Scientific	Cat# 51-0500
	SREBP1 Polyclonal Antibody	Thermo Fisher Scientific	Cat# PA1-337
	Mouse monoclonal anti-APP (22C11)	Thermo Fisher Scientific	Cat# 14-9749-82
	Rabbit polyclonal anti-BACE1	Thermo Fisher Scientific	Cat# PA1-757
	Goat polyclonal anti-RAB5	antibodies.com	Cat# A121672
Chemicals, peptides, and recombinant proteins	Ethanolamine (Etn)	Sigma-Aldrich	Cat# E9508-25 ML
	Ebsulfur	Medchemexpress	Cat# HY-128170
	Choline	Sigma-Aldrich	Cat#7017
	Ebselen	Sigma-Aldrich	Cat# E3520-25 MG
	Ivermectin	Sigma-Aldrich	Cat# I8898
	MG 624	Sigma-Aldrich	Cat# M3184-5 MG
	Ro 90-7501	Tocris	Cat# 2408
	Pentamidine isethionate salt	Sigma-Aldrich	Cat# P0547-250MG
	CP-339818	Sigma-Aldrich	Cat# C2499-5MG
	Leflunomide	Sigma-Aldrich	Cat# L5025
	Retinoic acid	Sigma-Aldrich	Cat# R2625
	Tegaserod maleate	Sigma-Aldrich	Cat# 189188-57-6
	Methylene Blue	Sigma-Aldrich	Cat# M9140
	Stem Cell Dissociation Reagent	ATCC	Cat# ACS-3010
	Cell Basement Membrane	ATCC	Cat# ACS-3035
	DMEM/F12 medium	Gibco/Life	Cat# 11320-033
	RPMI Medium 1640	Technologies	Cat# 11875-119
	DMEM (high glucose)	Gibco	Cat# CM002-050
	HEPES Buffer Solution (1 M)	GenDEPOT	Cat# CA011-010
	Penicillin/Streptomycin/Amphotericin B	Lonza	Cat# 17-745E
	Dulbecco's Phosphate Buffered Saline	Lonza	Cat# 17-512Q
	PBS pH 7.2	Gibco	Cat# 20012-027
	ReNcell Neural Stem Cell Freezing Medium	EMD Millipore	Cat# SCM007
	Matrigel	Corning	Cat# 356230
	Bovine Serum Albumin	Sigma-Aldrich	Cat# A2153
	DAPI	Cellomics Technology	Cat# 1860586
	SuperScript® VILO™ Master Mix	Invitrogen	Cat# 11755050
	Itaq™ Universal SYBR	Bio-Rad	Cat# 1725121
	Normal Donkey Serum	Jackson	Cat# 017-000-121
	LipidTOX™ Deep Red	Thermo Fisher Scientific	Cat# H34477
	BODIPY™ 558/568-C_{12}	Thermo Fisher Scientific	Cat# D3835
	ETNK2 recombinant protein	Mybiosource	Cat# MBS140838
	Hydrogen peroxide solution, 30 % (w/w) in H_2O	Sigma-Aldrich	Cat# H1009
	BAY 11-7082	Sigma-Aldrich	Cat# 196870
	Fatostatin	Sigma-Aldrich	Cat# AMBH6FD841CF
	DiIC18(3)	Invitrogen	Cat# #D3911
	Digitonin	Sigma-Aldrich	Cat# D141

	holo-Transferrin	Sigma-Aldrich	Cat#T0665
Critical commercial assays	Mouse IFN-γ ELISA Kit, Extra sensitive	Invitrogen	Cat# BMS609
	Mouse TNF-α ELISA Kit, High sensitive	Invitrogen	Cat# BMS607HS
	Mouse Granzyme B ELISA Kit	Invitrogen	Cat# BMS6029
	Phosphatidylethanolamine Assay Kit	Abcam	Cat# 241005
	Pierce™ BCA Protein Assay Kit	ThermoFisher	Cat# 23225
	ADP-Glo™ Kinase Assay kit	Promega	Cat# V6930
	SimpleChIP® Enzymatic Chromatin IP Kit	Cell Signaling Technology	Cat# 9002
	RNeasy Plus Mini Kit	QIAGEN	Cat# 74134
	In Situ Cell Death Detection Kit, Fluorescein	Roche	Cat# 11684795910
	Chromium Next GEM Single Cell 3' Kit v3.1	10X Genomics	Cat#1000269
	Chromium Nuclei Isolation with Rnase Inhibitor K	10X Genomics	Cat#1000494
	Dual Index Kit TT Set A 96 rxns	10X Genomics	Cat#1000215
	Chromium Next GEM Chip G Single Cell Kit	10X Genomics	Cat#1000127
Experimental model: Cell lines	DO-11-10 Cells	Dr. Xiang H.-F. Zhang (BCM)	N/A
	VM Human Neural Stem Cell Lines	EMD Millipore	Cat# SCC008
	HEK293-APP695-WT cell line	KYINNO BIOTECHNOLOGY	Cat#KC-0146
	Human peripheral blood CD8+ T cells, Frozen	STEMCELL	Cat#70027
Experimental Models: - Organis	Mouse: C57BL/6J	The Jackson Laboratory	Strain # 000664
	Mouse: 3xTg	The Jackson Laboratory	Strain # 004807
	Mouse: 5XFAD	The Jackson Laboratory	MMRRC Strain # 034848
Software and algorithms	FlowJo	BD Biosciences	RRID:SCR_008520
	GraphPad Prism 9	GraphPad	http://www.graphpad.com
	Adobe Illustrator	Adobe®	http://adobe.com
	Fiji (Image J)	Zulu OpenJDK	http://imagej.nih.gov/ij/
	RAWGraphs 2.0	Open source	http://app.rawgraphs.io
	MetaboAnalyst 5.0	NIH – Canada Research Chair	http://metaboanalyst.ca/
	UALCAN	PMID: 35078134	http://ualcan.path.uab.edu/
	Agora	National Institute on Aging	http://agora.adknowledgeportal.org
	GEPIA2	Zhang Lab	http://gepia2.cancer-pku.cn/
	NeuriteIQ	PMID: 22277654	N/A

Table S7. RT-qPCR and ChIP-qPCR primers, related to quantitative PCR (qPCR) part in Methods.

Gene	Sequence	Source
m*Etnk1* (*Eki1*) -FWD	GCTGTTCACAGATGGGATCACAAAC	Modified from PMID: 34234346
m*Etnk1* (*Eki1*) -RVS	TTGCCGTAAATCCTCACCAGAACTAC	Modified from PMID: 34234346
m*Pcyt2* -FWD	TGGTGCGATGGCTGCTATG	PMID: 34234346
m*Pcyt2* -RVS	CCCCTTATGCTTGGCAATCTCC	PMID: 34234346
m*Pcyt1a* -FWD	TGGATGCACAGAGTTCAGCTAAAGTC	Modified from PMID: 34234346
m*Pcyt1a* -RVS	TGGCTGCCGTAAACCAACTG	PMID: 34234346
m*Selenoi* (*Ept1*) -FWD	TCCTACTCCTGACATACTTCGACCC	Modified from PMID: 34234346
m*Selenoi* (*Ept1*) -RVS	CCACGACAATCCAAACCCAG	PMID: 34234346
m*Etnk2* -FWD	GCTTCAGCGTCACGGTGG	This paper*
m*Etnk2* -RVS	AATAGCTTGTTGGTGATGCCGTC	This paper*
m*Cept1* -FWD	GCTGGCAGTGATTGGAGGAC	This paper*
m*Cept1*-RVS	CACCACCTGTGAAGATTACACGG	This paper*
m*Pdcd1* (*Pd-1 Cd279*) -FWD	ACCCTGGTCATTCACTTGGG	PMID: 34234346
m*Pdcd1* (*Pd-1 Cd279*) -RVS	CATTTGCTCCCTCTGACACTG	PMID: 34234346
m*Cxcr3* (*Cd183*) -FWD	GGCTAGCTCGAACTTACCCGTAAC	This paper*
m*Cxcr3* (*Cd183*) -RVS	GCAGCTAAGGCACAGTAATGGTG	This paper*
m*Cxcr5* -FWD	ATGAACTACCCACTAACCCTGG	PMID: 34234346
m*Cxcr5* -RVS	TGTAGGGGAATCTCCGTGCT	PMID: 34234346
m*Lag-3* (*Cd223*) -FWD	GTCTCCATCACGTACAACCTCAAGG	This paper*
m*Lag-3* (*Cd223*) -RVS	GGAGTCCACTTGGCAATGAGC	This paper*
m*Ly108* (*Cd352*) -FWD	AAGCGTGCTTTCCAACTGGC	Origene
m*Ly108* (*Cd352*) -RVS	GCAGCATGTGGATGAGTTACCC	Origene
m*Rgs16* -FWD	ATCCGATCAGCCACCAAACTG	This paper*
m*Rgs16* -RVS	GAAGCAACTGGTAGTGGCAGCTTC	This paper*
m*Tigit* -FWD	CGGAGCCACAGGAATGGAAC	This paper*
m*Tigit* -RVS	CCTGTGGGTCAGCATAGTCATCTT	This paper*
m*Tim-3* (*Cd366*) -FWD	CCGCCTCTGGACTGCCAC	This paper*
m*Tim-3* (*Cd366*) -RVS	TGAGGTTGCCAAGTGACATATCACATAG	This paper*
m*Nfat1* -FWD	ACTTCACAGCGGAGTCCAAGGT	Origene
m*Nfat1* -RVS	GGATGTGCTTGTTCCGATACTCG	Origene
m*Nfat2* -FWD	GGTGCCTTTTGCGAGCAGTATC	Origene
m*Nfat2* -RVS	CGTATGGACCAGAATGTGACGG	Origene
m*Nr4a1* -FWD	GTGCAGTCTGTGGTGACAATGC	Origene
m*Nr4a1* -RVS	CAGGCAGATGTACTTGGCGCTT	Origene
m*Nr4a2* -FWD	CGCCGAAATCGTTGTCAGTACTG	This paper*
m*Nr4a2* -RVS	TCGGCTTCGAGGGTAAACGAC	This paper*
m*Nr4a3* -FWD	CCGAAACCGATGTCAGTACTGC	This paper*
m*Nr4a3* -RVS	CTCCTGTTGTAGTGGGCTCTTTGG	This paper*
m*Tox* -FWD	AAGATGGCGCACTGCTCTCC	This paper*
m*Tox* -RVS	ATGCTTGCCTGCTGTCTGATG	This paper*
m*Tox2* -FWD	GAAGCTGCCAAGAAGGAGTACCTG	This paper*
m*Tox2* -RVS	GGTTTGCCTGAGTGTTCTTGGC	This paper*
m*Tnfα* -FWD	GAGAAAGTCAACCTCCTCTCTG	This paper*
m*Tnfα* -RVS	GAAGACTCCTCCCAGGTATATG	This paper*
m*Gzmb* (*Ctla-1*) -FWD	GATTGCTCTAGGACAGGTGGCA	This paper*
m*Gzmb* (*Ctla-1*) -RVS	GTTGTCACAGCATGGTGGCAG	This paper*
mIfnγ -FWD	AAGACAATCAGGCCATCAGCAAC	This paper*
mIfnγ -RVS	CCTGTGGGTTGTTGACCTCAAAC	This paper*
m*Atg3* -FWD	ACACGGTGAAGGGAAAGGC	PMID: 25383539
m*Atg3* -RVS	TGGTGGACTAAGTGATCTCCAG	PMID: 25383539
m*Atg4a* -FWD	GCTGGTATGGATTCTGGGGAA	PMID: 25383539
m*Atg4a* -RVS	TGGGTTGTTCTTTTTGTCTCTCC	PMID: 25383539
m*Atg4b* -FWD	CATCCATCAGATAGCGCAAA	PMID: 25383539
m*Atg4b* -RVS	TGATTTCCTCCATCACCACA	PMID: 25383539
m*Atg4c* -FWD	AGATGAAAGCAAGATGTTGCCT	PMID: 25383539
m*Atg4c* -RVS	CCCTGTAGGTCAGCCATATTCTA	PMID: 25383539
m*Atg4d* -FWD	GTCAAGTATGGTTGGGCAGTT	PMID: 25383539
m*Atg4d* -RVS	TGTCACCCTCTCCCTCGAAAT	PMID: 25383539
m*Atg7* -FWD	TGCCTATGATGATCTGTGTC	PMID: 25383539
m*Atg7* -RVS	CACCAACTGTTATCTTTGTCC	PMID: 25383539
m*H2-d1* -FWD	GCTCTGAAATGTCTCTCCGAGATTG	This paper*
m*H2-d1* -RVS	ACAGGGAACATCAGACAATGTTGTG	This paper*
m*H2-k1* -FWD	CAGGCTGGTGAAGCAGAGAGAC	This paper*
m*H2-k1* -RVS	TTCAGGTCTGCTGTGATGGG	This paper*
m*Tbx21* -FWD	GGAGCCCACAAGCCATTACAG	This paper*
m*Tbx21* -RVS	GACATATAAGCGGTTCCCTGGC	This paper*
m*Gata3* -FWD	TGCGTGAGGAGTCTCCAAGTG	This paper*
m*Gata3* -RVS	ATGGGATCCGGATTCAGTGGT	This paper*
m*Fox3* -FWD	TCGCCTACTTCAGAAACCACCC	This paper*
m*Fox3* -RVS	CAAATTCATCTACGGTCCACACTGC	This paper*
m*IL6*-FWD	TCTATACCACTTCACAAGTCGGAGG	This paper
m*IL6*-RVS	CTGCAAGTGCATCATCGTTGTTC	This paper
m*Nrf1*-FWD	GGAGCTCTATCATGGCAGCG	This paper
m*Nrf1*-RVS	ACCAGATCCCAGAGTGGTGC	This paper

m*Lef1*-FWD	GCCACCGATGAGATGATCCC	This paper
m*Lef1*-RVS	TTGATGTCGGCTAAGTCGCC	This paper
m*Tcf7*-FWD	CAATCTGCTCATGCCCTACC	This paper
m*Tcf7*-RVS	CTTGCTTCTGGCTGATGTCC	This paper
m*Bcl6*-FWD	TGTGTAAGGCAAACCCTGTCAGC	This paper
m*Bcl6*-RVS	GCAACCTGTCAAGTCAGAACTTCTGC	This paper
m*Il7r*-FWD	GCCCATCTCCACTTCCTCAGTACTG	This paper
m*Il7r*-RVS	CATTTGGTTGTCGATGGAAGGG	This paper
m*36b4* -FWD	GAAACTGCTGCCTCACATCCG	This paper
m*36b4* -RVS	GCTGGCACAGTGACCTCACAC	This paper

The primers with “*” means that the primers were modified from Origene.

ChIP-qPCR primers

Gene	Sequence
h*SREBF1* F1	GCGTTTAATTAAGGCCAAGG
h*SREBF1* R1	GTTTTCAGGAGTGGCCTCAT
h*SREBF1* F2	AGCCCAGGCCCTTATTTCC
h*SREBF1* R2	AGTGTGCTTTTGGGTCAAGGA
h*ETNK* F	GACCGGAGGCGAGAAA
h*ETNK* R	TCTCGGAGAAAATTCCTGTT
