# Supplementary material for: Obesity-driven phosphatidylethanolamine dysregulation impairs neuroimmune crosstalk and accelerates Alzheimer’s pathogenesis
Source: Mol Neurodegener. 2026 Apr 15;21:25. doi: 10.1186/s13024-026-00943-3 (PMC13159212; doi:10.1186/s13024-026-00943-3)
Supplement: Supplementary file 2 — Supplementary Material 2 [file 13024_2026_943_MOESM2_ESM.pdf]

Figure S1

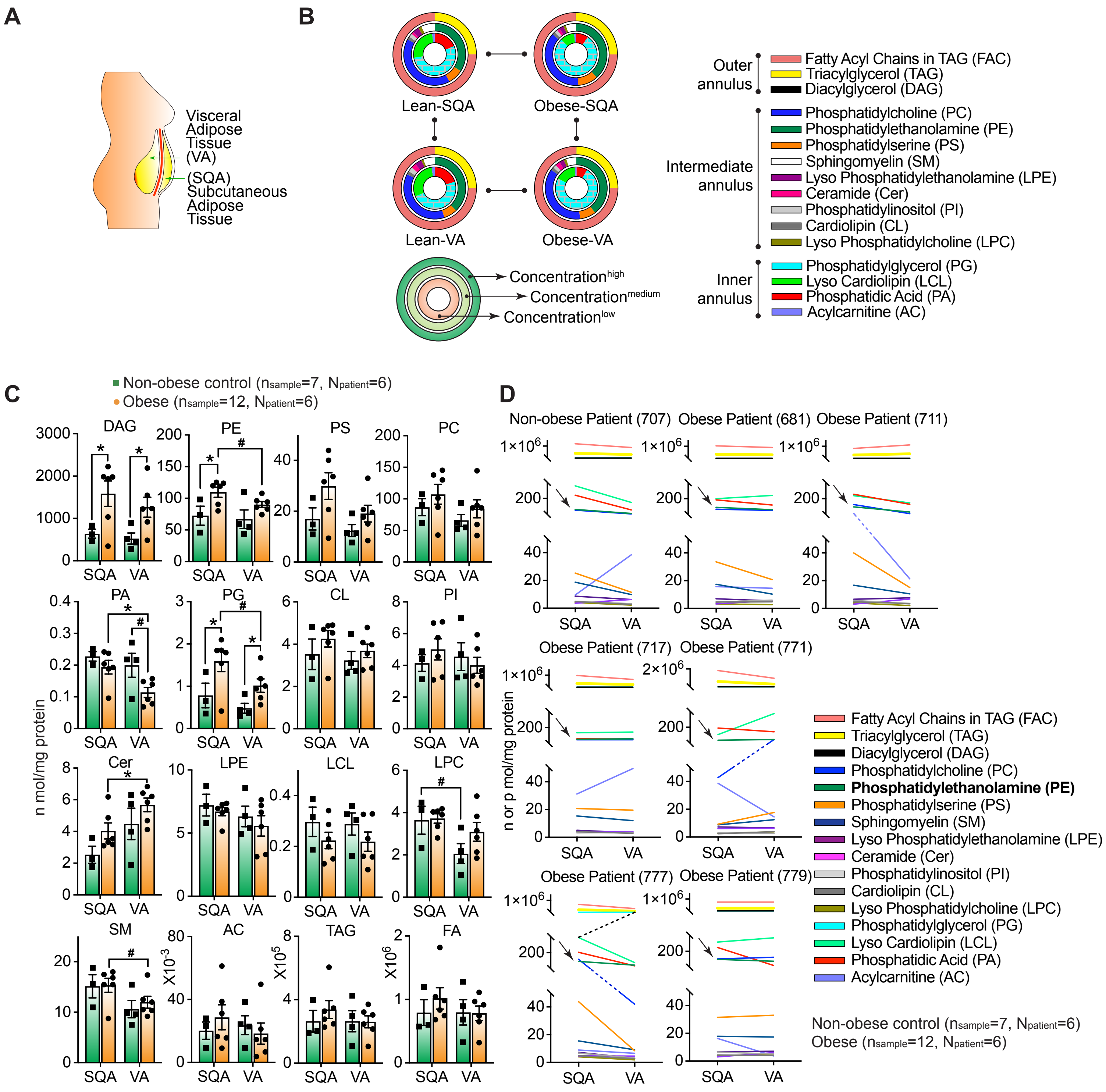

A
Visceral Adipose Tissue (VA)
(SQA) Subcutaneous Adipose Tissue
B
Lean-SQA
Obese-SQA
Lean-VA
Obese-VA
Concentration^high
Concentration^medium
Concentration^low
Outer annulus
Fatty Acyl Chains in TAG (FAC)
Triacylglycerol (TAG)
Diacylglycerol (DAG)
Intermediate annulus
Phosphatidylcholine (PC)
Phosphatidylethanolamine (PE)
Phosphatidylserine (PS)
Sphingomyelin (SM)
Lyso Phosphatidylethanolamine (LPE)
Ceramide (Cer)
Phosphatidylinositol (PI)
Cardiolipin (CL)
Lyso Phosphatidylcholine (LPC)
Inner annulus
Phosphatidylglycerol (PG)
Lyso Cardiolipin (LCL)
Phosphatidic Acid (PA)
Acylcarnitine (AC)
C
Non-obese control (nsample=7, Npatient=6)
Obese (nsample=12, Npatient=6)
n mol/mg protein
DAG
PE
PS
PC
PA
PG
CL
PI
Cer
LPE
LCL
LPC
SM
AC
TAG
FA
X10^-3
X10^5
X10^6
SQA VA
D
Non-obese Patient (707)
Obese Patient (681)
Obese Patient (711)
Obese Patient (717)
Obese Patient (771)
Obese Patient (777)
Obese Patient (779)
n or p mol/mg protein
SQA VA
Fatty Acyl Chains in TAG (FAC)
Triacylglycerol (TAG)
Diacylglycerol (DAG)
Phosphatidylcholine (PC)
Phosphatidylethanolamine (PE)
Phosphatidylserine (PS)
Sphingomyelin (SM)
Lyso Phosphatidylethanolamine (LPE)
Ceramide (Cer)
Phosphatidylinositol (PI)
Cardiolipin (CL)
Lyso Phosphatidylcholine (LPC)
Phosphatidylglycerol (PG)
Lyso Cardiolipin (LCL)
Phosphatidic Acid (PA)
Acylcarnitine (AC)
Non-obese control (nsample=7, Npatient=6)
Obese (nsample=12, Npatient=6)

Figure S2

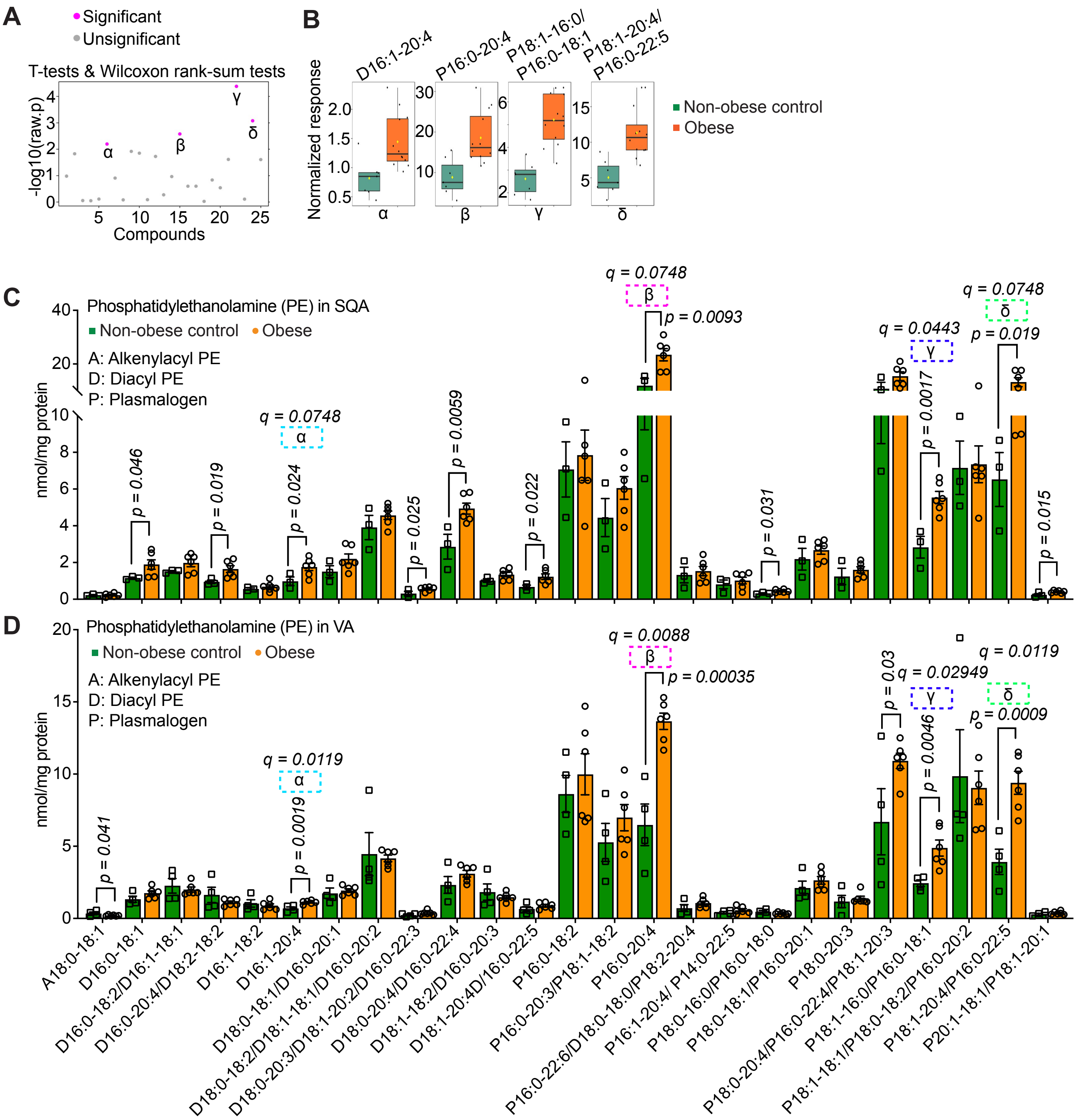

A
Significant
Unsignificant
T-tests & Wilcoxon rank-sum tests
-log10(raw.p)
Compounds
B
D16:1-20:4
P16:0-20:4
P18:1-16:0/ P16:0-18:1
P18:1-20:4/ P16:0-22:5
Normalized response
Non-obese control
Obese
C
Phosphatidylethanolamine (PE) in SQA
Non-obese control
Obese
A: Alkenylacyl PE
D: Diacyl PE
P: Plasmalogen
nmol/mg protein
q = 0.0748
p = 0.0093
q = 0.0443
p = 0.019
p = 0.0017
p = 0.046
p = 0.019
p = 0.024
p = 0.0059
p = 0.025
p = 0.022
p = 0.031
p = 0.015
D
Phosphatidylethanolamine (PE) in VA
Non-obese control
Obese
A: Alkenylacyl PE
D: Diacyl PE
P: Plasmalogen
nmol/mg protein
q = 0.0088
p = 0.00035
q = 0.0119
p = 0.0019
p = 0.041
p = 0.03
q = 0.02949
p = 0.0046
q = 0.0119
p = 0.0009
A18:0-18:1
D16:0-18:1
D16:0-18:2/D16:1-18:1
D16:0-20:4/D18:2-18:2
D16:1-18:2
D16:1-20:4
D18:0-18:1/D16:0-20:1
D18:0-18:2/D18:1-18:1/D16:0-20:2
D18:0-20:3/D18:1-20:2/D16:0-22:3
D18:0-20:4/D16:0-22:4
D18:1-18:2/D16:0-20:3
D18:1-20:4D/16:0-22:5
P16:0-18:2
P16:0-20:3/P18:1-18:2
P16:0-20:4
P16:0-22:6/D18:0-18:0/P18:2-20:4
P16:1-20:4/ P14:0-22:5
P18:0-16:0/P16:0-18:0
P18:0-18:1/P16:0-20:1
P18:0-20:3
P18:0-20:4/P16:0-22:4/P18:1-20:3
P18:1-16:0/P16:0-18:1
P18:1-18:1/P18:0-18:2/P16:0-20:2
P18:1-20:4/P16:0-22:5
P20:1-18:1/P18:1-20:1

Figure S3

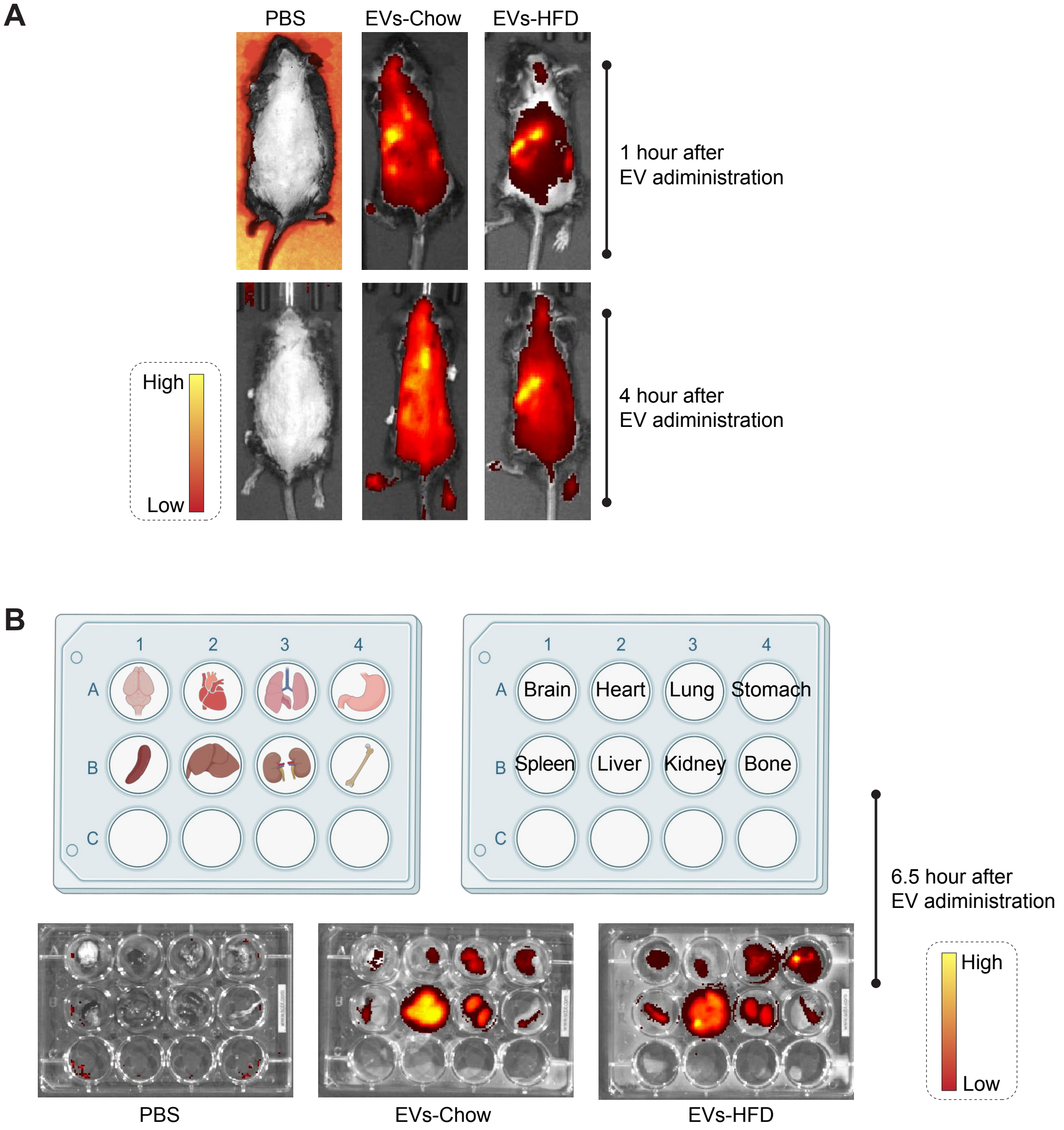
A
PBS
EVs-Chow
EVs-HFD
1 hour after
EV adiministration
4 hour after
EV adiministration
High
Low
B
1
2
3
4
A
B
C
Brain
Heart
Lung
Stomach
Spleen
Liver
Kidney
Bone
6.5 hour after
EV adiministration
PBS
EVs-Chow
EVs-HFD
High
Low

Figure S4

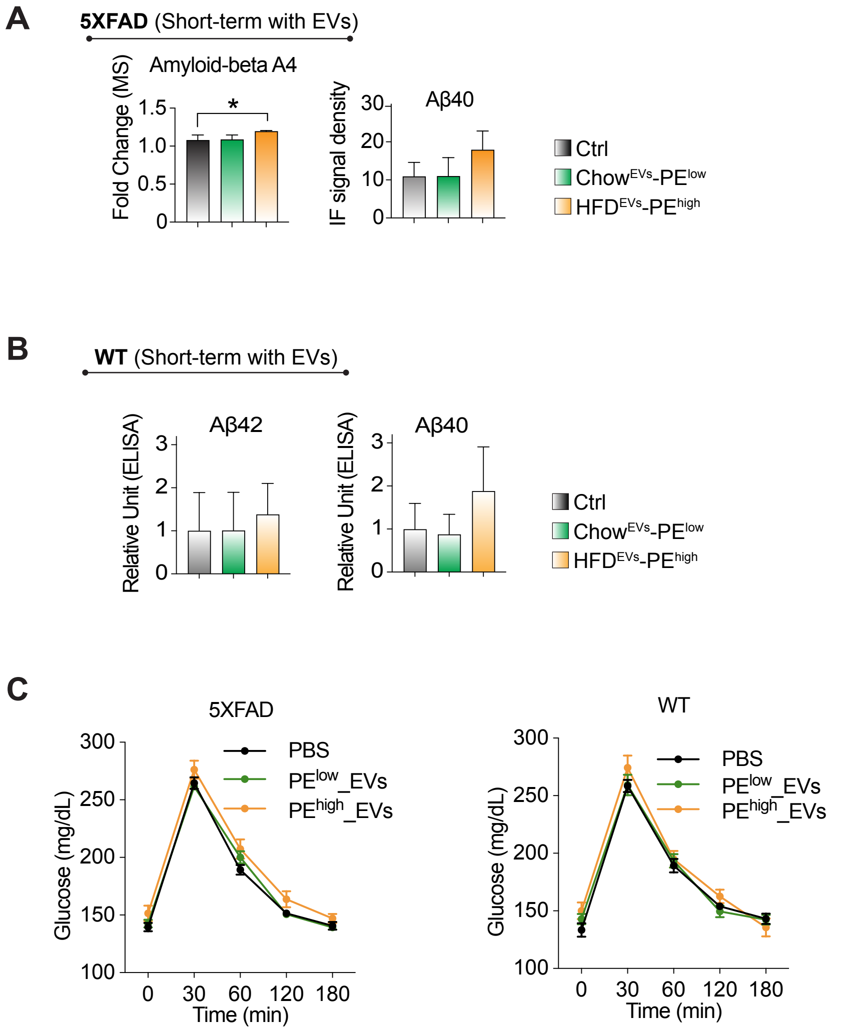

A
5XFAD (Short-term with EVs)
Amyloid-beta A4
Fold Change (MS)
1.5
1.0
0.5
0
*
Aβ40
IF signal density
30
20
10
0
Ctrl
Chow^{EVs}-PE^{low}
HFD^{EVs}-PE^{high}
B
WT (Short-term with EVs)
Aβ42
Relative Unit (ELISA)
3
2
1
0
Aβ40
Relative Unit (ELISA)
Ctrl
Chow^{EVs}-PE^{low}
HFD^{EVs}-PE^{high}
C
5XFAD
Glucose (mg/dL)
300
250
200
150
100
PBS
PE^{low}_EVs
PE^{high}_EVs
0
30
60
120
180
Time (min)
WT
Glucose (mg/dL)
PBS
PE^{low}_EVs
PE^{high}_EVs
Time (min)

Figure S5

A

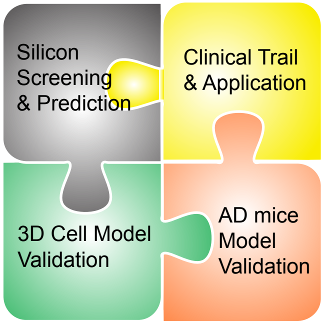

B

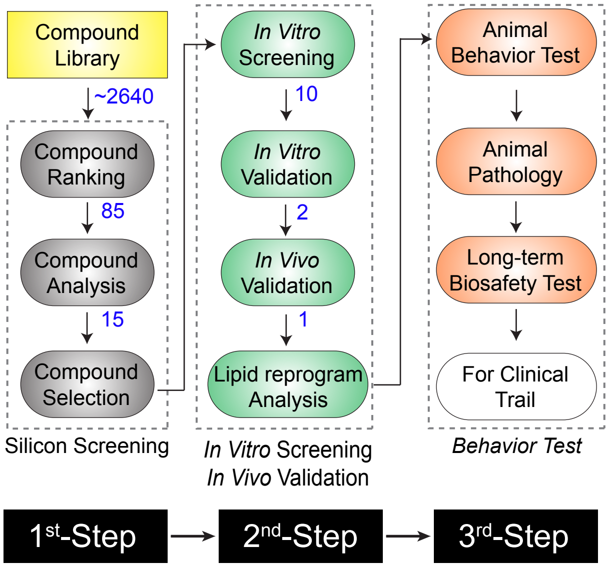

C

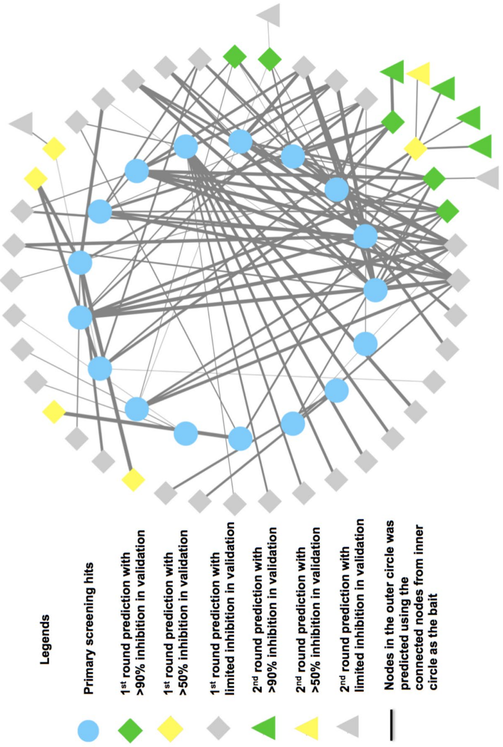

D

Compound	Formula	Function
Ebselen	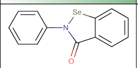	Antioxidant; inhibits mammalian lipoxygenases and glutathione S-transferase
Ivermectin	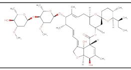	Positive allosteric modulator of α7 neuronal nicotinic acetylcholine receptor;
MG 624	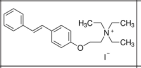	α7 nicotinic acetylcholine receptor antagonist; selectively inhibits
Ro 90-7501	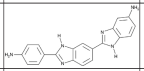	Inhibitor of Aβ42 fibril formation
Pentamidine isethionate	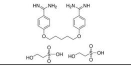	NMDA glutamate receptor antagonist; neuroprotective agent; antimicrobial agent
CP 339818	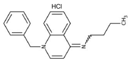	Non-peptide, potent KV3.1 channel blocker

Figure S6

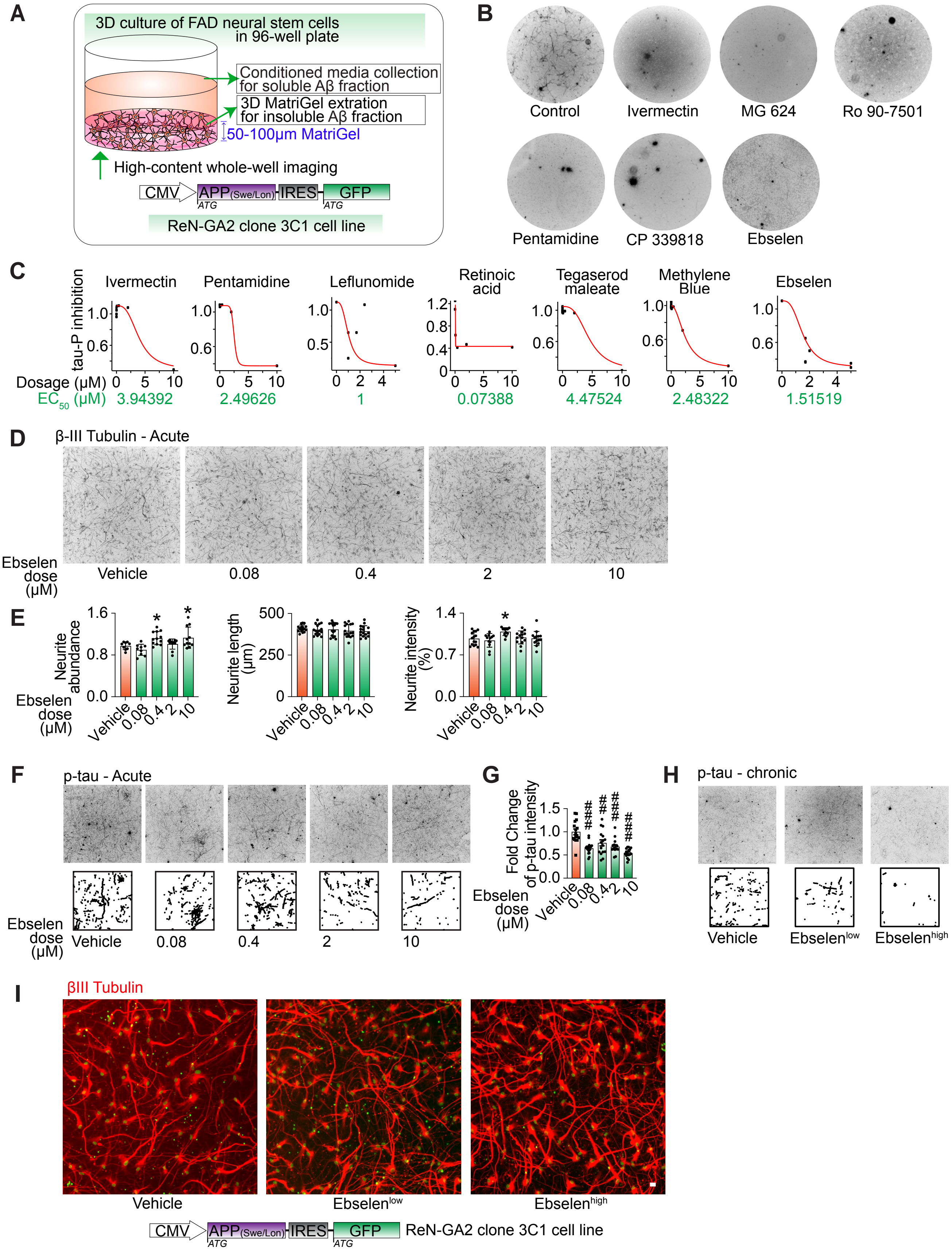

A
3D culture of FAD neural stem cells in 96-well plate
Conditioned media collection for soluble Aβ fraction
3D MatriGel extration for insoluble Aβ fraction
50-100μm MatriGel
High-content whole-well imaging
CMV
APP(Swe/Lon)
IRES
GFP
ATG
ATG
ReN-GA2 clone 3C1 cell line
B
Control
Ivermectin
MG 624
Ro 90-7501
Pentamidine
CP 339818
Ebselen
C
tau-P inhibition
Ivermectin
Pentamidine
Leflunomide
Retinoic acid
Tegaserod maleate
Methylene Blue
Ebselen
Dosage (μM)
EC50 (μM)
3.94392
2.49626
1
0.07388
4.47524
2.48322
1.51519
D
β-III Tubulin - Acute
Ebselen dose (μM)
Vehicle
0.08
0.4
2
10
E
Neurite abundance
Neurite length (μm)
Neurite intensity (%)
Ebselen dose (μM)
F
p-tau - Acute
Ebselen dose (μM)
Vehicle
0.08
0.4
2
10
G
Fold Change of p-tau intensity
Ebselen dose (μM)
H
p-tau - chronic
Vehicle
Ebselenlow
Ebselenhigh
I
βIII Tubulin
Vehicle
Ebselenlow
Ebselenhigh
CMV
APP(Swe/Lon)
IRES
GFP
ATG
ATG
ReN-GA2 clone 3C1 cell line

Figure S7

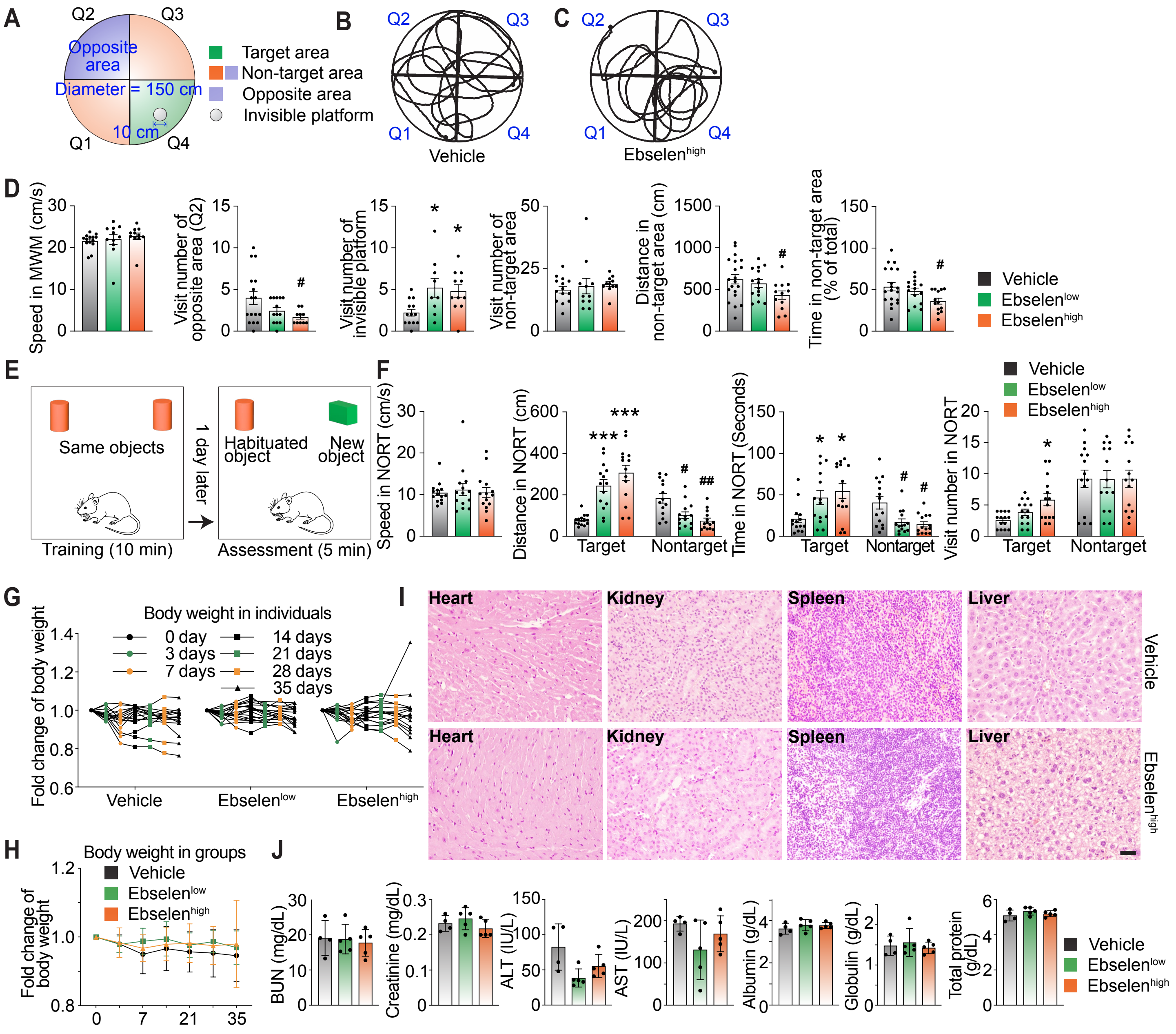

A
Q2
Q3
Opposite area
Diameter = 150 cm
10 cm
Q1
Q4
Target area
Non-target area
Opposite area
Invisible platform
B
Vehicle
C
Ebselenhigh
D
Speed in MWM (cm/s)
Visit number of opposite area (Q2)
Visit number of invisible platform
Visit number of non-target area
Distance in non-target area (cm)
Time in non-target area (% of total)
Vehicle
Ebselenlow
Ebselenhigh
E
Same objects
1 day later
Habituated object
New object
Training (10 min)
Assessment (5 min)
F
Speed in NORT (cm/s)
Distance in NORT (cm)
Time in NORT (Seconds)
Visit number in NORT
Target
Nontarget
G
Body weight in individuals
0 day
3 days
7 days
14 days
21 days
28 days
35 days
Fold change of body weight
I
Heart
Kidney
Spleen
Liver
H
Body weight in groups
J
BUN (mg/dL)
Creatinine (mg/dL)
ALT (IU/L)
AST (IU/L)
Albumin (g/dL)
Globulin (g/dL)
Total protein (g/dL)

Figure S8

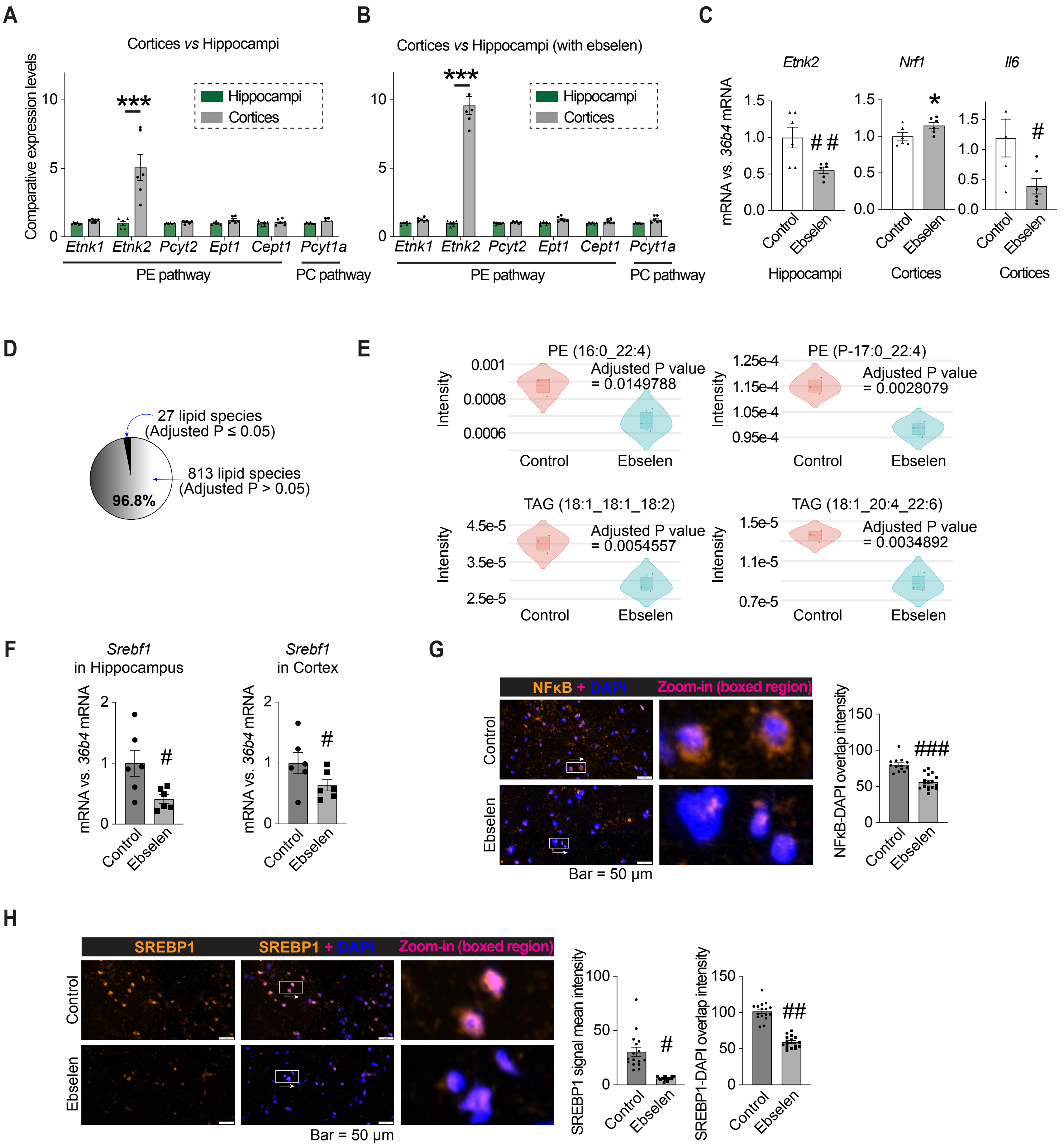

A
Cortices vs Hippocampi
Comparative expression levels
Hippocampi
Cortices
***
Etnk1 Etnk2 Pcyt2 Ept1 Cept1 Pcyt1a
PE pathway
PC pathway
B
Cortices vs Hippocampi (with ebselen)
C
Etnk2
Nrf1
Il6
mRNA vs. 36b4 mRNA
Control
Ebselen
Hippocampi
Cortices
Cortices
D
27 lipid species
(Adjusted P ≤ 0.05)
813 lipid species
(Adjusted P > 0.05)
96.8%
E
PE (16:0_22:4)
Adjusted P value
= 0.0149788
PE (P-17:0_22:4)
Adjusted P value
= 0.0028079
TAG (18:1_18:1_18:2)
Adjusted P value
= 0.0054557
TAG (18:1_20:4_22:6)
Adjusted P value
= 0.0034892
Intensity
Control
Ebselen
F
Srebf1
in Hippocampus
Srebf1
in Cortex
G
NFκB + DAPI
Zoom-in (boxed region)
Bar = 50 μm
NFκB-DAPI overlap intensity
###
H
SREBP1
SREBP1 + DAPI
Zoom-in (boxed region)
Bar = 50 μm
SREBP1 signal mean intensity
SREBP1-DAPI overlap intensity
##

Figure S9

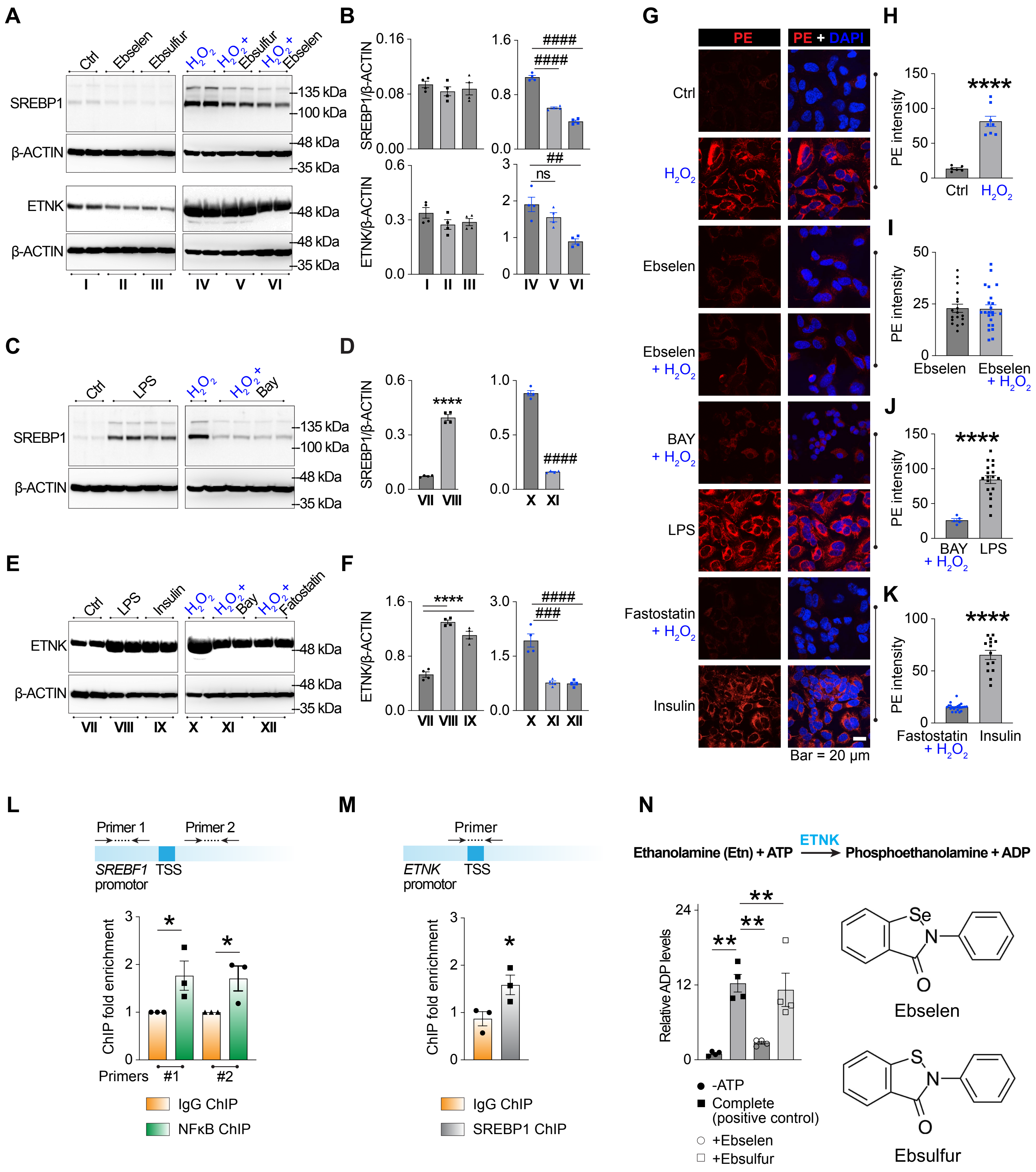

A
Ctrl
Ebselen
Ebsulfur
H2O2
H2O2 + Ebsulfur
H2O2 + Ebselen
SREBP1
β-ACTIN
ETNK
135 kDa
100 kDa
48 kDa
35 kDa
I II III IV V VI
B
SREBP1/β-ACTIN
ETNK/β-ACTIN
####
##
ns
C
Ctrl
LPS
H2O2
H2O2 + Bay
D
****
####
VII VIII X XI
E
Ctrl
LPS
Insulin
H2O2
H2O2 + Bay
H2O2 + Fatostatin
VII VIII IX X XI XII
F
###
G
PE
PE + DAPI
Ctrl
H2O2
Ebselen
Ebselen + H2O2
BAY + H2O2
LPS
Fastostatin + H2O2
Insulin
Bar = 20 μm
H
PE intensity
I
J
K
L
Primer 1
Primer 2
SREBF1 promotor
TSS
ChIP fold enrichment
Primers
#1
#2
IgG ChIP
NFκB ChIP
M
Primer
ETNK promotor
SREBP1 ChIP
N
Ethanolamine (Etn) + ATP
ETNK
Phosphoethanolamine + ADP
Relative ADP levels
-ATP
Complete (positive control)
+Ebselen
+Ebsulfur
Ebselen
Ebsulfur

Figure S10

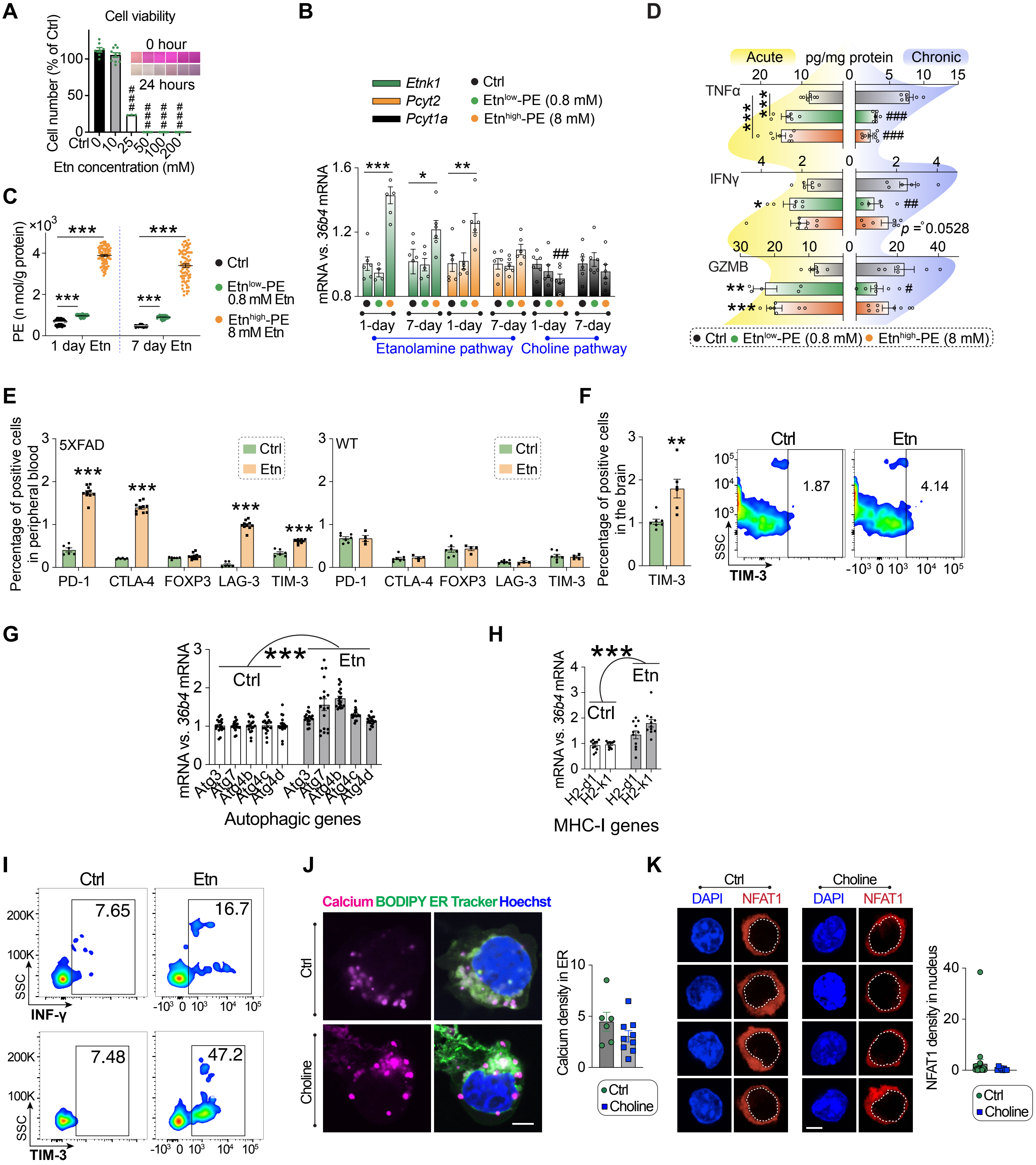

A
Cell viability
Cell number (% of Ctrl)
0 hour
24 hours
Ctrl 0 10 25 50 100 200
Etn concentration (mM)
B
Etnk1
Pcyt2
Pcyt1a
Ctrl
Etn^low-PE (0.8 mM)
Etn^high-PE (8 mM)
mRNA vs. 36b4 mRNA
1-day 7-day 1-day 7-day 1-day 7-day
Etanolamine pathway
Choline pathway
D
Acute
pg/mg protein
Chronic
TNFα
IFNγ
GZMB
p = 0.0528
Ctrl Etn^low-PE (0.8 mM) Etn^high-PE (8 mM)
C
PE (n mol/g protein)
Ctrl
Etn^low-PE 0.8 mM Etn
Etn^high-PE 8 mM Etn
1 day Etn
7 day Etn
E
Percentage of positive cells in peripheral blood
5XFAD
WT
Ctrl
Etn
PD-1 CTLA-4 FOXP3 LAG-3 TIM-3
F
Percentage of positive cells in the brain
TIM-3
Ctrl
Etn
1.87
4.14
SSC
TIM-3
G
mRNA vs. 36b4 mRNA
Ctrl
Etn
Atg3 Atg7 Atg4b Atg4c Atg4d
Autophagic genes
H
mRNA vs. 36b4 mRNA
Ctrl
Etn
H2-d1 H2-k1
MHC-I genes
I
Ctrl
Etn
7.65
16.7
7.48
47.2
SSC
INF-γ
TIM-3
J
Calcium BODIPY ER Tracker Hoechst
Ctrl
Choline
Calcium density in ER
Ctrl
Choline
K
Ctrl
Choline
DAPI NFAT1
NFAT1 density in nucleus
Ctrl
Choline

Figure S11

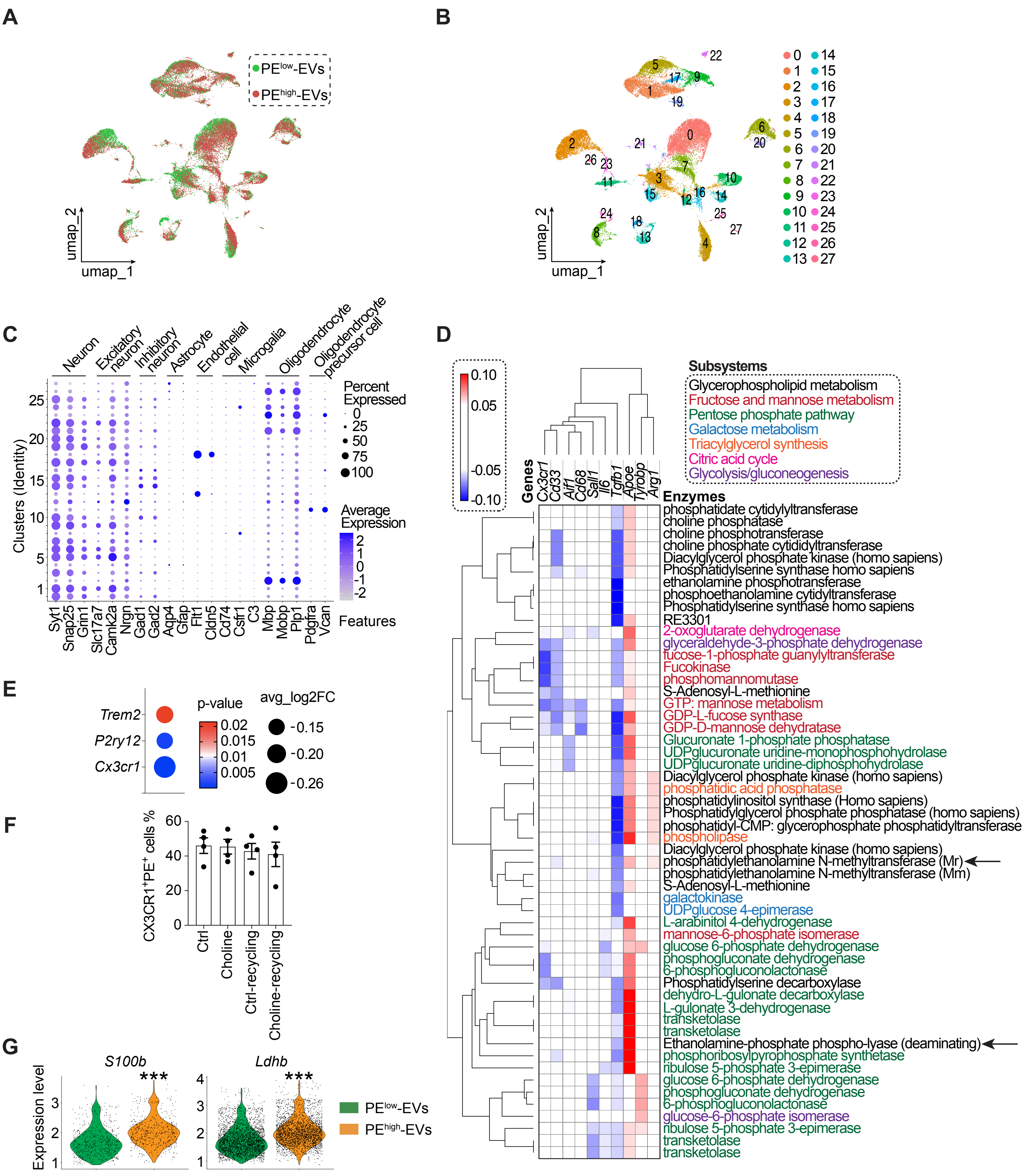
A
PE^low-EVs
PE^high-EVs
umap_2
umap_1
B
0 1 2 3 4 5 6 7 8 9 10 11 12 13 14 15 16 17 18 19 20 21 22 23 24 25 26 27
umap_2
umap_1
C
Neuron
Excitatory neuron
Inhibitory neuron
Astrocyte
Endothelial cell
Microglia
Oligodendrocyte
Oligodendrocyte precursor cell
Percent Expressed
0
25
50
75
100
Average Expression
2
1
0
-1
-2
Clusters (Identity)
Syt1 Snap25 Grin1 Slc17a7 Camk2a Nrgn Gad1 Gad2 Aqp4 Gfap Flt1 Cldn5 Cd74 Csfr1 C3 Mbp Mobp Plp1 Pdgfra Vcan
Features
D
0.10
0.05
-0.05
-0.10
Subsystems
Glycerophospholipid metabolism
Fructose and mannose metabolism
Pentose phosphate pathway
Galactose metabolism
Triacylglycerol synthesis
Citric acid cycle
Glycolysis/gluconeogenesis
Genes
Cx3cr1 Cd33 Aif1 Cd68 Sall1 Il6 Tgfb1 Apoe Tyrobp Arg1
Enzymes
phosphatidate cytidylyltransferase
choline phosphatase
choline phosphotransferase
choline phosphate cytididyltransferase
Diacylglycerol phosphate kinase (homo sapiens)
Phosphatidylserine synthase homo sapiens
ethanolamine phosphotransferase
phosphoethanolamine cytidyltransferase
Phosphatidylserine synthase homo sapiens
RE3301
2-oxoglutarate dehydrogenase
glyceraldehyde-3-phosphate dehydrogenase
fucose-1-phosphate guanylyltransferase
Fucokinase
phosphomannomutase
S-Adenosyl-L-methionine
GTP: mannose metabolism
GDP-L-fucose synthase
GDP-D-mannose dehydratase
Glucuronate 1-phosphate phosphatase
UDPglucuronate uridine-monophosphohydrolase
UDPglucuronate uridine-diphosphohydrolase
Diacylglycerol phosphate kinase (homo sapiens)
phosphatidic acid phosphatase
phosphatidylinositol synthase (Homo sapiens)
Phosphatidylglycerol phosphate phosphatase (homo sapiens)
phosphatidyl-CMP: glycerophosphate phosphatidyltransferase
phospholipase
Diacylglycerol phosphate kinase (homo sapiens)
phosphatidylethanolamine N-methyltransferase (Mr)
phosphatidylethanolamine N-methyltransferase (Mm)
S-Adenosyl-L-methionine
galactokinase
UDPglucose 4-epimerase
L-arabinitol 4-dehydrogenase
mannose-6-phosphate isomerase
glucose 6-phosphate dehydrogenase
phosphogluconate dehydrogenase
6-phosphogluconolactonase
Phosphatidylserine decarboxylase
dehydro-L-gulonate decarboxylase
L-gulonate 3-dehydrogenase
transketolase
transketolase
Ethanolamine-phosphate phospho-lyase (deaminating)
phosphoribosylpyrophosphate synthetase
ribulose 5-phosphate 3-epimerase
glucose 6-phosphate dehydrogenase
phosphogluconate dehydrogenase
6-phosphogluconolactonase
glucose-6-phosphate isomerase
ribulose 5-phosphate 3-epimerase
transketolase
transketolase
E
Trem2
P2ry12
Cx3cr1
p-value
0.02
0.015
0.01
0.005
avg_log2FC
-0.15
-0.20
-0.26
F
CX3CR1+PE+ cells %
60
40
20
0
Ctrl
Choline
Ctrl-recycling
Choline-recycling
G
S100b
Ldhb
***
***
Expression level
PE^low-EVs
PE^high-EVs

Figure S12

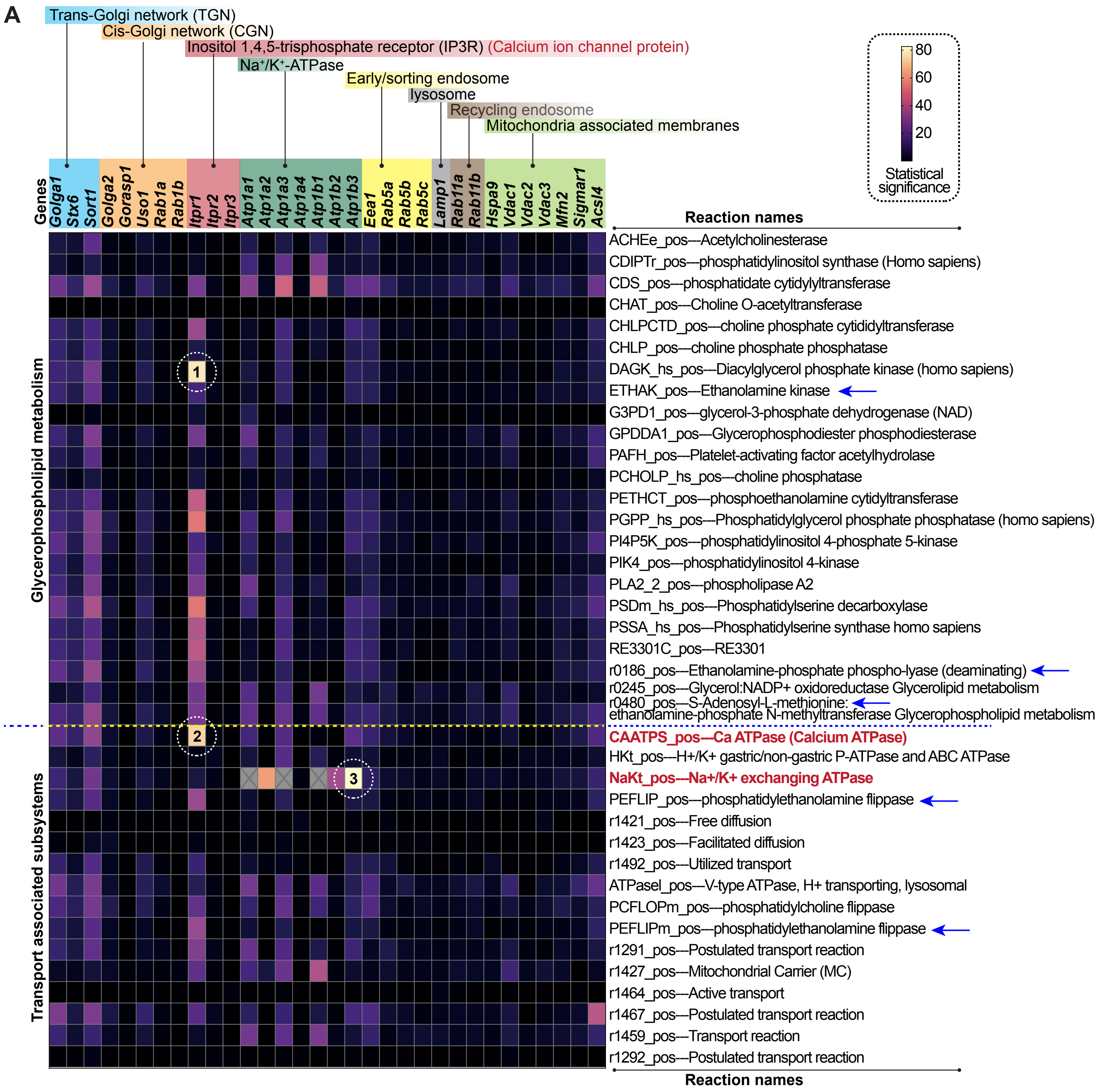

A
Trans-Golgi network (TGN)
Cis-Golgi network (CGN)
Inositol 1,4,5-trisphosphate receptor (IP3R) (Calcium ion channel protein)
Na+/K+-ATPase
Early/sorting endosome
lysosome
Recycling endosome
Mitochondria associated membranes
80
60
40
20
Statistical significance
Genes
Golga1
Stx6
Sort1
Golga2
Gorasp1
Uso1
Rab1a
Rab1b
Itpr1
Itpr2
Itpr3
Atp1a1
Atp1a2
Atp1a3
Atp1a4
Atp1b1
Atp1b2
Atp1b3
Eea1
Rab5a
Rab5b
Rab5c
Lamp1
Rab11a
Rab11b
Hspa9
Vdac1
Vdac2
Vdac3
Mfn2
Sigmar1
Acsl4
Reaction names
Glycerophospholipid metabolism
Transport associated subsystems
1
2
3
ACHEe_pos---Acetylcholinesterase
CDIPTr_pos---phosphatidylinositol synthase (Homo sapiens)
CDS_pos---phosphatidate cytidylyltransferase
CHAT_pos---Choline O-acetyltransferase
CHLPCTD_pos---choline phosphate cytididyltransferase
CHLP_pos---choline phosphate phosphatase
DAGK_hs_pos---Diacylglycerol phosphate kinase (homo sapiens)
ETHAK_pos---Ethanolamine kinase
G3PD1_pos---glycerol-3-phosphate dehydrogenase (NAD)
GPDDA1_pos---Glycerophosphodiester phosphodiesterase
PAFH_pos---Platelet-activating factor acetylhydrolase
PCHOLP_hs_pos---choline phosphatase
PETHCT_pos---phosphoethanolamine cytidyltransferase
PGPP_hs_pos---Phosphatidylglycerol phosphate phosphatase (homo sapiens)
PI4P5K_pos---phosphatidylinositol 4-phosphate 5-kinase
PIK4_pos---phosphatidylinositol 4-kinase
PLA2_2_pos---phospholipase A2
PSDm_hs_pos---Phosphatidylserine decarboxylase
PSSA_hs_pos---Phosphatidylserine synthase homo sapiens
RE3301C_pos---RE3301
r0186_pos---Ethanolamine-phosphate phospho-lyase (deaminating)
r0245_pos---Glycerol:NADP+ oxidoreductase Glycerolipid metabolism
r0480_pos---S-Adenosyl-L-methionine:
ethanolamine-phosphate N-methyltransferase Glycerophospholipid metabolism
CAATPS_pos---Ca ATPase (Calcium ATPase)
HKt_pos---H+/K+ gastric/non-gastric P-ATPase and ABC ATPase
NaKt_pos---Na+/K+ exchanging ATPase
PEFLIP_pos---phosphatidylethanolamine flippase
r1421_pos---Free diffusion
r1423_pos---Facilitated diffusion
r1492_pos---Utilized transport
ATPasel_pos---V-type ATPase, H+ transporting, lysosomal
PCFLOPm_pos---phosphatidylcholine flippase
PEFLIPm_pos---phosphatidylethanolamine flippase
r1291_pos---Postulated transport reaction
r1427_pos---Mitochondrial Carrier (MC)
r1464_pos---Active transport
r1467_pos---Postulated transport reaction
r1459_pos---Transport reaction
r1292_pos---Postulated transport reaction
Reaction names

Figure S13

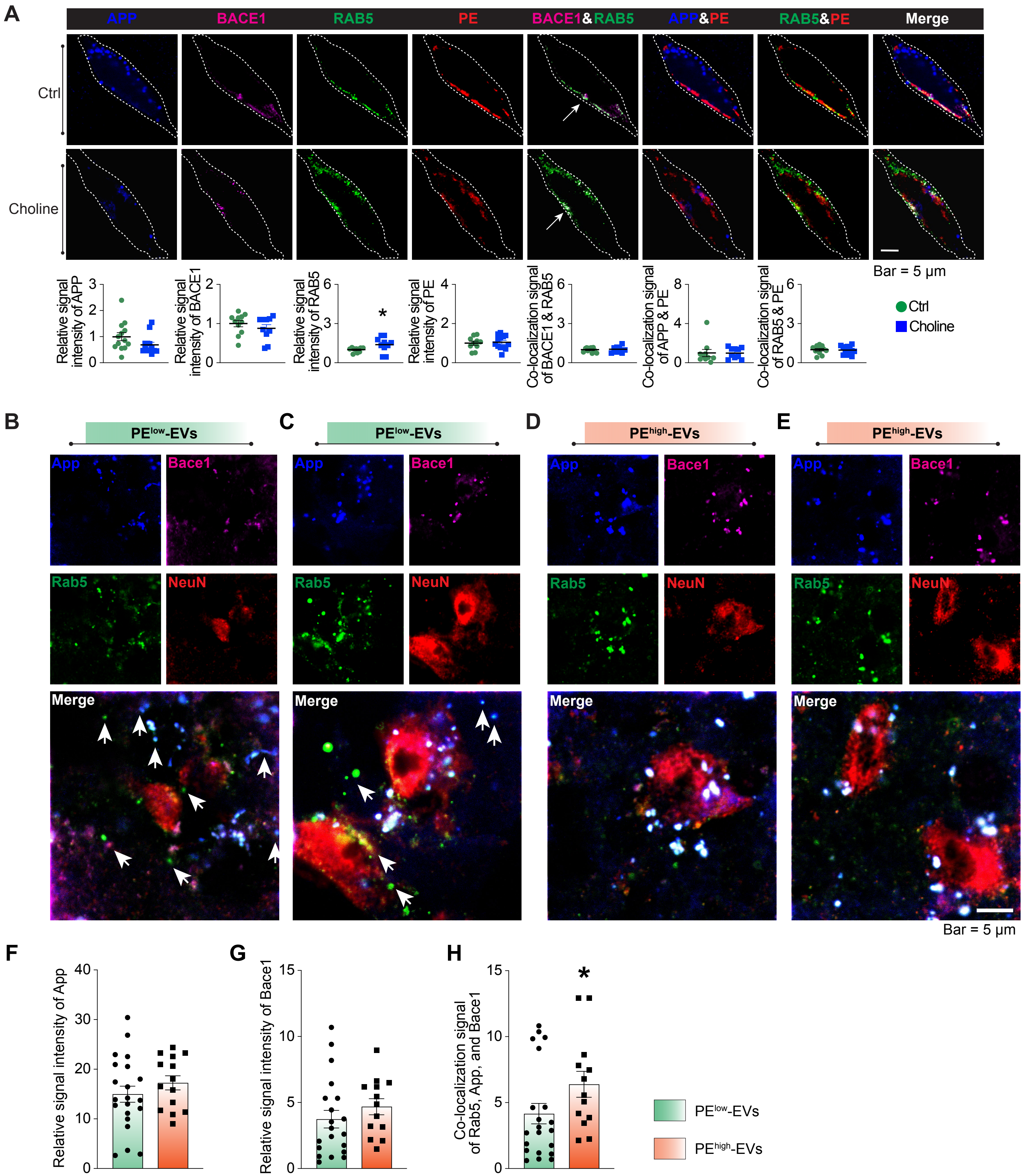

A
APP
BACE1
RAB5
PE
BACE1&RAB5
APP&PE
RAB5&PE
Merge
Ctrl
Choline
Bar = 5 μm
Relative signal intensity of APP
Relative signal intensity of BACE1
Relative signal intensity of RAB5
Relative signal intensity of PE
Co-localization signal of BACE1 & RAB5
Co-localization signal of APP & PE
Co-localization signal of RAB5 & PE
Ctrl
Choline
B
C
D
E
PElow-EVs
PEhigh-EVs
App
Bace1
Rab5
NeuN
Merge
Bar = 5 μm
F
G
H
Relative signal intensity of App
Relative signal intensity of Bace1
Co-localization signal of Rab5, App, and Bace1
PElow-EVs
PEhigh-EVs

Figure S14

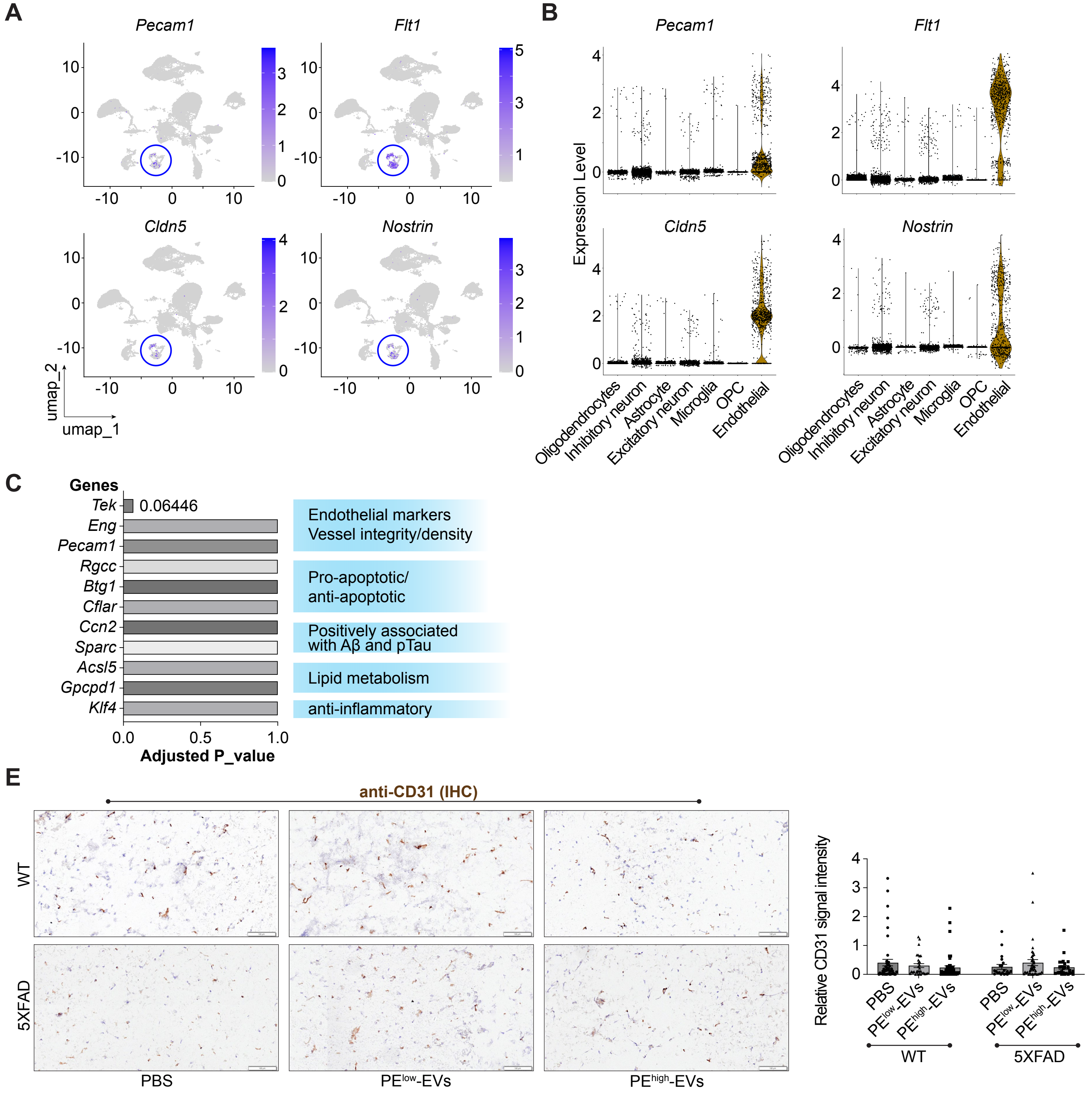

A
Pecam1
Flt1
Cldn5
Nostrin
umap_2
umap_1
B
Expression Level
Oligodendrocytes
Inhibitory neuron
Astrocyte
Excitatory neuron
Microglia
OPC
Endothelial
C
Genes
Tek
0.06446
Eng
Pecam1
Rgcc
Btg1
Cflar
Ccn2
Sparc
Acsl5
Gpcpd1
Klf4
Adjusted P_value
Endothelial markers
Vessel integrity/density
Pro-apoptotic/
anti-apoptotic
Positively associated
with Aβ and pTau
Lipid metabolism
anti-inflammatory
E
anti-CD31 (IHC)
WT
5XFAD
PBS
PElow-EVs
PEhigh-EVs
Relative CD31 signal intensity

Figure S15

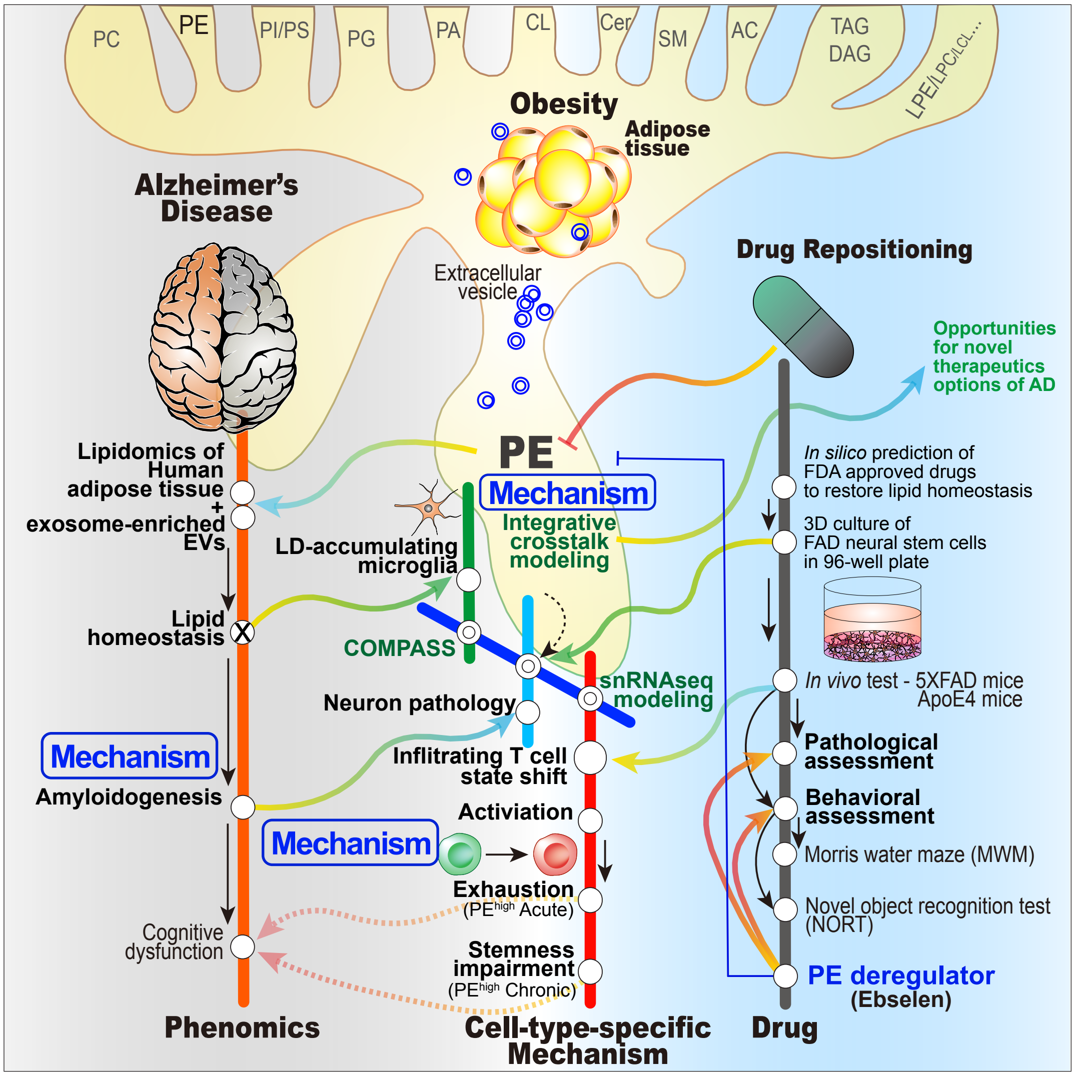

PC
PE
PI/PS
PG
PA
CL
Cer
SM
AC
TAG
DAG
LPE/LPC/LCL...
Obesity
Adipose tissue
Alzheimer's Disease
Extracellular vesicle
Drug Repositioning
Opportunities for novel therapeutics options of AD
PE
Mechanism
Integrative crosstalk modeling
Lipidomics of Human adipose tissue + exosome-enriched EVs
LD-accumulating microglia
In silico prediction of FDA approved drugs to restore lipid homeostasis
3D culture of FAD neural stem cells in 96-well plate
Lipid homeostasis
COMPASS
snRNAseq modeling
Neuron pathology
In vivo test - 5XFAD mice ApoE4 mice
Mechanism
Amyloidogenesis
Inflitrating T cell state shift
Pathological assessment
Activiation
Behavioral assessment
Mechanism
Morris water maze (MWM)
Exhaustion (PEhigh Acute)
Novel object recognition test (NORT)
Cognitive dysfunction
Stemness impairment (PEhigh Chronic)
PE deregulator (Ebselen)
Phenomics
Cell-type-specific Mechanism
Drug
