# Supplementary figures and images for: Obesity-driven phosphatidylethanolamine dysregulation impairs neuroimmune crosstalk and accelerates Alzheimer’s pathogenesis
Source: Mol Neurodegener. 2026 Apr 15;21:25. doi: 10.1186/s13024-026-00943-3 (PMC13159212; doi:10.1186/s13024-026-00943-3)

Uncropped western blot images

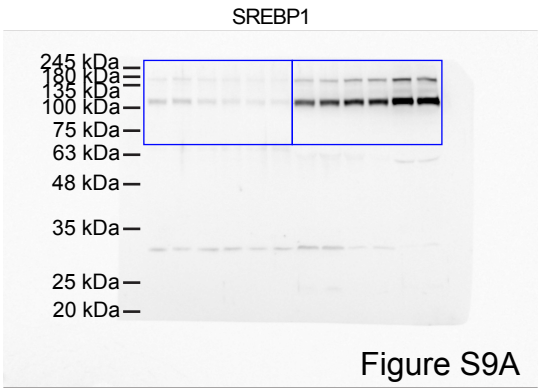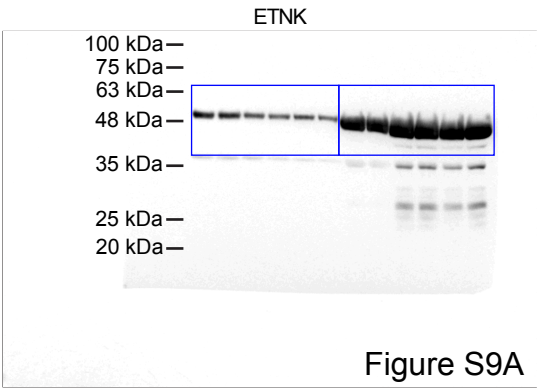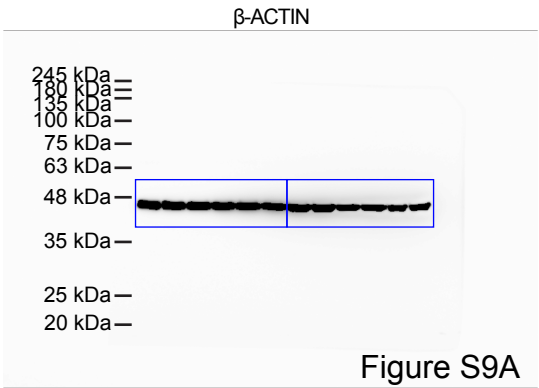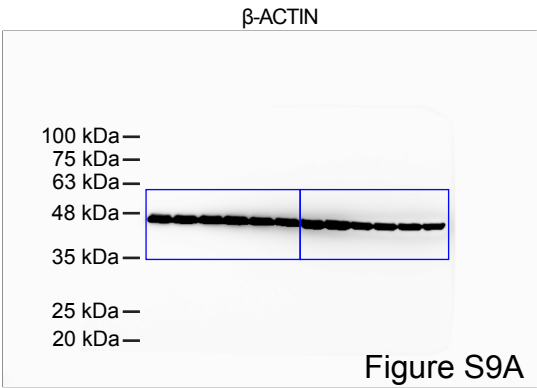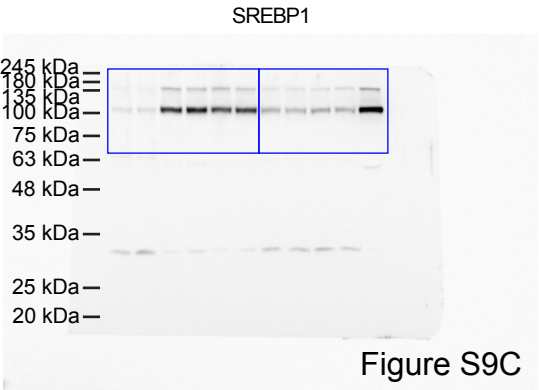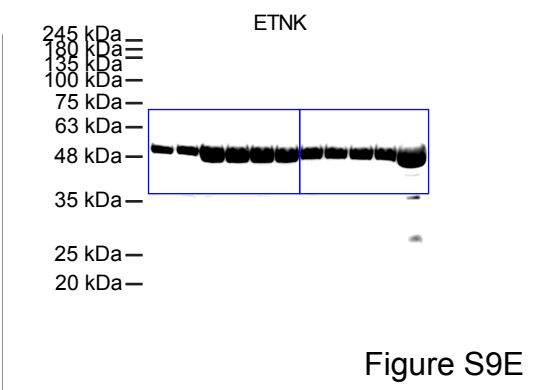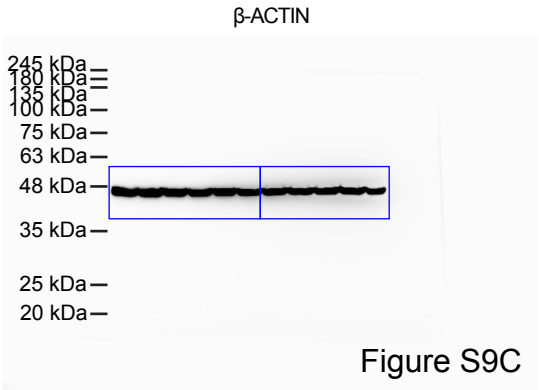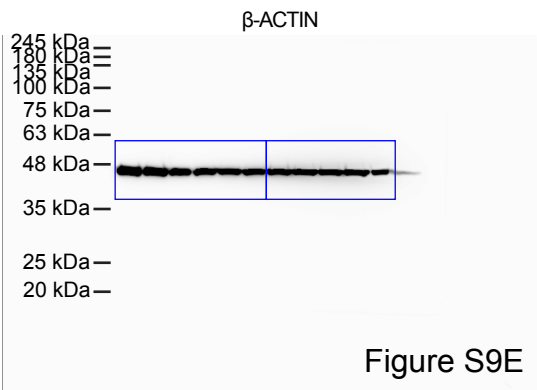

Supplement: Supplementary file 3 — Supplementary Material 3 [file 13024_2026_943_MOESM3_ESM.pdf]
