# Supplementary material for: Obesity-driven phosphatidylethanolamine dysregulation impairs neuroimmune crosstalk and accelerates Alzheimer’s pathogenesis
Source: Mol Neurodegener. 2026 Apr 15;21:25. doi: 10.1186/s13024-026-00943-3 (PMC13159212; doi:10.1186/s13024-026-00943-3)
Supplement: Supplementary file 4 — Supplementary Material 4 [file 13024_2026_943_MOESM4_ESM.docx]

**Fig. S1. Quantitative lipidomic profiling of human white adipose tissue,** **related to Fig. 1.**

**A**. Schematic illustration of human white adipose tissue depots, highlighting subcutaneous adipose tissue (SQA) and visceral adipose tissue (VA) sampling locations.
**B**. Concentric radar (annular) plots depicting the relative abundance of major lipid classes in SQA and VA from obese and non-obese individuals. Lipid classes are grouped into concentric annuli according to their concentration ranges: lipid classes with the highest concentrations are shown in the outer annulus, intermediate concentrations in the middle annulus, and the lowest concentrations in the inner annulus. Each colored segment represents a distinct lipid class as indicated in the legend.
**C**. Quantitative comparison of the mean abundance of 16 lipid classes between obese and non-obese groups in SQA and VA. Bar plots represent lipid levels normalized to protein content (nmol or pmol per mg protein, as indicated). Individual data points represent biological samples.
**D**. Paired comparison of lipid class abundance between SQA and VA within the same individual patient, illustrating depot-specific lipid remodeling. Each line connects matched SQA and VA samples from the same subject, with colors corresponding to lipid classes.
For panel **C**, each dot represents an individual sample (biological replicate). Data are presented as mean ± SEM; *, increase; ^#^, decrease; *^/#^*P* < 0.05. Statistical significance was determined using two-way ANOVA followed by Tukey’s multiple comparisons test. Sample sizes: non-obese (n_sample = 7, N_patient = 6) and obese (n_sample = 12, N_patient = 6).

**Fig. S2. Identification of top PE species enriched in white adipose tissue of obese individuals,** **related to Fig. 2.**

**A**. Statistical screening of phosphatidylethanolamine (PE) lipid species comparing obese and non-obese individuals. Each point represents an individual PE species. Significance was assessed using both two-tailed Student’s t-tests and Wilcoxon rank-sum tests. Magenta points indicate significantly different species based on raw P values below the predefined threshold, while gray points indicate non-significant species.
**B**. Box-and-whisker plots showing normalized abundance of the four PE species identified in panel **A** (α–δ). Boxes represent the interquartile range with the median indicated, whiskers denote the full data range, and individual points represent biological samples from obese and non-obese groups.
**C**. Quantitative comparison of the mean abundance of 25 PE molecular species in subcutaneous adipose tissue (SQA) between obese and non-obese individuals. PE subclasses are annotated as follows: A, alkenylacyl PE; D, diacyl PE; P, plasmalogen PE. Lipid levels are normalized to protein content (nmol per mg protein). Bar plots show mean ± SEM, with individual data points overlaid. Exact P values are indicated for significantly altered species.
**D**. Quantitative comparison of the mean abundance of the same 25 PE molecular species in visceral adipose tissue (VA) between obese and non-obese individuals. Data representation, normalization, and subclass annotations are as described in panel C.
For panels **C** and **D**, each data point represents an individual sample (biological replicate). Data are presented as mean ± SEM. Statistical significance was determined using two-tailed Student’s t-tests followed by Benjamini–Hochberg (BH) correction (*q* < 0.1). Sample sizes: non-obese (n_sample = 7, N_patient = 6) and obese (n_sample = 12, N_patient = 6).

**Fig. S3. In vivo biodistribution of EVs in wild-type mice, related to Fig. 3.**

**A–B**. In vivo and ex vivo fluorescence imaging of wild-type mice following systemic administration of labeled EVs.
**A**. Representative whole-body fluorescence images of mice injected with phosphate-buffered saline (PBS), EVs isolated from chow diet–fed mice (EVs-Chow), or EVs isolated from high-fat diet–fed mice (EVs-HFD). Images were acquired at 1 hour and 4 hours after EV administration to assess temporal biodistribution. Fluorescence signal intensity (red) reflects EV localization. Fur was removed prior to imaging to enhance signal detection and reduce background autofluorescence.
**B**. Ex vivo fluorescence imaging of major organs harvested 6.5 hours after EV administration. Organs analyzed include brain, heart, lung, stomach, spleen, liver, kidney, and bone, as illustrated schematically above the corresponding images. Representative fluorescence images are shown for PBS, EVs-Chow, and EVs-HFD groups, demonstrating organ-specific accumulation of EVs.
EVs were fluorescently labeled using the ExoGlow™-Vivo EV Labeling Kit prior to injection. Images are representative of 3 independent biological replicates.

**Fig. S4. PE^high^-EVs exacerbate AD pathology in 5XFAD mice, related to Fig. 3 & 4.**

**A–B**. Quantification of Alzheimer’s disease–related pathological markers in the brains of 5XFAD and WT mice following short-term EV treatment. Mice received vehicle control (PBS), EVs derived from chow diet–fed mice with low PE content (Chow^EVs^-PE^low^), or EVs derived from high-fat diet–fed mice with high PE content (HFD^EVs^-PE^high^). In 5XFAD mice (A), amyloid-β levels were assessed by mass spectrometry (Aβ42) and immunofluorescence signal density (Aβ40). In WT mice (B), Aβ42 and Aβ40 levels were quantified by ELISA.

**C**. Glucose tolerance tests (GTTs) performed in 5XFAD mice and WT littermates following two-week treatment with PBS, PE^low^-EVs, or PE^high^-EVs. Blood glucose levels were measured at the indicated time points after glucose administration to assess systemic metabolic effects of EV treatment.
(**A**–**B**), n = 4–6 mice per group; (**C**), n = 3 mice per group. In each group, individual mice were treated as biological replicates for statistical analysis. (**A**–**C**), Data are presented as mean ± SEM; *, increase; ^#^, decrease; */^#^*P* < 0.05. (**A**–**B**), Statistical significance was determined using two-tailed Student’s t-tests, with comparisons made only to the corresponding controls at the same time point and no direct comparisons made between chow and HFD groups; (**C**), Statistical significance was determined using two-way repeated-measures ANOVA followed by Dunnett’s multiple comparisons test.

**Fig. S5. Systematic drug repositioning for AD using the SMART framework, related to Fig. 5.**

**A**. Conceptual schematic of the SMART (Systems-based Multi-stage AD Repositioning and Translation) framework, illustrating the integrated pipeline for Alzheimer’s disease (AD) drug discovery and repositioning. The framework combines in silico screening and prediction, three-dimensional (3D) cell model validation, in vivo validation using AD mouse models, and downstream clinical translation.
**B**. Detailed workflow of the SMART drug repositioning pipeline. Starting from a large compound library, candidates are sequentially filtered through in silico screening and ranking, in vitro screening and validation, in vivo validation, and lipid reprogramming analysis. Promising compounds then advance to animal behavioral testing, pathological assessment, long-term biosafety evaluation, and potential clinical trial consideration. Numbers indicate the approximate number of compounds retained at each step.
**C**. Network-based summary of two pilot screening rounds conducted using the SMART framework. Nodes represent individual compounds, and edges indicate shared predicted targets or functional associations. Node colors and shapes denote compound prioritization status and progression across successive screening rounds, as defined in the legend.
**D**. Summary table of representative candidate compounds identified by the SMART framework, including chemical structures and annotated biological functions or molecular targets relevant to AD pathology.
Together, these panels illustrate the scalability, multi-level validation strategy, and translational potential of the SMART framework for systematic AD drug repositioning.

**Fig. S6. In vitro validation of candidate repositioned drugs in a 3D human neural stem cell model of AD, related to Fig. 5.**

**A**. Schematic of the 3D culture system using Alzheimer’s in a dish^TM^ clonal FAD ReNcell^®^ VM human neural stem cell (hNSC) line. Naïve hNSCs of ReN-GA2 clone 3C1 cell line (2.5 × 10^6^ cells) was embedded in Matrigel (bottom layer) and differentiated under conditioned media (top). CMV, cytomegalovirus promoter.

**B**. Representative whole-well immunofluorescence images of cells treated with eight candidate compounds and stained with anti–phospho-tau (AT8) antibody.

**C**. Dose–response curves showing EC_50_ values of selected compounds in reducing tau phosphorylation. Data are from three independent experiments performed in triplicate.

**D**. Representative βIII-tubulin staining in cells treated with vehicle or increasing doses of ebselen (0.08–10 μM) for 3 days.

**E**. Quantification of neurite abundance, neurite length, and neurite intensity, in the 3D disease system. Quantification was performed from 9–16 independent images (fields) per condition.

**F** & **G**. Representative and quantified AT8 staining following 3-day ebselen treatment. Signal thumbnails were generated using ImageJ and vectorized in Adobe Illustrator.

**H**. Representative IF staining with ⍺-p-tau antibody (AT8) in RecN-GA2 clone 3C1 cell line upon vehicle, ebselen^low^ (0.4 µM), and ebselen^high^ (2 µM) treatment for 3 weeks.

**I**. Representative IF staining with ⍺-βIII Tubulin antibody (red) and GFP labeled APP_Swe/Lon_ (green) in RecN-GA2 clone 3C1 cell line upon vehicle, ebselen^low^ (0.4 µM), and ebselen^high^ (2 µM) treatment for 3 weeks. Scale bar, 10 µm.

(**E** and **G**), Quantification was performed from 9–16 independent images (fields) per condition, with each image derived from an independent experiment; independent experiments were treated as biological replicates for statistical analysis. Data are presented as mean ± SEM; *, increase; ^#^, decrease; */^#^*P* < 0.05, **/^##^*P* < 0.01, and ***/^###^*P* < 0.001. Statistical significance was determined using one-way ANOVA followed by Tukey’s post hoc test.

**Fig. S7. Behavioral and toxicological evaluation of ebselen, related to Fig. 5.**

**A**. Schematic of the morris water maze (MWM) platform.

**B** & **C**. Representative trafficking records from MWM assessment.

**D**. Quantification of average speed, visits to the opposite quadrant (Q2), target platform, and non-target quadrants (Q1–Q3), trafficking distance, and time spent in non-target quadrants. Each group consisted of 9–19 mice, with approximately equal numbers of males and females.
**E**. Schematic illustration of the novel object recognition test (NORT).

**F**. Comparison of speed, distance, total time consumption, and visit numbers with target (new) and non-target (habituated) objects in NORT. Each group consisted of 14 mice, with approximately equal numbers of males and females.

**G** & **H**. Body weight monitoring over a 5-week treatment period (n = 7–10 mice/group, both sexes were included, with approximately equal numbers in each group).

**I**. Representative H&E staining of heart, kidney, spleen, and liver.

**J**. Biochemical and ELISA assay of blood urea nitrogen (BUN), creatinine, alanine transaminase (ALT), aspartate transaminase (AST), albumin, globulin, and total protein. n = 4-5 mice/group. Both sexes were included, with approximately equal numbers in each group.

(**D**, **F**, **H**, and **J**), Individual mice were treated as biological replicates for statistical analysis. For panels D, F, and J, each data point represents an individual mouse. (**H**), Body weight was monitored longitudinally in the same cohorts of mice over time (n = 7–10 mice per group). Data are presented as mean ± SEM; *, increase; #, decrease; */^#^*P* < 0.05, **/^##^*P* < 0.01, and ***/^###^*P* < 0.001. Statistical significance was determined using one-way ANOVA followed by Tukey’s post hoc test for (**D**, **F**, and **J**), and two-way repeated-measures ANOVA for (**H**).

**Fig. S8. Ebselen modulates lipid metabolic enzymes and stress-related gene expression in the brain, related to Fig. 5.**

**A-B.** Comparative expression levels of PE and PC associated enzymes in cortices and hippocampi with or without ebselen treatment. n = 6 female mice per group.

**C.** The summary of five to six independent RT-PCR studies on the expression of *Nrf1* (anti-oxidation), *Il6* (inflammation indicator), and *Etnk2* (PE enzyme) in mouse brain upon ebselen treatment. n =5~6 female mice per group.

**D–E.** Lipidomic profiling of brain tissues from control and ebselen-treated mice. (D) Pie chart summarizing the distribution of detected lipid species (n = 840), showing the proportion of species with adjusted P ≤ 0.05 between groups. (E) Violin plots show the relative intensities of selected PE and TAG species measured in brain tissue from control and ebselen-treated mice. Data were obtained from n = 3 female 5XFAD mice per group. Each dot represents the intensity of an individual lipid species. Boxes indicate the median and interquartile range. Adjusted P values (multiple-testing corrected) are shown for each comparison.

**F.** Quantitative RT–PCR analysis of *Srebf1* mRNA expression in the hippocampus and cortex of control and ebselen-treated mice (6 female 5XFAD per group).

**G.** Representative immunofluorescence images showing NFκB (orange) and DAPI (blue) staining in the cortex of control and ebselen-treated mice. Enlarged insets highlight nuclear localization patterns. Quantification of NFκB–DAPI overlap intensity is shown on the right. Scale bar, 50 μm.

**H.** Representative immunofluorescence images of SREBP1 (orange) and DAPI (blue) in the cortex of control and ebselen-treated mice. Right panels show quantification of SREBP1 signal intensity and SREBP1–DAPI overlap. Scale bar, 50 μm.

(**A**–**C**), Each data point represents an independent experiment and was treated as a biological replicate for statistical analysis. (**G** and **H**), Data are derived from 6 female 5XFAD mice (3 control and 3 ebselen-treated), with 1–7 images per mouse (13–18 images in total; each dot represents one image) analyzed per condition; individual mice were treated as biological replicates for statistical analysis. Data are presented as mean ± SEM; *, increase; ^#^, decrease; compared with the corresponding controls; */^#^*P* < 0.05, **/^##^*P* < 0.01, and ***/^###^*P* < 0.001. Statistical significance was determined using unpaired two-tailed Student’s t-tests (**A**–**C** and **F**–**H**). (**D** and **E**), Whole lipid species lipidomics data were analyzed using Benjamini–Hochberg (BH) correction for multiple comparisons.

**Fig. S9. Mechanistic analysis of the NFκB–SREBP1–ETNK signaling axis, related to Fig. 5.**

**A–B**. Immunoblot analysis of SREBP1 and ETNK protein expression in SH-SY5Y cells under the indicated treatment conditions. Cells were treated with vehicle control, ebselen (10 μM), or ebsulfur (10 μM), as indicated. β-ACTIN was used as a loading control. Corresponding densitometric quantification is shown on the right. Data shown represent results from four independent experiments.

**C–D**. Immunoblot analysis of SREBP1 protein levels in SH-SY5Y cells. Lipopolysaccharide (LPS) (1 μg/mL) was used as a canonical activator of NF-κB signaling, while H₂O₂ (100 μM) served as an oxidative stress stimulus. BAY 11-7082 (Bay, 1 μM) was used where indicated as an NFκB pathway inhibitor. β-ACTIN was used as a loading control, and quantification of band intensities is shown. Data shown represent results from four independent experiments.

**E–F**. Immunoblot analysis of ETNK protein expression in SH-SY5Y cells. Insulin (100 nM) was used as an activator of SREBP1 signaling, whereas H₂O₂ was used to induce oxidative stress. Fatostatin (20 μM) was applied as a pharmacological inhibitor of SREBP activation. Corresponding densitometric quantification normalized to β-ACTIN is shown. Data shown represent results from four independent experiments.

**G**. Representative immunofluorescence images showing cellular phosphatidylethanolamine (PE) distribution (red) and nuclear counterstaining with DAPI (blue) in SH-SY5Y cells under the indicated treatment conditions. Quantification of PE signal intensity is shown on the right. Scale bar, 20 μm.

**H–K**. Quantification of PE signal intensity following the indicated treatments in (G). Each data point represents an individual field of view (n = 5–23 fields per condition).

**L–M**. Chromatin immunoprecipitation (ChIP)–qPCR analysis of transcription factor occupancy at the *SREBF1* and *ETNK* promoters. Enrichment was calculated relative to IgG controls using primer sets flanking the transcription start site (TSS). Data shown represent results from four independent experiments.

**N**. In vitro enzymatic assay measuring ADP production from ethanolamine catalyzed by ETNK. The schematic at the top illustrates the rate-limiting step of de novo phosphatidylethanolamine (PE) biosynthesis, in which ethanolamine is phosphorylated by ethanolamine kinase (ETNK) to generate phosphoethanolamine in an ATP-dependent manner. Chemical structures of ebselen and ebsulfur are shown on the right. Bar graphs depict relative ADP production as a readout of ETNK enzymatic activity under the indicated reaction conditions. Reactions were performed in the absence of ATP (–ATP), in complete reaction mixtures (positive control), or in the presence of ebselen (10 μM) or ebsulfur (10 μM). Data shown represent results from four independent experiments.

(**B**, **D**, **F**, **L**, **M**, and **N**), Each data point represents an independent experiment (3–4 independent experiments), and independent experiments were treated as biological replicates for statistical analysis. (**H**–**K**), Data are derived from 5 independent experiments, with 1–5 images per experiment (5–22 images in total; each dot represents one image) analyzed per condition; independent experiments were treated as biological replicates for statistical analysis. All quantitative data are presented as mean ± SEM; *, increase; ^#^, decrease; compared with the corresponding controls; */^#^*P* < 0.05, **/^##^*P* < 0.01, ***/^###^*P* < 0.001, and ****/^####^*P* < 0.0001. Statistical significance was determined using one-way ANOVA followed by Tukey’s multiple comparisons test (**B**, **F**, **L**, and **N**) or unpaired two-tailed Student’s t-tests (**D**, **H**–**K**, and **M**).

**Fig. S10. T cell-intrinsic responses to PE levels and crosstalk with neurons, related to Fig. 6.**

**A**. Cell viability analysis of DO-11-10 cells treated with varying doses of ethanolamine (Etn: 10, 25, 50, 100, and 200 µM). n = 3–10 measurements per group from three independent experiments. Phenolsulfonphthalein colorimetric changes were used to estimate pH variation at 0 and 24 hours post-treatment.

**B**. The summary of three to six independent RT-PCR analyses of indicated key enzymes (*Etnk1*, *Pcyt2*, and *Pcyt1a*) of the *de novo* PE/PC biosynthesis in DO-11-10 T cell model upon acute (1-day) or chronic (7-day) Etn^low/high^-PE exposure.

**C**. ELISA assay of cellular PE levels upon Etn^low^-PE and Etn^high^-PE treatment of DO-11-10 T cell model for 1 day and 7 days. n = 13 measurements for each group from three independent experiments with duplication for each sample.

**D**. ELISA assay of indicated cytokines (TNF-⍺, IFN-𝛾, and GZMB) in (Fig. 6A) 7^th^ column in the supernatant of DO-11-10 T cell model upon Etn^low^-PE and Etn^high^-PE treatment for 1 or 7 days. n = 3~4 measurements for each group from three independent experiments with duplication for each sample.

**E**. Flow cytometry quantification of immune checkpoint receptor–positive cells in peripheral blood from 5XFAD and WT mice (females, 52 weeks old) following Ctrl or Etn treatment. Bar graphs show the percentage of marker-positive cells for PD-1, CTLA-4, FOXP3, LAG-3, and TIM-3. Each dot represents one mouse (approximately n = 5–10 per group), and bars indicate mean ± SEM. Statistical significance is denoted by asterisks.

**F**. Flow cytometry analysis of TIM-3–positive cells in the brain of female 5XFAD mice (52 weeks old) following Ctrl or Etn treatment. Left, quantification of the percentage of TIM-3–positive cells; each dot represents one mouse (approximately n = 5–7 per group), with bars indicating mean ± SEM. Right, representative flow cytometry density plots illustrating TIM-3 staining in Ctrl and Etn groups (percentage values shown within gates). Statistical significance is denoted by asterisks.

**G-H.** Summary of three to six independent RT-PCR analyses assessing the expression of autophagy-related genes (*Atg3, Atg7, Atg4b, Atg4c, Atg4d*) and MHC-I genes (H2-d1 and H2-k1) in primary neurons with or without Etn treatment.

**I.** Representative flow cytometry of DO-11-10 cells co-cultured with primary neurons, stained for IFN-γ (TCR activation marker) and TIM-3 (T cell exhaustion marker).
**J**. Representative IF staining and quantification of ex vivo human peripheral blood CD8+ T cells with/without choline treatment using Oregon Green™ 488 BAPTA-1, BODIPY ER tracker, and Hoechst. Scale bar, 5 µm.

**K**. IF staining and quantification of CD8+ T cells stained with α-NFAT1 antibody and DAPI, with/without choline treatment. Dashed lines indicate nuclear contours. Scale bar, 5 µm.

(**A**, **C**, and **D**), Data are derived from three independent experiments. For panels A and C, each data point represents an individual measurement, whereas for panel D, each data point represents a technical replicate (duplication) within an independent experiment. (**B**, **G**, and **H**), Data are derived from three to six independent experiments. For panel B, each data point represents an independent experiment, whereas for panels G and H, each data point represents a value from technical replicates (duplication or triplication) within an independent experiment. (**E** and **F**), Each data point represents an individual mouse. (**J** and **K**), Data are derived from three independent experiments, with a total of 6–14 images analyzed per condition (each dot represents one image). Across all panels, independent experiments or individual mice were treated as biological replicates for statistical analysis. Data are presented as mean ± SEM; *, increase; ^#^, decrease; */^#^*P* < 0.05, **/^##^*P* < 0.01, and ***/^###^*P* < 0.001. Statistical significance was determined using one-way ANOVA followed by Tukey’s post hoc test for (**A**–**D**) and unpaired two-tailed Student’s t-tests for (**E**, **F**, **G**, **H**, **J**, and **K**).

**Fig. S11. Single-nucleus RNA-seq analysis of 5XFAD brain treated with PE^high^-EVs and PE^low^-EVs, related to Fig. 6.**

**A**. Uniform manifold approximation and projection (UMAP) plot of 17,905 cells acquired from the PE^low^-EVs group and 19,654 cells from the PE^high^-EVs group.

**B**. UMAP plot of 37,559 cells grouped into 28 clusters from 5XFAD brains treated with engineered EVs. These 28 clusters were further consolidated into 7 main clusters (Fig. **6H**) based on cluster-specific markers (Table S4).

**C**. Dot plot of the expression levels of canonical marker genes for cluster 0-27 in (**B**).

**D.** Spearman correlation of COMPASS scores with the expression of microphage dysfunction genes. Non-significant correlations shown in white. Phosphatidylethanolamine N-methyltransferase is an enzyme primarily responsible for the biosynthesis of phosphatidylcholine (PC) from phosphatidylethanolamine (PE) through methylation reactions. Ethanolamine-phosphate phosphor-lyase (deaminating) is an enzyme that catalyzes the deamination and cleavage of ethanolamine phosphate (Etn-P) into acetaldehyde, phosphate, and ammonia. This enzyme indirectly maintains phospholipid balance by regulating the availability of precursors for phosphatidylethanolamine (PE) synthesis. Disruptions in its function may affect lipid homeostasis, compromising cellular membrane integrity and function and altering membrane dynamics and signaling.

**E**. Dot plot of *Trem2*, *P2ry12*, and *Cx3cr1* in the microglia cluster.

**F**. CX3CR1 recycling assay in primary microglia with or without Choline treatment.

**G**. Violin plots showing the expression levels of *S100b,* and *Ldhb* in the excitatory neuron cluster.

(**F**), Data are derived from four independent experiments, and each data point represents an independent experiment, which was treated as a biological replicate for statistical analysis. Data are presented as mean ± SEM. Statistical significance was determined using one-way ANOVA followed by Tukey’s post hoc test. (**G**), Data are presented as violin plots. Statistical significance was determined using the Wilcoxon rank-sum test; ****P* < 0.001.

**Fig. S12. Correlation between COMPASS-inferred activity and membrane remodeling–associated genes, related to Fig. 7.**

Genes associated with the trans-Golgi network (TGN), cis-Golgi network (CGN), inositol 1,4,5-trisphosphate receptor (IP3R), Na^+^/K^+^-ATPase, early/sorting endosome, lysosome, recycling endosome, and mitochondrial-associated membranes were used to calculate correlations with COMPASS-derived results. These subsystems encompass glycerophospholipid metabolism and transport-associated subsystems. Among the correlation results, three pixels exhibited the highest statistical significance. Specifically, the second and third pixels - representing calcium ion channel proteins and sodium-potassium-calcium channel genes, respectively - showed strong statistical significance with “Transport-associated subsystems”. The blue arrows represent enzymes associated with PE metabolism.

**Fig. S13. PE levels modulate neuronal APP and BACE1 endosomal localization, related to Fig. 7.**

**A.** Representative IF staining and the statistics with ⍺-APP, ⍺-BACE1, ⍺-RAB5 antibodies and ⍺-PE probe in HEK293-APP695-WT cells with or without choline treatment. Scale bar, 5 µm. The white dashed line marks the contour just external to the cell membrane, rather than the plasma membrane itself.

**B–E**. Representative confocal images of brain sections from female mice treated with PE^low^-EVs (B, C) or PE^high^-EVs (D, E). Brain sections were immunostained for APP (blue), BACE1 (magenta), Rab5 (green; early endosome marker), and NeuN (red; neuronal marker). Merged images illustrate the spatial distribution of APP and BACE1 relative to Rab5-positive endosomal compartments in NeuN-positive neurons. Arrows indicate representative regions showing APP and BACE1 signals with minimal overlap with Rab5-positive endosomes. Scale bar, 5 μm.

**F-G**. Quantification of relative signal intensity of APP (F) and BACE1 (G) in neurons following treatment with PE^low^-EVs or PE^high^-EVs.

**H**. Quantification of co-localization signal among Rab5, APP, and BACE1.

(**A** and **F**–**G**), Data are derived from three independent experiments, with 4–8 images per experiment (10–21 images in total; each dot represents one image) analyzed per condition; independent experiments were treated as biological replicates for statistical analysis. For panels **F**–**G**, n = 3 mice per group. Data are presented as mean ± SEM; *, increase; **P* < 0.05 compared with the corresponding controls. Statistical significance was determined using unpaired two-tailed Student’s t-tests.

**Fig. S14. EV treatment maintains endothelial homeostasis and blood–brain barrier integrity, related to Fig. 7.**

**A**. UMAP feature plots showing expression of canonical endothelial markers *Pecam1*, *Flt1*, *Cldn5*, and *Nostrin*, highlighting a robust endothelial cluster (blue circles).

**B**. Violin plots demonstrating cell-type–specific enrichment of these endothelial markers, confirming clear separation of endothelial cells from other brain cell populations.

**C**. Differential expression analysis of the endothelial cluster showing no significant changes (adjusted P values) in genes related to endothelial identity, vessel integrity/density, apoptosis pathways, lipid metabolism, or inflammatory signaling.

**E**. Immunohistochemical assessment of vascular density in WT and 5XFAD mouse cortex after EV treatment. Representative cortical IHC images showing CD31 staining (brown signal), a marker of vascular integrity and vessel density, in WT and 5XFAD mice treated with PBS, PE^low^–EVs, or PE^high^–EVs for two weeks. Quantification (right) shows comparable CD31 signal intensity across treatment groups. Scale bars: 100 μm. Data are derived from three independent mice per condition, with 10–20 images per mouse (21–56 images in total; each dot represents one image) analyzed per condition; individual mice were treated as biological replicates for statistical analysis. Data are presented as mean ± SEM. Statistical significance was determined using two-way ANOVA followed by Dunnett’s multiple comparisons test.

**Fig. S15. Proposed model of high PE levels from obese adipose tissue driving AD pathology.**

We analyzed lipid composition in adipose tissue obtained through surgical biopsies from midlife obese and non-obese individuals. Principal component analysis of the lipidomic profiles revealed a significant enrichment of PE in the obese group. Increased PE flux (PE^high^) was shown to promote lipid droplet LD accumulation in AD models and to enhance amyloidogenesis in the AD brain, thereby contributing to disease progression. By contrast, restoring PE homeostasis improved cognitive performance, as demonstrated by neuropathological and behavioral evaluations in AD mice.

At the mechanistic level, high PE levels activated autophagic, immune, and membrane remodeling pathways, leading to spatiotemporal disruption of intracellular signaling such as calcium dynamics, destabilization of essential membrane structures including the endoplasmic reticulum, and T cell exhaustion through both neuron-dependent and neuron-independent processes. High PE levels also induced immune and membrane remodeling alterations in microglia and excitatory neurons, resulting in microglial dysfunction and increased production of neurotoxic Aβ, ultimately causing neuronal damage.

These results highlight PE-driven biological processes, particularly those involving membrane remodeling, as important contributors to AD pathology. They also point to the therapeutic relevance of targeting PE-associated signaling pathways, including those regulating membrane dynamics, the calcineurin–NFAT axis, and communication between distinct brain cell types.
